# Germanyl triazoles as a platform for CuAAC diversification and chemoselective orthogonal cross-coupling

**DOI:** 10.3762/bjoc.20.265

**Published:** 2024-12-05

**Authors:** John M Halford-McGuff, Thomas M Richardson, Aidan P McKay, Frederik Peschke, Glenn A Burley, Allan J B Watson

**Affiliations:** 1 EaStCHEM, School of Chemistry, University of St Andrews, North Haugh, St Andrews, Fife, KY16 9ST, UK; 2 Department of Pure & Applied Chemistry, University of Strathclyde, Glasgow, G1 1XL, UK

**Keywords:** chemoselectivity, click chemistry, copper, germanium, triazole

## Abstract

We report the synthesis of germanyl triazoles formed via a copper-catalysed azide–alkyne cycloaddition (CuAAC) of germanyl alkynes. The reaction is often high yielding, functional group tolerant, and compatible with complex molecules. The installation of the Ge moiety enables further diversification of the triazole products, including chemoselective transition metal-catalysed cross-coupling reactions using bifunctional boryl/germyl species.

## Introduction

**Scheme 1:**
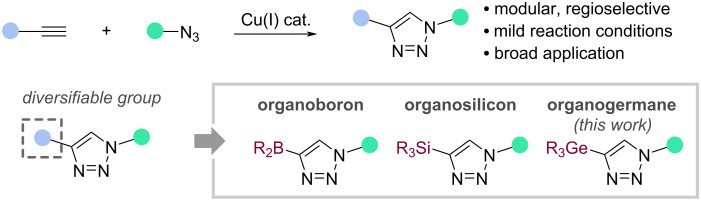
The CuAAC reaction and installation of functional groups for product diversification.

Since its inception, click chemistry has been established as a powerful approach for molecule synthesis. Strategies within click chemistry include several widely used reactions such as the (hetero-)Diels–Alder reaction [1–2], alkene hydrothiolation [3], and an array of amide-bond-forming chemistries [4]. However, by virtue of the access to alkyne and azide precursors and the formation of a single 1,4-disubstituted triazole product, the copper-catalysed azide–alkyne cycloaddition (CuAAC) remains the archetypal click reaction (Scheme 1) [5].

The reaction has shown applicability on small and large scale, as well as under flow conditions [6], and extensive scope across a range of benign solvent conditions [7–10]. In addition, the CuAAC reaction uses inexpensive Cu catalysts [11], is insensitive towards oxygen and water [12–13], and consistently delivers high yields and (where relevant) enantioselectivities [8–10,14–19]. As such, the reaction has been used extensively throughout drug discovery [20–21], chemical biology [22–23], and materials science [24–27]. Orthogonal alkyne reactivity can also be observed under certain systems [28–30]. The reaction typically uses a Cu(II) pre-catalyst, which is converted to a mechanistically-required Cu(I) species in situ through the addition of a reductant (e.g., sodium ascorbate, NaAsc) [31–32], or via Glaser–Hay alkyne homocoupling [33–34].

The mild and accessible nature of the CuAAC reaction has allowed the use of azide or alkyne components that bear functional groups for subsequent product diversification (Scheme 1). For example, protected alkynylboron reagents can be employed [35–37], such as *N*-methyliminodiacetic acid (MIDA)boronate esters [38], potassium trifluoroborates [39], and others [40–42]. Similarly, organosilicon reagents have proven useful in various Cu- and Pd-catalysed C–X-bond-forming strategies [43–51], including widespread use across several CuAAC methodologies [52–54].

Germanium-based functional groups have recently emerged as highly useful components for transition-metal-catalysed cross-couplings. Schoenebeck and co-workers have shown that Ge-based compounds are versatile reagents within chemoselective cross-coupling processes for the formation of a variety of C–C and C–X bonds [55–63]. Importantly, these transformations can take place in the presence of borylated functional groups, allowing orthogonal cross-coupling, whilst also offering excellent stability compared to boron-based reagents [57–67].

Based on their utility and stability, germanium units could therefore be useful within CuAAC reactions and offer potential as functional handles for downstream elaboration of CuAAC products. To date, the main use of germanyl alkynes in (3 + 2) cycloadditions has been limited to a small number of Huisgen (non-Cu-catalysed) reactions [68–69]. Zaitsev and co-workers reported the synthesis and CuAAC reactions of a dialkynyl germane to access 1,2-bis(triazolyl)tetraphenyldigermanes [70]. Here, we report the development of germanyl alkynes as CuAAC components, with exploration of their scope and downstream diversification.

## Results and Discussion

**Scheme 2:**
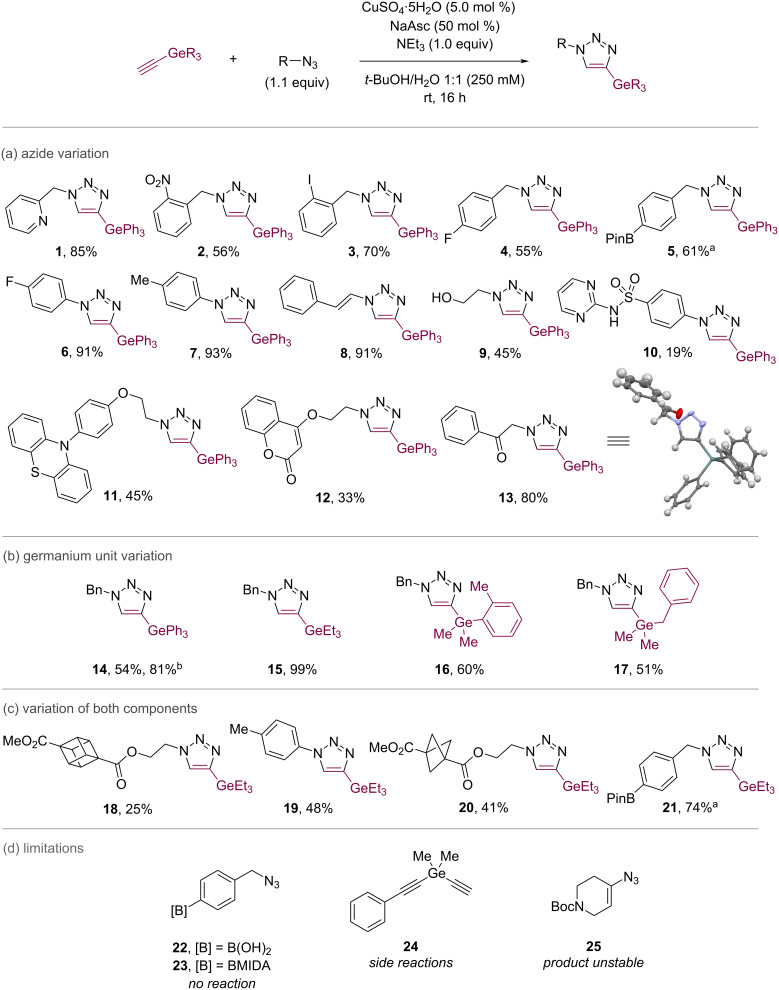
Scope of germanyl acetylene CuAAC. Alkyne (1.0 equiv), azide (1.1 equiv), CuSO_4_·5H_2_O (5.0 mol %), NaAsc (50 mol %), NEt_3_ (1.0 equiv), *t*-BuOH/H_2_O 1:1 (250 mM), N_2_, rt, 16 h. Isolated yields. ^a^Reaction performed with CsF (2.0 equiv) as an additive. ^b^Reaction performed at rt for 64 h.

We undertook an exploratory survey of CuAAC reaction conditions using benzyl azide and triethylgermanyl acetylene (see Supporting Information File 1). The most effective conditions were found to be based on the classical combination of CuSO_4_/NaAsc, with optimisation (see Supporting Information File 1) delivering the general conditions shown in Scheme 2. These afforded a clean conversion to the desired triazole products **1**–**21** without any observable degermylation or other side reactions that could be anticipated based on transmetalation to Cu [43].

The generality of the CuAAC process was explored using a range of azides (Scheme 2a), with variation of the germanyl alkyne motif (Scheme 2b), and with variation of both components (Scheme 2c). In general, the CuAAC process worked effectively, tolerating the functional groups for which the CuAAC is well-known – in all cases the remaining mass balance was accounted for by the germanyl acetylene, suggesting sluggish CuAAC reactivity compared to other alkynes, which typically require much shorter reaction times. Extending the reaction time provided a higher conversion to the product **14**. Yields were observed to be greater for aryl azides (e.g., **4** vs **6**). Heterocycles such as pyridine (**1**), pyrimidine (**10**), phenothiazine (**11**), and chromene (**12**) were tolerated. Benzylic azides were accommodated including those bearing nitro (**2**), iodo (**3**), and boronic ester groups (**5**, **21**). Strained rings were effective including cubane (**18**) and bicyclopentane (**20**). While **18** and **20** were isolated in lower yield, no evidence of ring opening was observed and the starting material could be recovered in each case, consistent with observations by Lam and MacMillan [71–72]. Variation of the steric and electronic parameters of the germanyl acetylene was straightforward (**14**–**17**; Scheme 2b). Several limitations were observed (Scheme 2d): benzyl azides displaying an arylboronic acid and MIDA ester (**22** and **23**) gave no reaction, side reactions were observed with a dialkynyl germane (**24**), and the product derived from azide **25** was unstable to purification.

**Scheme 3:**
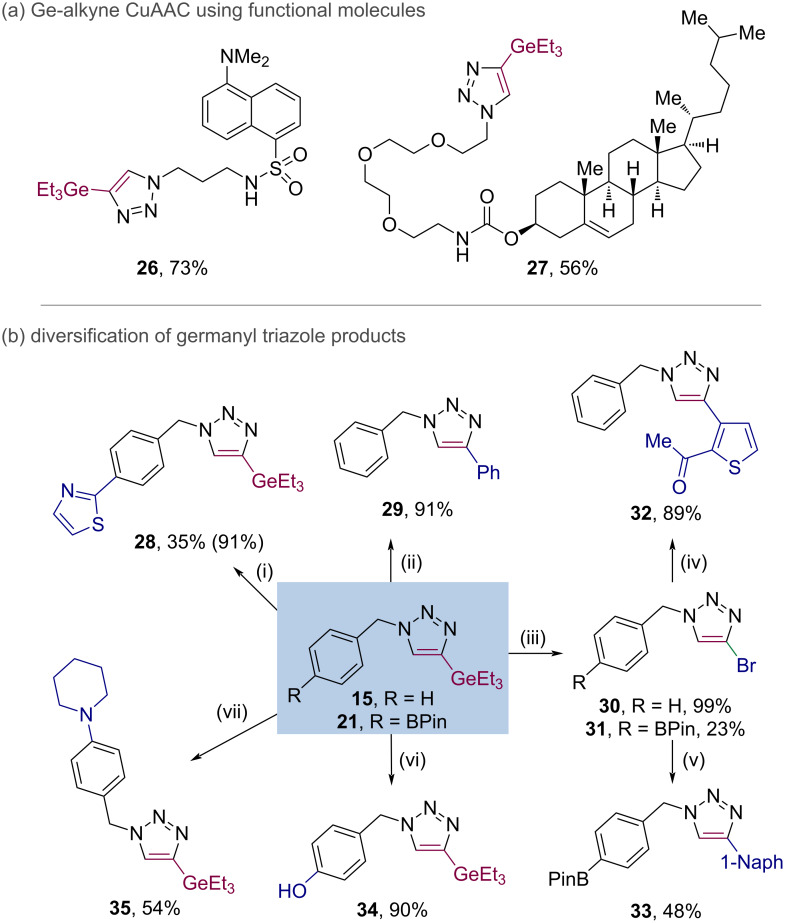
(a) Application of Ge-alkyne CuAAC to functional molecules. (b) Functionalisation of germylated triazoles. Isolated yields unless stated. (i) Pd(PPh_3_)_4_ (10 mol %), 2-bromothiazole (1.2 equiv), KCl (3.0 equiv), PhMe/EtOH 4:1, N_2_, 100 °C, 16 h. NMR yield in parentheses. (ii) Pd_2_(dba)_3_ (2.5 mol %), iodobenzene (1.5 equiv), AgBF_4_ (1.5 equiv), DMF, N_2_, 80 °C, 16 h. (iii) NBS (2.0 equiv), DMF, air, rt, 2 h. (iv) Pd(dtbpf)Cl_2_ (10 mol %), 2-acetylthiophen-3-ylboronic acid (1.2 equiv), K_3_PO_4_ (2.0 equiv), iPrOH/H_2_O 3:4, N_2_, 85 °C, 16 h. (v) Pd(dtbpf)Cl_2_ (2.0 mol %), 1-naphthylzinc bromide (1.2 equiv), THF, N_2_, 45 °C, 16 h. (vi) Cu(OAc)_2_·H_2_O (30 mol %), B(OH)_3_ (2.0 equiv), DBU (2.0 equiv), MeCN, air, 70 °C, 24 h. (vii) Cu(OAc)_2_·H_2_O (30 mol %), B(OH)_3_ (2.0 equiv), piperidine (2.0 equiv), MeCN, air, 70 °C, 24 h. See Supporting Information File 1 for full details.

To further demonstrate the compatibility and utility of germanyl alkynes in CuAAC reactions, we applied the CuAAC process to more challenging substrates. Using fluorophore- and cholesterol-derived azides, coupling with the triethylgermanyl alkyne delivered the expected products **26** and **27**, respectively, in good yield, enabling possible downstream diversification of these functional molecules of relevance to chemical biology (Scheme 3a).

The utility of the germanyl triazole products was then assessed by subsequent derivatisation of exemplar compounds **15** and **21** (Scheme 3b). Chemoselective Suzuki–Miyaura cross-coupling of the BPin moiety in **21** was straightforward, giving **28** in excellent yield [73]. Similarly, cross-coupling of the GeEt_3_ moiety in **15** under conditions developed by Schoenebeck and co-workers gave **29** [57]. Bromodegermanylation using NBS employing conditions from Schoenebeck gave bromotriazoles **30** and **31** in moderate to excellent yield [62]. These could then undergo Suzuki–Miyaura cross-coupling to give **32** or chemoselective Negishi coupling to give **33** [74]. Finally, BPin **21** could be oxidised to the phenol derivative **34** or cross-coupled with piperidine under Chan–Lam conditions to give the aniline derivative **35** in good yield [75].

## Conclusion

In summary, we have developed a general method towards the synthesis of germanyl triazoles. These reagents are generally compatible but seem to be less reactive than other classes of alkyne. The germanyl alkyne CuAAC is applicable to functional group-rich molecules, opening opportunities for downstream diversification by chemoselective functionalisation strategies [76]. The germanyl group installed in the triazole products can be used as a reactive handle for further diversification including cross-coupling reactions.

## Supporting Information

The research data supporting this publication can be accessed at https://doi.org/10.17630/53959471-068e-483e-bcd4-920e6761926b and CCDC 2355570 contains the supplementary crystallographic data for this study.

File 1 Characterization data and copies of NMR spectra.

File 2 Crystallographic information file (cif) for compound **13**.

File 3 Checkcif file for compound **13**.

## Data Availability

Data generated and analyzed during this study is openly available at https://doi.org/10.17630/53959471-068e-483e-bcd4-920e6761926b.

## References

[1] Tasdelen M A (2011). Polym Chem.

[2] Eschenbrenner‐Lux V, Kumar K, Waldmann H (2014). Angew Chem, Int Ed.

[3] Hoyle C E, Bowman C N (2010). Angew Chem, Int Ed.

[4] Li H, Aneja R, Chaiken I (2013). Molecules.

[5] Kolb H C, Finn M G, Sharpless K B (2001). Angew Chem, Int Ed.

[6] Hatit M Z C, Reichenbach L F, Tobin J M, Vilela F, Burley G A, Watson A J B (2018). Nat Commun.

[7] Melo A, Monteiro L, Lima R M F, de Oliveira D M, de Cerqueira M D, El-Bachá R S (2011). Oxid Med Cell Longevity.

[8] Meldal M, Tornøe C W (2008). Chem Rev.

[9] Haldón E, Nicasio M C, Pérez P J (2015). Org Biomol Chem.

[10] García-Álvarez J, Díez J, Gimeno J (2010). Green Chem.

[11] Wang K, Bi X, Xing S, Liao P, Fang Z, Meng X, Zhang Q, Liu Q, Ji Y (2011). Green Chem.

[12] Fu F, Martinez A, Wang C, Ciganda R, Yate L, Escobar A, Moya S, Fouquet E, Ruiz J, Astruc D (2017). Chem Commun.

[13] Nebra N, García-Álvarez J (2020). Molecules.

[14] Vala D P, Vala R M, Patel H M (2022). ACS Omega.

[15] Cook T L, Walker J A, Mack J (2013). Green Chem.

[16] Girard C, Önen E, Aufort M, Beauvière S, Samson E, Herscovici J (2006). Org Lett.

[17] Chtchigrovsky M, Primo A, Gonzalez P, Molvinger K, Robitzer M, Quignard F, Taran F (2009). Angew Chem, Int Ed.

[18] Zhu R-Y, Chen L, Hu X-S, Zhou F, Zhou J (2020). Chem Sci.

[19] Liu E-C, Topczewski J J (2019). J Am Chem Soc.

[20] Lal K, Yadav P, Kumar A, Kumar A, Paul A K (2018). Bioorg Chem.

[21] Rani A, Singh G, Singh A, Maqbool U, Kaur G, Singh J (2020). RSC Adv.

[22] Wright M H, Sieber S A (2016). Nat Prod Rep.

[23] Sapienza P J, Currie M M, Lancaster N M, Li K, Aubé J, Goldfarb D, Cloer E W, Major M B, Lee A L (2021). ACS Chem Biol.

[24] Döhler D, Michael P, Binder W H (2017). Acc Chem Res.

[25] Meldal M (2008). Macromol Rapid Commun.

[26] Pacini A, Nitti A, Vitale M, Pasini D (2023). Int J Mol Sci.

[27] Zaccaria C L, Cedrati V, Nitti A, Chiesa E, Martinez de Ilarduya A, Garcia-Alvarez M, Meli M, Colombo G, Pasini D (2021). Polym Chem.

[28] Hatit M Z C, Sadler J C, McLean L A, Whitehurst B C, Seath C P, Humphreys L D, Young R J, Watson A J B, Burley G A (2016). Org Lett.

[29] Hatit M Z C, Seath C P, Watson A J B, Burley G A (2017). J Org Chem.

[30] Seath C P, Burley G A, Watson A J B (2017). Angew Chem, Int Ed.

[31] Rodionov V O, Fokin V V, Finn M G (2005). Angew Chem, Int Ed.

[32] Rostovtsev V V, Green L G, Fokin V V, Sharpless K B (2002). Angew Chem, Int Ed.

[33] Hein J E, Fokin V V (2010). Chem Soc Rev.

[34] Bunschoten R P, Peschke F, Taladriz-Sender A, Alexander E, Andrews M J, Kennedy A R, Fazakerley N J, Lloyd Jones G C, Watson A J B, Burley G A (2024). J Am Chem Soc.

[35] Huang J, Macdonald S J F, Harrity J P A (2009). Chem Commun.

[36] Huang J, Macdonald S J F, Cooper A W J, Fisher G, Harrity J P A (2009). Tetrahedron Lett.

[37] Dai C, Cheng Y, Cui J, Wang B (2010). Molecules.

[38] Grob J E, Nunez J, Dechantsreiter M A, Hamann L G (2011). J Org Chem.

[39] Jung S h, Choi K, Pae A N, Lee J K, Choo H, Keum G, Cho Y S, Min S-J (2014). Org Biomol Chem.

[40] Zu B, Guo Y, He C (2021). J Am Chem Soc.

[41] Van Belois A, Maar R R, Workentin M S, Gilroy J B (2019). Inorg Chem.

[42] Li J, Tanaka H, Imagawa T, Tsushima T, Nakamoto M, Tan J, Yoshida H (2024). Chem – Eur J.

[43] Lam P Y S, Deudon S, Hauptman E, Clark C G (2001). Tetrahedron Lett.

[44] Denmark S E, Smith R C, Chang W-T T, Muhuhi J M (2009). J Am Chem Soc.

[45] Denmark S E, Regens C S (2008). Acc Chem Res.

[46] Hirabayashi K, Mori A, Kawashima J, Suguro M, Nishihara Y, Hiyama T (2000). J Org Chem.

[47] Nakao Y, Takeda M, Matsumoto T, Hiyama T (2010). Angew Chem, Int Ed.

[48] Hagiwara E, Gouda K-i, Hatanaka Y, Hiyama T (1997). Tetrahedron Lett.

[49] Hatanaka Y, Hiyama T (1988). J Org Chem.

[50] Denmark S E, Wehrli D (2000). Org Lett.

[51] Denmark S E, Choi J Y (1999). J Am Chem Soc.

[52] Yamamoto K, Kanezashi M, Tsuru T, Ohshita J (2017). Polym J.

[53] Venkatesh G B, Hari Prasad S (2015). Phosphorus, Sulfur Silicon Relat Elem.

[54] Li L, Shang T, Ma X, Guo H, Zhu A, Zhang G (2015). Synlett.

[55] Fricke C, Schoenebeck F (2020). Acc Chem Res.

[56] Rogova T, Ahrweiler E, Schoetz M D, Schoenebeck F (2024). Angew Chem, Int Ed.

[57] Fricke C, Sherborne G J, Funes‐Ardoiz I, Senol E, Guven S, Schoenebeck F (2019). Angew Chem, Int Ed.

[58] Dahiya A, Schoetz M D, Schoenebeck F (2023). Angew Chem, Int Ed.

[59] Dahiya A, Gevondian A G, Schoenebeck F (2023). J Am Chem Soc.

[60] Dahiya A, Fricke C, Schoenebeck F (2020). J Am Chem Soc.

[61] Sherborne G J, Gevondian A G, Funes‐Ardoiz I, Dahiya A, Fricke C, Schoenebeck F (2020). Angew Chem, Int Ed.

[62] Fricke C, Deckers K, Schoenebeck F (2020). Angew Chem, Int Ed.

[63] Kaithal A, Sasmal H S, Dutta S, Schäfer F, Schlichter L, Glorius F (2023). J Am Chem Soc.

[64] Luo Y, Tian T, Nishihara Y, Lv L, Li Z (2021). Chem Commun.

[65] Xu Q-H, Xiao B (2022). Org Chem Front.

[66] Li W-F, Xu Q-H, Miao Q-Y, Xiao B (2024). J Org Chem.

[67] Han A-C, Xiao L-J, Zhou Q-L (2024). J Am Chem Soc.

[68] Piterskaya Y L, Khramchikhin A V, Stadnichuk M D (1996). Zh Obshch Khim.

[69] Demina M M, Nguyen T L H, Shaglaeva N S, Mareev A V, Medvedeva A S (2012). Russ J Org Chem.

[70] Zaitsev K V, Veshchitsky G A, Oprunenko Y F, Kharcheva A V, Moiseeva A A, Gloriozov I P, Lermontova E K (2023). Chem – Asian J.

[71] Smith E, Jones K D, O’Brien L, Argent S P, Salome C, Lefebvre Q, Valery A, Böcü M, Newton G N, Lam H W (2023). J Am Chem Soc.

[72] Wiesenfeldt M P, Rossi-Ashton J A, Perry I B, Diesel J, Garry O L, Bartels F, Coote S C, Ma X, Yeung C S, Bennett D J (2023). Nature.

[73] Pérez‐Perarnau A, Preciado S, Palmeri C M, Moncunill‐Massaguer C, Iglesias‐Serret D, González‐Gironès D M, Miguel M, Karasawa S, Sakamoto S, Cosialls A M (2014). Angew Chem, Int Ed.

[74] Wang C, Tobrman T, Xu Z, Negishi E-i (2009). Org Lett.

[75] Vantourout J C, Miras H N, Isidro-Llobet A, Sproules S, Watson A J B (2017). J Am Chem Soc.

[76] Peschke F, Taladriz-Sender A, Andrews M J, Watson A J B, Burley G A (2023). Angew Chem, Int Ed.

