# Supplementary material for: Germanyl triazoles as a platform for CuAAC diversification and chemoselective orthogonal cross-coupling
Source: Beilstein J Org Chem. 2024 Dec 5;20:3198–204. doi: 10.3762/bjoc.20.265 (PMC11635283; doi:10.3762/bjoc.20.265)
Supplement: File 1 — Characterization data and copies of NMR spectra. [file Beilstein_J_Org_Chem-20-3198-s001.pdf]

BEILSTEIN JOURNAL OF ORGANIC CHEMISTRY

Supporting Information

for

Germanyl triazoles as a platform for CuAAC diversification and chemoselective orthogonal cross-coupling

John M. Halford-McGuff, Thomas M. Richardson, Aidan P. McKay, Frederik Peschke, Glenn A. Burley and Allan J. B. Watson

Beilstein J. Org. Chem. **2024,** *20,* 3198–3204. doi:10.3762/bjoc.20.265

Characterization data and copies of NMR spectra

License and Terms: This is a supporting information file under the terms of the Creative Commons Attribution License (https://creativecommons.org/licenses/by/4.0). Please note that the reuse, redistribution and reproduction in particular requires that the author(s) and source are credited and that individual graphics may be subject to special legal provisions.
The license is subject to the *Beilstein Journal of Organic Chemistry* terms and conditions: (https://www.beilstein-journals.org/bjoc/terms)

Table of contents

1	General experimental information	S2
2	Optimisation	S3
3	Starting material synthesis	S5
4	Product synthesis and characterisation data	S13
5	X-ray diffraction data	S28
6	References	S29
7	Spectra	S30

1. General experimental information

Reagents and solvents were obtained from commercial suppliers and were not purified further unless specified. Purification (where specified) was performed following the standard procedures.[1] Unless otherwise specified, reactions were carried out using dry solvents which were obtained from a PureSolv SPS-400-5 solvent purification system.

Reactions were carried out in standard borosilicate glassware or 2 mL HPLC vials with septum caps. Glassware was either flame-dried under vacuum or allowed to dry in a 180 °C oven for 24 h before use and then sparged with nitrogen. Room temperature (rt) was approximately 18 °C. Reactions at high temperature were heated using a DrySyn metal heating bath or a silicone oil bath. Reactions at 0 °C were performed using an ice/water bath, −5 °C reaction temperatures were achieved with an ice/brine mixture, and −78 °C used dry ice/acetone baths.

TLC was carried out using Merck aluminum-backed silica plates coated with F_{254} fluorescent indicator, analysed under UV light, and developed using aqueous $KMnO_4$ or ethanolic vanillin solutions, where appropriate. Flash column chromatography was performed using silica gel (40–62 µm, Fluorochem).

^{1}H, ^{13}C (DEPTQ), and ^{19}F NMR (with or without ^{1}H decoupling) spectra were recorded by either a Bruker AVII 400 (BBFO probe) or AVIII-HD 500 (and AVIII 500 with BBFO+ and Prodigy BBFO probes, respectively) at 400-101-376 MHz or at 500, 126, 377 MHz, respectively. ^{1}H NMR spectra at 700 MHz, ^{13}C at 176 MHz, and ^{19}F NMR at 659 MHz (without ^{1}H decoupling) were recorded on a Bruker AVIII-HD 700 with Prodigy TCI probe. ^{11}B NMR spectra were recorded on a Bruker AV400 at 126 MHz. All spectra were recorded at rt with the deuterated solvents used as a lock for spectra and internal reference (*d*-chloroform: ^{1}H, 7.26 ppm; ^{13}C, 77.16 ppm; d_6-acetone: ^{1}H, 2.05 ppm, ^{13}C, 29.8 ppm; d_6-dimethylsulfoxide: ^{1}H 2.50 ppm, ^{13}C 39.5 ppm; d_3-acetonitrile: ^{1}H 1.94 ppm, ^{13}C 1.3 ppm). For ^{11}B NMR, samples were externally referenced to $F_3B{\cdot}OEt_2$ in $CDCl_3$. NMR spectra are reported as follows: chemical shift/ppm (multiplicity, coupling constant(s), number of nuclei). Multiplicity given as br (broad), s (singlet), d (doublet), t (triplet), q (quartet), quint (quintet), h (hextet), m (multiplet), and combinations thereof. Throughout, ^{13}C signals adjacent to boron were not observed. Signals which overlap with one another are described as multiplets. IR spectra were recorded using a Shimadzu IT Affinity-1 Fourier transform IR spectrophotometer with a Specac Quest ATR (diamond puck). The spectra were recorded as specified in the procedure as films (using CH_2Cl_2), as solids, or as neat liquids. Transmittance was recorded with maximal absorption wavenumbers given as cm^{-1}. Mass spectra were recorded on a Bruker micrOTOF benchtop ESI with either positive or negative electrospray ionization or EI using a Thermo Mat 900XP, double focusing high-resolution mass spectrometer. Note: a number of germanium alkynes are not detectable by HRMS due to excess fragmentation, although their click products, where appropriate, were characterised by HRMS.[2,3]

2. Optimisation

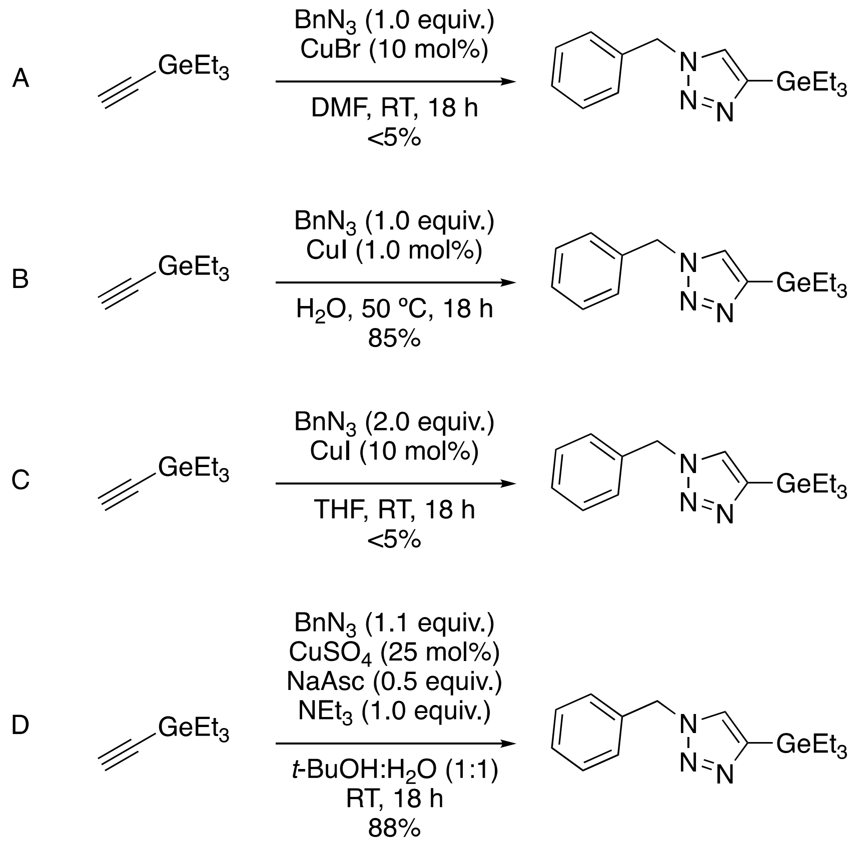

Scheme S1: Standard CuAAC procedures probed.

Scheme S1A procedure:

An HPLC vial was charged with CuBr (0.8 mg, 5.00 µmol, 10 mol %) and the vial was then sealed, evacuated, and backfilled with N_2 (3 ×). DMF (500 µL) was added followed by benzyl azide (6.3 µL, 50.0 µmol, 1.0 equiv), then triethyl(ethynyl)germane (9.0 µL, 50.0 µmol, 1.0 equiv) and the solution was stirred for 18 h at rt. After this, the reaction was quenched with 10% aq. NH_3 solution (5 mL) and extracted with CH_2Cl_2 (3 × 5 mL). The combined organic extracts were dried over Na_2SO_4, filtered, and concentrated in vacuo. NMR yield was then determined from the crude reaction mixture through comparison to a trichloroethylene (TCE) (9.0 µL, 100 µmol, 2.0 equiv) internal standard.

Scheme S1B procedure:

An HPLC vial was charged with CuI (0.2 mg, 1.0 µmol, 1.0 mol %) and the vial was then sealed, evacuated, and backfilled with N_2 (3 ×). H_2O (500 µL) was added followed by benzyl azide (12.5 µL, 100 µmol, 1.0 equiv), then triethyl(ethynyl)germane (18.0 µL, 100 µmol, 1.0 equiv), and the solution was heated to 50 °C and stirred for 18 h. After this, the reaction was quenched with 10% aq. NH_3 solution (5 mL) and extracted with CH_2Cl_2 (3 × 5 mL). The combined organic extracts were dried over Na_2SO_4, filtered, and concentrated in vacuo. The NMR yield was then determined from the crude reaction mixture through comparison to a trichloroethylene (TCE) (9.0 µL, 100 µmol, 2.0 equiv) internal standard.

Scheme S1C procedure:

An HPLC vial was charged with CuI (1.0 mg, 5.00 µmol, 10 mol %) and the vial was then sealed, evacuated, and backfilled with N_2 (3 ×). THF (500 µL) was added followed by benzyl azide (6.3 µL, 100 µmol, 2.0 equiv), then triethyl(ethynyl)germane (9.0 µL, 50.0 µmol, 1.0 equiv) and the solution was stirred for 18 h at rt. After this, the reaction was quenched with 10% aq. NH_3 solution (5 mL) and extracted with CH_2Cl_2 (3 × 5 mL). The combined organic extracts were dried over Na_2SO_4, filtered, and concentrated in vacuo. The NMR yield was then determined from the crude reaction mixture through comparison to a trichloroethylene (TCE) (9.0 µL, 100 µmol, 2.0 equiv) internal standard.

Scheme S1D procedure:

An HPLC vial was charged with sodium L-ascorbate (5.0 mg, 25.0 µmol, 0.50 equiv) and $CuSO_4$ (3.1 mg, 12.5 µmol, 25 mol %). The vial was then sealed, evacuated, and backfilled with N_2 (3 ×), before adding a 1:1 mixture of *t*-BuOH/H_2O (200 µL, 0.25 M). Triethylamine (7.0 µL, 50.0 µmol, 1.0 equiv) was added followed by benzyl azide (6.9 µL, 55.0 µmol, 1.1 equiv), and triethyl(ethynyl)germane (9.0 µL, 50.0 µmol, 1.0 equiv) and the solution was stirred for 18 h at rt. After this, the reaction was quenched with 10% aq. NH_3 solution (5.0 mL) and extracted with CH_2Cl_2 (3 × 5 mL). The combined organic extracts were dried over Na_2SO_4, filtered, and concentrated in vacuo. The NMR yield was then determined from the crude reaction mixture through comparison to a trichloroethylene (TCE) (9.0 µL, 100 µmol, 2.0 equiv) internal standard.

Table S1: Ge CuAAC optimisation.

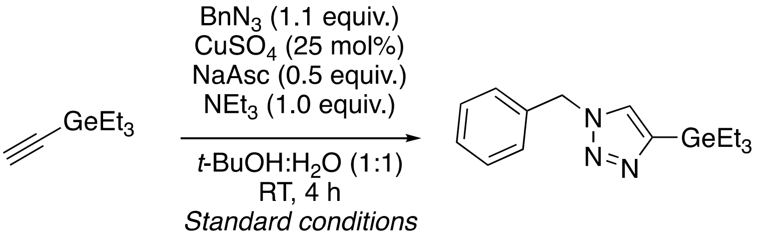

Entry	Changes from standard conditions	Yield (%)[a]
**1**	-	36
**2**	18 h	88
**3**	No NaAsc	24
**4**	No base	41
**5**	No *t*-BuOH	50
**6**	No H_2O	58
**7**	$CuSO_4$•$5H_2O$ (5.0 mol%), 16 h	>99

[a]Determined by ^{1}H NMR using trichloroethylene (TCE) as internal standard. 50.0 µmol scale.

Table S1 procedure:

An HPLC vial was charged with sodium L-ascorbate (5.0 mg, 25.0 µmol, 0.50 equiv) and either $CuSO_4$ (3.1 mg, 12.5 µmol, 25 mol %) or $CuSO_4\cdot 5H_2O$ (0.6 mg, 2.5 µmol, 5.0 mol %). The vial was then sealed, evacuated, and backfilled with N_2 (3 ×), before adding a 1:1 mixture of *t*-BuOH/H_2O (200 µL, 0.25 M). Triethylamine (7.0 µL, 50.0 µmol, 1.0 equiv) was added followed by benzyl azide (6.9 µL, 55.0 µmol, 1.1 equiv), and triethyl(ethynyl)germane (9.0 µL, 50.0 µmol, 1.0 equiv) and the solution was stirred for four hours at rt. After this, the reaction was quenched with 10% aq. NH_3 solution (5 mL) and extracted with CH_2Cl_2 (3 × 5 mL). The combined organic extracts were dried over Na_2SO_4, filtered, and concentrated in vacuo. The NMR yield was then determined from the crude reaction mixture through comparison to a trichloroethylene (TCE) (9.0 µL, 50.0 µmol, 2.0 equiv) internal standard.

3. Starting material synthesis

General Procedure A

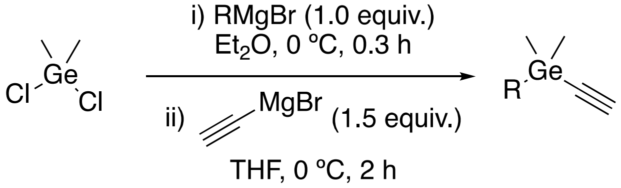

A flame-dried flask was charged with dichlorodimethylgermane (116 µL, 1.00 mmol, 1.0 equiv) and Et_2O (20 mL, 0.05 M) under N_2 and the resulting mixture was cooled to 0 °C. A solution of the chosen Grignard reagent (1.0 equiv) was added dropwise, and the mixture was stirred for 20 min. A solution of ethynylmagnesium bromide in THF (3.00 mL, 0.5 M, 1.50 mmol, 1.5 equiv) was added dropwise at 0 °C. The mixture was stirred for two hours while allowing it to warm to rt. The mixture was quenched by the addition of H_2O (5 mL) and the biphasic system was extracted with Et_2O (3 × 10 mL). The combined organic extracts were dried over Na_2SO_4, filtered, and concentrated in vacuo to give the crude product, which was purified by flash column chromatography. Respective purification methods are disclosed below.

General Procedure B

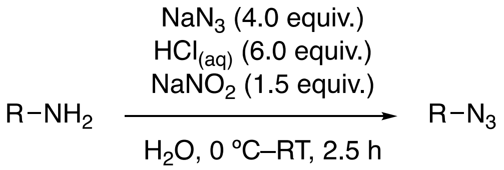

A round-bottomed flask was charged with the chosen amine (1.0 equiv) and aq. 6 M HCl (6.0 equiv) and the mixture was cooled to 0 °C. A solution of sodium nitrite (1.5 equiv) in distilled H_2O (≈ 1.0 M) was added and the resulting solution was stirred at 0 °C for 30 min. A solution of sodium azide (4.0 equiv.) in distilled H_2O (≈ 3.0 M) was added dropwise at 0 °C. The mixture was stirred for two hours while allowing it to warm to rt. The mixture was quenched with sat. aq. $NaHCO_3$ (5 mL per mmol amine) and extracted with Et_2O (3 × 10 mL per mmol amine). The combined organic extracts were washed with brine, dried over Na_2SO_4, filtered, and concentrated in vacuo to give the desired product as a brown liquid, which was used without further purification.

General Procedure C

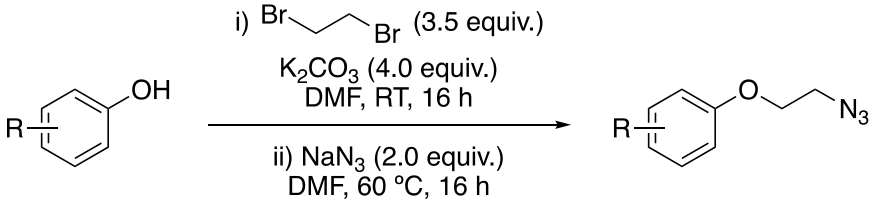

An oven-dried flask was charged with the chosen phenol (1.0 equiv), 1,2-dibromoethane (3.5 equiv), and DMF (0.4 M). K_2CO_3 (4.0 equiv) was added, and the mixture was stirred at rt for 16 h. H_2O (5 mL per mmol of phenol) was added, and the mixture was extracted with Et_2O (5 mL per mmol of phenol). The organic extract was washed with H_2O (2 × 5 mL per mmol of phenol), then brine (3 × 5 mL per mmol of phenol). The combined organic extracts were dried over $MgSO_4$, filtered, and concentrated in vacuo to yield a residue that was used in the next step without further purification.

A flask was charged with the prepared alkyl bromide (1.0 equiv) and DMF (0.3 M). NaN_3 (2.0 equiv) was added, and the mixture was heated to 60 °C and stirred for 16 h. The mixture was allowed to cool to rt and H_2O (5 mL per mmol of alkyl bromide) was added. The mixture was extracted with EtOAc (5 mL per mmol of alkyl bromide). The organic extract was washed with H_2O (2 × 5 mL per mmol of phenol), then brine (3 × 5 mL per mmol of phenol). The organic extract was dried over $MgSO_4$, filtered, and concentrated in vacuo to give the crude product, which was purified by flash column chromatography. Respective purification methods are disclosed below.

General Procedure D

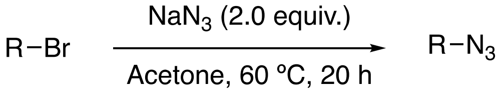

An oven-dried microwave vial was charged with the chosen alkyl bromide (1.0 equiv) and acetone (1.0 M). NaN_3 (2.0 equiv) was added, and the mixture was heated to 60 °C and stirred for 20 h. The mixture was allowed to cool to rt and H_2O (10 mL per mmol of reactant) was added. The mixture was extracted with Et_2O (2 × 20 mL per mmol of reactant), and the combined organic extracts were washed with brine (3 × 10 mL per mmol of reactant), dried over Na_2SO_4, filtered, and concentrated in vacuo to give the crude product, which was purified by flash column chromatography. Respective purification methods are disclosed below.

Benzyl(ethynyl)dimethylgermane (S1)

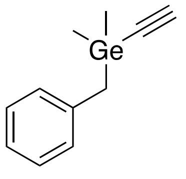

Prepared according to **General Procedure A** using an Et_2O solution of benzylmagnesium chloride (810 µL, 1.23 M, 1.00 mmol, 1.0 equiv) to afford the crude product. This was purified by flash column chromatography (silica, hexane) to give the desired product as a colourless oil (71.9 mg, 329 µmol, 33%).

^{1}H NMR (500 MHz, $CDCl_3$) δ 7.24 (t, J = 7.6 Hz, 2H), 7.13 – 7.07 (m, 3H), 2.41 (s, 2H), 2.32 (s, 1H), 0.31 (s, 6H).

^{13}C NMR (126 MHz, $CDCl_3$) δ 139.3, 128.5, 128.1, 124.8, 93.0, 88.9, 26.0, –2.3.

IR (ATR, film) ν_{max} 3285, 2913, 2029, 1600, 1493, 1452, 1209, 810, 752 cm^{-1}.

HRMS inconclusive due to excessive fragmentation.

Ethynyldimethyl(*o*-tolyl)germane (S2)

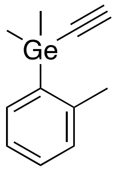

Prepared according to **General Procedure A** using an Et_2O solution of *o*-tolylmagnesium chloride (1.00 mL, 1.0 M, 1.00 mmol, 1.0 equiv) to afford the crude product. This was purified by flash column chromatography (silica, 10% Et_2O in hexane) to give the desired product as a colourless oil (86.7.mg, 396 µmol, 40%).

^{1}H NMR (500 MHz, $CDCl_3$) δ 7.60 (dd, J = 7.1, 1.8 Hz, 1H), 7.31 – 7.27 (m, 1H), 7.23 – 7.17 (m, 2H), 2.49 (s, 3H), 2.43 (s, 1H), 0.66 (s, 6H).

^{13}C NMR (126 MHz, $CDCl_3$) δ 142.9, 136.5, 134.0, 130.0, 129.7, 125.5, 93.6, 88.9, 22.9, −0.1.

IR (ATR, film) ν_{max} 3273, 2914, 2029, 1558, 1447, 1240, 1125, 810 cm^{-1}.

HRMS inconclusive due to excessive fragmentation.

4-Azido-*N*-(pyrimidin-2-yl)benzenesulfonamide (S3)

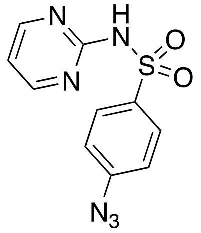

Prepared according to **General Procedure B** using 4-amino-*N*-(pyrimidin-2-yl)benzenesulfonamide (2.50 g, 10.0 mmol, 1.0 equiv) to give the desired product as a white solid, which was used without further purification (2.50 g, 9.00 mmol, 90%).

^{1}H NMR (500 MHz, DMSO-*d*$_6$) δ 11.76 (br. s, 1H), 8.49 (d, *J* = 4.9 Hz, 2H), 7.98 (d, *J* = 8.7 Hz, 2H), 7.28 (d, *J* = 8.7 Hz, 2H), 7.04 (t, *J* = 4.9 Hz, 1H).

^{13}C NMR (126 MHz, DMSO-*d*$_6$) δ 158.4, 157.0, 144.1, 136.6, 129.6, 119.4, 115.7.

Spectral data in agreement with the literature.[4]

10-(4-(2-Azidoethoxy)phenyl)-10*H*-phenothiazine (S4)

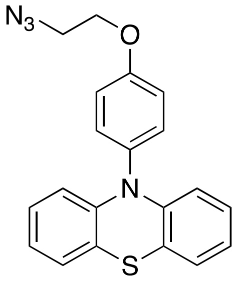

Prepared according to **General Procedure C** using 4-(10*H*-phenothiazin-10-yl)phenol (291 mg, 1.00 mmol, 1.0 equiv) to afford the crude product. This was purified by flash column chromatography (silica, 5–20% Et_2O in hexane) to give the desired product as a white solid (159 mg, 440 µmol, 44%).

^{1}H NMR (500 MHz, $CDCl_3$) δ 7.36 – 7.29 (m, 2H), 7.17 – 7.10 (m, 2H), 6.99 (dd, *J* = 7.4, 1.7 Hz, 2H), 6.86 – 6.81 (m, 2H), 6.81 – 6.77 (m, 2H), 6.18 (dd, *J* = 8.1, 1.4 Hz, 2H), 4.24 (t, *J* = 4.9 Hz, 2H), 3.67 (t, *J* = 4.9 Hz, 2H).

^{13}C NMR (126 MHz, $CDCl_3$) δ 158.0, 144.7, 134.1, 132.6, 127.7, 126.8, 122.5, 119.8, 116.6, 115.8, 67.4, 50.3.

IR (ATR, solid) ν_{max} 2089, 1506, 1456, 1298, 1229, 1057, 1042, 908 cm^{-1}.

HRMS (ESI) Calculated for 361.1118 *m*/*z*, found 361.1113 *m*/*z* $[C_{20}H_{16}N_4OS+H]^+$.

4-(2-Azidoethoxy)-2*H*-chromen-2-one (S5)

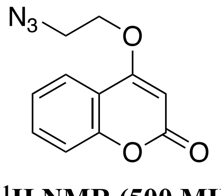

Prepared according to **General Procedure C** using 4-hydroxy-2*H*-chromen-2-one (973 mg, 6.00 mmol, 1.0 equiv) to afford the crude product. This was purified by flash column chromatography (silica, 5–20% Et_2O in hexane) to give the desired product as a white solid (560 mg, 2.40 mmol, 40%).

^{1}H NMR (500 MHz, $CDCl_3$) δ 7.84 (dd, *J* = 7.9, 1.6 Hz, 1H), 7.57 (ddd, *J* = 8.7, 7.3, 1.6 Hz, 1H), 7.34 (dd, *J* = 8.5, 1.1 Hz, 1H), 7.30 (ddd, *J* = 8.2, 7.3, 1.1 Hz, 1H), 5.69 (s, 1H), 4.31 (t, *J* = 4.8 Hz, 2H), 3.75 (t, *J* = 4.8 Hz, 2H).

^{13}C NMR (126 MHz, $CDCl_3$) δ 165.3, 162.7, 153.5, 132.9, 124.3, 123.2, 117.0, 115.4, 91.1, 68.4, 49.8

Spectral data in agreement with the literature.[5]

2-(Azidomethyl)pyridine (S6)

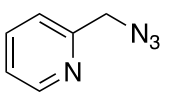

A flask was charged with NaN_3 (1.18 g, 18.2 mmol, 1.8 equiv) and DMF (10 mL). 2-(Bromomethyl)pyridine hydrobromide (2.53 g, 10.0 mmol, 1.0 equiv) was added followed by K_2CO_3 (2.51 g, 18.2 mmol, 1.8 equiv) and the mixture was stirred at rt for 16 h. The mixture was diluted with H_2O (20 mL) and extracted with Et_2O (3 × 10 mL). The combined organic extracts were dried over $MgSO_4$, filtered, and concentrated in vacuo to give the desired product as a brown oil, which was used without further purification (1.15 g, 8.58 mmol, 86%).

^{1}H NMR (700 MHz, $CDCl_3$) δ 8.61 – 8.55 (m, 1H), 7.75 – 7.65 (m, 1H), 7.33 (d, J = 7.8 Hz, 1H), 7.23 (dd, J = 7.5, 4.9 Hz, 1H), 4.47 (s, 2H).

^{13}C NMR (176 MHz, $CDCl_3$) δ 155.8, 149.8, 137.1, 123.0, 122.1, 55.8.

Spectral data in agreement with the literature.[6]

1-(Azidomethyl)-4-fluorobenzene (S7)

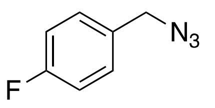

A flask was charged with NaN_3 (1.30 g, 20.0 mmol, 2.0 equiv) and DMF (10 mL). 4-Fluorobenzyl bromide (1.25 mL, 10.0 mmol, 1.0 equiv) was added and the mixture was heated to 60 °C and stirred for 16 h. The mixture was allowed to cool to rt, diluted with H_2O (20 mL) and extracted with Et_2O (3 × 10 mL). The combined organic extracts were washed with brine (5 × 10 mL), dried over $MgSO_4$, filtered, and concentrated in vacuo to give the desired product as a colourless oil, which was used without further purification (1.50 g, 9.92 mmol, 99%).

^{1}H NMR (700 MHz, $CDCl_3$) δ 7.32 – 7.28 (m, 2H), 7.11 – 7.05 (m, 2H), 4.32 (s, 2H).

^{13}C NMR (176 MHz, $CDCl_3$) δ 162.8 (d, $^1J_{CF}$ = 247.2 Hz), 131.3 (d, $^3J_{CF}$ = 3.1 Hz), 130.2 (d, $^2J_{CF}$ = 8.3 Hz), 115.9, 54.2.

^{19}F NMR (659 MHz, $CDCl_3$) δ –113.57 (tt, J = 8.8, 4.8 Hz).

Spectral data in agreement with the literature.[7]

1-Azido-4-fluorobenzene (S8)

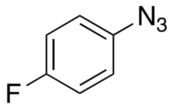

Prepared according to **General Procedure B** using 4-fluoroaniline (1.11 g, 10.0 mmol, 1.0 equiv) to give the desired product as a brown oil, which was used without further purification (1.37 g, 10.0 mmol, >99%).

^{1}H NMR (500 MHz, $CDCl_3$) δ 7.08 – 7.02 (m, 2H), 7.02 – 6.96 (m, 2H).

^{13}C NMR (176 MHz, $CDCl_3$) δ 160.1 (d, $^1J_{CF}$ = 244.3 Hz), 136.0 (d, $^4J_{CF}$ = 2.3 Hz), 120.5 (d, $^3J_{CF}$ = 8.2 Hz), 116.8 (d, $^2J_{CF}$ = 23.1 Hz).

^{19}F NMR (659 MHz, $CDCl_3$) δ –117.73 (tt, J = 8.5, 4.3 Hz).

Spectral data in agreement with the literature.[8]

1-Azido-4-methylbenzene (S9)

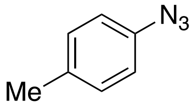

Prepared according to **General Procedure B** using *p*-toluidine (1.07 g, 10.0 mmol, 1.0 equiv) to give the desired product as a brown oil, which was used without further purification (1.33 g, 10.0 mmol, >99%).

^{1}H NMR (700 MHz, $CDCl_3$) δ 7.18 – 7.14 (m, 2H), 6.95 – 6.90 (m, 2H), 2.34 (d, J = 3.2 Hz, 3H).

^{13}C NMR (176 MHz, $CDCl_3$) δ 137.3, 134.7, 130.5, 119.0, 21.0.

Spectral data in agreement with the literature.[8]

2-Azido-1-phenylethan-1-one (S10)

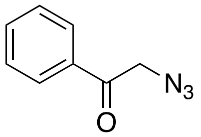

A flask was charged with 2-bromoacetophenone (1.59 g, 8.00 mmol, 1.0 equiv) and DMF (8.0 mL). NaN_3 (1.04 g, 16.0 mmol, 2.0 equiv) was added and the mixture was stirred at rt for three hours. H_2O (20 mL) was added, and the mixture was extracted with Et_2O (3 × 10 mL). The combined organic extracts were washed with brine (5 × 10 mL), dried over $MgSO_4$, filtered, and concentrated in vacuo to give the desired product as a brown solid, which was used without further purification (1.29 g, 8.00 mmol, >99%).

^{1}H NMR (500 MHz, $CDCl_3$) δ 7.91 (dd, J = 8.3, 1.4 Hz, 2H), 7.66 – 7.59 (m, 1H), 7.53 – 7.47 (m, 2H), 4.56 (s, 2H).

^{13}C NMR (126 MHz, $CDCl_3$) δ 193.3, 134.5, 134.3, 129.1, 128.1, 55.0.

Spectral data in agreement with the literature.[9]

1-(Azidomethyl)-2-nitrobenzene (S11)

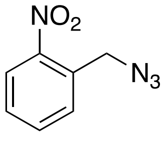

Prepared according to **General Procedure D** from 1-(bromomethyl)-2-nitrobenzene (648 mg, 3.00 mmol, 1.0 equiv) to afford the crude product. This was purified by flash column chromatography (silica, 0–10% Et_2O in hexane) to give the desired product as a yellow oil (475 mg, 2.67 mmol, 89%).

^{1}H NMR (400 MHz, $CDCl_3$) δ 8.17 – 8.04 (m, 1H), 7.74 – 7.62 (m, 2H), 7.55 – 7.49 (m, 1H), 4.85 (s, 2H).

^{13}C NMR (101 MHz, $CDCl_3$) δ 147.8, 134.1, 131.7, 130.3, 129.2, 125.4, 52.1.

Spectral data in agreement with the literature.[10]

1-(Azidomethyl)-2-iodobenzene (S12)

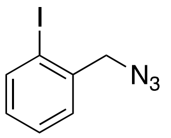

Prepared according to **General Procedure D** from 1-(bromomethyl)-2-iodobenzene (891 mg, 3.00 mmol, 1.0 equiv) to afford the crude product. This was purified by flash column chromatography (silica, 0–5% Et_2O in hexane) to give the desired product as a colourless oil (598 mg, 2.31 mmol, 77%).

^{1}H NMR (400 MHz, $CDCl_3$) δ 7.88 (dd, *J* = 8.0, 0.9 Hz, 1H), 7.44 – 7.34 (m, 2H), 7.10 – 6.98 (m, 1H), 4.46 (s, 2H).

^{13}C NMR (101 MHz, $CDCl_3$) δ 139.9, 138.3, 130.1, 129.6, 128.8, 99.1, 59.2.

Spectral data in agreement with the literature.[11]

2-(4-(Azidomethyl)phenyl)-4,4,5,5-tetramethyl-1,3,2-dioxaborolane (S13)

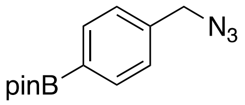

Prepared according to **General Procedure D** from 2-(4-(bromomethyl)phenyl)-4,4,5,5-tetramethyl-1,3,2-dioxaborolane (743 mg, 2.50 mmol, 1.0 equiv) to afford the crude product. This was purified by flash column chromatography (silica, 0–5% Et_2O in hexane) to give the desired product as a colourless oil (84.2 mg, 325 µmol, 13%).

^{1}H NMR (400 MHz, $CDCl_3$) δ 7.92 – 7.76 (m, 2H), 7.34 – 7.30 (m, 2H), 4.35 (s, 2H), 1.35 (s, 12H).

^{13}C NMR (101 MHz, $CDCl_3$) δ 138.4, 135.4, 127.6, 84.1, 54.9, 33.5, 25.0.

^{11}B NMR (128 MHz, $CDCl_3$) δ 31.0.

Spectral data in agreement with the literature.[12]

(*E*)-(2-Azidovinyl)benzene (S14)

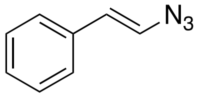

An oven-dried microwave vial was charged with styreneboronic acid (148 mg, 1.00 mmol, 1.0 equiv), MeOH (3.0 mL), and anhydrous $CuSO_4$ (16.0 mg, 100 µmol, 10 mol %). NaN_3 (78.0 mg, 1.20 mmol, 1.2 equiv) was added and the mixture was stirred at rt open to air for 16 h. The mixture was concentrated in vacuo to afford the crude product. This was purified by flash column chromatography (silica, 0–10% Et_2O in hexane) to give the desired product as a yellow oil (37.7 mg, 260 µmol, 26%).

^{1}H NMR (400 MHz, $CDCl_3$) δ 7.35 – 7.26 (m, 4H), 7.25 – 7.20 (m, 1H), 6.61 (d, *J* = 13.8 Hz, 1H), 6.28 (d, *J* = 13.8 Hz, 1H).

^{13}C NMR (101 MHz, $CDCl_3$) δ 135.2, 128.9, 127.5, 126.8, 126.0, 119.9.

Spectral data in agreement with the literature.[13]

1-(2-Azidoethyl) 3-methyl bicyclo[1.1.1]pentane-1,3-dicarboxylate (S15)

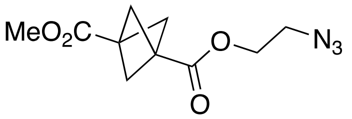

A flame-dried flask was charged with 3-(methoxycarbonyl)bicyclo[1.1.1]pentane-1-carboxylic acid (170 mg, 1.00 mmol, 1.0 equiv), DMAP (12.2 mg, 100 µmol, 10 mol %), DCC (268 mg, 1.30 mmol, 1.3 equiv), and CH_2Cl_2 (5.0 mL). 2-Azidoethan-1-ol (131 mg, 1.50 mmol, 1.5 equiv) was added and the mixture was stirred for 16 h at rt. The reaction mixture was filtered and concentrated in vacuo to afford the crude product. This was purified by flash column chromatography (silica, 0–20% Et_2O in hexane) to give the desired product as a white solid (203 mg, 849 µmol, 85%).

^{1}H NMR (700 MHz, CDCl$_3$) δ 4.30 – 4.26 (m, 2H), 3.69 (s, 3H), 3.48 – 3.42 (m, 2H), 2.35 (s, 6H).

^{13}C NMR (176 MHz, CDCl$_3$) δ 169.7, 169.0, 63.9, 53.0, 52.0, 49.7, 37.8, 37.7.

IR (ATR, film) ν_{max} 2926, 2102, 1729, 1439, 1285, 1209, 1194, 1159, 1144, 1051 cm^{-1}.

HRMS (ESI) Calculated for 240.0979 *m/z*, found 240.0975 *m/z* $[C_{10}H_{13}N_3O_4+H]^+$.

4-(Methoxycarbonyl)cubane-1-carboxylic acid (S16)

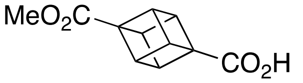

A flask was charged with dimethyl cubane-1,4-dicarboxylate (231 mg, 1.05 mmol, 1.1 equiv) and THF (7.0 mL). NaOH (40.0 mg, 1.00 mmol, 1.0 equiv) in MeOH (7.0 mL) was added, and the mixture was stirred for 16 h at rt, then concentrated in vacuo. The residue was suspended in CH_2Cl_2 (10 mL) and filtered. The filtered solid was dissolved in H_2O (5.0 mL) and acidified with dilute aq. HCl until a pH 1 was reached. The aqueous mixture was then extracted with CH_2Cl_2 (3 × 10 mL) and the combined organic extracts were dried over Na_2SO_4, filtered, and concentrated in vacuo to give the desired product as a white solid, which was used without further purification (110 mg, 533 µmol, 53%).

^{1}H NMR (500 MHz, CDCl$_3$) δ 4.30 – 4.24 (m, 6H), 3.71 (s, 3H).

^{13}C NMR (126 MHz, CDCl$_3$) δ 177.3, 172.0, 56.0, 55.6, 51.8, 47.3, 47.2.

Spectral data in agreement with the literature.[14]

1-(2-Azidoethyl) 4-methyl cubane-1,4-dicarboxylate (S17)

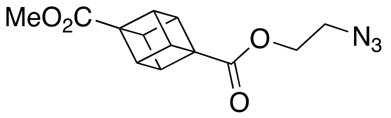

A flame-dried flask was charged with 4-(methoxycarbonyl)cubane-1-carboxylic acid (103 mg, 500 µmol, 1.0 equiv), DMAP (8.6 mg, 70.0 µmol, 14 mol %), EDCI (144 mg, 750 µmol, 1.5 equiv), and CH_2Cl_2 (2.5 mL). 2-Azidoethan-1-ol (47.9 mg, 550 µmol, 1.1 equiv) was added and the mixture was stirred for 16 h at rt. H_2O (10 mL) was added, the organic phase was collected, and the aqueous phase was extracted with CH_2Cl_2 (3 × 10 mL). The combined organic extracts were dried over Na_2SO_4, filtered, and concentrated in vacuo to afford the crude product. This was purified by flash column chromatography (silica, 0–20% Et_2O in hexane) to give the desired product as a white solid (71.8 mg, 261 µmol, 52%).

^{1}H NMR (700 MHz, CDCl$_3$) δ 4.30 (dd, *J* = 5.6, 4.6 Hz, 2H), 4.28 – 4.21 (m, 6H), 3.70 (s, 3H), 3.49 – 3.44 (m, 2H).

^{13}C NMR (176 MHz, CDCl$_3$) δ 172.0, 171.3, 63.5, 56.0, 55.7, 51.8, 49.9, 47.3, 47.2.

IR (ATR, film) ν_{max} 2997, 2110, 2091, 1717, 1439, 1321, 1271, 1213, 1198, 1088, 1076,1034, 924, 841, 829, 785 cm^{-1}.

HRMS (ESI) Calculated for 276.0979 *m*/*z*, found 276.0973 *m*/*z* $[C_{13}H_{13}N_3O_4+H]^+$.

Triethyl(ethynyl)germane (S18)

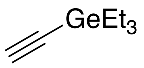

A flame-dried flask was charged with chlorotriethylgermane (1.00 g, 5.12 mmol, 1.0 equiv) and Et_2O (20 mL) under N_2. Ethynylmagnesium bromide in THF (9.50 mL, 0.5 M, 4.75 mmol, 1.5 equiv) was added dropwise and the mixture was stirred at rt for two hours. The mixture was quenched by the addition of H_2O (50 mL) and the organic phase was collected. The aqueous phase was extracted with Et_2O (3 × 50 mL), and the combined organic extracts were dried over Na_2SO_4, filtered, and concentrated in vacuo to give the desired product as a yellow oil, which was used without further purification (850 mg, 4.60 mmol, 90%).

^{1}H NMR (400 MHz, $CDCl_3$) δ 2.27 (s, 1H), 1.16 – 1.06 (m, 9H), 0.88 (m, 6H).

^{13}C NMR (101 MHz, $CDCl_3$) δ 94.9, 87.9, 9.0, 5.7.

Spectral data in agreement with the literature.[15]

Ethynyltriphenylgermane (S19)

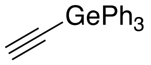

A flame-dried flask was charged with chlorotriphenylgermane (1.47 g, 4.33 mmol, 1.0 equiv) and Et_2O (20 mL) under N_2. Ethynylmagnesium bromide in THF (20.0 mL, 0.5 M, 10.0 mmol, 2.3 equiv) was added dropwise and the mixture was stirred at rt for two hours. The mixture was quenched by the addition of H_2O (50 mL) and the organic phase was collected. The aqueous phase was extracted with Et_2O (3 × 50 mL), and the combined organic extracts were dried over Na_2SO_4, filtered, and concentrated in vacuo to give the desired product as a white solid, which was used without further purification (1.42 g, 4.32 mmol, >99%).

^{1}H NMR (500 MHz, $CDCl_3$) δ 7.67 (d, *J* = 5.4 Hz, 6H), 7.51 – 7.40 (m, 9H), 2.68 (s, 1H).

^{13}C NMR (101 MHz, $CDCl_3$) δ 134.6, 133.9, 129.8, 128.6, 96.0, 84.9.

Spectral data in agreement with the literature.[16]

4. Product synthesis and characterisation data

General Procedure E

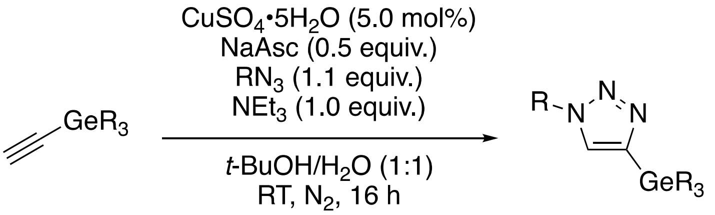

A HPLC vial was charged with sodium L-ascorbate (0.50 equiv), $CuSO_4 \cdot 5H_2O$ (5.0 mol %), ethynylgermane (1.0 equiv – **if solid**) and azide (1.1 equiv – **if solid**). The vial was then evacuated and backfilled with N_2 (3 ×), before adding a 1:1 mixture of *t*-BuOH/H_2O (0.25 M). To this was added triethylamine (1.0 equiv), followed by azide (1.1 equiv – **if liquid**), and ethynylgermane (1.0 equiv – **if liquid**), and the resulting mixture was stirred at rt under N_2 for 16 h. After this, the reaction was quenched with 10% aq. NH_3 solution (25 mL per mmol alkyne) and extracted with CH_2Cl_2 (3 × 25 mL per mmol alkyne), dried over Na_2SO_4, filtered, and concentrated in vacuo. NMR yield was then determined from the crude reaction mixture through comparison to a TCE (1.0 equiv) internal standard. Respective purification methods are disclosed below.

2-((4-(Triphenylgermyl)-1*H*-1,2,3-triazol-1-yl)methyl)pyridine (1)

Prepared according to **General Procedure E** using ethynyltriphenylgermane (65.8 mg, 200 μmol, 1.0 equiv) and 2-(azidomethyl)pyridine (29.5 mg, 220 μmol, 1.1 equiv) to afford the crude product. This was purified by flash column chromatography (silica, 10% EtOAc in hexane, followed by acetone flush) to give the desired product as a yellow solid (78.8 mg, 170 μmol, 85%).

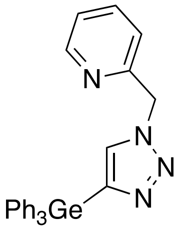

^{1}H NMR (500 MHz, $CDCl_3$) δ 8.56 (ddd, *J* = 4.9, 1.8, 0.9 Hz, 1H), 7.70 (s, 1H), 7.67 (td, *J* = 7.7, 1.8 Hz, 1H), 7.63 – 7.56 (m, 6H), 7.44 – 7.34 (m, 9H), 7.24 (ddd, *J* = 7.7, 4.9, 1.1 Hz, 1H), 7.17 (dt, *J* = 7.8, 1.1 Hz, 1H), 5.71 (s, 2H).

^{13}C NMR (126 MHz, $CDCl_3$) δ 143.4, 135.4, 135.3, 135.2, 135.1, 130.4, 129.5, 129.2, 128.7, 128.5, 128.0, 53.8.

IR (ATR, film) ν_{max} 3067, 3049, 1485, 1431, 1193, 1092, 1045, 997 cm^{-1}.

HRMS (ESI) Calculated for 465.1129 *m/z*, found 465.1139 *m/z* $[C_{26}H_{22}GeN_4+H]^+$.

1-(2-Nitrobenzyl)-4-(triphenylgermyl)-1*H*-1,2,3-triazole (2)

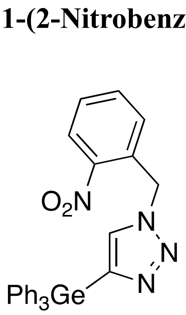

Prepared according to **General Procedure E** using ethynyltriphenylgermane (65.8 mg, 200 μmol, 1.0 equiv) and 1-(azidomethyl)-2-nitrobenzene (57.0 mg, 220 μmol, 1.1 equiv) to afford the crude product. This was purified by flash column chromatography (silica, 0–50% EtOAc in hexane) to give the desired product as a pale-yellow solid (56.6 mg, 112 μmol, 56%).

^{1}H NMR (700 MHz, $CDCl_3$) δ 8.13 (dd, *J* = 8.1, 1.3 Hz, 1H), 7.70 (s, 1H), 7.64 – 7.57 (m, 7H), 7.51 (td, *J* = 7.8, 1.4 Hz, 1H), 7.44 – 7.36 (m, 9H), 7.04 (dd, *J* = 7.8, 1.4 Hz, 1H), 5.99 (s, 2H).

^{13}C NMR (176 MHz, $CDCl_3$) δ 147.5, 143.8, 135.2, 135.2, 134.5, 131.6, 131.2, 130.3, 129.6, 129.6, 128.6, 125.5, 50.5.

IR (ATR, film) ν_{max} 1528, 1485, 1431, 1341, 1265, 1094, 1044 cm^{-1}.

HRMS (ESI) Calculated for 509.1027 *m*/*z*, found 509.1031 *m*/*z* $[C_{27}H_{22}GeN_4O_2+H]^+$.

1-(2-Iodobenzyl)-4-(triphenylgermyl)-1*H*-1,2,3-triazole (3)

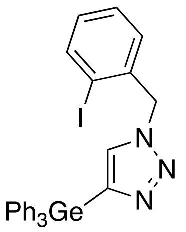

Prepared according to **General Procedure E** using ethynyltriphenylgermane (65.8 mg, 200 µmol, 1.0 equiv) and 1-(azidomethyl)-2-iodobenzene (57.0 mg, 220 µmol, 1.1 equiv) to afford the crude product. This was purified by flash column chromatography (silica, 0–30% EtOAc in hexane) to give the desired product as a white, waxy solid (82.8 mg, 141 µmol, 70%).

^{1}H NMR (500 MHz, CDCl$_3$) δ 7.87 (d, *J* = 7.9 Hz, 1H), 7.65 – 7.57 (m, 7H), 7.44 – 7.35 (m, 9H), 7.32 (t, *J* = 7.6 Hz, 1H), 7.08 – 6.98 (m, 2H), 5.66 (s, 2H).

^{13}C NMR (126 MHz, CDCl$_3$) δ 143.3, 139.9, 137.7, 135.4, 135.3, 134.3, 131.0, 130.4, 129.5, 129.2, 128.5, 98.4, 58.1.

IR (ATR, film) ν_{max} 3048, 1483, 1431, 1308, 1265, 1188, 1092, 1044, 1015, 997, 814 cm^{-1}.

HRMS (ESI) Calculated for 590.0143 *m*/*z*, found 590.0147 *m*/*z* $[C_{27}H_{22}GeN_3I+H]^+$.

1-(4-Fluorobenzyl)-4-(triphenylgermyl)-1*H*-1,2,3-triazole (4)

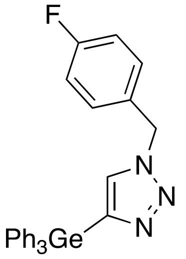

Prepared according to **General Procedure E** using ethynyltriphenylgermane (65.8 mg, 200 µmol, 1.0 equiv) and 4-fluorobenzyl azide (33.3 mg, 220 µmol, 1.1 equiv) to afford the crude product. This was purified by flash column chromatography (silica, 10% EtOAc in hexane, followed by acetone flush) to give the desired product as a white solid (52.3 mg, 109 µmol, 55%).

^{1}H{^{19}F} NMR (500 MHz, CDCl$_3$) δ 7.60 – 7.56 (m, 6H), 7.46 (s, 1H), 7.43 – 7.35 (m, 9H), 7.26 – 7.21 (m, 2H), 7.07 – 7.01 (m, 2H), 5.54 (s, 2H).

^{13}C NMR (126 MHz, CDCl$_3$) δ 162.9 (d, $^1J_{CF}$ = 248.0 Hz), 143.6, 135.3, 135.2, 130.9 (d, $^4J_{CF}$ = 3.9 Hz), 130.2, 130.0 (d, $^3J_{CF}$ = 8.7 Hz), 129.5, 128.5, 116.2 (d, $^2J_{CF}$ = 22.1 Hz), 53.1.

^{19}F{^{1}H} NMR (470 MHz, CDCl$_3$) δ −112.96.

IR (ATR, film) ν_{max} 3069, 3048, 2320, 1653, 1508, 1431, 1225, 1094, 1045, 841, 772 cm^{-1}.

HRMS (ESI) Calculated for 482.1082 *m*/*z*, found 482.1081 *m*/*z* $[C_{27}H_{21}FGeN_3+H]^+$.

1-(4-(4,4,5,5-Tetramethyl-1,3,2-dioxaborolan-2-yl)benzyl)-4-(triphenylgermyl)-1*H*-1,2,3-triazole (5)

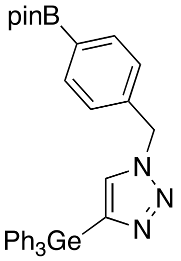

Prepared according to **General Procedure E** using ethynyltriphenylgermane (32.9 mg, 100 μmol, 1.0 equiv) and 2-(4-(azidomethyl)phenyl)-4,4,5,5-tetramethyl-1,3,2-dioxaborolane (28.5 mg, 110 μmol, 1.1 equiv) with the addition of CsF (30.4 mg, 200 μmol, 2.0 equiv) to afford the crude product. This was purified by flash column chromatography (silica, 0–20% EtOAc in hexane) to give the desired product as a white solid (36.1 mg, 61.4 μmol, 61%).

^{1}H NMR (500 MHz, $CDCl_3$) δ 7.79 (d, J = 7.6 Hz, 2H), 7.60 – 7.55 (m, 6H), 7.45 (s, 1H), 7.42 – 7.33 (m, 9H), 7.24 (d, J = 7.7 Hz, 2H), 5.59 (s, 2H), 1.34 (s, 12H).

^{13}C NMR (126 MHz, $CDCl_3$) δ 143.5, 137.9, 135.6, 135.4, 135.2, 130.3, 129.5, 128.5, 127.3, 84.1, 53.8, 25.0.

^{11}B NMR (128 MHz, $CDCl_3$) δ 31.8.

IR (ATR, film) ν_{max} 2978, 1614, 1485, 1431, 1360, 1323, 1267, 1142, 1090, 1045, 858 cm^{-1}.

HRMS (ESI) Calculated for 590.2029 *m*/*z*, found 590.2029 *m*/*z* [$C_{33}H_{34}BGeN_3O_2$+H]$^+$.

1-(4-Fluorophenyl)-4-(triphenylgermyl)-1*H*-1,2,3-triazole (6)

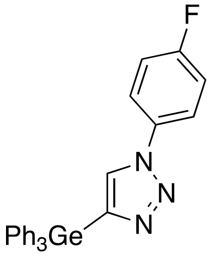

Prepared according to **General Procedure E** using ethynyltriphenylgermane (65.8 mg, 200 μmol, 1.0 equiv) and 4-fluorophenyl azide (24.7 μL, 220 μmol, 1.1 equiv) to afford the crude product. This was purified by flash column chromatography (silica, 10–20% EtOAc in hexane, followed by acetone flush) to give the desired product as a pale yellow solid (84.8 mg, 182 μmol, 91%).

^{1}H{^{19}F} NMR (500 MHz, $CDCl_3$) δ 7.89 (s, 1H), 7.75 – 7.68 (m, 2H), 7.67 – 7.63 (m, 6H), 7.47 – 7.37 (m, 9H), 7.22 – 7.16 (m, 2H).

^{13}C NMR (126 MHz, $CDCl_3$) δ 162.5 (d, $^{1}J_{CF}$ = 248.8 Hz), 144.2, 135.3, 135.1, 133.5, 129.7, 128.7, 128.6, 122.8 (d, $^{3}J_{CF}$ = 8.9 Hz), 116.8 (d, $^{2}J_{CF}$ = 23.4 Hz).

^{19}F{^{1}H} NMR (470 MHz, $CDCl_3$) δ −112.41.

IR (ATR, film) ν_{max} 3134, 2924, 2320, 1558, 1516, 1431, 1094, 1038, 847, 750 cm^{-1}.

HRMS (ESI) Calculated for 468.0926 *m*/*z*, found 468.0928 *m*/*z* [$C_{26}H_{20}FGeN_3$+H]$^+$.

1-(*p*-Tolyl)-4-(triphenylgermyl)-1*H*-1,2,3-triazole (7)

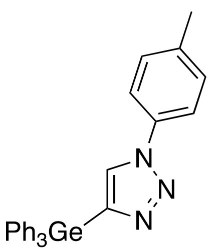

Prepared according to **General Procedure E** using ethynyltriphenylgermane (65.8 mg, 200 μmol, 1.0 equiv) and *p*-tolyl azide (29.3 mg, 220 μmol, 1.1 equiv) to afford the crude product. This was purified by flash column chromatography (silica, 10% EtOAc in hexane) to give the desired product as a yellow oil (86.2 mg, 187 μmol, 93%).

^{1}**H NMR (500 MHz, CDCl$_3$)** δ 7.90 (s, 1H), 7.67 – 7.63 (m, 6H), 7.62 – 7.59 (m, 2H), 7.47 – 7.35 (m, 9H), 7.29 (d, *J* = 8.3 Hz, 2H), 2.41 (s, 3H).

^{13}**C NMR (126 MHz, CDCl$_3$)** δ 143.7, 138.8, 135.3, 135.3, 134.9, 130.3, 129.6, 128.6, 128.5, 120.8, 21.2.

IR (ATR, film) ν_{max} 3067, 3048, 2922, 1558, 1520, 1432, 1094, 1036, 816 cm^{-1}.

HRMS (ESI) Calculated for 464.1177 *m/z*, found 464.1178 *m/z* [$C_{27}H_{23}GeN_3$+H]$^+$.

(*E*)-1-Styryl-4-(triphenylgermyl)-1*H*-1,2,3-triazole (8)

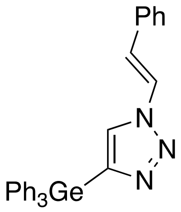

Prepared according to **General Procedure E** using ethynyltriphenylgermane (65.8 mg, 200 µmol, 1.0 equiv) and (*E*)-(2-azidovinyl)benzene (31.9 mg, 220 µmol, 1.1 equiv) to afford the crude product. This was purified by flash column chromatography (silica, 0–30% EtOAc in hexane) to give the desired product as a yellow solid (86.3 mg, 182 µmol, 91%).

^{1}**H NMR (700 MHz, CDCl$_3$)** δ 7.85 (s, 1H), 7.83 (d, *J* = 14.7 Hz, 1H), 7.70 – 7.64 (m, 6H), 7.48 – 7.40 (m, 11H), 7.39 (t, *J* = 7.6 Hz, 2H), 7.36 – 7.31 (m, 1H), 7.16 (d, *J* = 14.7 Hz, 1H).

^{13}**C NMR (176 MHz, CDCl$_3$)** δ 143.6, 135.2, 135.0, 133.8, 129.6, 129.1, 128.7, 128.5, 127.8, 126.7, 122.9, 121.7.

IR (ATR, film) ν_{max} 3051, 1655, 1485, 1431, 1265, 1200, 1186, 1092, 1028, 941 cm^{-1}.

HRMS (ESI) Calculated for 476.1177 *m/z*, found 476.1185 *m/z* [$C_{28}H_{23}GeN_3$+H]$^+$.

2-(4-(Triphenylgermyl)-1*H*-1,2,3-triazol-1-yl)ethan-1-ol (9)

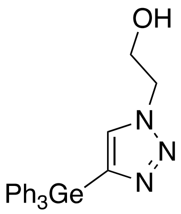

Prepared according to **General Procedure E** using ethynyltriphenylgermane (65.8 mg, 200 µmol, 1.0 equiv) and 2-azidoethan-1-ol (19.2 mg, 220 µmol, 1.1 equiv) to afford the crude product. This was purified by flash column chromatography (silica, 0–100% EtOAc in hexane) to give the desired product as a white solid (37.5 mg, 90.1 µmol, 45%).

^{1}**H NMR (700 MHz, CDCl$_3$)** δ 7.66 – 7.54 (m, 7H), 7.52 – 7.32 (m, 9H), 4.52 – 4.47 (m, 2H), 4.07 (q, *J* = 5.0 Hz, 2H), 2.46 (t, *J* = 5.8 Hz, 1H).

^{13}**C NMR (176 MHz, CDCl$_3$)** δ 142.7, 135.2, 135.1, 131.5, 129.5, 128.4, 61.1, 52.2.

IR (ATR, film) ν_{max} 1485, 1431, 1200, 1188, 1092, 1057, 1026, 999 cm^{-1}.

HRMS (ESI) Calculated for 418.0969 *m/z*, found 418.0963 *m/z* [$C_{22}H_{21}GeN_3O$+H]$^+$.

N-(Pyrimidin-2-yl)-4-(4-(triphenylgermyl)-1*H*-1,2,3-triazol-1-yl)benzenesulfonamide (10)

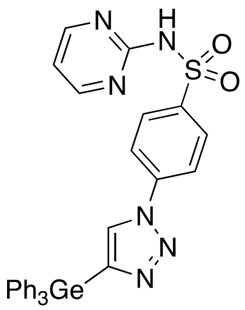

Prepared according to **General Procedure E** using ethynyltriphenylgermane (65.8 mg, 200 µmol, 1.0 equiv) and 4-azido-*N*-(pyrimidin-2-yl)benzenesulfonamide (60.8 mg, 220 µmol, 1.1 equiv) to afford the crude product. This was purified by flash column chromatography (silica, 0–100% EtOAc in hexane) to give the desired product as a pale-yellow solid (23.0 mg, 38.1 µmol, 19%).

^{1}H NMR (400 MHz, CDCl$_3$) δ 10.96 (s, 1H), 8.61 (d, *J* = 5.0 Hz, 2H), 8.33 – 8.25 (m, 2H), 7.98 (s, 1H), 7.95 – 7.87 (m, 2H), 7.66 – 7.57 (m, 6H), 7.48 – 7.36 (m, 9H), 7.00 (t, *J* = 4.9 Hz, 1H).

^{13}C NMR (176 MHz, CDCl$_3$) δ 158.8, 156.7, 145.1, 140.3, 139.4, 135.2, 134.8, 130.6, 129.8, 128.7, 128.4, 120.4, 116.2.

IR (ATR, film) ν_{max} 1580, 1505, 1485, 1431, 1410, 1344, 1163, 1092, 1030, 947, 801 cm^{-1}.

HRMS (ESI) Calculated for 607.0966 *m*/*z*, found 607.0969 *m*/*z* $[C_{30}H_{24}GeN_6O_2S+H]^+$.

10-(4-(2-(4-(Triphenylgermyl)-1*H*-1,2,3-triazol-1-yl)ethoxy)phenyl)-10*H*-phenothiazine (11)

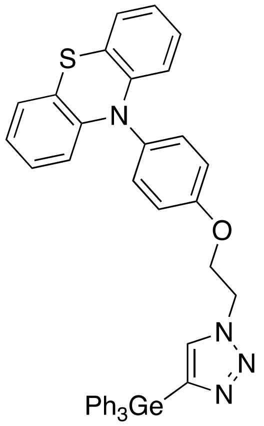

Prepared according to **General Procedure E** using ethynyltriphenylgermane (65.8 mg, 200 µmol, 1.0 equiv) and 10-(4-(2-azidoethoxy)phenyl)-10*H*-phenothiazine (79.3 mg, 220 µmol, 1.1 equiv) to afford the crude product. This was purified by flash column chromatography (silica, 0–100% EtOAc in hexane) to give the desired product as an off-white solid (61.9 mg, 89.8 µmol, 45%).

^{1}H NMR (500 MHz, CDCl$_3$) δ 7.78 (s, 1H), 7.65 – 7.59 (m, 6H), 7.45 – 7.35 (m, 9H), 7.31 – 7.27 (m, 2H), 7.03 – 6.97 (m, 4H), 6.85 – 6.75 (m, 4H), 6.21 – 6.08 (m, 2H), 4.86 (t, *J* = 5.0 Hz, 2H), 4.44 (t, *J* = 5.1 Hz, 2H).

^{13}C NMR (126 MHz, CDCl$_3$) 157.6, 144.6, 143.2, 135.3, 135.3, 134.4, 132.6, 131.9, 129.6, 128.5, 127.0, 126.8, 122.5, 119.9, 116.6, 115.8, 66.9, 49.4.

IR (ATR, film) ν_{max} 1605, 1508, 1460, 1443, 1429, 1300, 1237, 1094, 1038, 1028, 909 cm^{-1}.

HRMS (ESI) Calculated for 691.1581 *m*/*z*, found 691.1583 *m*/*z* $[C_{40}H_{32}GeN_4OS+H]^+$.

4-(2-(4-(Triphenylgermyl)-1*H*-1,2,3-triazol-1-yl)ethoxy)-2*H*-chromen-2-one (12)

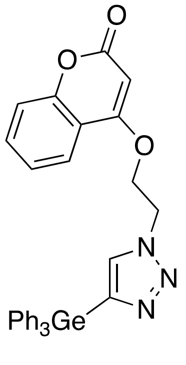

Prepared according to **General Procedure E** using ethynyltriphenylgermane (65.8 mg, 200 µmol, 1.0 equiv) and 4-(2-azidoethoxy)-2*H*-chromen-2-one (50.9 mg, 220 µmol, 1.1 equiv.) to afford the crude product. This was purified by flash column chromatography (silica, 0–60% EtOAc in hexane) to give the desired product as a white solid (37.5 mg, 66.9 µmol, 33%).

^{1}H NMR (500 MHz, CDCl$_3$) δ 7.68 (s, 1H), 7.61 – 7.52 (m, 8H), 7.43 – 7.37 (m, 3H), 7.37 – 7.29 (m, 7H), 7.16 (ddd, *J* = 8.2, 7.4, 1.1 Hz, 1H), 5.66 (s, 1H), 4.93 (d, *J* = 5.1 Hz, 2H), 4.53 (t, *J* = 5.1 Hz, 2H).

^{13}C NMR (126 MHz, CDCl$_3$) δ 164.7, 162.3, 153.4, 143.8, 135.2, 135.1, 132.8, 131.4, 129.6, 128.6, 124.3, 122.7, 117.1, 115.2, 91.4, 67.3, 48.5.

IR (ATR, film) ν_{max} 2986, 1972, 2901, 1721, 1624, 1431, 1379, 1240, 1109, 1092, 1047, 1028 cm^{-1}.

HRMS (ESI) Calculated for 562.1181 *m*/*z*, found 562.1178 *m*/*z* [$C_{31}H_{25}GeN_3O_3$+H]$^+$.

1-Phenyl-2-(4-(triphenylgermyl)-1*H*-1,2,3-triazol-1-yl)ethan-1-one (13)

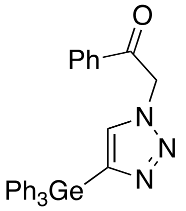

Prepared according to **General Procedure E** using ethynyltriphenylgermane (65.8 mg, 200 μmol, 1.0 equiv) and 2-azido-1-phenylethan-1-one (35.5 mg, 220 μmol, 1.1 equiv) to afford the crude product. This was purified by flash column chromatography (silica, 0–100% EtOAc in hexane) to give the desired product as a pale-yellow solid (78.7 mg, 161 μmol, 80%).

^{1}H NMR (500 MHz, CDCl$_3$) δ 8.01 – 7.96 (m, 2H), 7.70 (s, 1H), 7.69 – 7.64 (m, 1H), 7.64 – 7.60 (m, 6H), 7.57 – 7.51 (m, 2H), 7.45 – 7.32 (m, 9H), 5.90 (s, 2H).

^{13}C NMR (126 MHz, CDCl$_3$) δ 190.5, 143.4, 135.4, 135.3, 134.7, 134.2, 132.4, 129.5, 129.3, 128.5, 128.3, 55.1.

IR (ATR, film) ν_{max} 1703, 1485, 1451, 1431, 1231, 1092, 1055, 995 cm^{-1}.

HRMS (ESI) Calculated for 492.1126 *m*/*z*, found 492.1133 *m*/*z* [$C_{28}H_{23}GeN_3O$+H]$^+$.

1-Benzyl-4-(triphenylgermyl)-1*H*-1,2,3-triazole (14)

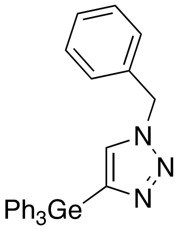

Prepared according to **General Procedure E** using triethyl(ethynyl)germane (37.0 mg, 200 μmol, 1.0 equiv) and benzyl azide (27.5 μL, 220 μmol, 1.1 equiv) to afford the crude product. This was purified by flash column chromatography (silica, 30% EtOAc in hexane) to give the desired product as a white solid (49.6 mg, 107 μmol, 54%).

^{1}H NMR (500 MHz, CDCl$_3$) δ 7.61 – 7.55 (m, 6H), 7.48 (s, 1H), 7.44 – 7.31 (m, 12H), 7.26 – 7.22 (m, 2H), 5.58 (s, 2H).

^{13}C NMR (126 MHz, CDCl$_3$) δ 143.4, 135.4, 135.2, 135.1, 130.4, 129.5, 129.2, 128.7, 128.5, 128.0, 53.8.

IR (ATR, film) ν_{max} 3067, 3049, 1485, 1456, 1429, 1192, 1092, 1045, 1028, 999 cm^{-1}.

HRMS (ESI) Calculated for 464.1177 *m*/*z*, found 464.1179 *m*/*z* [$C_{27}H_{23}GeN_3$+H]$^+$.

1-Benzyl-4-(triethylgermyl)-1*H*-1,2,3-triazole (15)

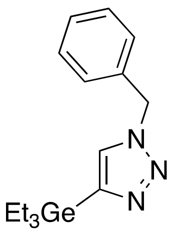

Prepared according to **General Procedure E** using triethyl(ethynyl)germane (92.4 mg, 500 μmol, 1.0 equiv) and benzyl azide (73.2 mg, 550 μmol, 1.1 equiv) to afford the crude product. This was purified by flash column chromatography (silica, 0–20% EtOAc in hexane) to give the desired product as a white solid (157 mg, 494 μmol, 99%).

^{1}H NMR (500 MHz, CDCl$_3$) δ 7.42 – 7.32 (m, 4H), 7.24 (dd, *J* = 7.9, 1.7 Hz, 2H), 5.57 (s, 2H), 1.12 – 0.92 (m, 15H).

^{13}C NMR (126 MHz, CDCl$_3$) δ 145.0, 135.2, 129.0, 128.5, 128.4, 127.9, 53.5, 8.9, 4.5.

IR (ATR, film) ν_{max} 2949, 2930, 2905, 2870, 1497, 1456, 1190, 1098, 1045, 1022 cm^{-1}.

HRMS (ESI) Calculated for 320.1177 *m/z*, found 320.1190 *m/z* [$C_{15}H_{23}GeN_3$+H]$^+$.

1-Benzyl-4-(dimethyl(*o*-tolyl)germyl)-1*H*-1,2,3-triazole (16)

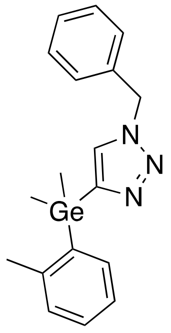

Prepared according to **General Procedure E** using ethynyldimethyl(*o*-tolyl)germane (43.8 mg, 200 µmol, 1.0 equiv) and benzyl azide (27.5 µL, 220 µmol, 1.1 equiv) to afford the crude product. This was purified by flash column chromatography (silica, 30% EtOAc in hexane) to give the desired product as a colourless oil (42.5 mg, 121 µmol, 60%).

^{1}H NMR (500 MHz, CDCl$_3$) δ 7.43 – 7.30 (m, 5H), 7.29 – 7.21 (m, 3H), 7.18 – 7.11 (m, 2H), 5.56 (s, 2H), 2.37 (s, 3H), 0.74 (s, 6H).

^{13}C NMR (126 MHz, CDCl$_3$) δ 146.6, 143.3, 137.7, 135.1, 134.1, 130.0, 129.4, 129.2, 128.7, 128.6, 128.1, 125.3, 53.7, 23.2, −1.5.

IR (ATR, film) ν_{max} 2911, 2311, 1558, 1456, 1192, 1045, 806 cm^{-1}.

HRMS (ESI) Calculated for 376.0840 *m/z*, found 376.0835 *m/z* [$C_{18}H_{21}GeN_3$+Na]$^+$.

1-Benzyl-4-(benzyldimethylgermyl)-1*H*-1,2,3-triazole (17)

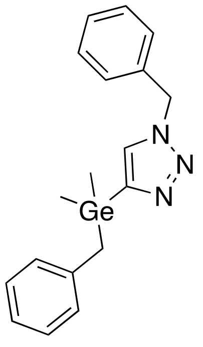

Prepared according to **General Procedure E** using benzyl(ethynyl)dimethylgermane (43.8 mg, 200 µmol, 1.0 equiv) and benzyl azide (27.5 µL, 220 µmol, 1.1 equiv) to afford the crude product. This was purified by flash column chromatography (silica, 30% EtOAc in hexane) to give the desired product as a colourless oil (35.8 mg, 102 µmol, 51%).

^{1}H NMR (500 MHz, CDCl$_3$) δ 7.42 – 7.32 (m, 3H), 7.22 (dd, *J* = 7.4, 2.1 Hz, 2H), 7.14 – 7.09 (m, 3H), 7.02 (t, *J* = 7.3 Hz, 1H), 6.95 – 6.90 (m, 2H), 5.52 (s, 2H), 2.48 (s, 2H), 0.40 (s, 6H).

^{13}C NMR (126 MHz, CDCl$_3$) δ 146.0, 140.2, 135.1, 129.2, 128.7, 128.4, 128.3, 128.1, 128.0, 124.3, 53.7, 25.4, −3.5.

IR (ATR, film) ν_{max} 3024, 2909, 2311, 1491, 1456, 1192, 1045, 808, 758 cm^{-1}.

HRMS (ESI) Calculated for 354.1020 *m/z*, found 354.1025 *m/z* [$C_{18}H_{21}GeN_3$+H]$^+$.

1-Methyl 4-(2-(4-(triethylgermyl)-1*H*-1,2,3-triazol-1-yl)ethyl) cubane-1,4-dicarboxylate (18)

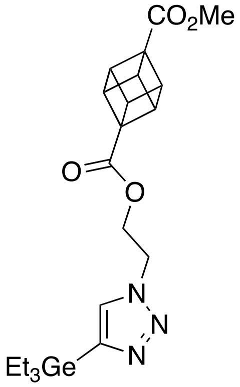

Prepared according to **General Procedure E** using triethyl(ethynyl)germane (37.0 mg, 200 µmol, 1.0 equiv) and 1-(2-azidoethyl) 4-methylcubane-1,4-dicarboxylate (60.6 mg, 220 µmol, 1.1 equiv) to afford the crude product. This was purified by flash column chromatography (silica, 0–20% EtOAc in hexane) to give the desired product as a white solid (15.9 mg, 50.0 µmol, 25%).

^{1}H NMR (500 MHz, $CDCl_3$) δ 7.49 (s, 1H), 4.67 (t, *J* = 5.4 Hz, 2H), 4.50 (t, *J* = 5.3 Hz, 2H), 4.25 – 4.15 (m, 6H), 3.70 (s, 3H), 1.14 – 0.98 (m, 15H).

^{13}C NMR (126 MHz, $CDCl_3$) δ 171.9, 170.9, 144.8, 129.2, 62.6, 56.0, 55.6, 51.8, 48.5, 47.2, 47.1, 9.0, 4.7.

IR (ATR, film) ν_{max} 2951, 1721, 1435, 1319, 1217, 1192, 1088, 841 cm^{-1}.

HRMS (ESI) Calculated for 462.1443 *m*/*z*, found 462.1434 *m*/*z* $[C_{21}H_{29}GeN_3O_4+H]^+$.

1-(*p*-Tolyl)-4-(triethylgermyl)-1*H*-1,2,3-triazole (19)

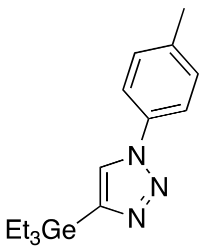

Prepared according to **General Procedure E** using triethyl(ethynyl)germane (37.0 mg, 200 µmol, 1.0 equiv) and *p*-tolyl azide (29.3 mg, 220 µmol, 1.1 equiv) to afford the crude product. This was purified by flash column chromatography (silica, 10% EtOAc in hexane) to give the desired product as a yellow oil (30.4 mg, 95.6 µmol, 48%).

^{1}H NMR (500 MHz, $CDCl_3$) δ 7.86 (s, 1H), 7.62 (d, *J* = 8.2 Hz, 2H), 7.29 (d, *J* = 8.1 Hz, 2H), 2.41 (s, 3H), 1.16 – 1.03 (m, 15H).

^{13}C NMR (126 MHz, $CDCl_3$) δ 145.3, 138.5, 135.1, 130.3, 126.7, 120.7, 21.2, 9.1, 4.7.

IR (ATR, film) ν_{max} 2949, 2930, 2870, 1520, 1456, 1198, 1034, 980, 816, 799 cm^{-1}.

HRMS (ESI) Calculated for 320.1177 *m*/*z*, found 320.1183 *m*/*z* $[C_{15}H_{23}GeN_3+H]^+$.

1-Methyl 3-(2-(4-(triethylgermyl)-1*H*-1,2,3-triazol-1-yl)ethyl) bicyclo[1.1.1]pentane-1,3-dicarboxylate (20)

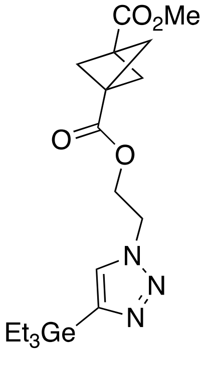

Prepared according to **General Procedure E** using triethyl(ethynyl)germane (37.0 mg, 200 µmol, 1.0 equiv) and 1-(2-azidoethyl) 3-methyl bicyclo[1.1.1]pentane-1,3-dicarboxylate (52.6 mg, 220 µmol, 1.1 equiv) to afford the crude product. This was purified by flash column chromatography (silica, 10–40% EtOAc in hexane, followed by acetone flush) to give the desired product as a colourless oil (34.7 mg, 81.8 µmol, 41%).

^{1}H NMR (500 MHz, $CDCl_3$) δ 7.48 (s, 1H), 4.66 (t, *J* = 5.3 Hz, 2H), 4.47 (t, *J* = 5.3 Hz, 2H), 3.68 (s, 3H), 2.27 (s, 6H), 1.09 – 1.02 (m, 15H).

^{13}C NMR (126 MHz, $CDCl_3$) δ 169.5, 168.6, 144.9, 129.2, 62.9, 52.9, 52.0, 48.3, 37.8, 37.5, 9.1, 4.6.

IR (ATR, film) ν_{max} 2953, 2872, 2311, 1734, 1506, 1288, 1209, 1045 cm^{-1}.

HRMS (ESI) Calculated for 426.1443 *m*/*z*, found 426.1452 *m*/*z* $[C_{18}H_{29}GeN_3O_4+H]^+$.

1-(4-(4,4,5,5-Tetramethyl-1,3,2-dioxaborolan-2-yl)benzyl)-4-(triethylgermyl)-1*H*-1,2,3-triazole (21)

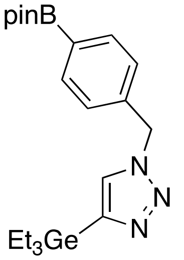

Prepared according to **General Procedure E** using triethyl(ethynyl)germane (37.0 mg, 200 µmol, 1.0 equiv) and 2-(4-(azidomethyl)phenyl)-4,4,5,5-tetramethyl-1,3,2-dioxaborolane (57.0 mg, 220 µmol, 1.1 equiv) with the addition of CsF (60.8 mg, 400 µmol, 2.0 equiv) to afford the crude product. This was purified by flash column chromatography (silica, 0–20% EtOAc in hexane) to give the desired product as a white solid (65.7 mg, 148 µmol, 74%).

^{1}H NMR (400 MHz, $CDCl_3$) δ 7.80 (d, *J* = 8.1 Hz, 2H), 7.35 (s, 1H), 7.23 (d, *J* = 8.0 Hz, 2H), 5.58 (s, 2H), 1.34 (s, 12H), 1.07 – 0.97 (m, 15H).

^{13}C NMR (101 MHz, $CDCl_3$) 145.2, 138.2, 135.6, 128.6, 127.4, 84.1, 53.6, 25.0, 9.0, 4.6.

^{11}B NMR (128 MHz, $CDCl_3$) δ 31.0.

IR (ATR, film) ν_{max} 2949, 1614, 1360, 1144, 1088, 1022, 858 cm^{-1}.

HRMS (ESI) Calculated for 446.2029 *m*/*z*, found 446.2029 *m*/*z* $[C_{21}H_{34}BGeN_3O_2+H]^+$.

(4-(Azidomethyl)phenyl)boronic acid (22)

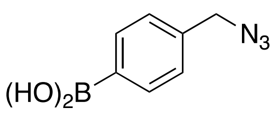

Prepared according to **General Procedure D** using (4-(bromomethyl)phenyl)boronic acid (645 mg, 3.00 mmol, 1.0 equiv) to afford the crude product. This was purified by precipitation from Et_2O with hexane to give the desired product as a white solid (457 mg, 2.58 mmol, 86%).

^{1}H NMR (400 MHz, DMSO-*d*$_6$) δ 8.07 (s, 2H), 7.85 – 7.74 (m, 2H), 7.32 (d, *J* = 8.0 Hz, 2H), 4.44 (s, 2H).

^{13}C NMR (101 MHz, DMSO-*d*$_6$) δ 137.2, 134.5, 127.4, 53.6.

^{11}B NMR (128 MHz, DMSO-*d*$_6$) δ 28.5.

Spectral data consistent with the literature.[17]

2-(4-(Azidomethyl)phenyl)-6-methyl-1,3,6,2-dioxazaborocane-4,8-dione (23)

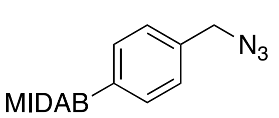

A flask was charged with (4-(hydroxymethyl)phenyl)boronic acid (760 mg, 5.00 mmol, 1.0 equiv.), 2,2'-(methylazanediyl)diacetic acid (736 mg, 5.00 mmol, 1.0 equiv.) and PhMe (25 mL). The flask was fitted with a Dean-Stark trap, filled with PhMe, and the flask was heated to 140 ºC with stirring for 20 h. After this time, the mixture was cooled to RT and concentrated *in vacuo* to afford the crude product. This was purified by flash column chromatography (silica, 0–20% acetone in EtOAc) to give 4-(hydroxymethyl)-MIDA boronate as a white solid (132 mg, 10%).

This solid (132 mg, 500 µmol, 1.0 equiv.) was subsequently dissolved in DMF (2.5 mL) under an atmosphere of N_2 and cooled to 0 ºC, before DPPA (130 µL, 600 µmol, 1.2 equiv.) and DBU (90 µL, 600 µmol, 1.2 equiv.) were added sequentially. The mixture was then warmed to RT, before being stirred for 15 h. After this time, the reaction was diluted by addition of EtOAc (20 mL), and the organic phase was washed with H_2O (3 × 10 mL). The organic extract was dried over Na_2SO_4, filtered, and concentrated *in vacuo* to afford the crude product. This was purified by flash column chromatography (silica, 0–20% acetone in EtOAc) to give the desired product as a white solid (111 mg, 385 µmol, 77%).

^{1}H NMR (400 MHz, MeCN-*d*$_3$) δ 7.57 – 7.49 (m, 2H), 7.44 – 7.31 (m, 2H), 4.41 (s, 2H), 4.07 (d, *J* = 17.1 Hz, 2H), 3.90 (d, *J* = 17.1 Hz, 2H), 2.49 (s, 3H).

^{13}C NMR (101 MHz, MeCN-*d*$_3$) δ 169.5, 137.9, 134.0, 128.9, 62.8, 55.1, 48.5.

^{11}B NMR (128 MHz, MeCN-*d*$_3$) δ 11.3.

Spectral data consistent with the literature.[18]

Ethynyldimethyl(phenylethynyl)germane (24)

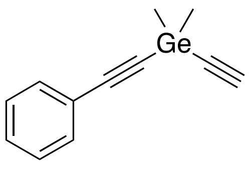

A flame-dried flask was evacuated, backfilled with N_2 (3 ×), then charged with phenylacetylene (110 µL, 1.00 mmol, 1.0 equiv) and Et_2O (5.0 mL). The flask was cooled to −78 °C, *n*-butyllithium in hexane (500 µL, 2.20 M, 1.10 mmol, 1.1 equiv) was added dropwise, and the resulting mixture was stirred at −78 °C for one hour. A separate flame-dried flask was evacuated, backfilled with N_2 (3 ×), then charged with dichlorodimethylgermane (116 µL, 1.00 mmol, 1.0 equiv), Et_2O (5.0 mL) and cooled to 0 °C. The prepared lithium acetylide solution was added dropwise to the dichlorogermane, and the resulting mixture was stirred at 0 °C for 20 min before ethynylmagnesium bromide in THF (3.00 mL, 0.5 M, 1.50 mmol, 1.5 equiv) was added dropwise at the same temperature. This mixture was stirred for two hours whilst being warmed to rt. The mixture was diluted with Et_2O (10 mL) and quenched by the addition of H_2O (10 mL) and the organic phase was collected. The aqueous phase was extracted with Et_2O (3 × 20 mL), and the combined organic extracts were dried over Na_2SO_4, filtered, and concentrated in vacuo to afford the crude product. This was purified by flash column chromatography (silica, hexane) to give the desired product as a colourless oil (79.2 mg, 346 µmol, 35%).

^{1}H NMR (500 MHz, CDCl$_3$) δ 7.48 (d, *J* = 7.9 Hz, 2H), 7.30 (d, *J* = 7.5 Hz, 3H), 2.40 (s, 1H), 0.66 (s, 6H).

^{13}C NMR (126 MHz, CDCl$_3$) δ 132.2, 128.8, 128.3, 122.9, 105.0, 92.7, 89.7, 86.5, 0.8.

IR (ATR, film) ν_{max} 3273, 2160, 2305, 1489, 1443, 1215, 845, 814, 758 cm^{-1}.

HRMS inconclusive due to excess fragmentation.

***tert*-Butyl 4-azido-3,6-dihydropyridine-1(2*H*)-carboxylate (25)**

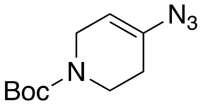

An oven-dried microwave vial was charged with *tert*-butyl 4-(4,4,5,5-tetramethyl-1,3,2-dioxaborolan-2-yl)-3,6-dihydropyridine-1(2*H*)-carboxylate (454 mg, 2.00 mmol, 1.0 equiv.), MeOH (6.0 mL), and anhydrous $CuSO_4$ (49.9 mg, 200 µmol, 1.0 equiv.). NaN_3 (156 mg, 2.40 mmol, 1.2 equiv.) was added and the mixture was stirred at RT open to air for 16 h. The mixture was concentrated *in vacuo* to afford the crude product. This was purified by flash column chromatography (silica, 0–10% Et_2O in hexane) to give the desired product as a red oil (371 mg, 83%).

^{1}H NMR (500 MHz, CDCl$_3$) δ 5.39 – 5.18 (m, 1H), 3.99 – 3.95 (m, 2H), 3.58 (t, *J* = 5.7 Hz, 2H), 2.20 – 2.15 (m, 2H), 1.47 (s, 9H).

^{13}C NMR (176 MHz, CDCl$_3$) δ 154.7, 134.4, 110.4 – 106.9 (m), 80.1, 43.8 – 41.5 (m), 39.5, 28.5, 26.3.

IR (ATR, film) ν_{max} 2976, 2106, 1691, 1413, 1364, 1236, 1157, 1113, 1092, 768 cm^{-1}.

HRMS (ESI) Calculated for 225.1346 *m/z*, found 225.1346 *m/z* $[C_{10}H_{16}N_4O_2+H]^+$.

5-(Dimethylamino)-*N*-(3-(4-(triethylgermyl)-1*H*-1,2,3-triazol-1-yl)propyl)naphthalene-1-sulfonamide (26)

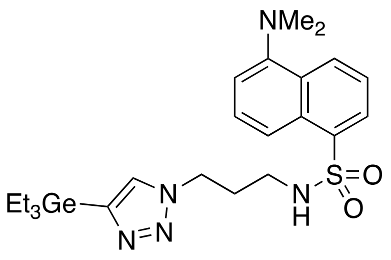

Prepared according to **General Procedure E** using triethyl(ethynyl)germane (9.0 µL, 50 µmol, 1.0 equiv) and 5-(dimethylamino)-*N*-(3-(4-(triethylgermyl)-1*H*-1,2,3-triazol-1-yl)propyl)naphthalene-1-sulfonamide (18.0 mg, 55.0 µmol, 1.1 equiv) to afford the crude product. This was purified by flash column chromatography (silica, 10–100% EtOAc in hexane, followed by acetone flush) to give the desired product as a colourless oil (19.0 mg, 36.6 µmol, 73%).

^{1}H NMR (500 MHz, CDCl$_3$) δ 8.54 (dt, *J* = 8.6, 1.1 Hz, 1H), 8.26 (dt, *J* = 8.6, 1.0 Hz, 1H), 8.20 (dd, *J* = 7.2, 1.3 Hz, 1H), 7.58 (dd, *J* = 8.6, 7.6 Hz, 1H), 7.50 (dd, *J* = 8.6, 7.3 Hz, 1H), 7.43 (s, 1H), 7.19 (dd, *J* = 7.6, 1.0 Hz, 1H), 5.05 (t, *J* = 6.5 Hz, 1H), 4.41 (t, *J* = 6.5 Hz, 2H), 2.88 (s, 6H), 2.86 (q, *J* = 6.3 Hz, 2H), 2.03 (p, *J* = 6.4 Hz, 2H), 1.10 – 1.05 (m, 9H), 1.04 – 0.99 (m, 6H).

^{13}C NMR (126 MHz, CDCl$_3$) δ 152.3, 144.7, 134.6, 130.8, 130.1, 129.8, 129.6, 129.2, 128.8, 123.3, 118.6, 115.5, 46.4, 45.5, 40.2, 30.7, 9.1, 4.7.

HRMS (ESI) Calculated for 520.1796 *m*/*z*, found 520.1793 *m*/*z* [$C_{23}H_{35}GeN_5O_2$+H]$^+$.

(3*S*,9*S*,10*R*,13*R*,14*S*,17*R*)-10,13-Dimethyl-17-((*R*)-6-methylheptan-2-yl)-2,3,4,7,8,9,10,11,12,13,14,15,16,17-tetradecahydro-1*H*-cyclopenta[*a*]phenanthren-3-yl (2-(2-(2-(2-(4-(triethylgermyl)-1*H*-1,2,3-triazol-1-yl)ethoxy)ethoxy)ethoxy)ethyl)carbamate (27)

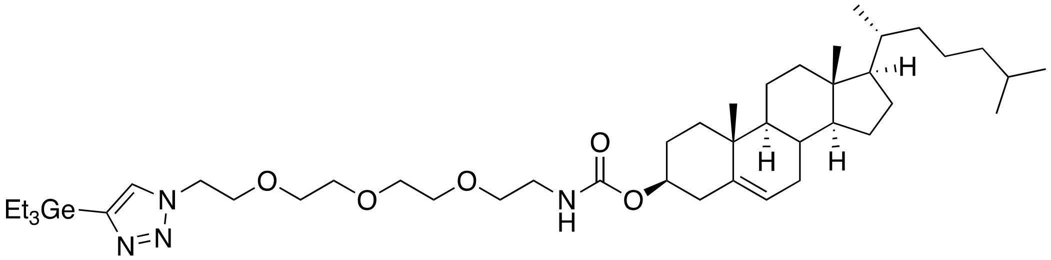

Prepared according to **General Procedure E** using triethyl(ethynyl)germane (9.0 µL, 50 µmol, 1.0 equiv) and (3*S*,9*S*,10*R*,13*R*,14*S*,17*R*)-10,13-dimethyl-17-((*R*)-6-methylheptan-2-yl)-2,3,4,7,8,9,10,11,12,13,14,15,16,17-tetradecahydro-1*H*-cyclopenta[*a*]phenanthren-3-yl (2-(2-(2-(2-azidoethoxy)ethoxy)ethoxy)ethyl)carbamate (34.7 mg, 55.0 µmol, 1.1 equiv) to afford the crude product. This was purified by flash column chromatography (silica, 10–100% EtOAc in hexane, followed by acetone flush) to give the desired product as a colourless oil (23.0 mg, 28.2 µmol, 56%).

^{1}H NMR (500 MHz, CDCl$_3$) δ 7.62 (s, 1H), 5.36 (dt, *J* = 5.0, 2.1 Hz, 1H), 5.13 (d, *J* = 5.9 Hz, 1H), 4.61 – 4.54 (m, 2H), 4.54 – 4.41 (m, 1H), 3.89 (t, *J* = 4.7 Hz, 2H), 3.59 (d, *J* = 4.4 Hz, 8H), 3.53 (t, *J* = 5.1 Hz, 2H), 3.35 (q, *J* = 5.3 Hz, 2H), 2.41 – 2.32 (m, 1H), 2.25 (t, *J* = 12.7 Hz, 1H), 2.05 – 1.74 (m, 6H), 1.63 – 0.89 (m, 39H), 0.86 (dd, *J* = 6.6, 2.3 Hz, 6H), 0.67 (s, 3H).

^{13}C NMR (126 MHz, CDCl$_3$) δ 156.3, 144.3, 140.0, 129.9, 122.6, 74.5, 70.7, 70.7, 70.7, 70.4, 70.3, 69.9, 56.8, 56.3, 50.2, 49.9, 42.5, 40.8, 39.9, 39.7, 38.7, 37.1, 36.7, 36.3, 35.9, 32.0, 32.0, 29.8, 28.4, 28.3, 28.1, 24.4, 24.0, 23.0, 22.7, 21.2, 19.5, 18.9, 12.0, 9.1, 4.7.

HRMS (ESI) Calculated for 817.5257 *m*/*z*, found 817.5277 *m*/*z* [$C_{44}H_{78}GeN_4O_5$+H]$^+$.

2-(4-((4-(Triethylgermyl)-1*H*-1,2,3-triazol-1-yl)methyl)phenyl)thiazole (28)

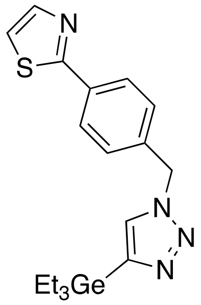

An oven-dried microwave vial was sealed, evacuated, and backfilled with N_2 (3 ×), before being charged with 2-bromothiazole (9.0 µL, 100 µmol, 1.0 equiv) and a 4:1 mixture of degassed toluene/EtOH (0.66 mL, 0.15 M). To this solution aq. 2 M Na_2CO_3 (0.40 mL), KCl (22.4 mg, 300 µmol, 3.0 equiv), and 1-(4-(4,4,5,5-tetramethyl-1,3,2-dioxaborolan-2-yl)benzyl)-4-(triethylgermyl)-1*H*-1,2,3-triazole (53.3 mg, 120 µmol, 1.2 equiv) were added sequentially. After refilling the headspace with N_2, $Pd(PPh_3)_4$ (12.0 mg, 10.0 µmol, 10 mol %) was added, and the reaction mixture was stirred at 100 °C overnight. The reaction mixture was then cooled to rt and the mixture was filtered through celite (eluting with CH_2Cl_2 (10 mL)). The filtrate was dried over Na_2SO_4, filtered, and concentrated in vacuo to afford the crude product. This was purified by flash column chromatography (silica, 10–100% acetone in hexane) and the residue obtained was taken up in EtOH (300 µL, 0.33 M), combined with $ZnCl_2$ (27.3 mg, 200 µmol, 2.0 equiv) and left to stir at rt for three hours. The resulting mixture was filtered, with the filtrate concentrated *in vacuo* leaving a crude yellow oil. This was then washed through silica with Et_2O to give the desired product as a yellow solid (14.0 mg, 34.9 µmol, 35%).

^{1}H NMR (500 MHz, $CDCl_3$) δ 8.00 – 7.94 (m, 2H), 7.87 (d, *J* = 3.3 Hz, 1H), 7.41 (s, 1H), 7.35 (d, *J* = 3.3 Hz, 1H), 7.34 – 7.30 (m, 2H), 5.61 (s, 2H), 1.09 – 0.99 (m, 15H).

^{13}C NMR (126 MHz, $CDCl_3$) δ 145.4, 144.0, 137.1, 134.0, 128.6, 128.5, 127.3, 125.7, 119.4, 53.3, 9.1, 4.7.

IR (ATR, film) ν_{max} 2951, 2928, 2870, 2311, 1653, 1558, 1541, 1506, 1456, 1093, 1043, 769 cm^{-1}.

HRMS (ESI) Calculated for 403.1006 *m/z*, found 403.1000 *m/z* $[C_{18}H_{24}GeN_4S+H]^+$.

1-Benzyl-4-phenyl-1*H*-1,2,3-triazole (29)

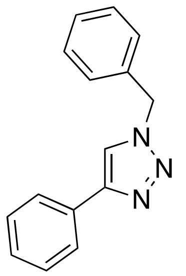

An oven-dried microwave vial was charged with 1-benzyl-4-(triethylgermyl)-1*H*-1,2,3-triazole (31.8 mg, 100 µmol, 1.0 equiv), $Pd_2(dba)_3$ (2.3 mg, 2.50 µmol, 2.5 mol %), and $AgBF_4$ (29.2 mg, 150 µmol, 1.5 equiv). The vial was sealed, evacuated, and backfilled with N_2 (3 ×). DMF (300 µL) and PhI (17.0 µL, 150 µmol, 1.5 equiv) were added and the mixture was heated to 80 °C and stirred for 16 h. After allowing to cool to rt, the vial was unsealed and sat. aq. NH_4Cl (2.0 mL) was added, and the mixture was filtered through cotton wool (eluting with Et_2O (10 mL)). The organic phase was collected, and the aqueous phase was extracted with Et_2O (3 × 20 mL) and the combined organic extracts were dried over Na_2SO_4, filtered and concentrated in vacuo to afford the crude product. This was purified by flash column chromatography (silica, 0–15% EtOAc in hexane) to give the desired product as a white solid (21.4 mg, 91.0 µmol, 91%).

^{1}H NMR (500 MHz, $CDCl_3$) δ 7.86 – 7.78 (m, 2H), 7.66 (s, 1H), 7.43 – 7.36 (m, 5H), 7.31 (ddt, *J* = 7.3, 5.1, 1.2 Hz, 3H), 5.57 (s, 2H).

^{13}C NMR (126 MHz, $CDCl_3$) δ 148.4, 134.8, 130.7, 129.3, 128.9, 128.9, 128.3, 128.2, 125.8, 119.6, 54.4.

Spectral data consistent with the literature.[19]

1-Benzyl-4-bromo-1*H*-1,2,3-triazole (30)

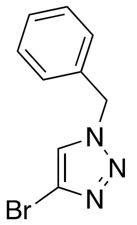

A flask was charged with 1-benzyl-4-(triethylgermyl)-1*H*-1,2,3-triazole (71.3 mg, 224 μmol, 1.0 equiv) and DMF (750 μL). NBS (289 mg, 448 μmol, 2.0 equiv) was added and the mixture was stirred at rt for two hours. Sat. aq. $Na_2S_2O_3$ solution (10 mL) was added followed by CH_2Cl_2 (10 mL). The organic phase was collected, and the aqueous phase was further extracted with CH_2Cl_2 (3 × 10 mL). The combined organic extracts were dried over Na_2SO_4, filtered, and concentrated in vacuo to afford the crude product. This was purified by flash column chromatography (silica, 0–20% EtOAc in hexane) to give the desired product as a white solid (53.4 mg, 224 μmol, >99%).

^{1}H NMR (500 MHz, $CDCl_3$) δ 7.46 (s, 1H), 7.38 (m, 3H), 7.30 – 7.25 (m, 2H), 5.52 (s, 2H).

^{13}C NMR (126 MHz, $CDCl_3$) δ 133.8, 129.3, 129.1, 128.2, 123.7, 120.9, 54.9.

Spectral data consistent with the literature.[20]

4-Bromo-1-(4-(4,4,5,5-tetramethyl-1,3,2-dioxaborolan-2-yl)benzyl)-1*H*-1,2,3-triazole (31)

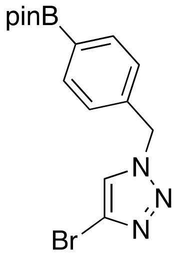

A flask was charged with 1-(4-(4,4,5,5-tetramethyl-1,3,2-dioxaborolan-2-yl)benzyl)-4-(triethylgermyl)-1*H*-1,2,3-triazole (39.6 mg, 89.2 μmol, 1.0 equiv) and DMF (300 μL). NBS (31.8 mg, 178 μmol, 2.0 equiv) was added and the mixture was stirred at rt for two hours. Sat. aq. $Na_2S_2O_3$ solution (10 mL) was added followed by CH_2Cl_2 (10 mL). The organic phase was collected, and the aqueous phase was further extracted with CH_2Cl_2 (2 × 10 mL). The combined organic extracts were dried over Na_2SO_4, filtered, and concentrated in vacuo to afford the crude product. This was purified by flash column chromatography (silica, 0–20% EtOAc in hexane) to give the desired product as a white solid (7.5 mg, 21 μmol, 23%).

^{1}H NMR (400 MHz, $CDCl_3$) δ7.86 – 7.78 (m, 2H), 7.41 (s, 1H), 7.30 – 7.24 (m, 2H), 5.53 (s, 2H), 1.34 (s, 12H).

^{13}C NMR (101 MHz, $CDCl_3$) δ 136.5, 135.7, 127.5, 123.6, 120.9, 84.1, 54.9, 24.9.

^{11}B NMR (128 MHz, $CDCl_3$) δ 30.8.

IR (ATR, film) ν_{max} 2976, 1614, 1398, 1358, 1325, 1263, 1142, 1088, 1042, 1020, 980, 858 cm^{-1}.

HRMS (ESI) Calculated for 364.0827 *m*/*z*, found 364.0836 *m*/*z* $[C_{15}H_{19}BBrN_3O_2+H]^+$.

1-(3-(1-Benzyl-1*H*-1,2,3-triazol-4-yl)thiophen-2-yl)ethan-1-one (32)

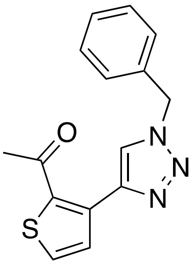

An oven-dried microwave vial was charged with 1-benzyl-4-bromo-1*H*-1,2,3-triazole (18.0 mg, 75.6 μmol, 1.0 equiv), Pd(dtbpf)Cl_2 (4.9 mg, 7.56 μmol, 10 mol %), K_3PO_4 (32.1 mg, 151 μmol, 2.0 equiv), and (2-acetylthiophen-3-yl)boronic acid (15.4 mg, 90.7 μmol, 1.2 equiv). The vial was sealed, evacuated, and backfilled with N_2 (3 ×), before iPrOH (120 μL) and H_2O (150 μL) were added sequentially. The solution was heated to 85 °C and stirred for 16 h. After allowing to cool to rt, the mixture was filtered through celite (eluting with MeOH (10 mL)) and concentrated in vacuo. H_2O (10 mL) and EtOAc (10 mL) were added, the organic phase was collected then the aqueous phase was further extracted with EtOAc (2 × 10 mL). The combined organic extracts were dried over Na_2SO_4, filtered, and concentrated in vacuo to afford the crude product. This was purified by flash column chromatography (silica, 0–15% EtOAc in hexane) to give the desired product as a red solid (19.1 mg, 67.4 μmol, 89%).

^{1}H NMR (500 MHz, $CDCl_3$) δ 8.73 (s, 1H), 8.05 (d, *J* = 5.2 Hz, 1H), 7.54 (d, *J* = 5.2 Hz, 1H), 7.40 – 7.30 (m, 5H), 5.58 (s, 2H), 2.57 (s, 3H).

^{13}C NMR (126 MHz, $CDCl_3$) δ 190.9, 142.8, 136.0, 135.0, 134.9, 131.3, 130.1, 129.2, 128.8, 128.1, 125.6, 54.3, 30.2.

IR (ATR, film) ν_{max} 1662, 1506, 1454, 1408, 1356, 1283, 1213, 1047, 964, 874, 758 cm^{-1}.

HRMS (ESI) Calculated for 284.0852 *m/z*, found 284.0858 *m/z* $[C_{15}H_{13}N_3OS+H]^+$.

4-(Naphthalen-1-yl)-1-(4-(4,4,5,5-tetramethyl-1,3,2-dioxaborolan-2-yl)benzyl)-1*H*-1,2,3-triazole (33)

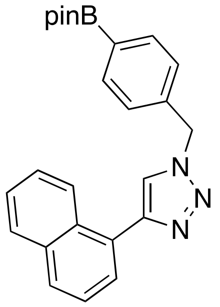

An oven-dried microwave vial was charged with magnesium turnings (3.5 mg, 130 μmol, 1.3 equiv) and a crystal of iodine then sealed, evacuated, and backfilled with N_2 (3 ×). THF (500 μL) was added followed by 1-iodonapthalene (17.5 μL, 120 μmol, 1.2 equiv). The vial was heated gently with a heat gun to initiate Grignard formation and the resulting mixture was stirred for one hour at rt. The vial was decapped under a blanket of N_2 and $ZnBr_2$ (27.0 mg, 120 μmol, 1.2 equiv) was added, with the vial then resealed and the solution stirred for 0.5 h. A separate oven-dried microwave vial was charged with Pd(dtbpf)Cl_2 (0.7 mg, 1.0 μmol, 1.0 equiv) and 4-bromo-1-(4-(4,4,5,5-tetramethyl-1,3,2-dioxaborolan-2-yl)benzyl)-1*H*-1,2,3-triazole (36.4 mg, 100 μmol, 1.0 equiv). The vial was sealed, evacuated, and backfilled with N_2. To this, the 1-naphthylzinc bromide solution was added dropwise and, once addition was completed, the mixture was heated to 45 °C and stirred for 16 h. After this time, the reaction mixture was cooled to rt, and aq. 2 M HCl (5 mL) was added followed by sat. aq. $NaHCO_3$ until a pH of >7 was reached. CH_2Cl_2 (10 mL) was added, and the mixture was transferred to a separating funnel. The organic phase was collected, and the aqueous phase was further extracted with CH_2Cl_2 (3 × 10 mL). The combined organic extracts were dried over Na_2SO_4, filtered, and concentrated in vacuo to afford the crude product. This was purified by flash column chromatography (silica, 0–15% EtOAc in hexane) to give the desired product as a white solid (19.8 mg, 48.1 μmol, 48%).

^{1}H NMR (400 MHz, $CDCl_3$) δ 8.39 – 8.29 (m, 1H), 7.92 – 7.82 (m, 4H), 7.70 (s, 1H), 7.68 (dd, *J* = 7.1, 1.3 Hz, 1H), 7.53 – 7.46 (m, 3H), 7.41 – 7.35 (m, 2H), 5.67 (s, 2H), 1.34 (s, 12H).

^{13}C NMR (101 MHz, $CDCl_3$) δ 147.6, 137.6, 135.8, 134.0, 131.2, 129.1, 128.6, 128.1, 127.6, 127.4, 126.8, 126.1, 125.6, 125.4, 122.6, 84.2, 54.5, 25.0.

^{11}B NMR (128 MHz, $CDCl_3$) δ 30.7.

IR (ATR, film) ν_{max} 2976, 1614, 1406, 1398, 1360, 1325, 1267, 1213, 1167, 1142, 1088, 1049, 1020, 962, 756, 818, 777 cm^{-1}.

HRMS (ESI) Calculated for 412.2191 *m/z*, found 412.2195 *m/z* $[C_{25}H_{26}BN_3O_2+H]^+$.

4-((4-(Triethylgermyl)-1*H*-1,2,3-triazol-1-yl)methyl)phenol (34)

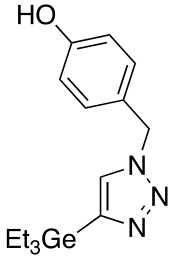

An HPLC vial was charged with 1-(4-(4,4,5,5-tetramethyl-1,3,2-dioxaborolan-2-yl)benzyl)-4-(triethylgermyl)-1*H*-1,2,3-triazole (44.4 mg, 100 μmol, 1.0 equiv), $B(OH)_3$ (12.4 mg, 200 μmol, 2.0 equiv), $Cu(OAc)_2 \cdot H_2O$ (6.0 mg, 30.0 μmol, 30 mol %), and 4 Å MS (50 mg). The vial was capped, a bleed needle was equipped then MeCN (150 μL) and DBU (30.0 μL, 200 μmol, 2.0 equiv) were added, then the mixture was heated at 70 °C for 24 h. After allowing to cool to rt, the mixture was transferred to a separating funnel and diluted with CH_2Cl_2 (10 mL) and 10% aq. NH_3 solution (10 mL). The organic phase was collected, and the aqueous phase was further extracted with CH_2Cl_2 (2 × 10 mL). The combined organic extracts were dried over Na_2SO_4, filtered, and concentrated in vacuo to afford the crude product. This was purified by flash column chromatography (silica, 0–50% EtOAc in hexane) to give the desired product as a brown solid (30.2 mg, 90.4 μmol, 90%).

^{1}H NMR (500 MHz, $CDCl_3$) δ 7.37 (s, 1H), 7.35 – 7.32 (m, 2H), 7.20 – 7.16 (m, 2H), 5.53 (s, 2H), 1.10 – 1.01 (m, 15H).

NB: OH proton not observed.

^{13}C NMR (126 MHz, $CDCl_3$) δ 145.4, 134.7, 133.8, 129.4, 129.4, 128.4, 52.9, 9.0, 4.7.

IR (ATR, film) ν_{max} 2953, 2872, 1492, 1090, 1045, 1017, 097, 806, 758 cm^{-1}.

HRMS (ESI) Calculated for 320.1178 *m*/*z*, found 320.1172 *m*/*z* $[C_{15}H_{23}GeN_3O{-}OH{+}2H]^+$.

1-(4-((4-(Triethylgermyl)-1*H*-1,2,3-triazol-1-yl)methyl)phenyl)piperidine (35)

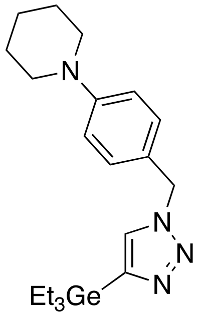

An HPLC vial was charged with 1-(4-(4,4,5,5-tetramethyl-1,3,2-dioxaborolan-2-yl)benzyl)-4-(triethylgermyl)-1*H*-1,2,3-triazole (44.4 mg, 100 μmol, 1.0 equiv), $B(OH)_3$ (12.4 mg, 200 μmol, 2.0 equiv), $Cu(OAc)_2 \cdot H_2O$ (6.0 mg, 30.0 μmol, 30 mol %), and 4 Å MS (50 mg). The vial was capped, a bleed needle was equipped then MeCN (150 μL) and piperidine (20.0 μL, 200 μmol, 2.0 equiv) were added, then the mixture was heated at 70 ºC for 24 h. After allowing to cool to rt, the mixture was transferred to a separating funnel and diluted with CH_2Cl_2 (10 mL) and 10% aq. NH_3 solution (10 mL). The organic phase was collected, and the aqueous phase was further extracted with CH_2Cl_2 (2 × 10 mL). The combined organic extracts were dried over Na_2SO_4, filtered, and concentrated in vacuo to afford the crude product. This was purified by flash column chromatography (silica, 0–20% EtOAc in hexane) to give the desired product as an orange solid (21.7 mg, 54.1 μmol, 54%).

^{1}H NMR (500 MHz, $CDCl_3$) δ 7.32 (s, 1H), 7.16 (d, *J* = 8.6 Hz, 2H), 6.91 (d, *J* = 8.7 Hz, 2H), 5.45 (s, 2H), 3.23 – 3.14 (m, 4H), 1.70 (p, *J* = 5.8 Hz, 4H), 1.59 (q, *J* = 6.2 Hz, 2H), 1.09 – 0.97 (m, 15H).

^{13}C NMR (126 MHz, $CDCl_3$) δ 152.1, 144.8, 129.4, 128.2, 125.9, 116.5, 53.3, 50.4, 25.8, 24.3, 9.1, 4.7.

IR (ATR, film) ν_{max} 2932, 1907, 1870, 1614, 1516, 1452, 1383, 1238, 1190, 1130, 1096, 1045, 1024, 810, 757 cm^{-1}.

HRMS (ESI) Calculated for 403.1912 *m*/*z*, found 403.1894 *m*/*z* $[C_{20}H_{32}GeN_4{+}H]^+$.

5. X-ray diffraction data

X-ray diffraction data for compound **13** were collected at 100 K using a Rigaku FR-X Ultrahigh Brilliance Microfocus RA generator/confocal optics with XtaLAB P200 diffractometer [Mo Kα radiation (λ = 0.71073 Å)]. Data were collected (using a calculated strategy) and processed (including correction for Lorentz, polarization and absorption) using CrysAlisPro.[21] The structure was solved by dual-space methods (SHELXT[22]) and refined by full-matrix least-squares against F^2 (SHELXL-2019/3[23]). Non-hydrogen atoms were refined anisotropically, and hydrogen atoms were refined using a riding model. All calculations were performed using the Olex2[24] interface. Selected crystallographic data are presented in **Table S2**. CCDC 2355570 contains the supplementary crystallographic data for this paper. These data can be obtained free of charge from The Cambridge Crystallographic Data Centre via www.ccdc.cam.ac.uk/structures.

Table S2. Selected crystallographic data.

	**13**
formula	$C_{28}H_{23}N_3OGe$
fw	490.08
crystal description	colourless plate
crystal size [mm^3]	0.09 x 0.09 x 0.01
space group	$P2_12_12_1$
a [Å]	7.19194(13)
b [Å]	15.2354(4)
c [Å]	42.5349(9)
vol [Å]^3	4660.64(18)
Z	8
ρ (calc) [g/cm^3]	1.397
μ [mm^{-1}]	1.340
F(000)	2016.0
reflections collected	52500
independent reflections (R_{int})	10795 (0.0487)
parameters, restraints	596, 144
GooF on F^2	1.090
R_1 [$I > 2\sigma(I)$]s	0.0524
wR_2 (all data)	0.1014
largest diff. peak/hole [e/Å^3]	1.40/-1.01
flack parameter	0.49(3)

6. References

1. W. L. F. Armarego and C. Chai, *Purification of Laboratory Chemicals*, 6th Ed., Elsevier: Oxford, 2009.
2. M. Fishwick, M. G. H. Wallbridge, *J. Chem. Soc. A*, 1971, 57–63.
3. J. Wei, J. Chen, J. M. Miller, *Rapid Commun. Mass Spectrom.* 2001, **15**, 169–181.
4. N. H. El-Dershaby, S. A. El-Hawash, S. E. Kassab, H. G. Daabees, A. E. Abdel Moneim, M. M. M. El-Miligy, *Pharmaceuticals*, 2022, **15**, 1165.
5. T. H. Sum, T. J. Sum, W. R. J. D. Galloway, S. Collins, D. G. Twigg, F. Hollfelder, D. R. Spring, *Molecules*, 2016, **21**, 1230.
6. A. Aljabour, H. Awada, L. Song, H. Sun, S. Offenthaler, F. Yari, M. Bechmann, M. C. Scharber, W. Schöfberger, *Angew. Chem. Int. Ed.*, 2023, **62**, e202302208.
7. H. Wongoso, M. Ono, T. Yamasaki, K. Kumata, M. Higuchi, M.-R. Zhang, M. J. Fulham, A. Katsifis, P. A. Keller, *RSC Med. Chem.*, 2023, **14**, 858–868.
8. L.-J. Chen, C.-J. Kuo, C.-F. Liang, *J. Org. Chem.*, 2023, **88**, 10501–10507.
9. M. V. Vita, J. Waser, *Org. Lett.*, 2013, **15**, 3246–3249.
10. X. Li, J.-N. Song, S. Karmakar, Y. Lu, Y. Lv, P. Liao, Z. Liu, *Chem. Commun.*, 2022, **58**, 13783–13786.
11. P. T. G. Rabet, G. Fumagalli, S. Boyd, M. F. Greaney, *Org. Lett.*, 2016, **18**, 1646–1649.
12. C. Russo, M. C. Leech, J. M. Walsh, J. I. Higham, L. Giannessi, E. Lambert, C. Kiaku, D. L. Poole, J. Mason, C. A. I. Goodall, P. Devo, M. Giustiniano, M. Radi, K. Lam, *Angew. Chem. Int. Ed.*, 2023, **62**, e202309563.
13. L. Ren, N. Jiao, *Chem. Commun.*, 2014, **50**, 3706–3709.
14. L. Donnier-Valentin, S. Kassamba, J. Legros, C. Fressigné, D. Vuluga, R. C. D. Brown, B. Lunclau, M. De Paolis, *Org. Lett.*, 2023, **25**, 8580–8584.
15. M. Mato, M. Montesinos-Magraner, A. R. Sugranyes, A. M. Echavarren, *J. Am. Chem. Soc.*, 2021, **143**, 10760–10769.
16. K. Kojima, S. Uchida, H. Kinoshita, K. Miura, *Org. Lett.* 2021, **23**, 4598–4602.
17. J. Zhou, P. Stapleton, S. Haider, J. Healy, *Bioorg. Med. Chem.*, 2018, **26**, 2921–2927.
18. C. K. L. Gordon, D. Wu, A. Pusuluri, T. A. Feagin, A. T. Csordas, M. S. Eisenstein, C. J. Hawker, J. Niu, H. T. Soh, *ACS Chem. Biol.*, 2019, **14**, 2652–2662.
19. P. Du, J.-F. Li, Y.-Y. Liu, J.-F. Ma, G.-H. Xu, *Dalton Trans.*, 2020, **49**, 3715–3722.
20. D. B. Emerin, V. V. Fokin, *J. Am. Chem. Soc.*, 2021, **143**, 18374–18379.
21. *CrysAlisPro* v1.171.43.109a Rigaku Oxford Diffraction, Rigaku Corporation, *Tokyo*, Japan, 2023.
22. G. Sheldrick, *Acta Crystallogr., Sect. A: Found. Adv.* 2015, **71**, 3–8.
23. G. Sheldrick, *Acta Crystallogr. Sect. C: Struct. Chem.* 2015, **71**, 3–8.
24. O. V. Dolomanov, L. J. Bourhis, R. J. Gildea, J. A. K. Howard, H. Puschmann, *J. Appl. Crystallogr.* 2009, **42**, 339–341.

7. Spectra

S1 – ^{1}H

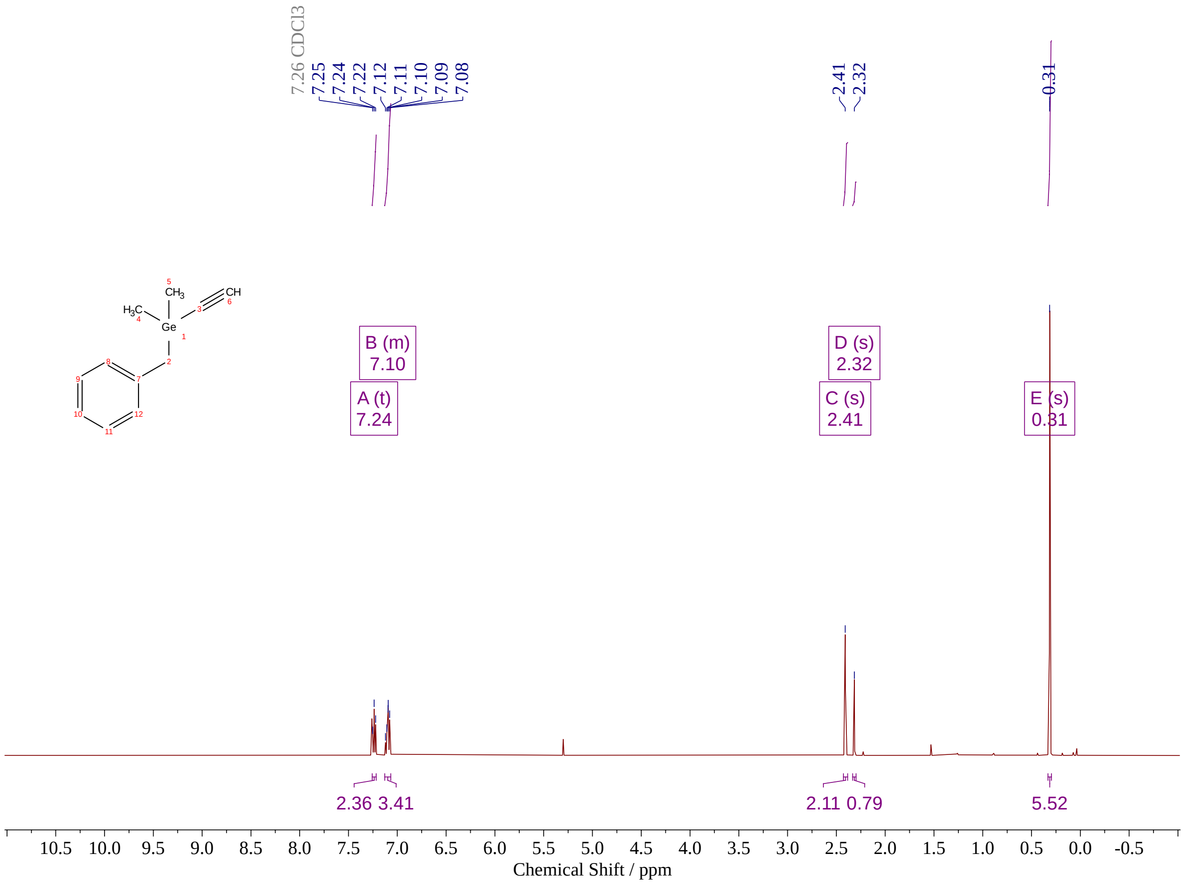

^{13}C DEPTQ

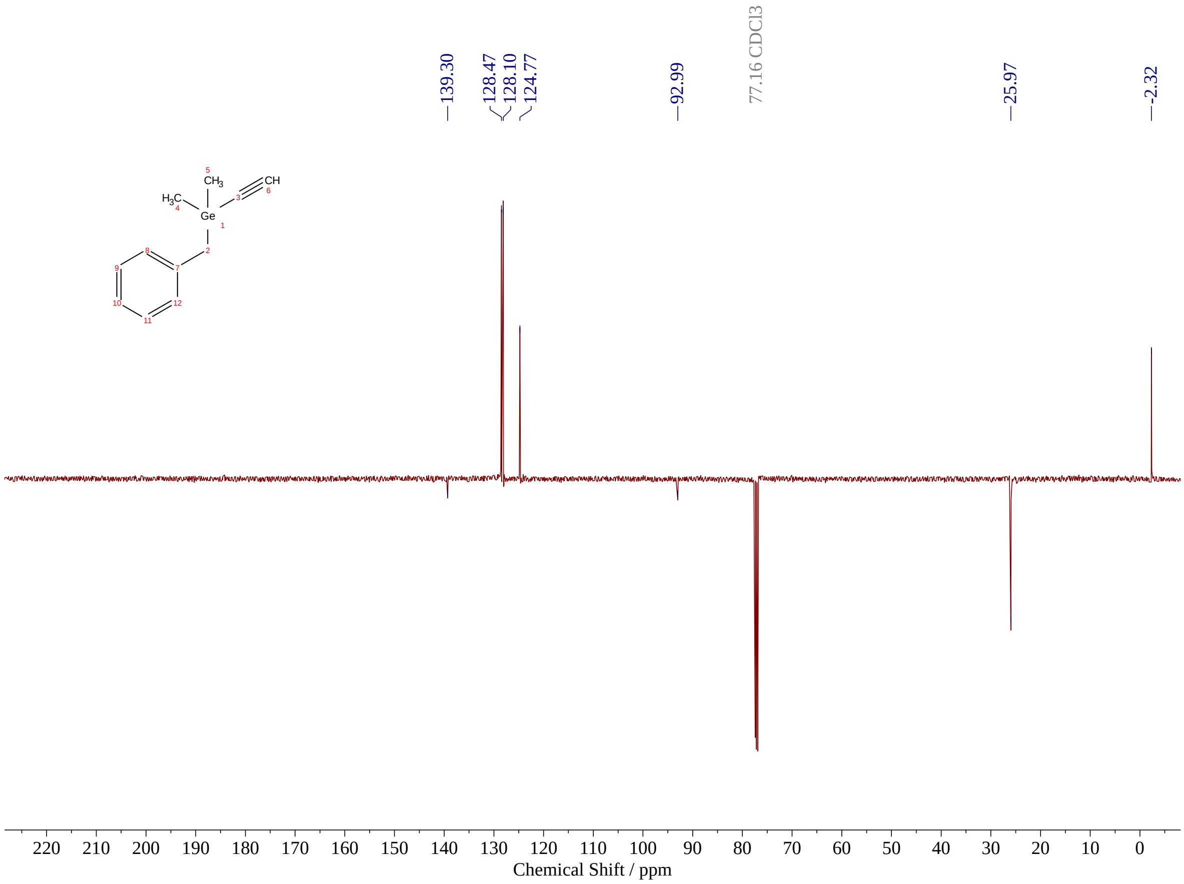

S2 – ^{1}H

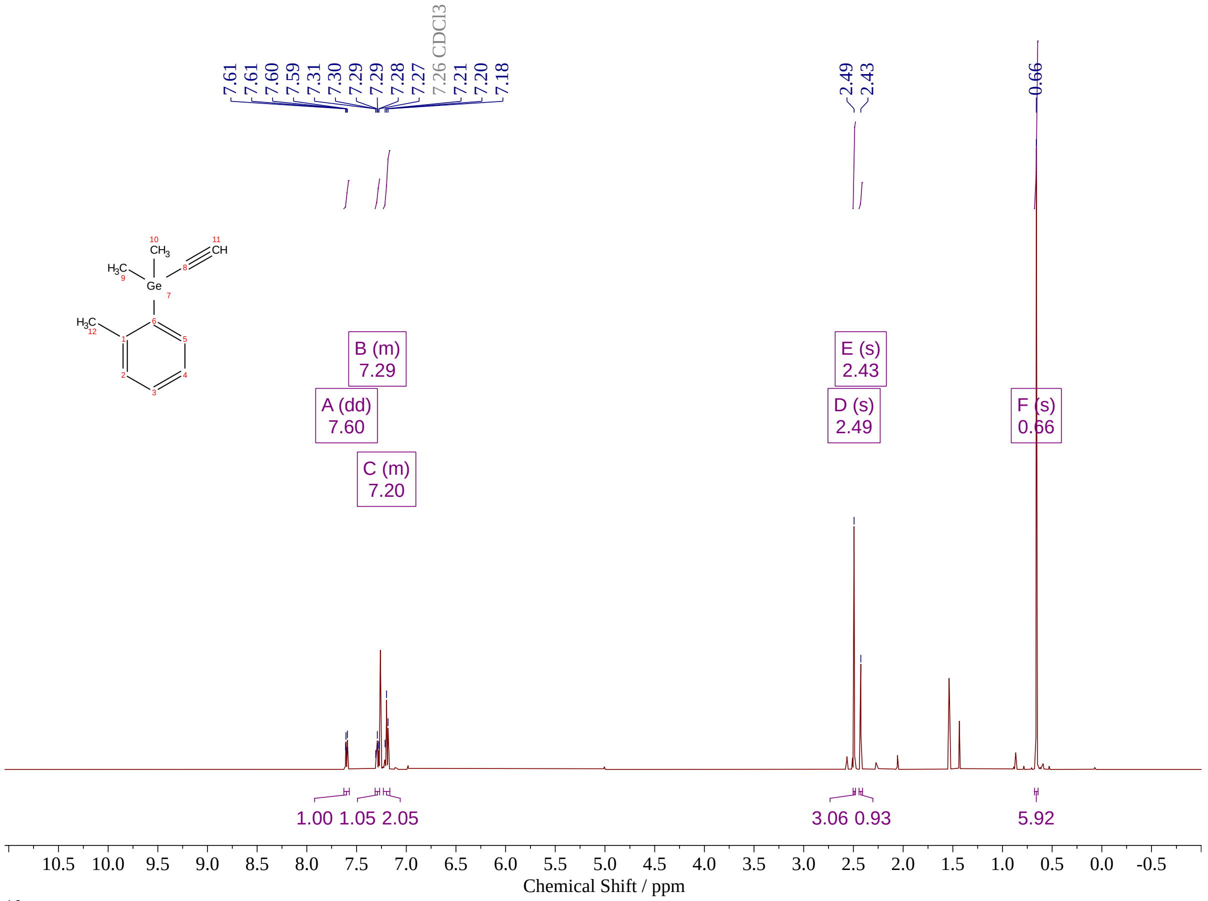

^{13}C DEPTQ

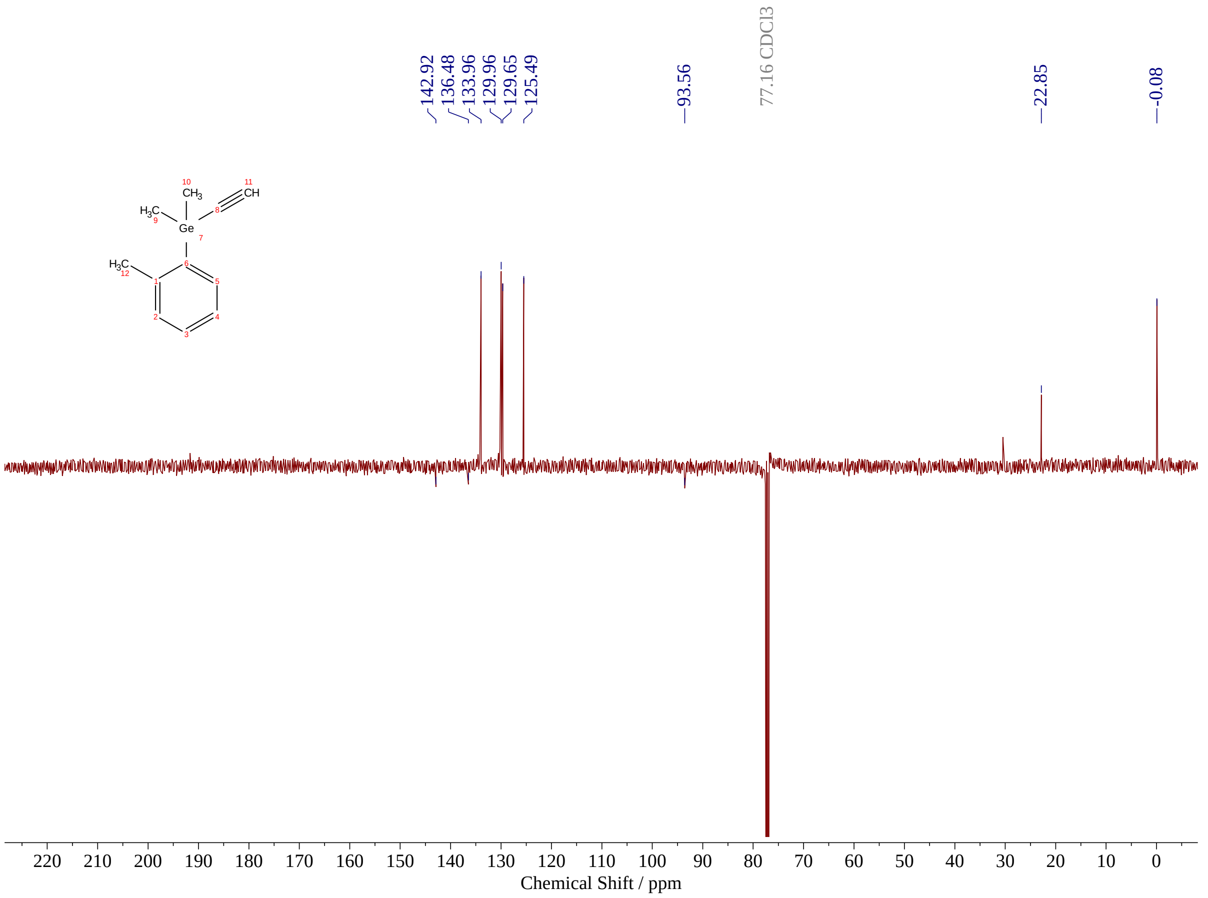

S3 – ^{1}H

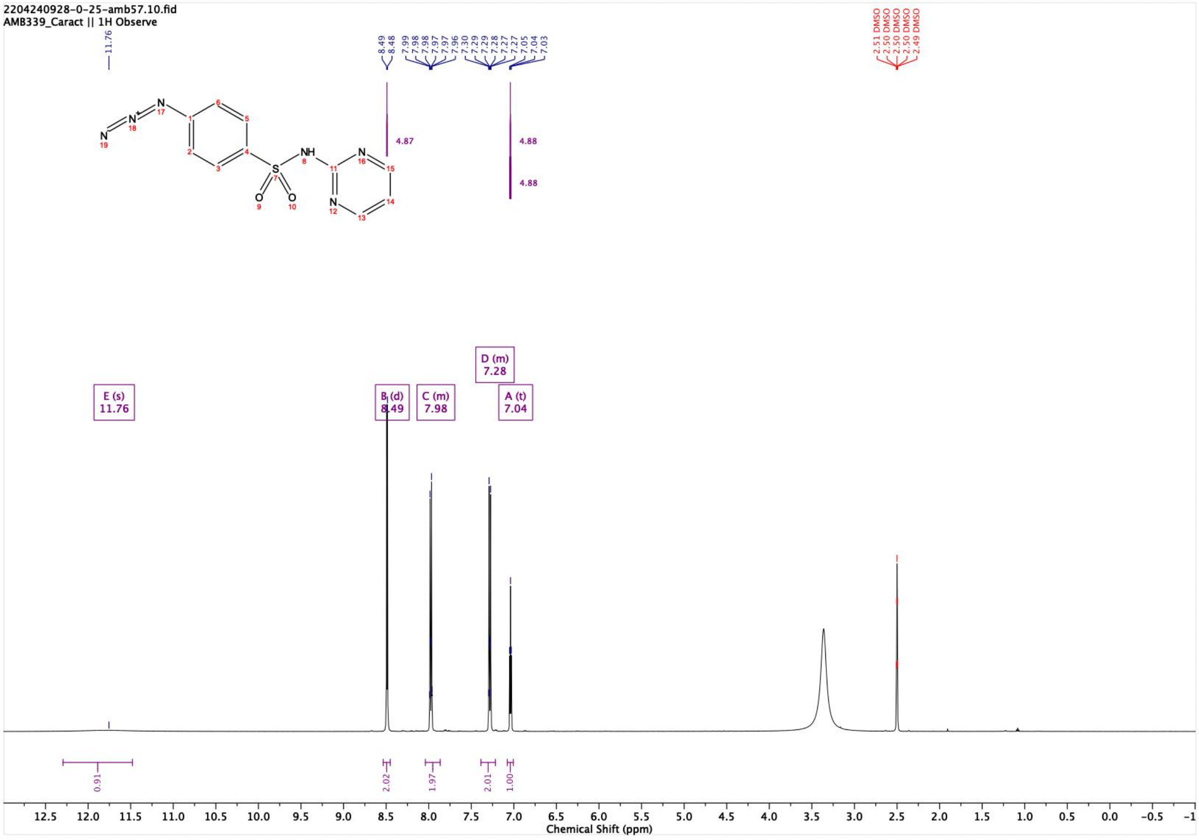
2204240928-0-25-amb57.10.fid
AMB339_Caract || 1H Observe
E (s) 11.76
B (d) 8.49
C (m) 7.98
D (m) 7.28
A (t) 7.04
Chemical Shift (ppm)

^{13}C

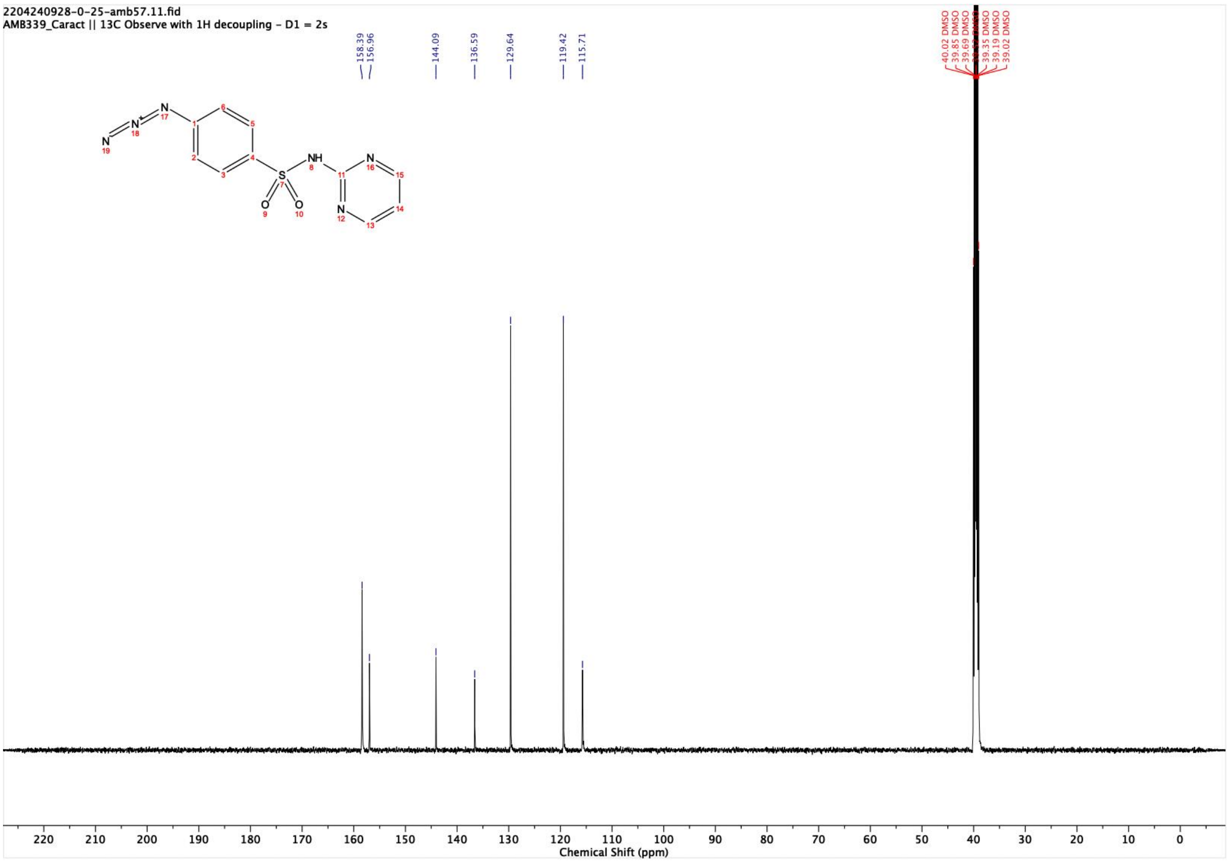
2204240928-0-25-amb57.11.fid
AMB339_Caract || 13C Observe with 1H decoupling - D1 = 2s
158.39
156.96
144.09
136.59
129.64
119.42
115.71
Chemical Shift (ppm)

S4 – ^{1}H

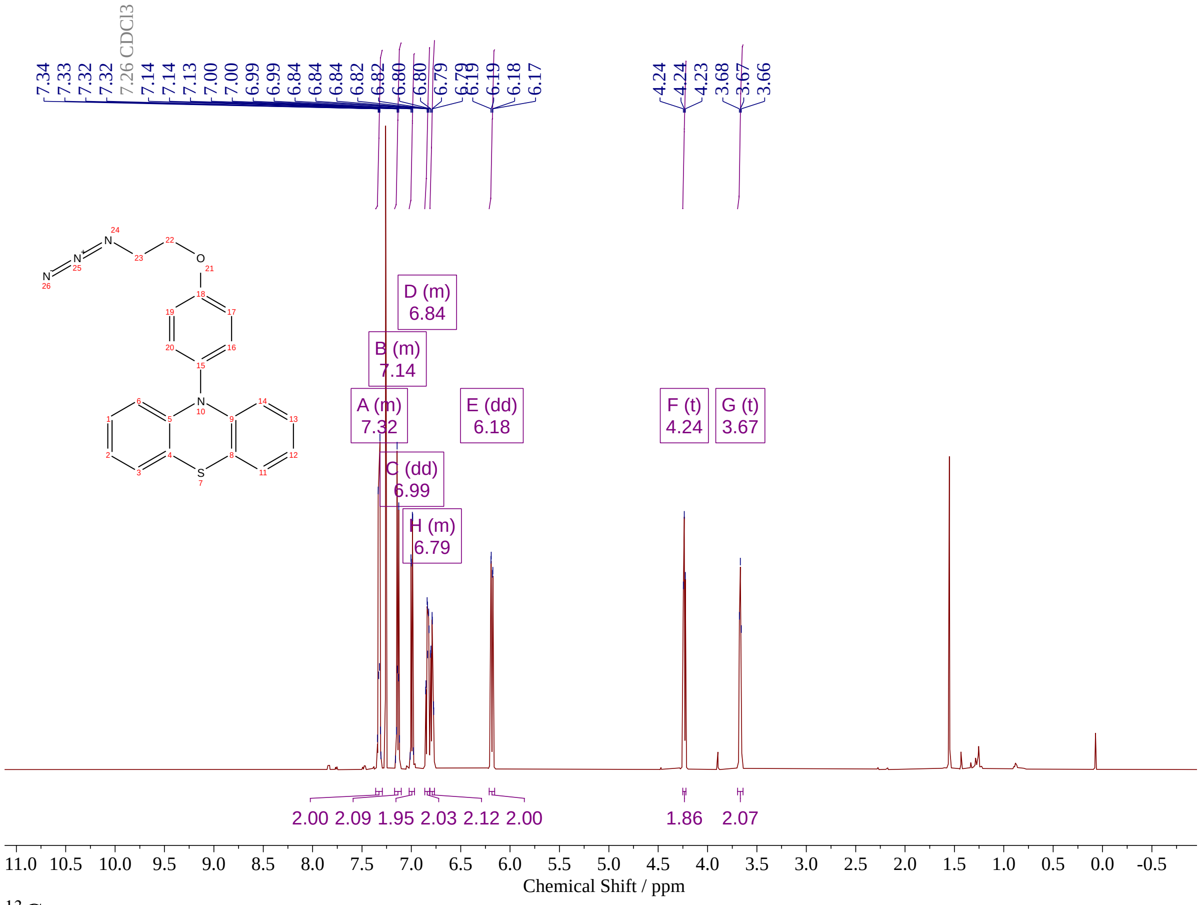

7.34 7.33 7.32 7.32 7.26 CDCl3 7.14 7.14 7.13 7.00 7.00 6.99 6.99 6.84 6.84 6.84 6.82 6.82 6.80 6.80 6.79 6.79 6.19 6.19 6.18 6.17
4.24 4.24 4.23 3.68 3.67 3.66
D (m) 6.84
B (m) 7.14
A (m) 7.32
E (dd) 6.18
F (t) 4.24
G (t) 3.67
C (dd) 6.99
H (m) 6.79
2.00 2.09 1.95 2.03 2.12 2.00
1.86 2.07
11.0 10.5 10.0 9.5 9.0 8.5 8.0 7.5 7.0 6.5 6.0 5.5 5.0 4.5 4.0 3.5 3.0 2.5 2.0 1.5 1.0 0.5 0.0 -0.5
Chemical Shift / ppm

^{13}C

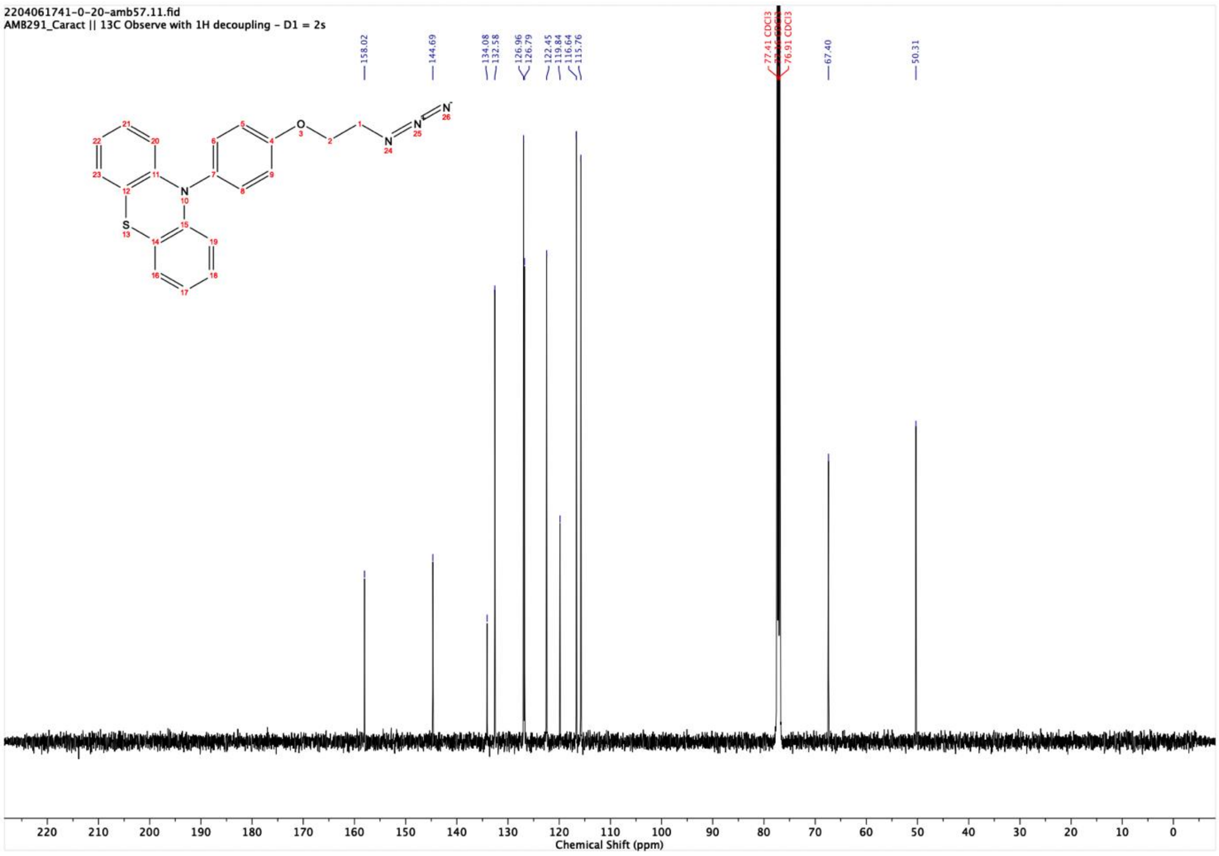

2204061741-0-20-amb57.11.fid
AMB291_Caract || 13C Observe with 1H decoupling - D1 = 2s
158.02
144.69
134.08
132.58
126.96
126.79
122.45
119.84
116.64
115.76
77.41 CDCl3
76.91 CDCl3
67.40
50.31
220 210 200 190 180 170 160 150 140 130 120 110 100 90 80 70 60 50 40 30 20 10 0
Chemical Shift (ppm)

S5 – ^{1}H

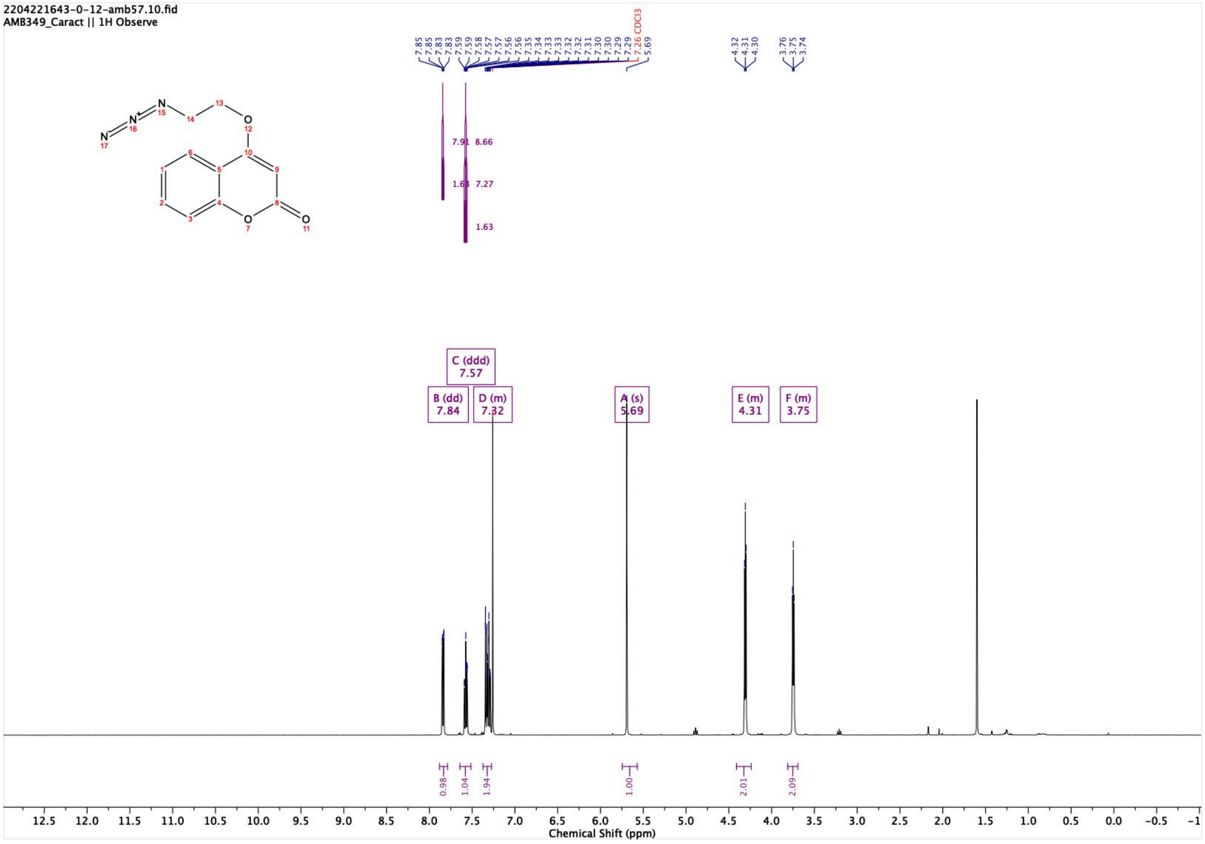

2204221643-0-12-amb57.10.fid
AMB349_Caract || 1H Observe
7.26 CDCl3
C (ddd) 7.57
B (dd) 7.84
D (m) 7.32
A (s) 5.69
E (m) 4.31
F (m) 3.75
Chemical Shift (ppm)

^{13}C

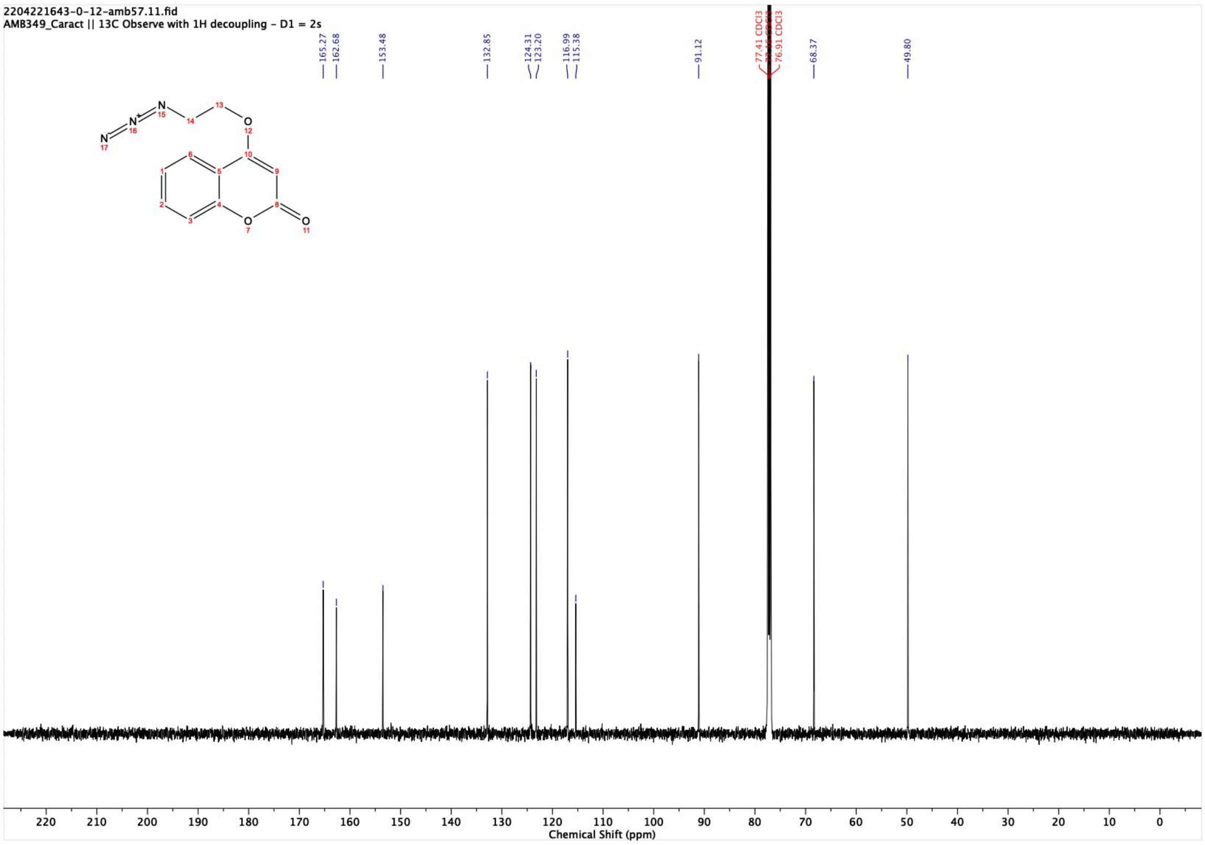

2204221643-0-12-amb57.11.fid
AMB349_Caract || 13C Observe with 1H decoupling - D1 = 2s
165.27
162.68
153.48
132.85
124.31
123.20
116.99
115.38
91.12
77.41 CDCl3
76.91 CDCl3
68.37
49.80
Chemical Shift (ppm)

S6 – ^{1}H

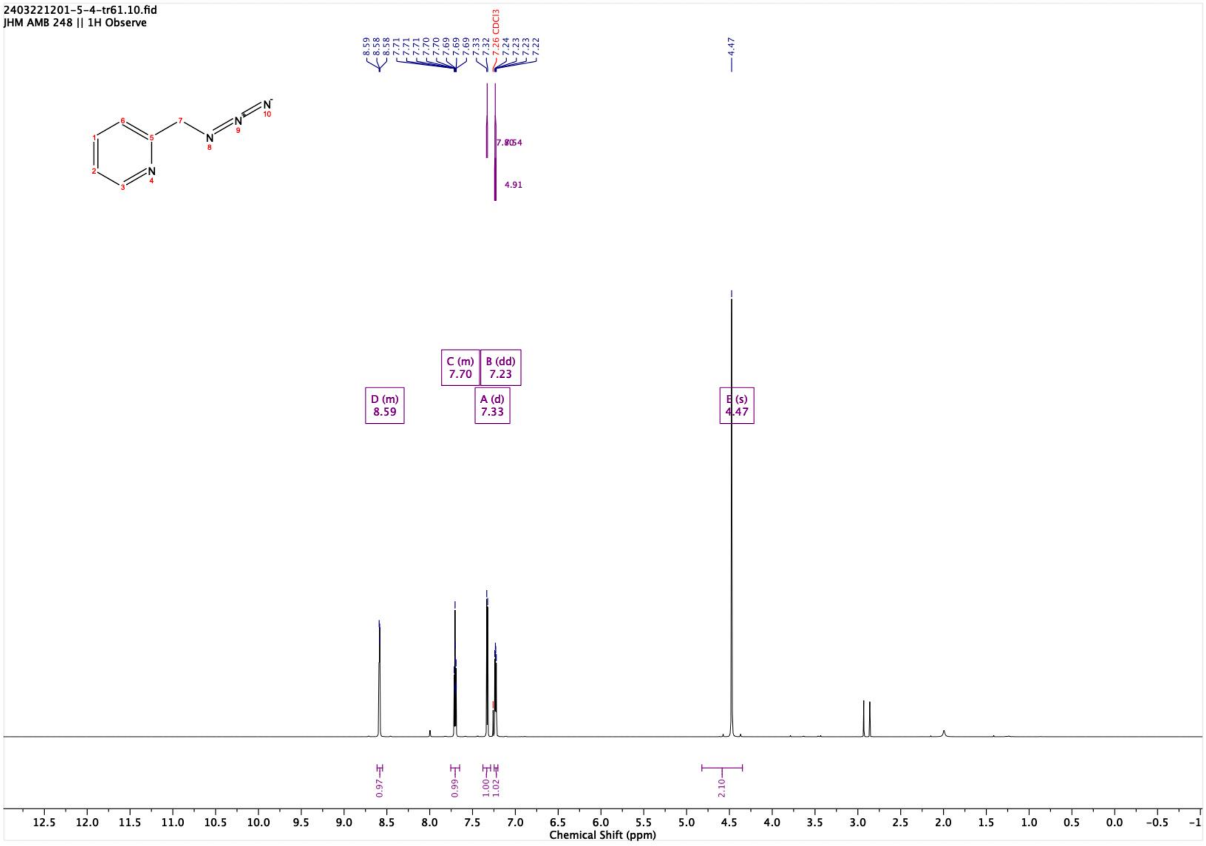

^{13}C DEPTQ

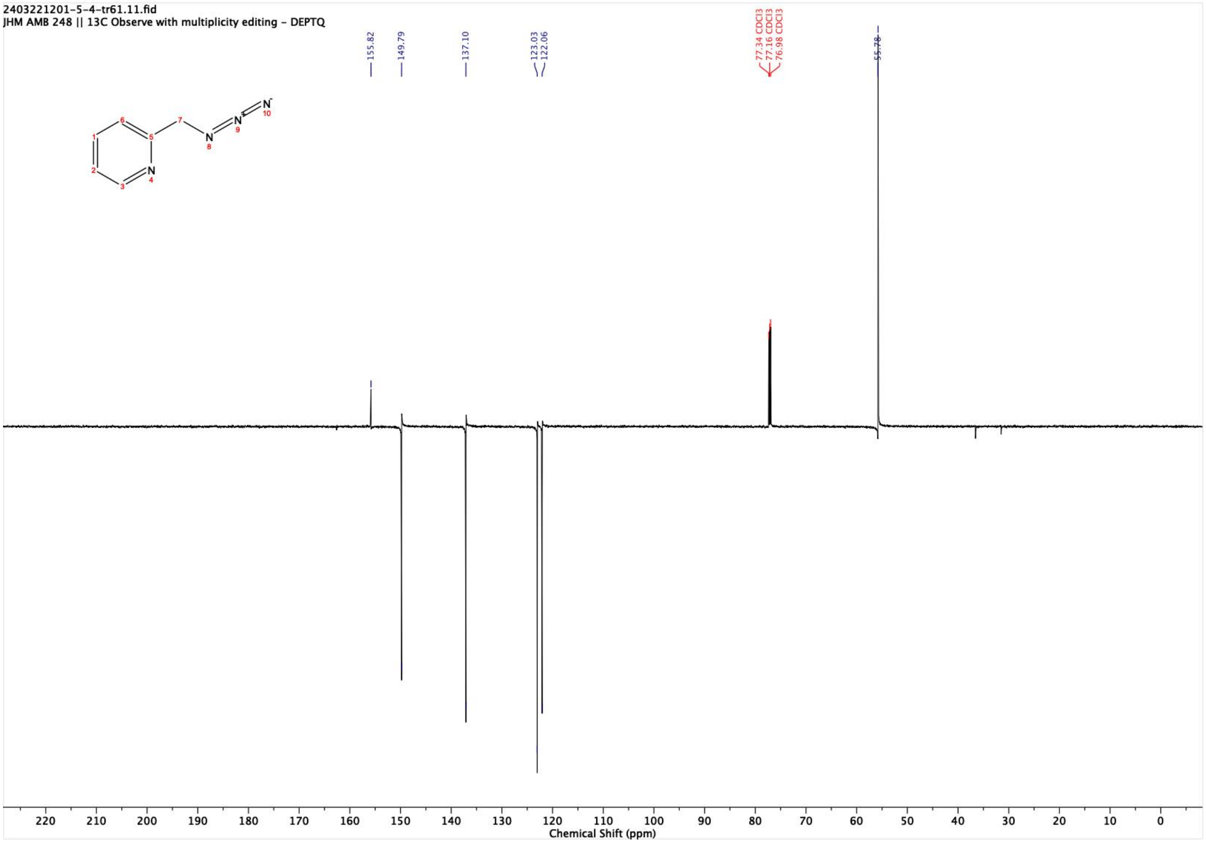

S7 – ^{1}H

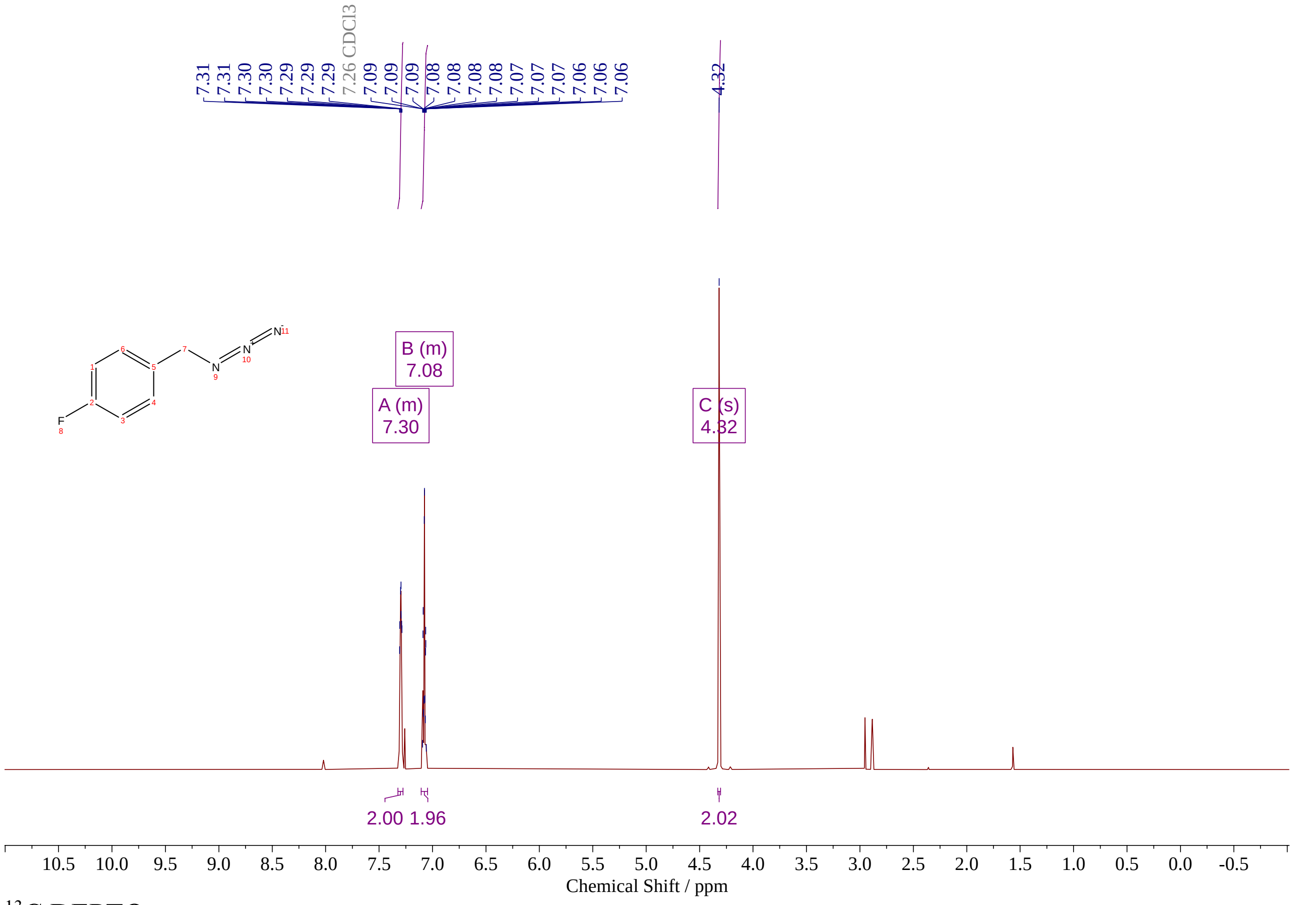

^{13}C DEPTQ

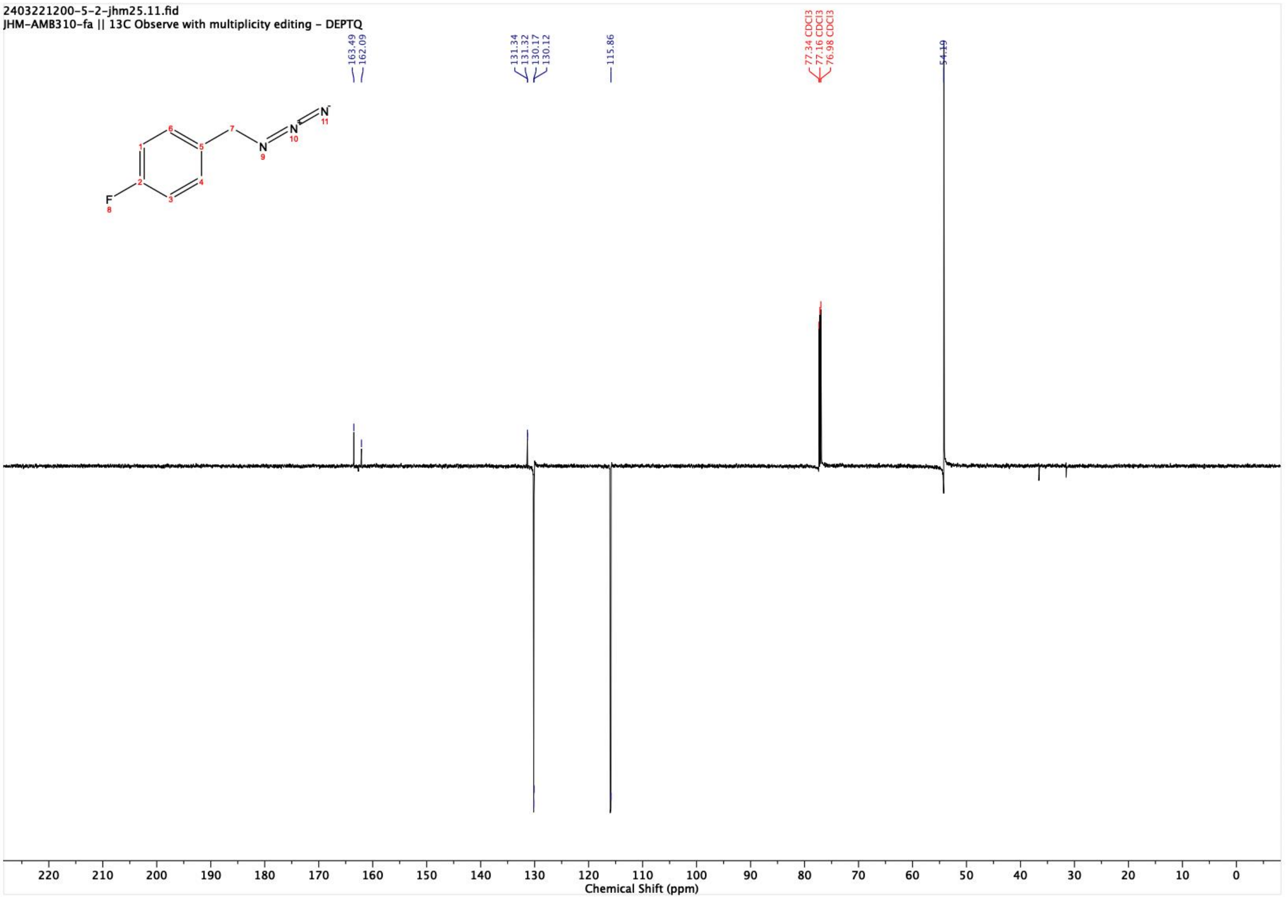

^{19}F

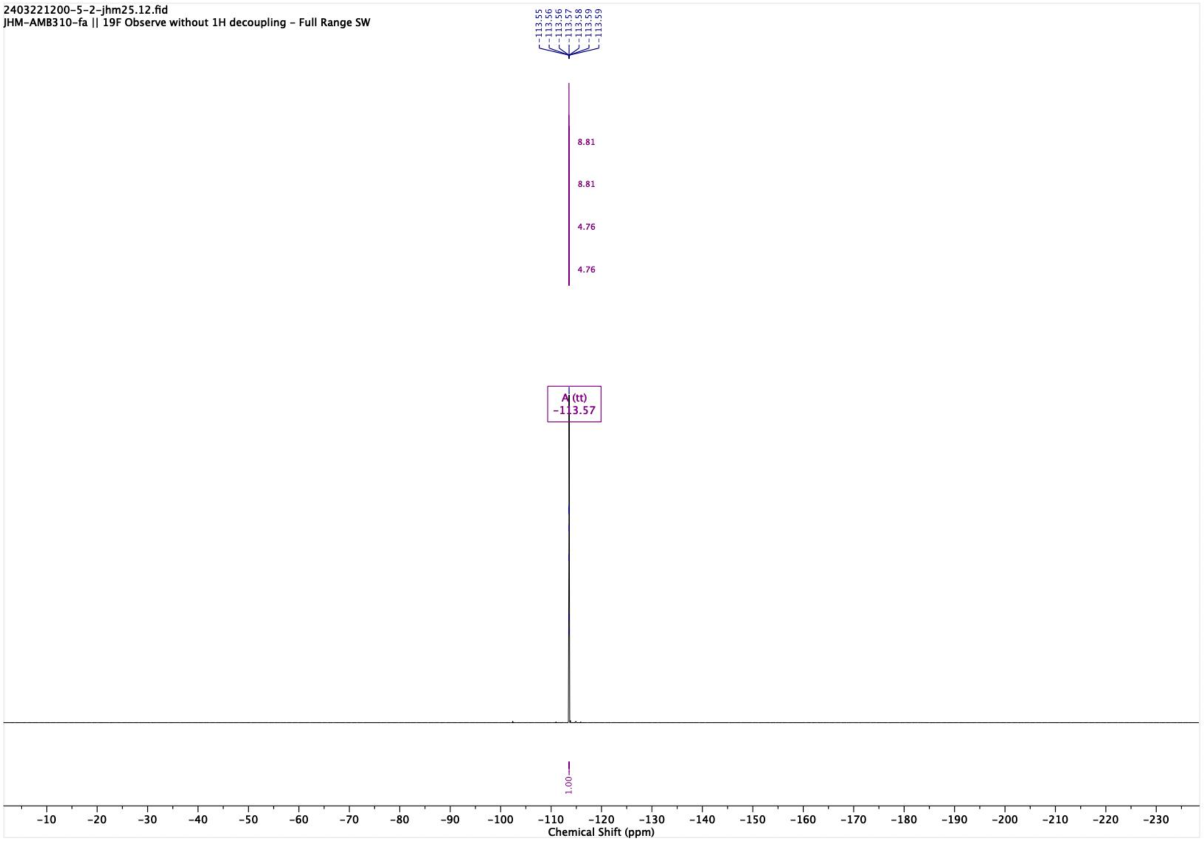
2403221200-5-2-jhm25.12.fid
JHM-AMB310-fa || 19F Observe without 1H decoupling - Full Range SW
-113.55
-113.56
-113.56
-113.57
-113.58
-113.59
-113.59
8.81
8.81
4.76
4.76
A (tt)
-113.57
1.00
-10
-20
-30
-40
-50
-60
-70
-80
-90
-100
-110
-120
-130
-140
-150
-160
-170
-180
-190
-200
-210
-220
-230
Chemical Shift (ppm)

S8 – ^{1}H

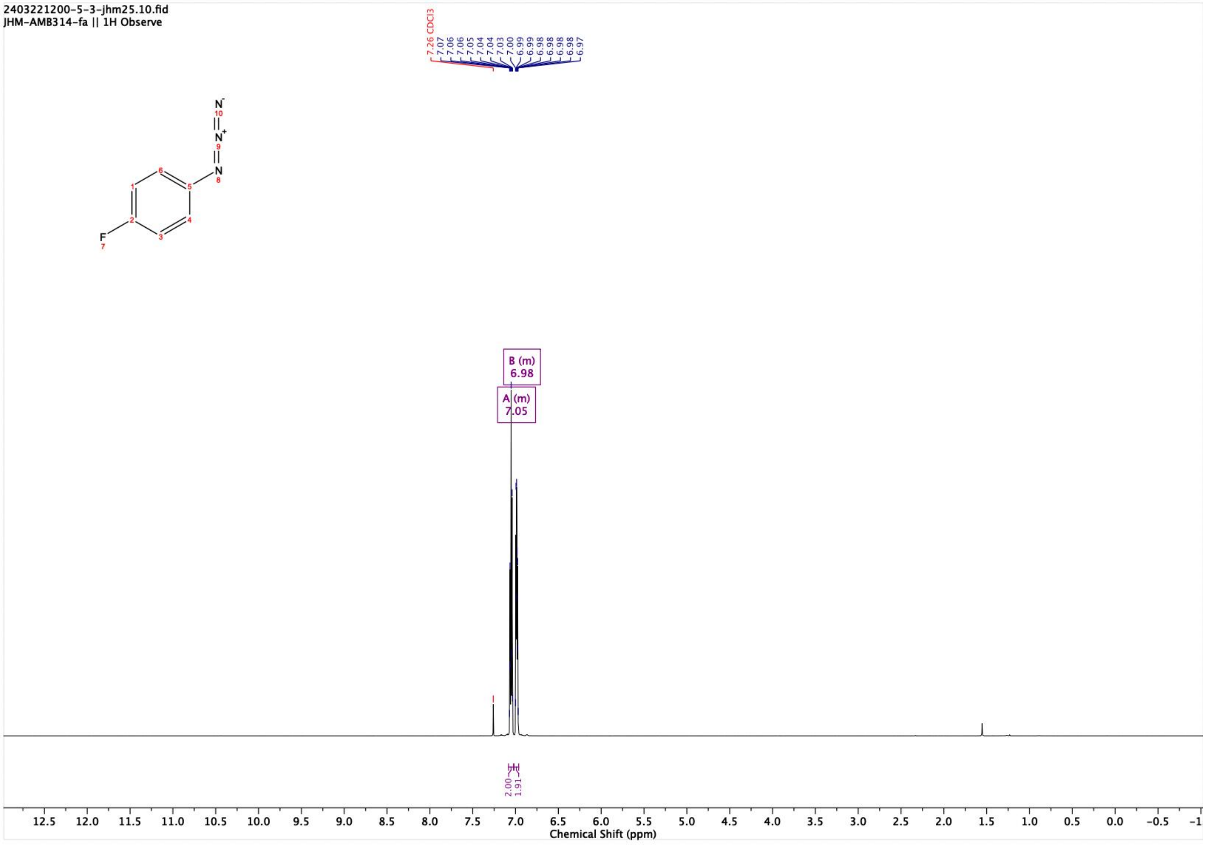

^{13}C DEPTQ

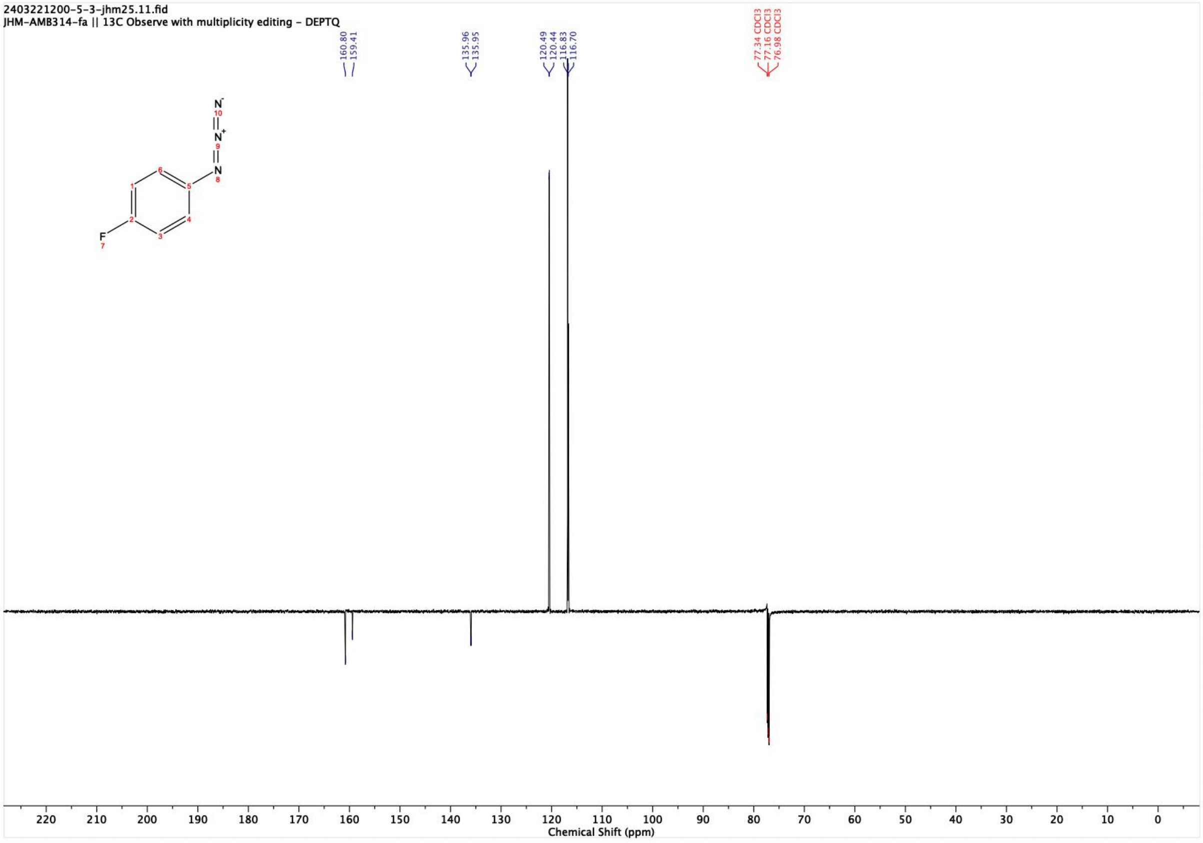

^{19}F

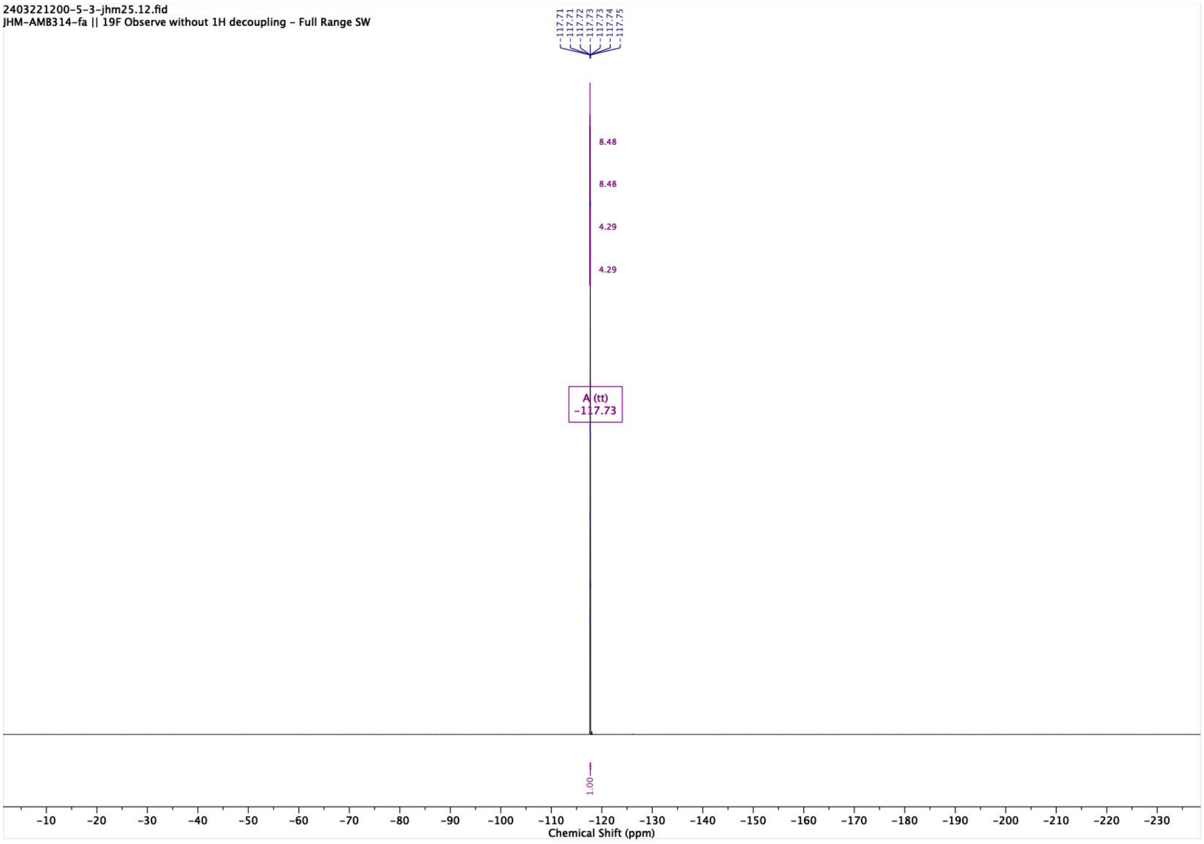

2403221200-5-3-jhm25.12.fid
JHM-AMB314-fa || 19F Observe without 1H decoupling - Full Range SW
-117.71
-117.71
-117.72
-117.73
-117.73
-117.74
-117.75
8.48
8.48
4.29
4.29
A (tt)
-117.73
1.00
-10
-20
-30
-40
-50
-60
-70
-80
-90
-100
-110
-120
-130
-140
-150
-160
-170
-180
-190
-200
-210
-220
-230
Chemical Shift (ppm)

S9 – ^{1}H

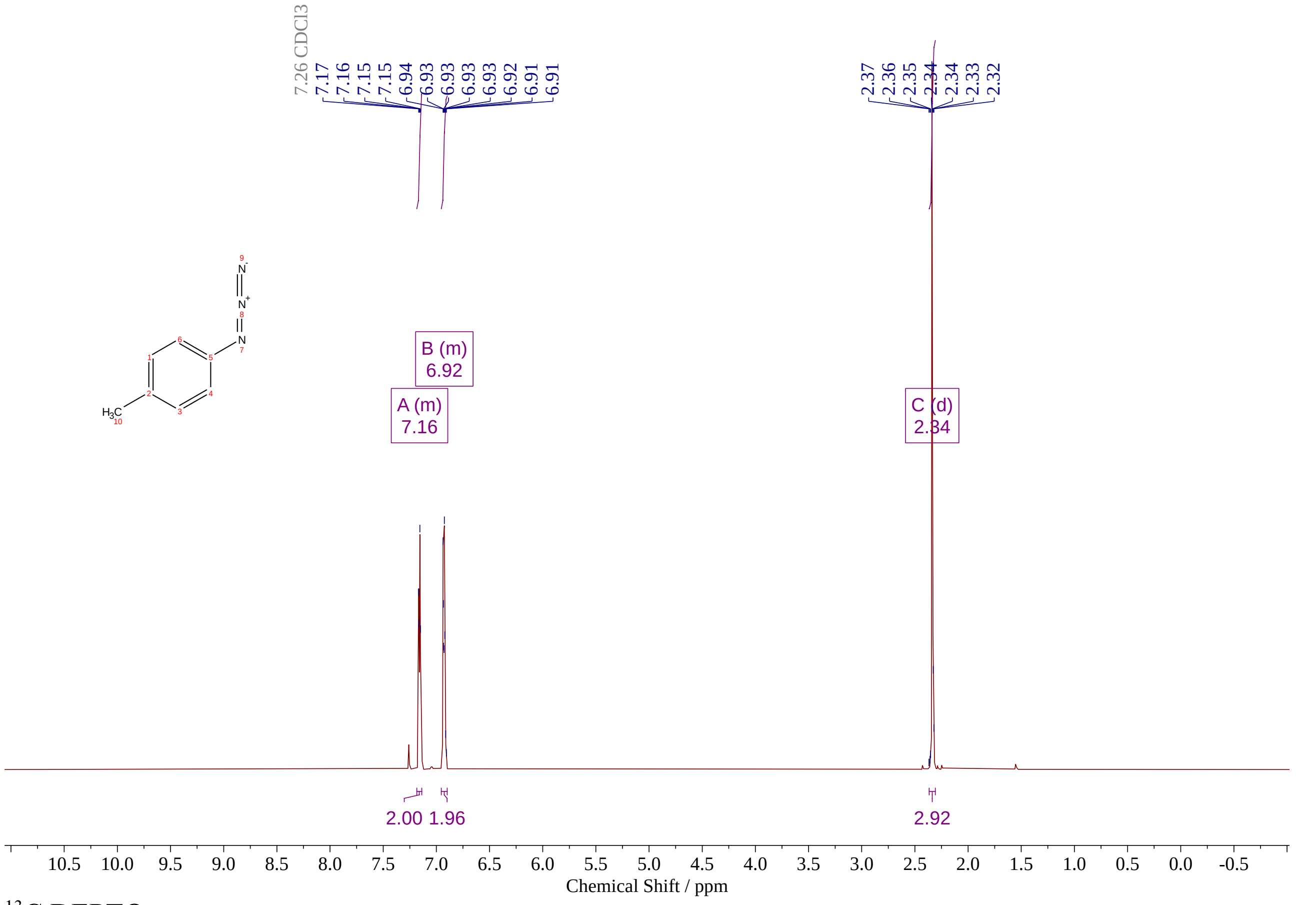

^{13}C DEPTQ

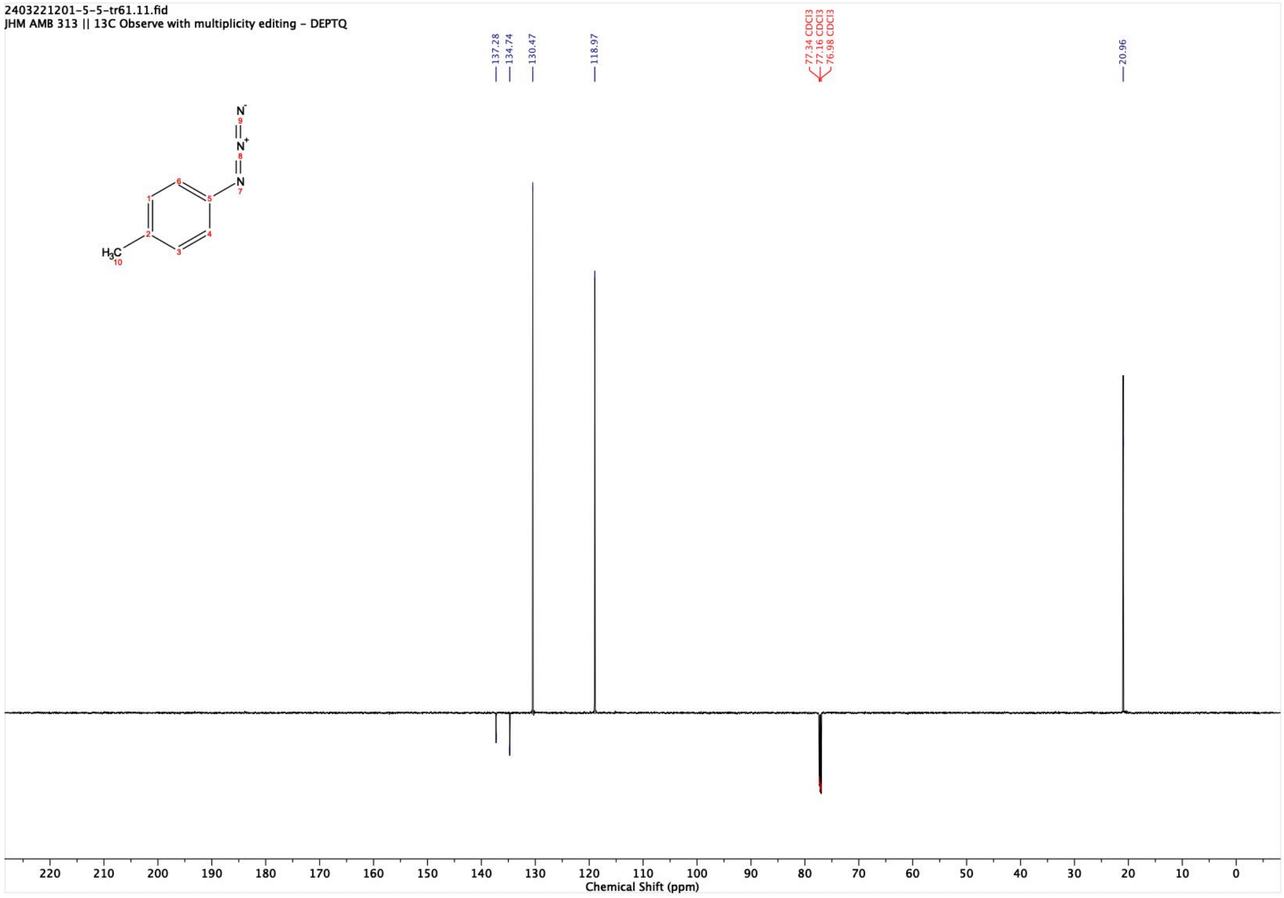

S10 – ^{1}H

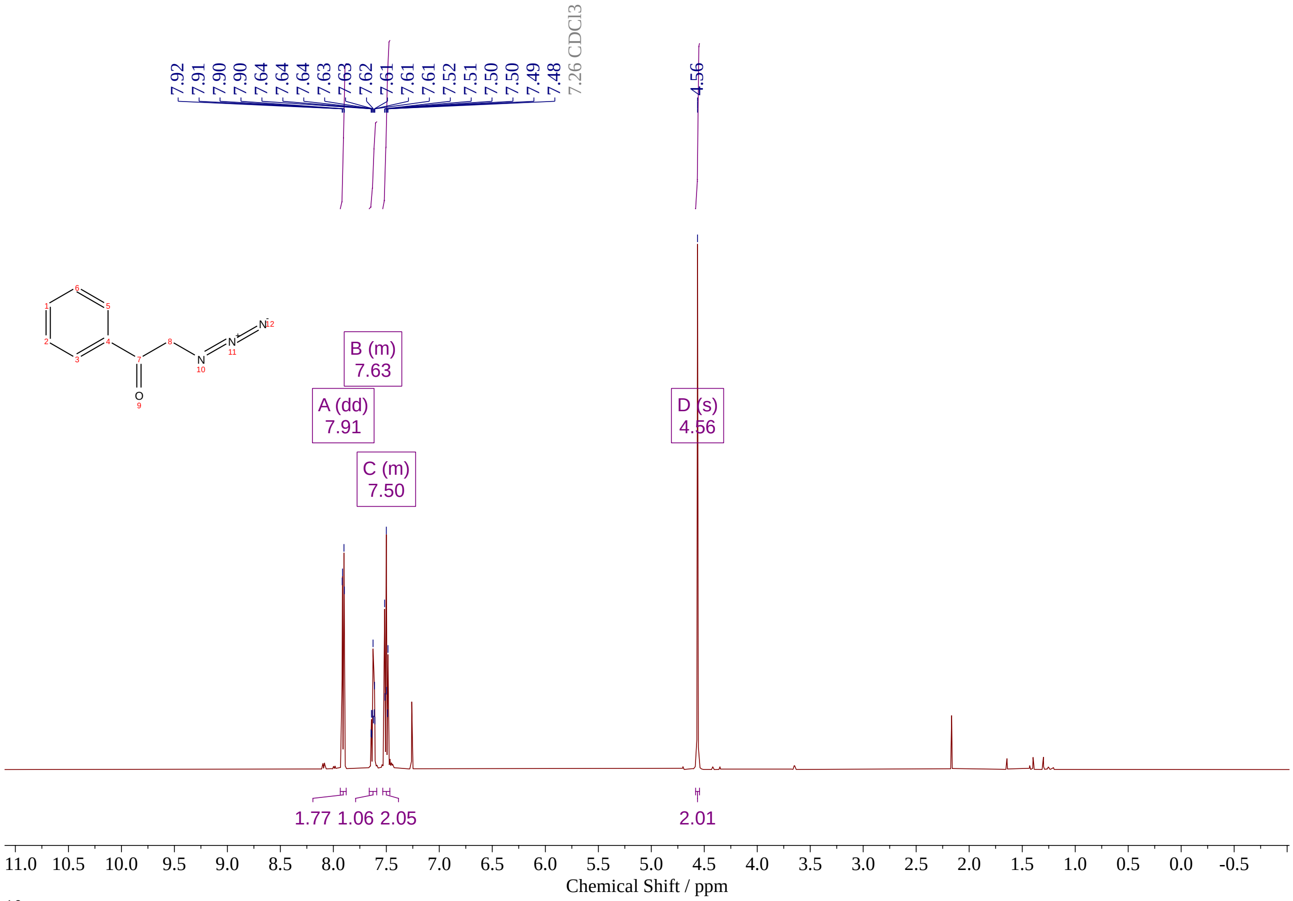

^{13}C DEPTQ

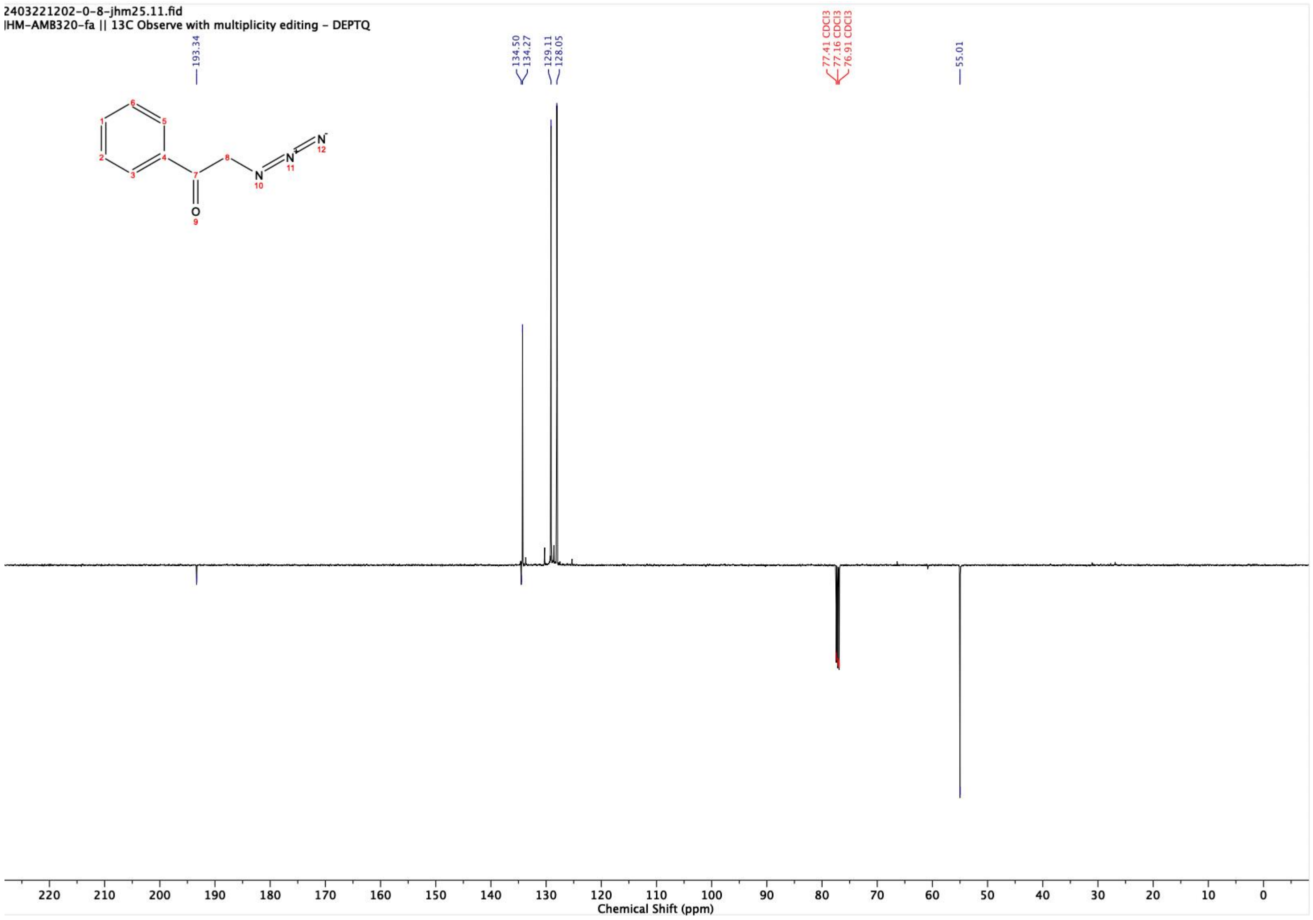

S11 – ^{1}H

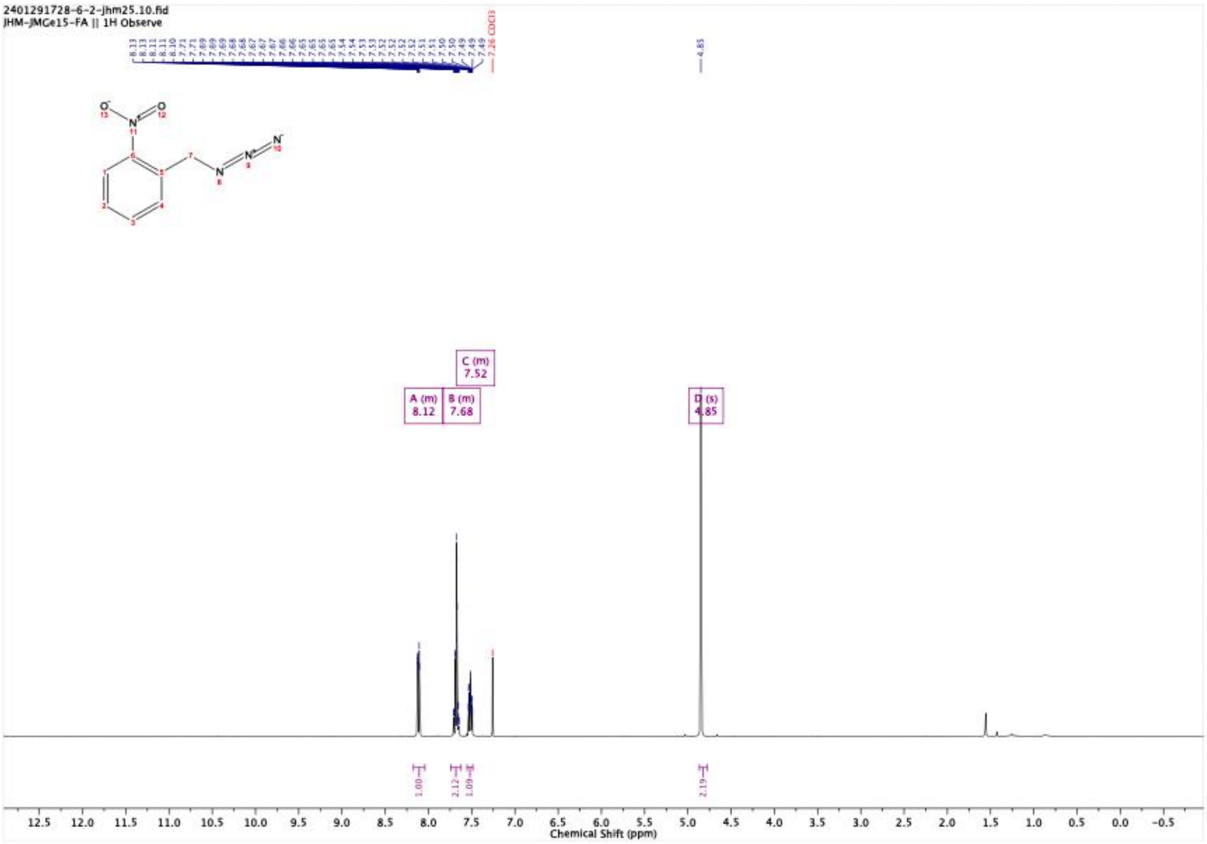

^{13}C DEPTQ

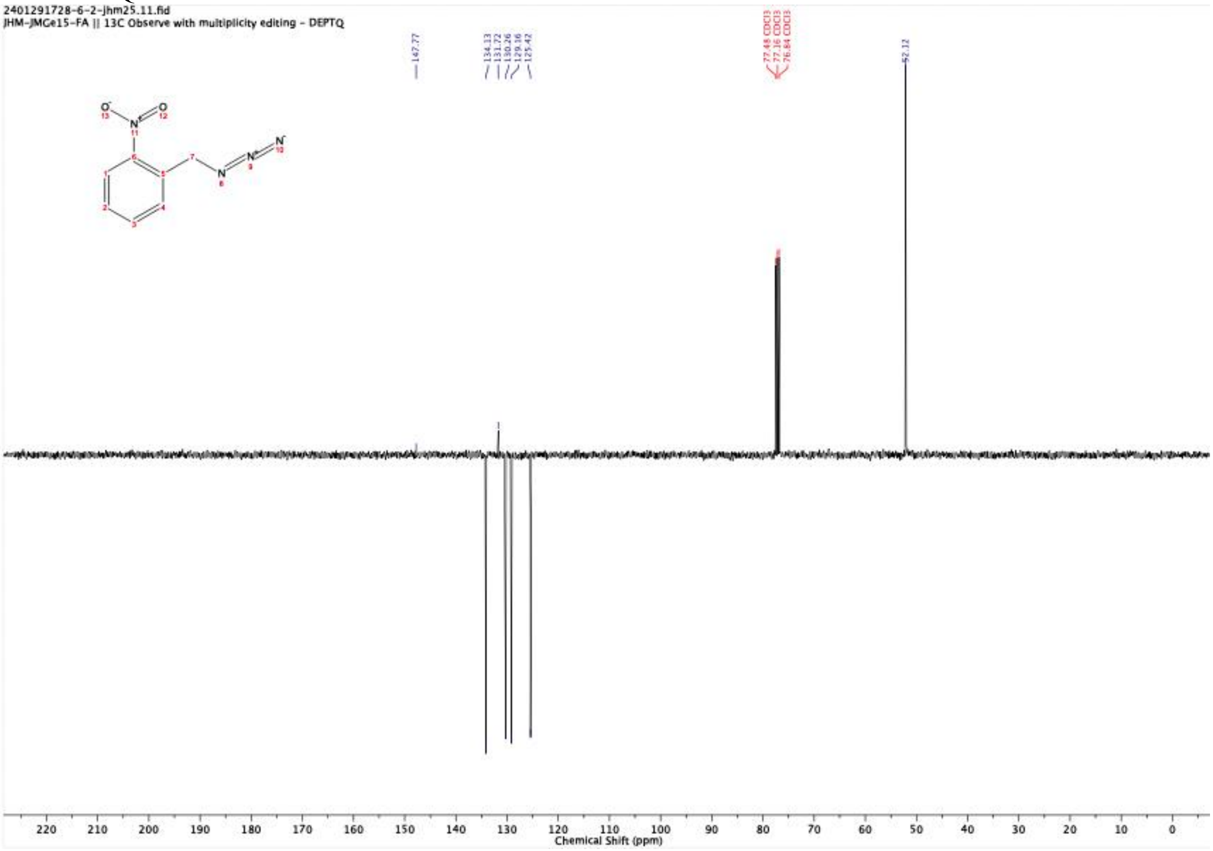

S12 – ^{1}H

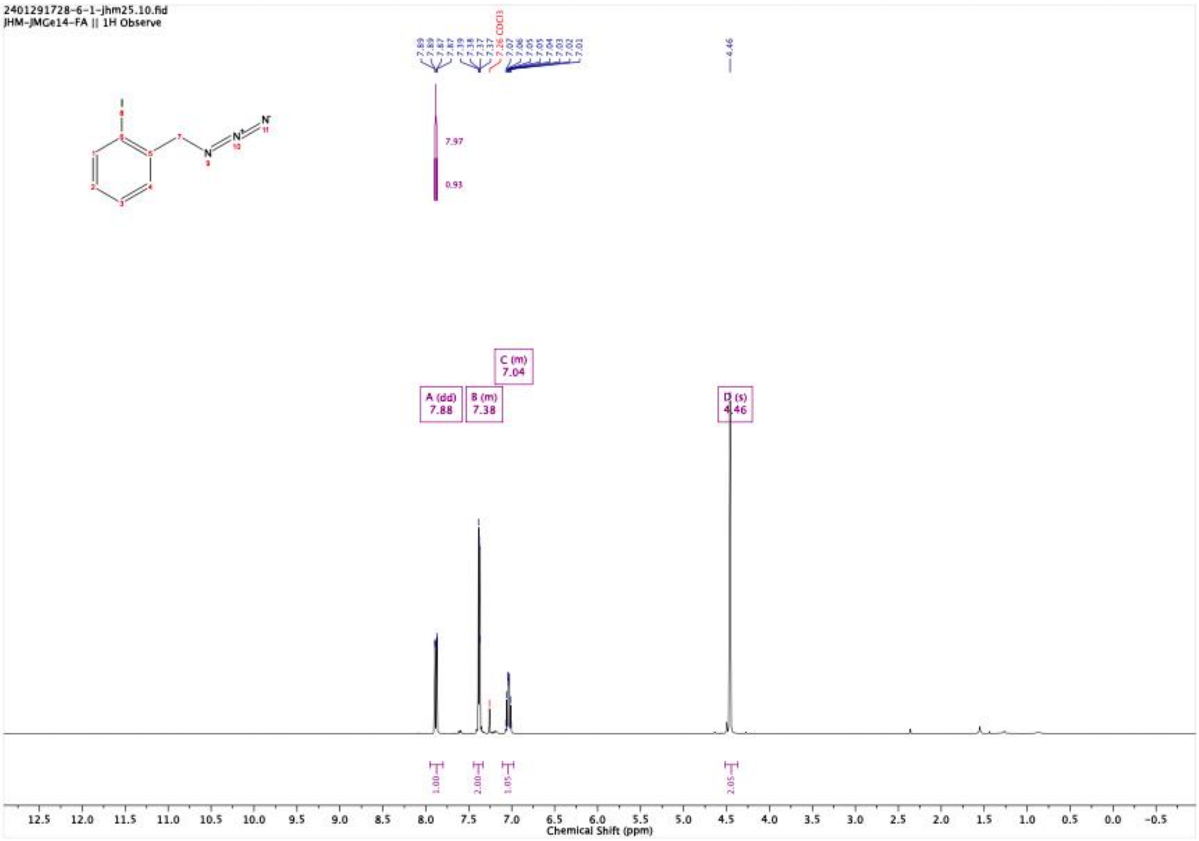

^{13}C DEPTQ

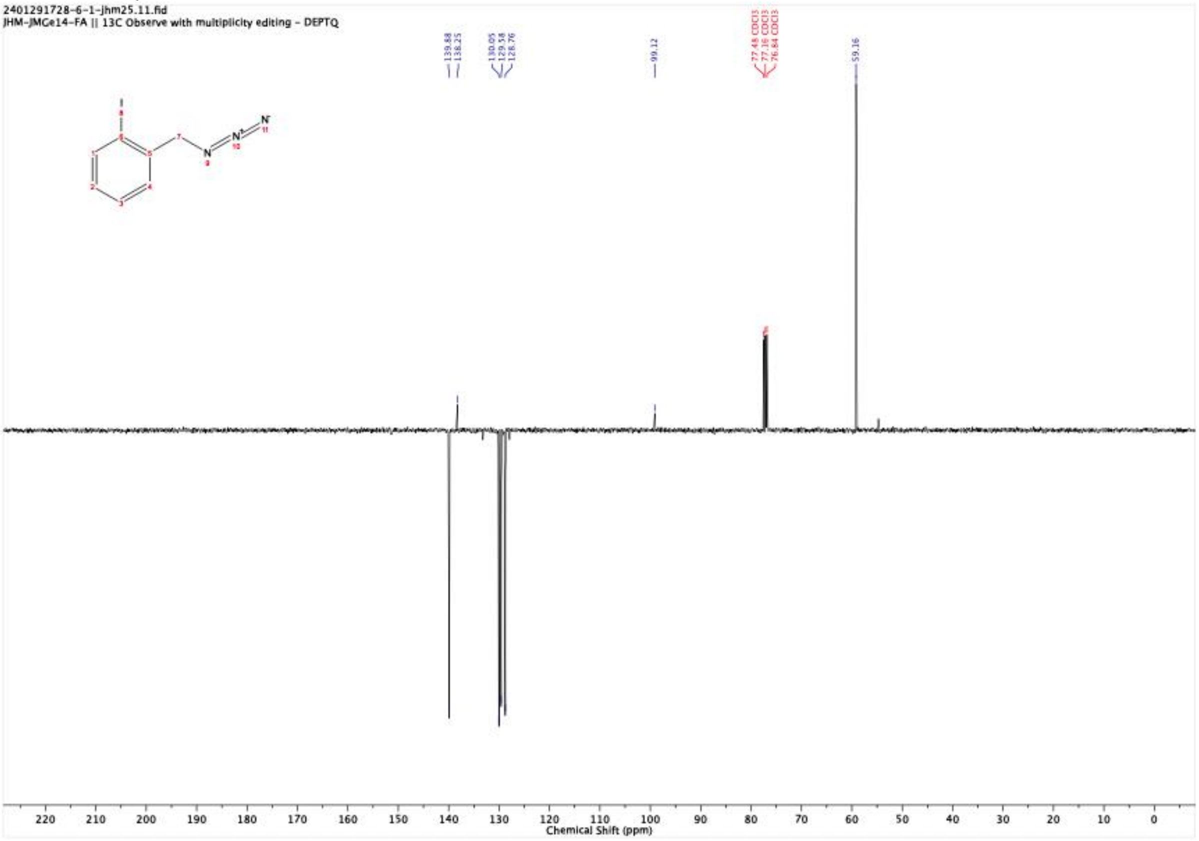

S13 – ^{1}H

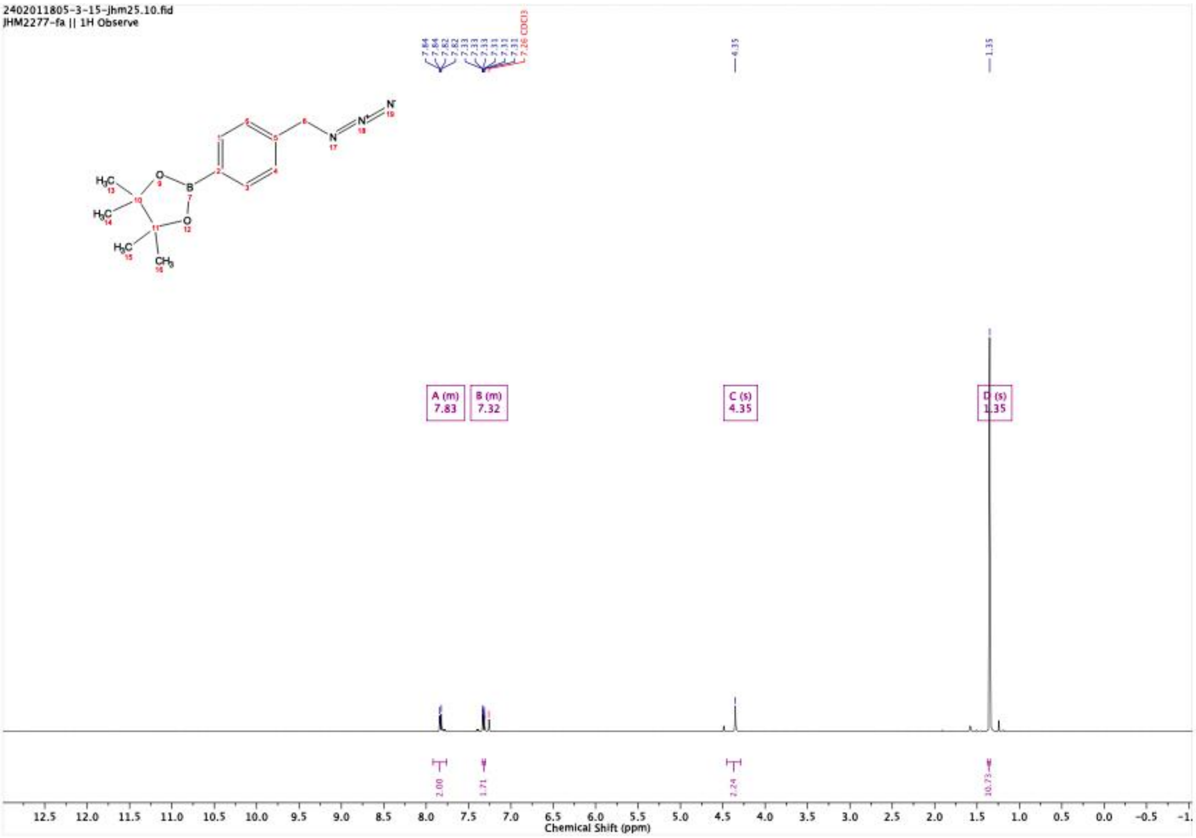

^{13}C DEPTQ

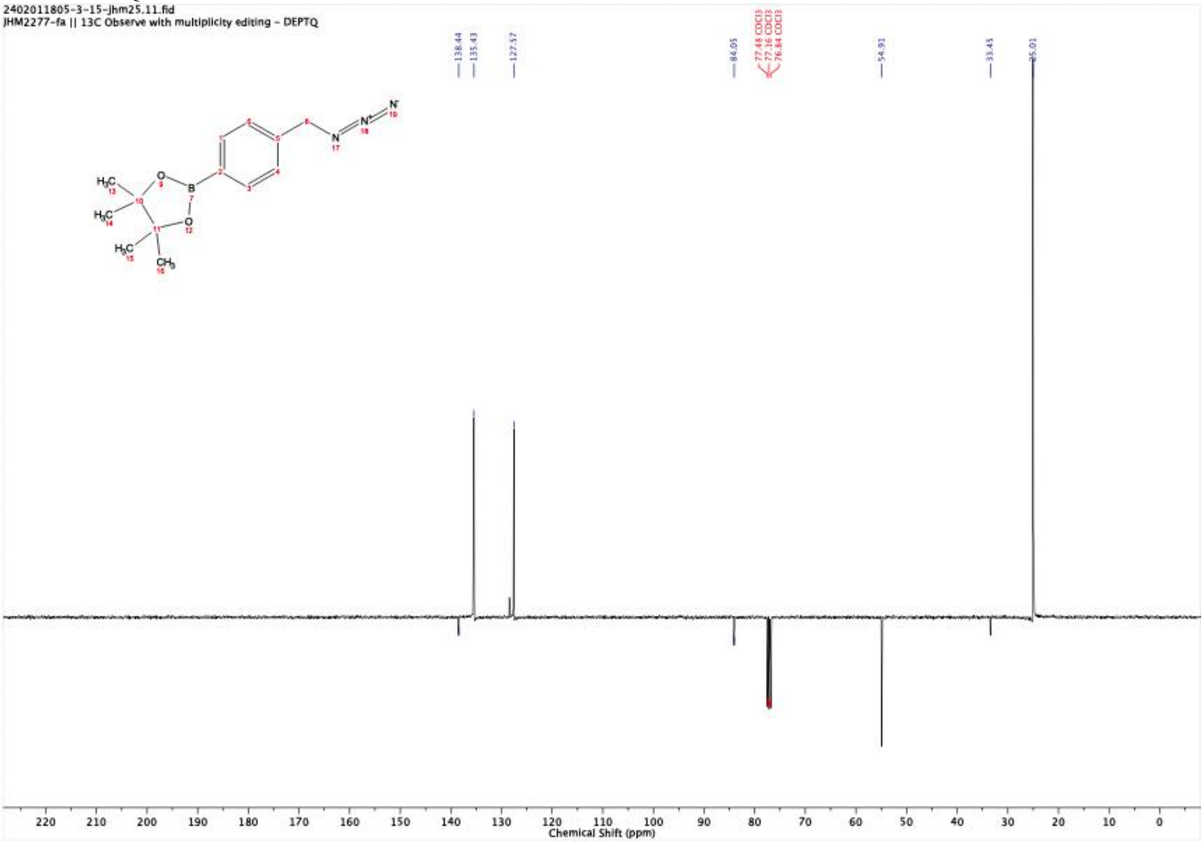

^{11}B

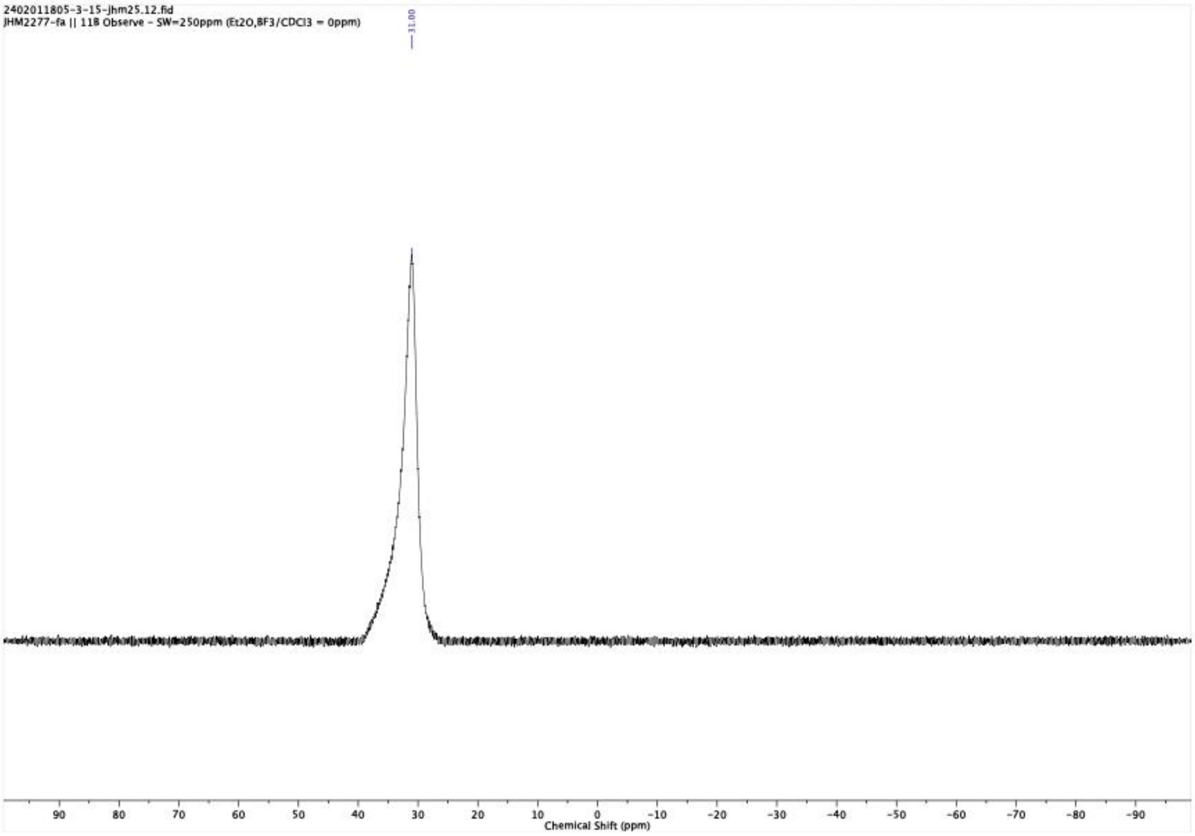

2402011805-3-15-jhm25.12.fid
JHM2277-fa || 11B Observe - SW=250ppm (Et2O,BF3/CDCl3 = 0ppm)
31.00
90
80
70
60
50
40
30
20
10
0
-10
-20
-30
-40
-50
-60
-70
-80
-90
Chemical Shift (ppm)

S14 – ^{1}H

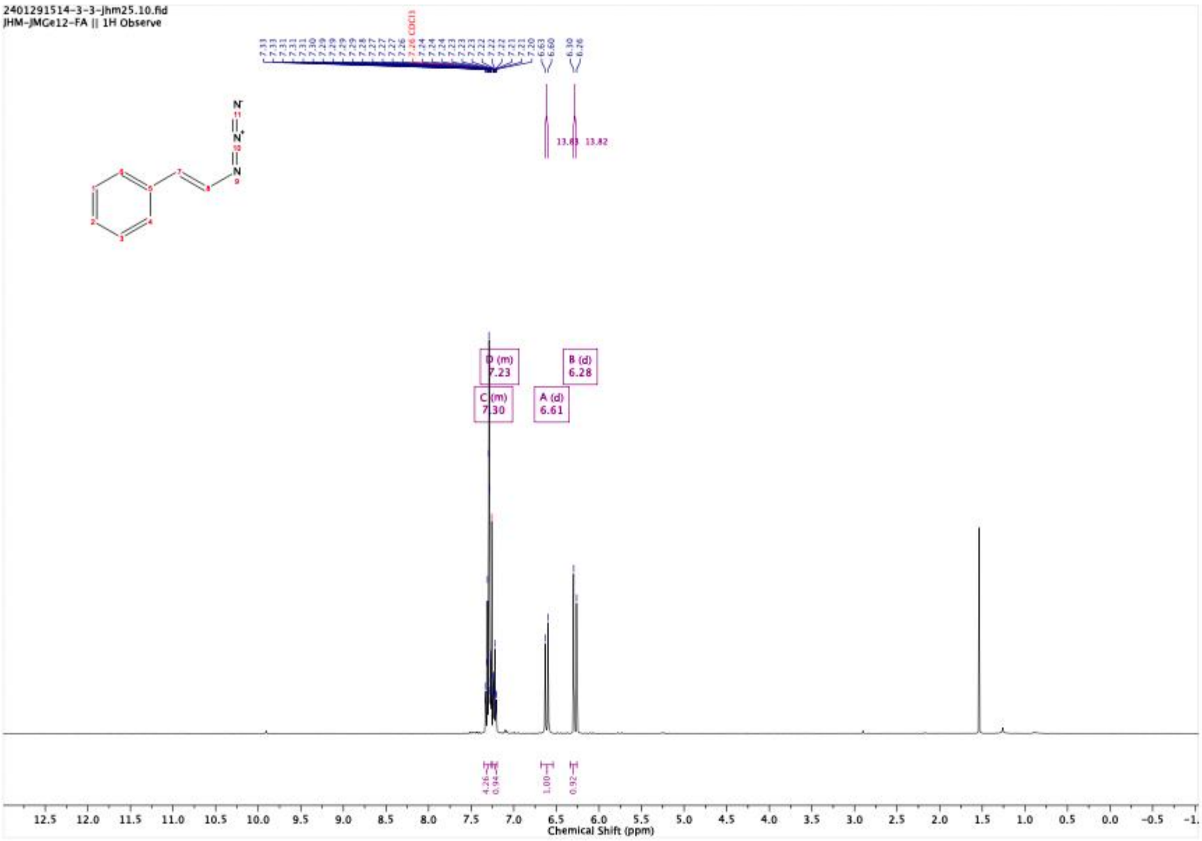

^{13}C DEPTQ

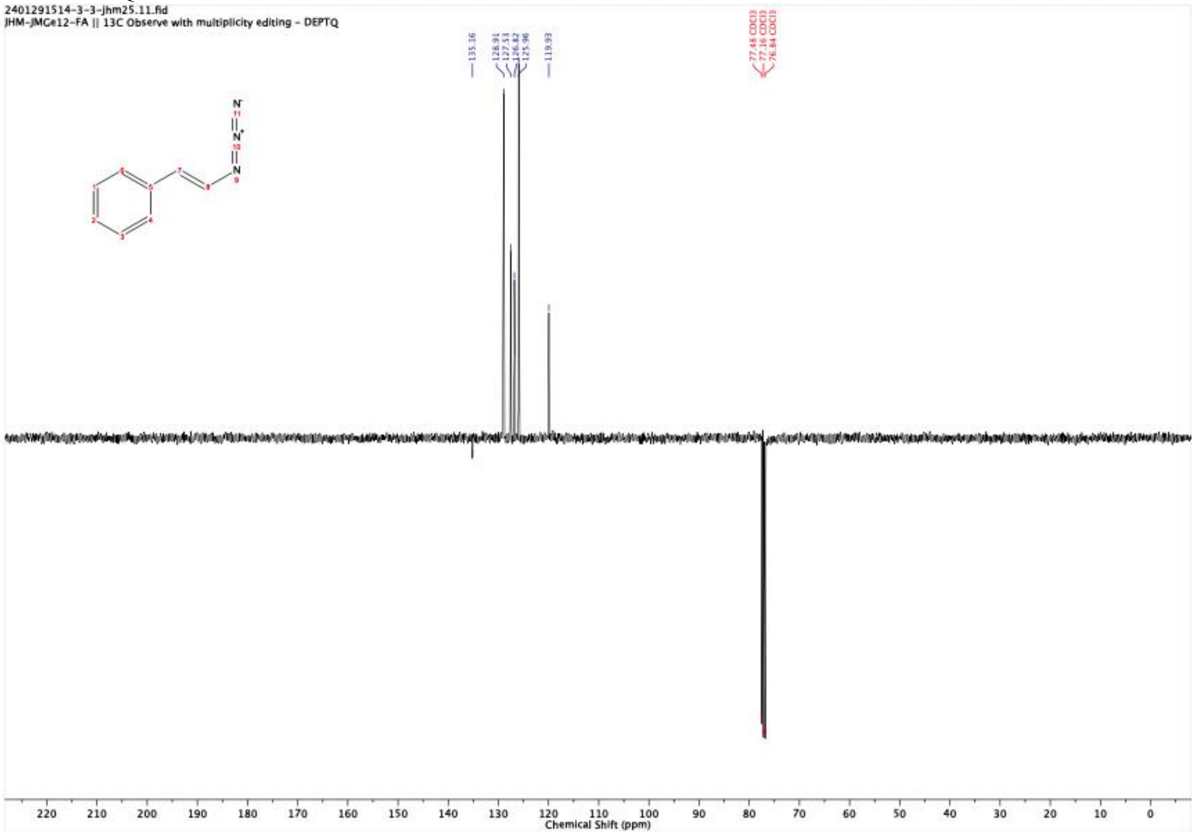

S15 – ^{1}H

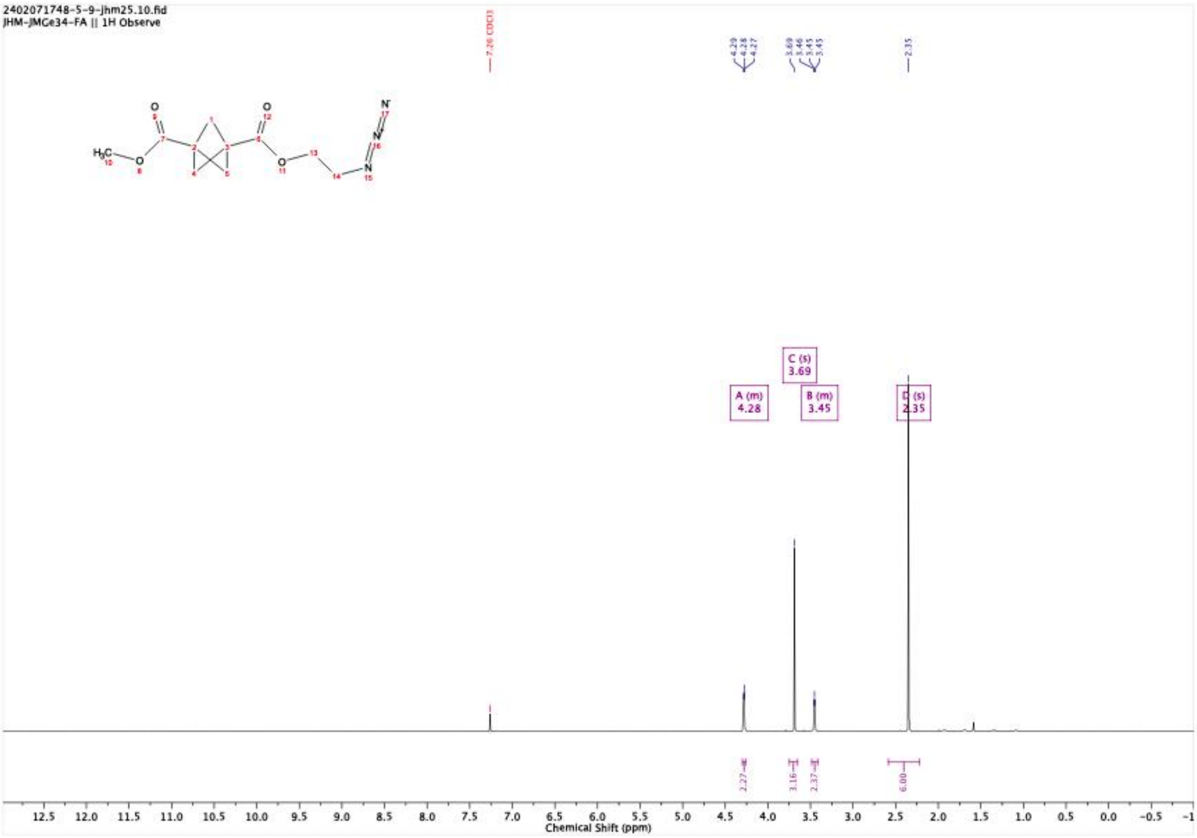

^{13}C DEPTQ

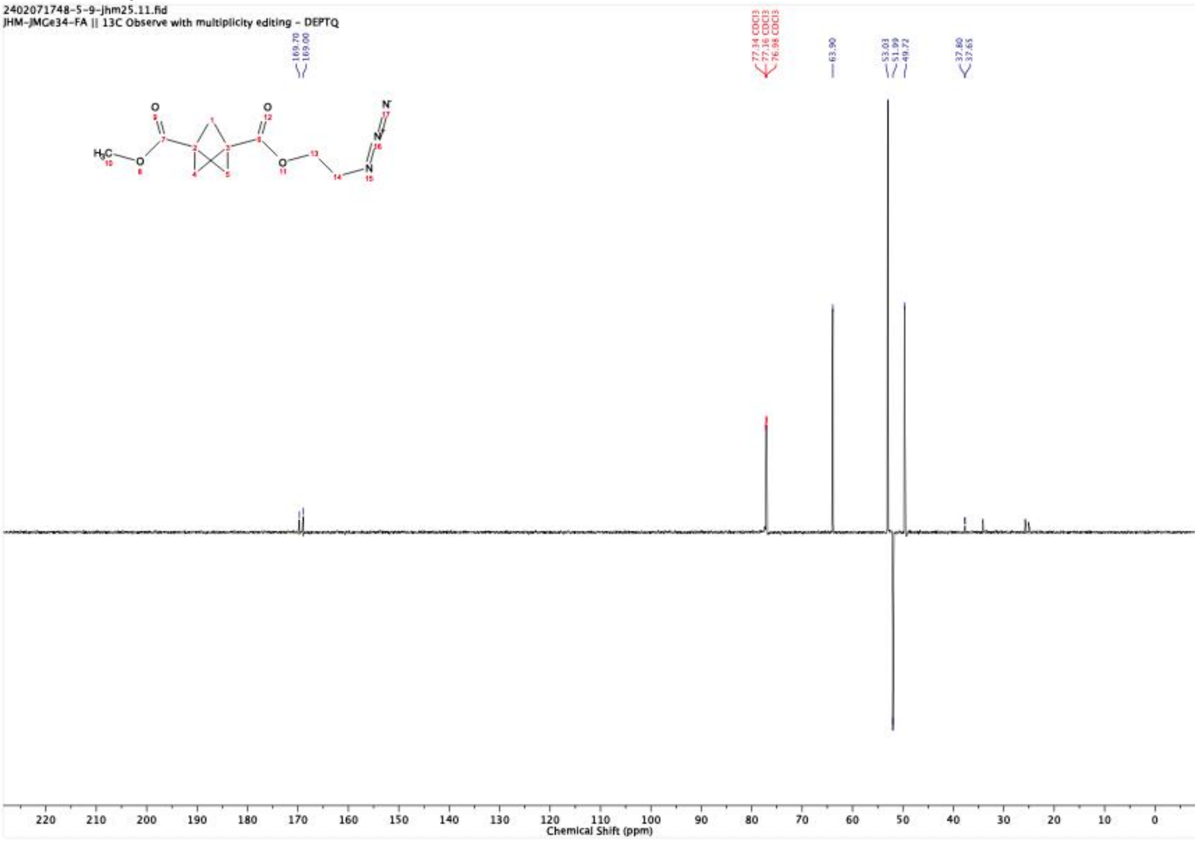

S16 – ^{1}H

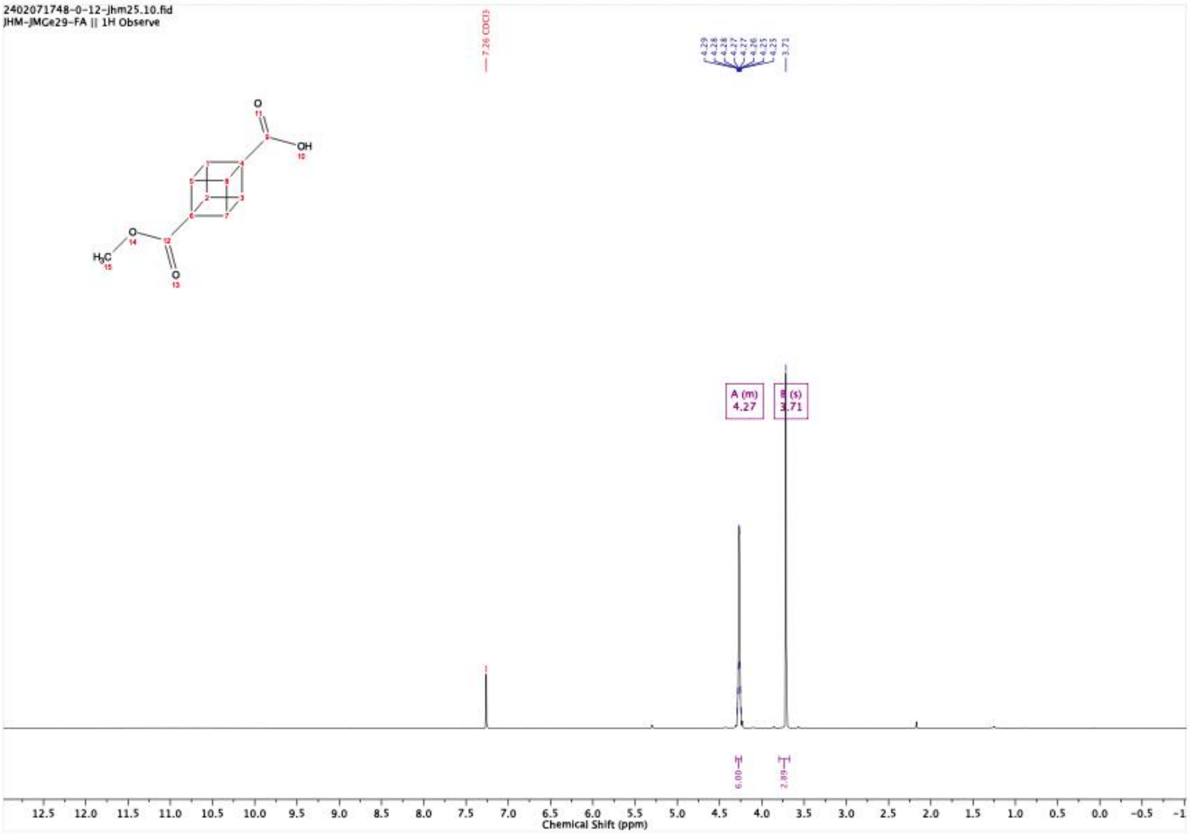

^{13}C DEPTQ

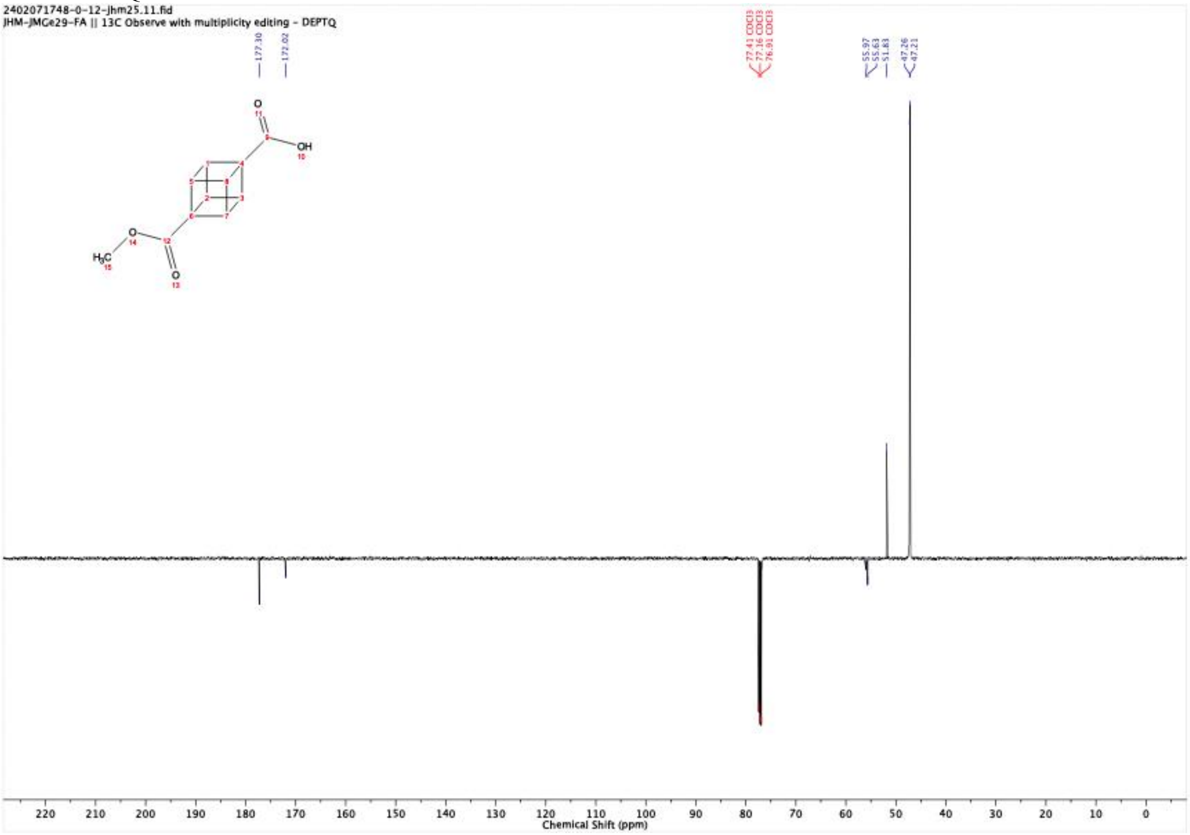

S17 – ^{1}H

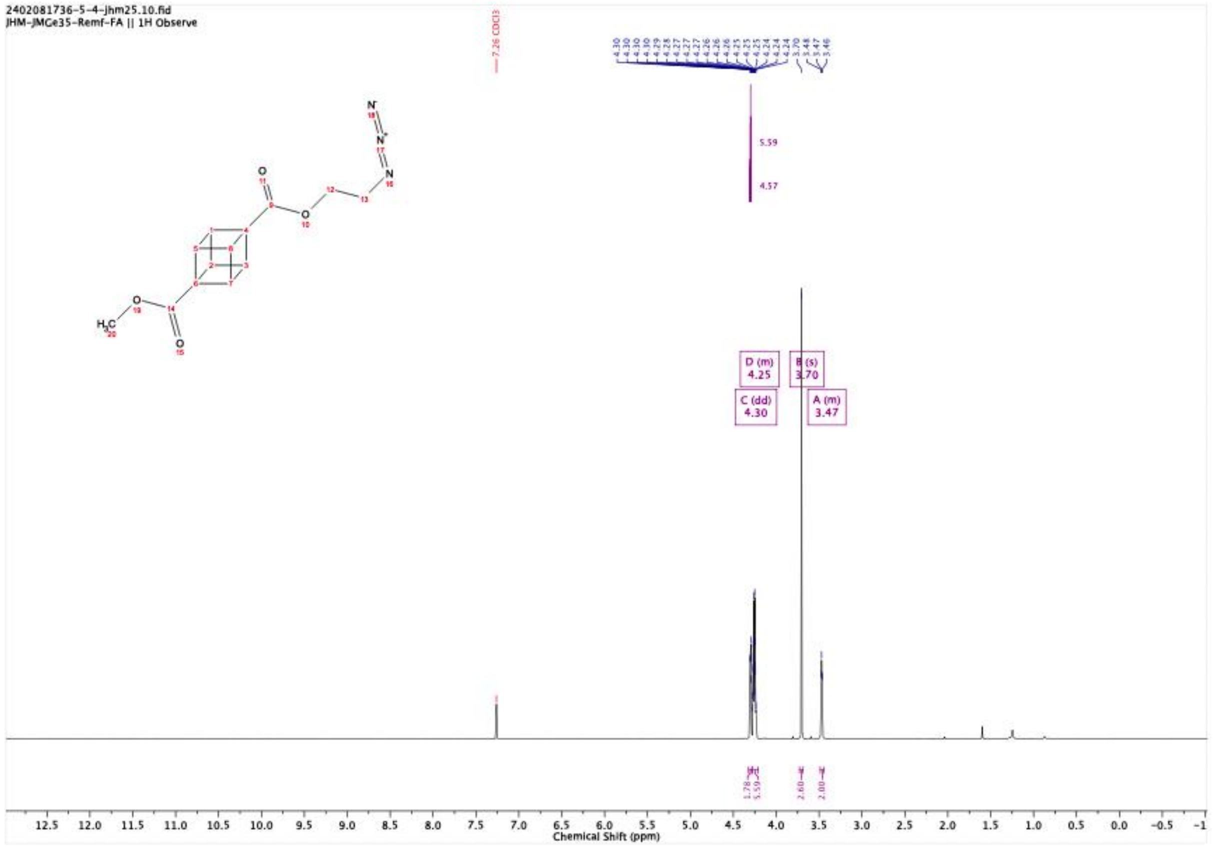

^{13}C DEPTQ

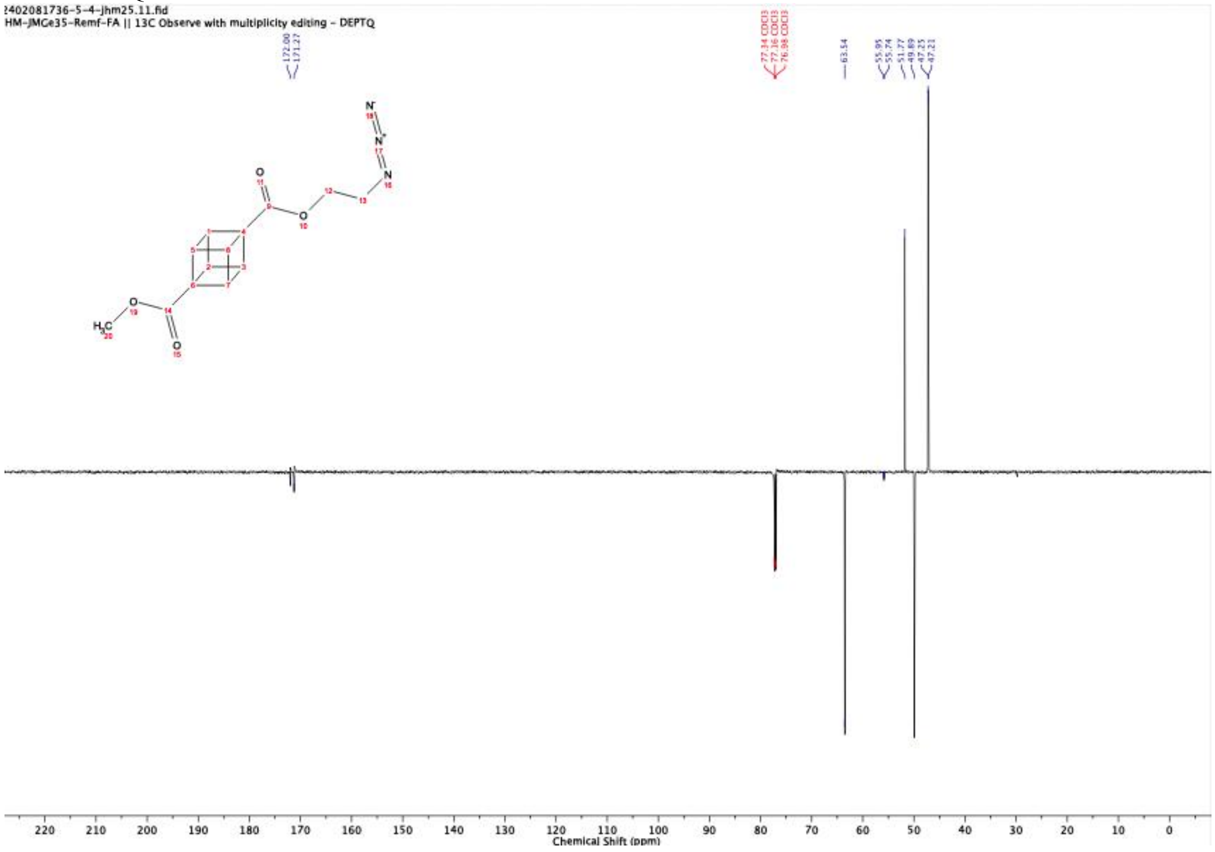

S18 – ¹H

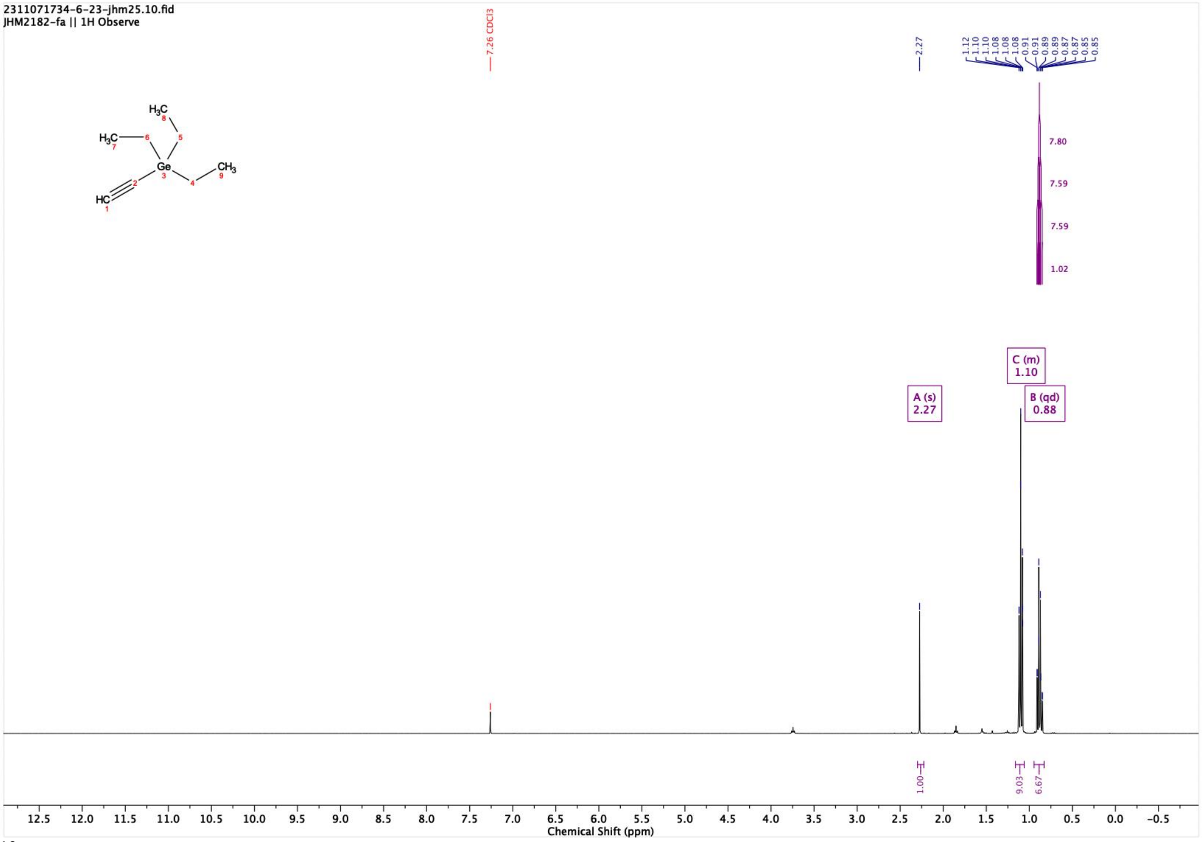

^{13}C DEPTQ

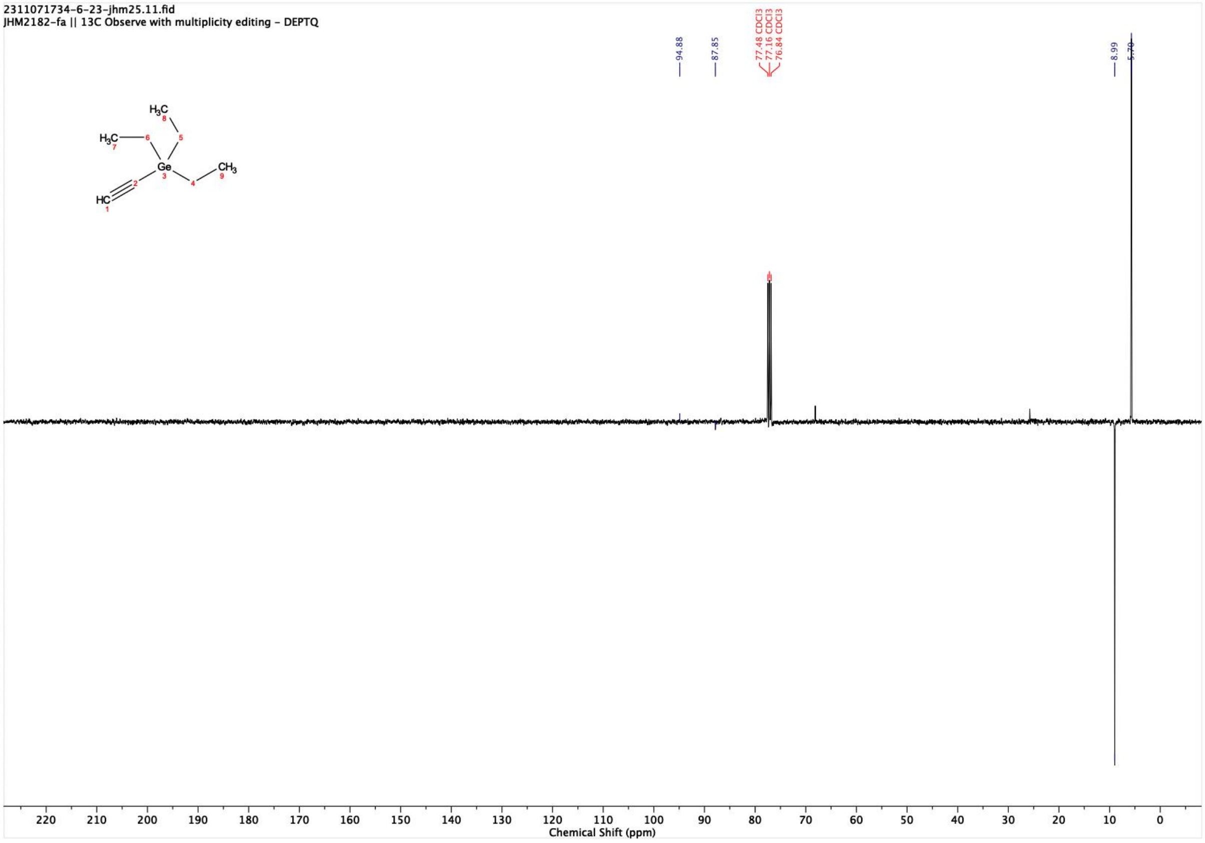

S19 – ^{1}H

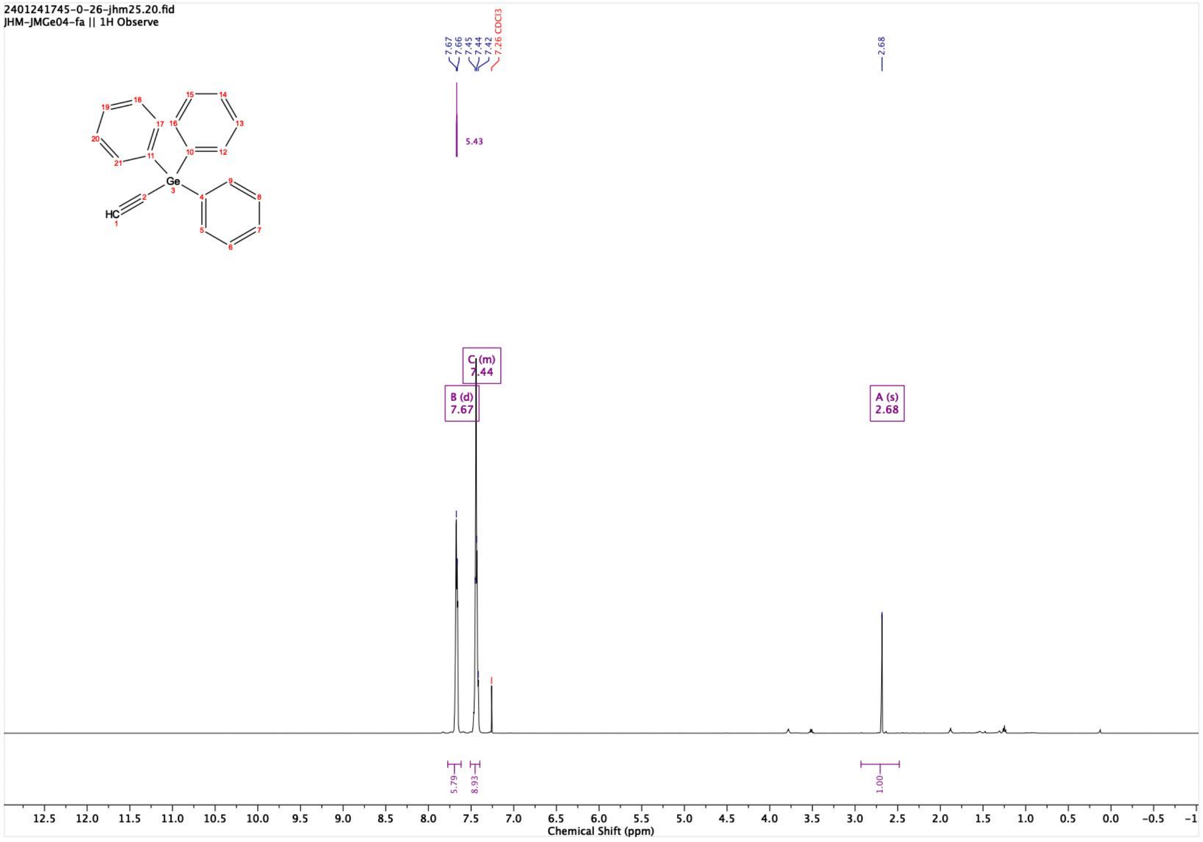

^{13}C DEPTQ

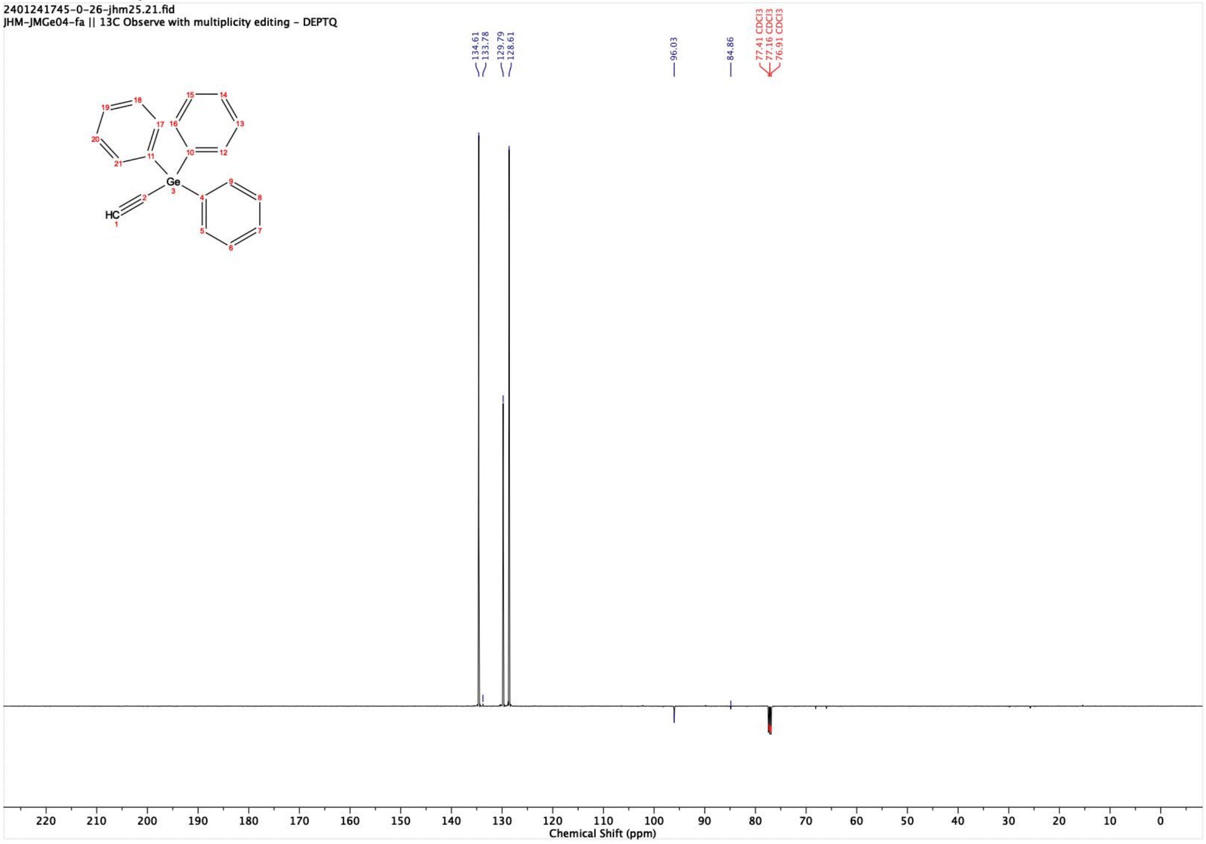

1 – ^{1}H

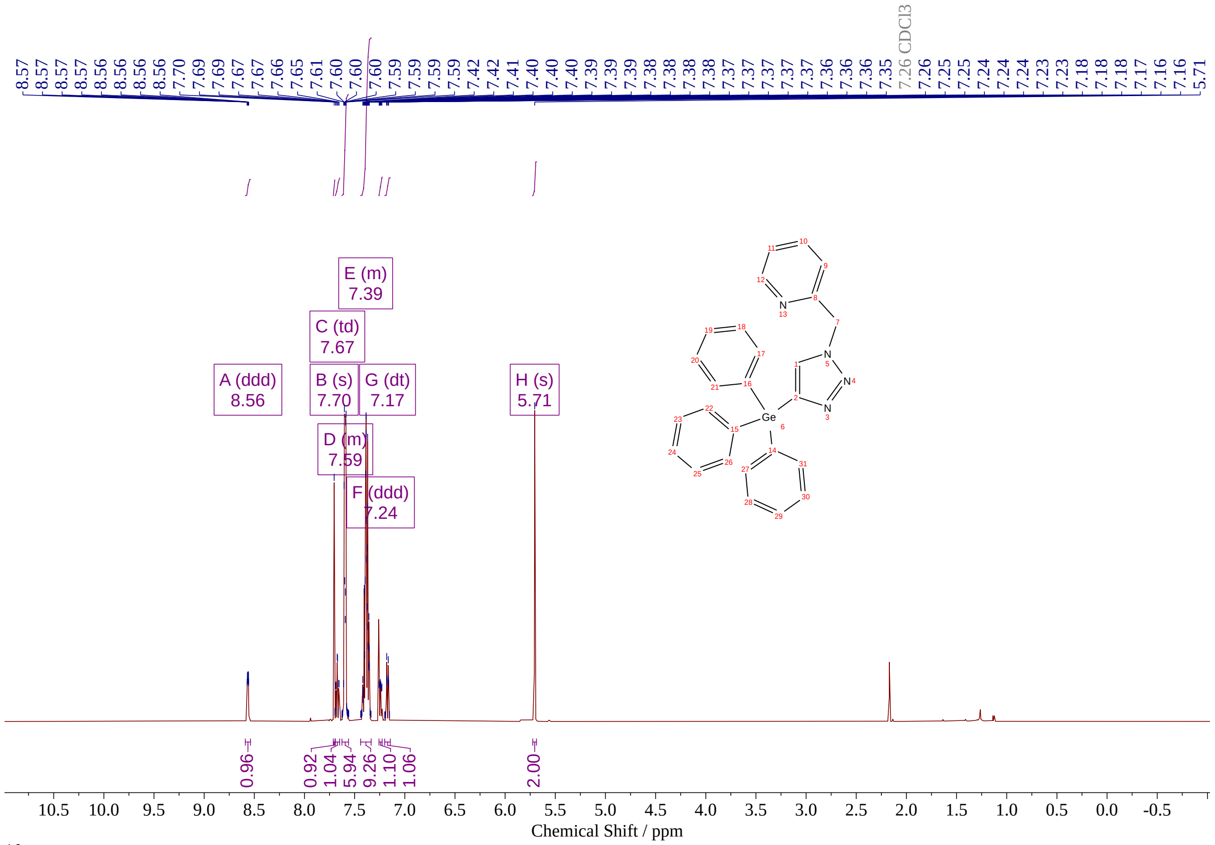

^{13}C DEPTQ

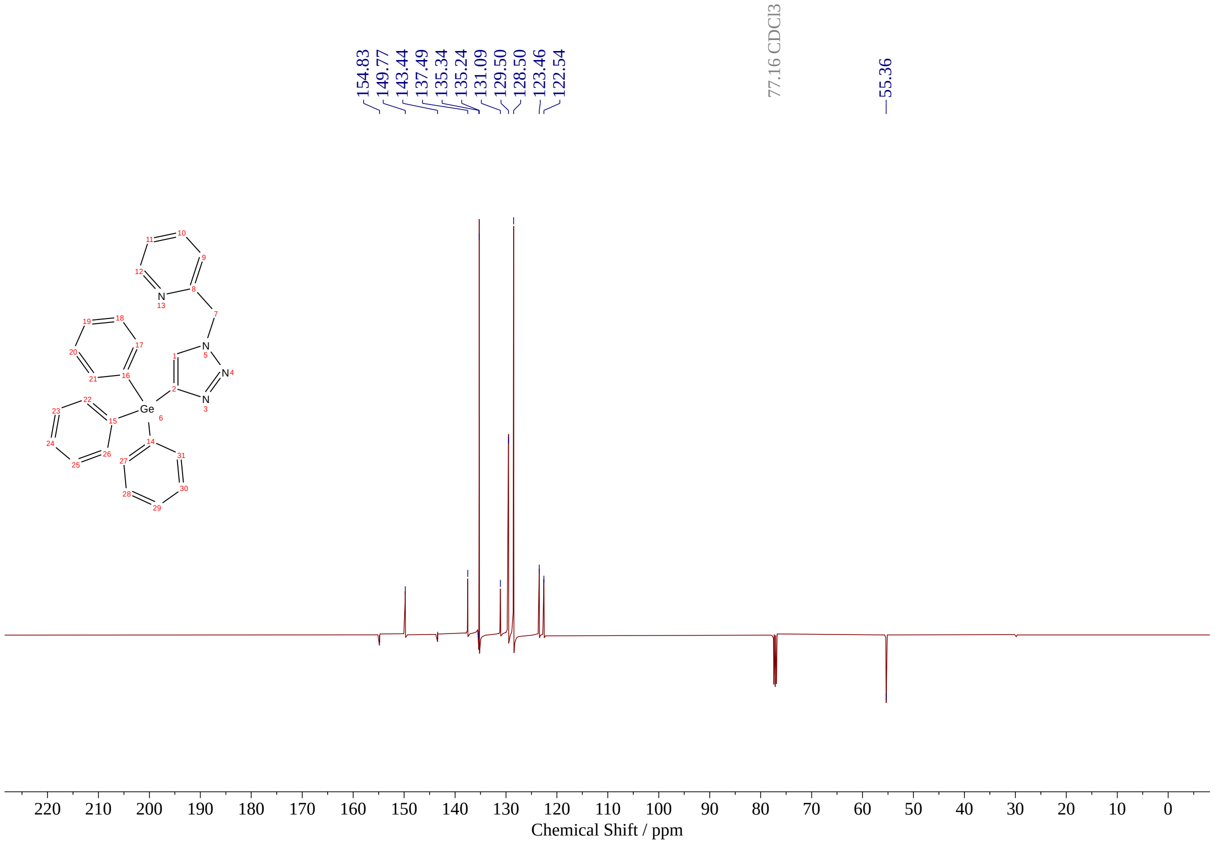

2 – ^{1}H

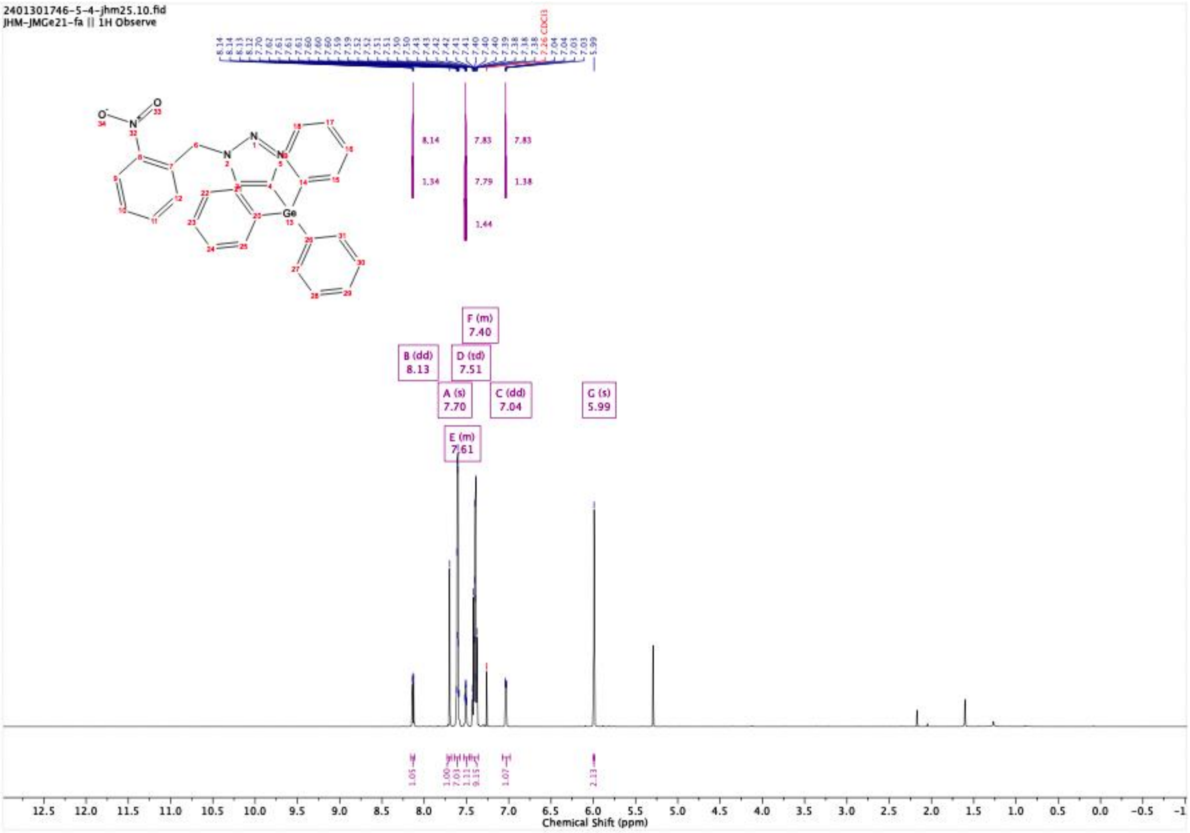

^{13}C DEPTQ

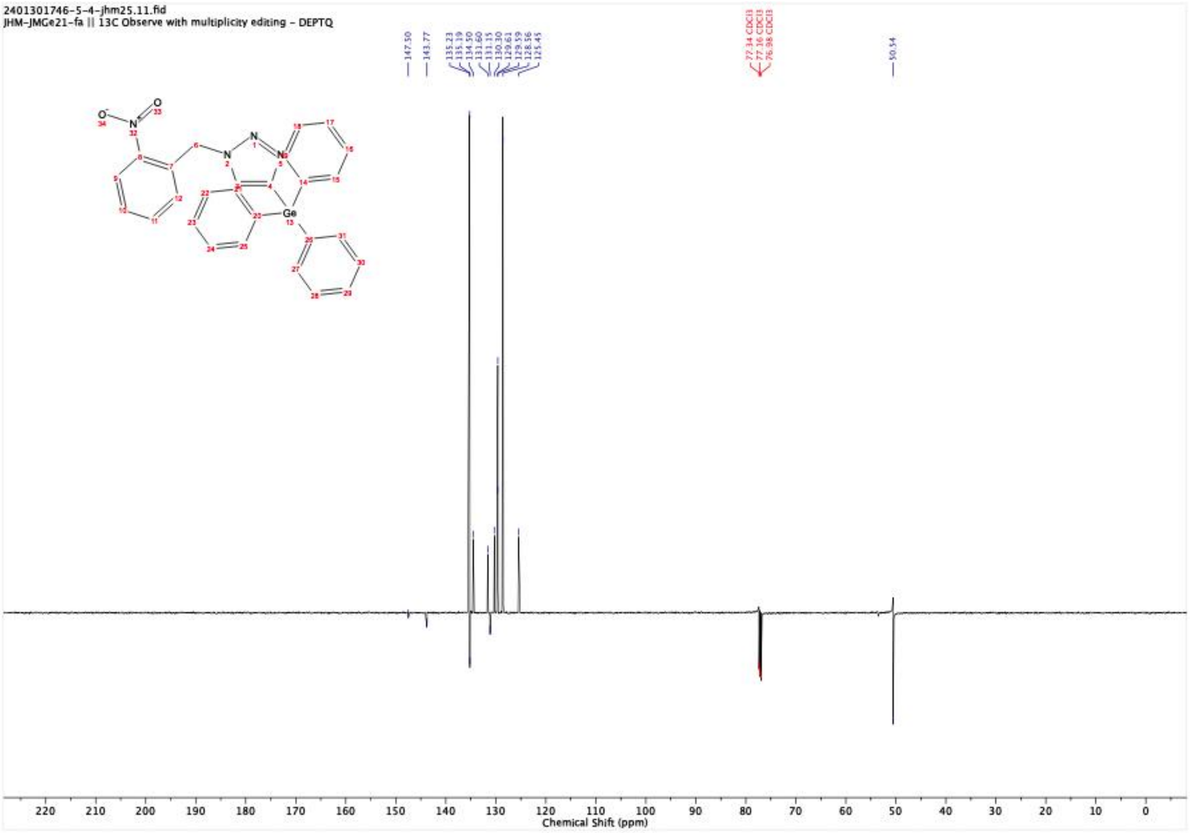

3 – ^{1}H

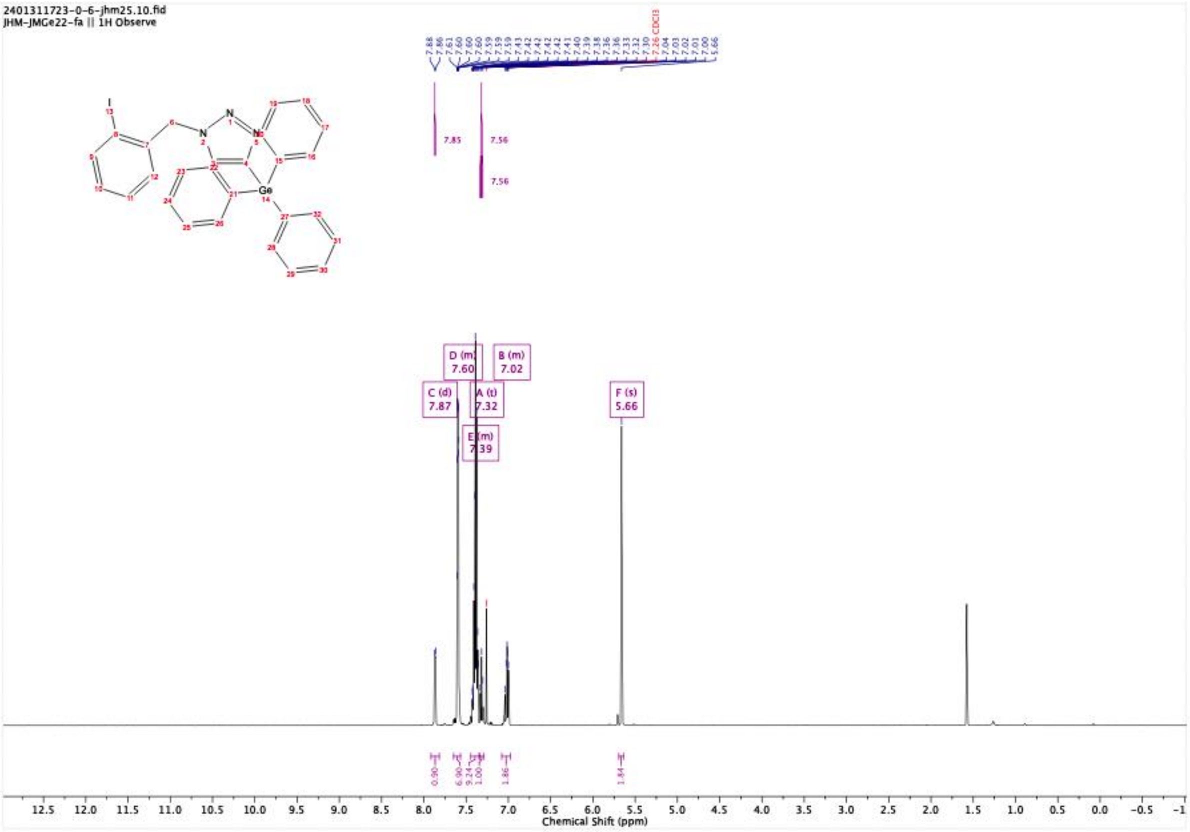

^{13}C DEPTQ

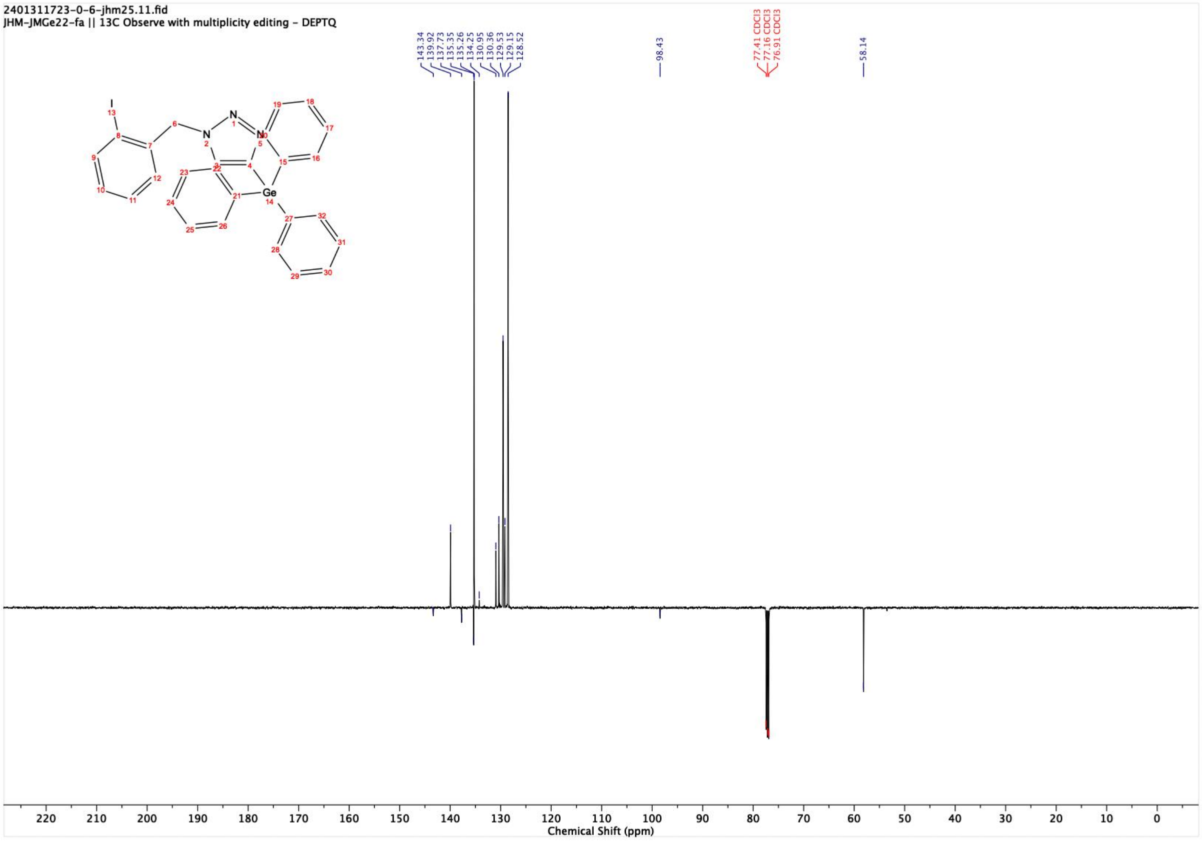

4 – $^{1}H\{^{19}F\}$

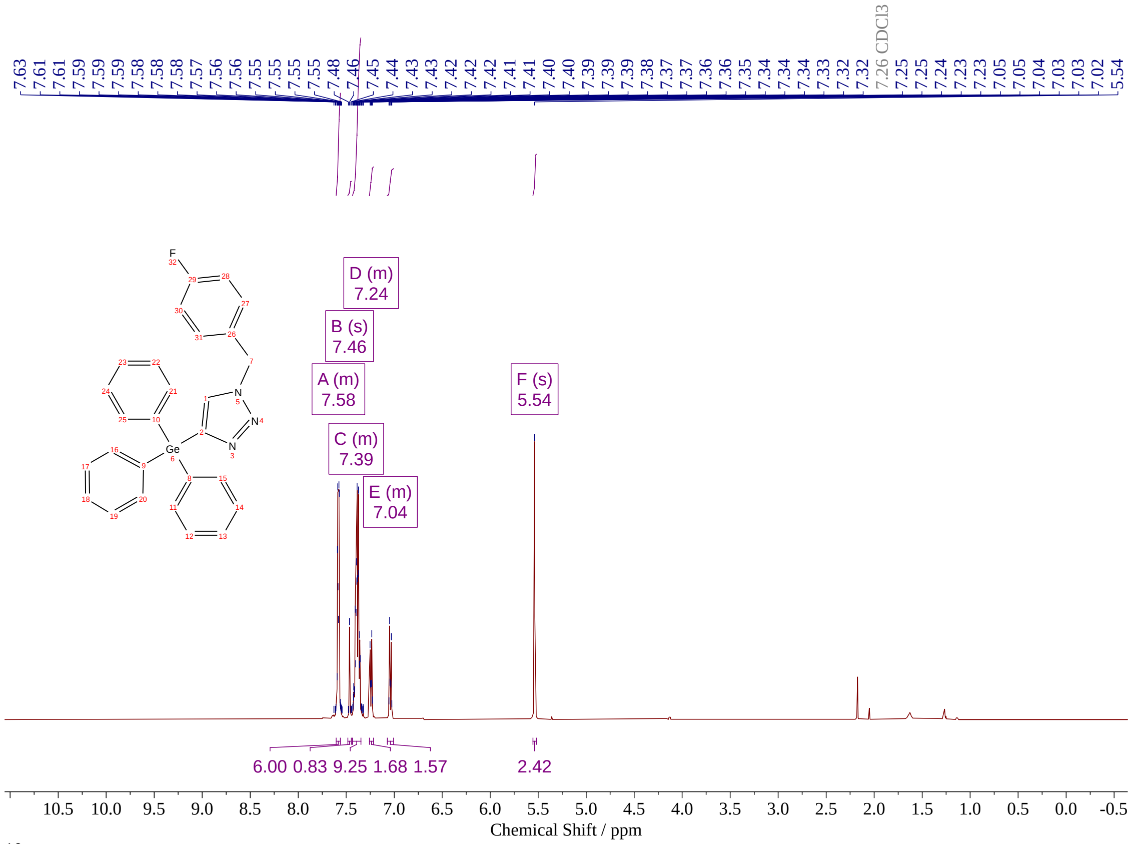

^{13}C DEPTQ

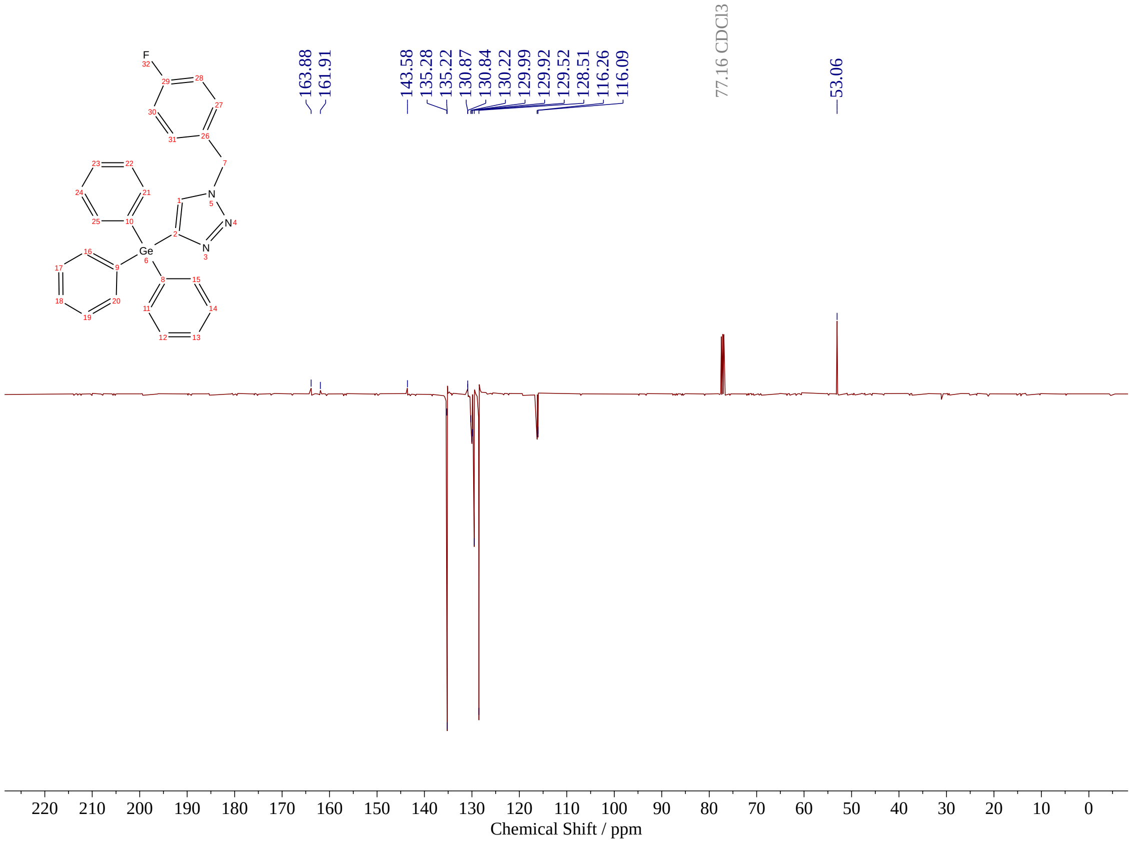

$^{19}F\{^{1}H\}$

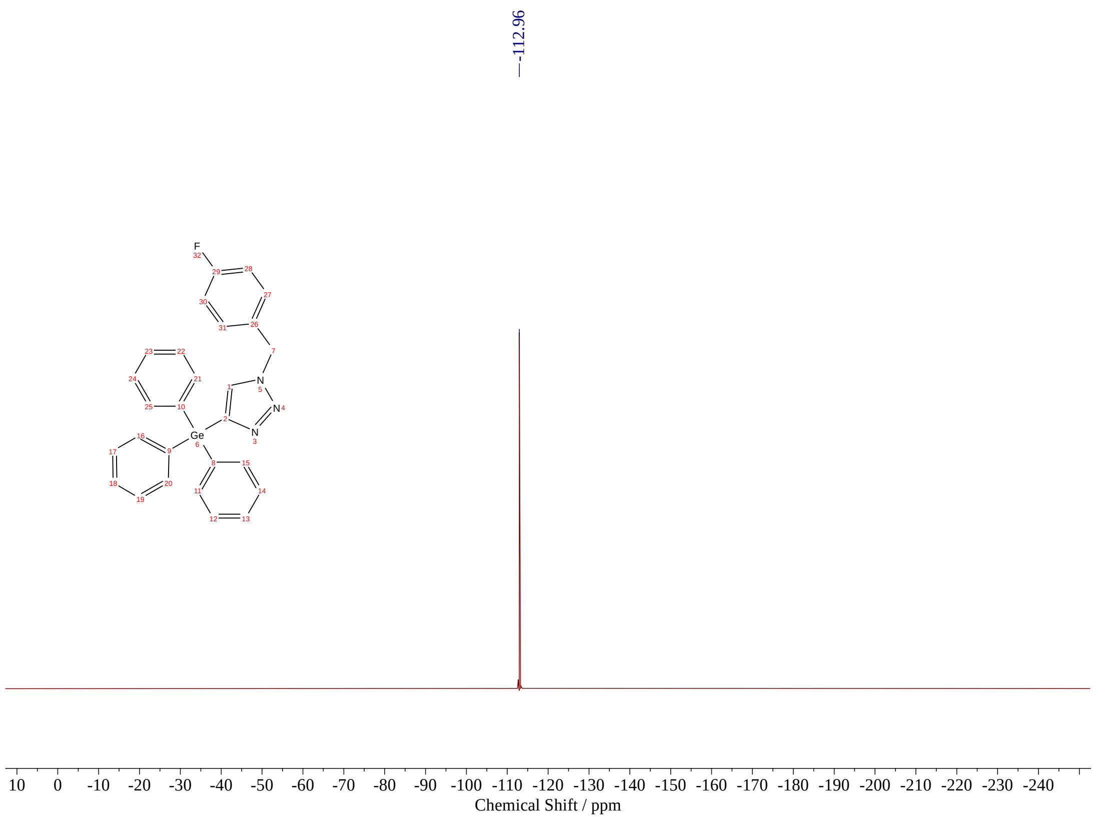
-112.96
F
Ge
N
N
N
10 0 -10 -20 -30 -40 -50 -60 -70 -80 -90 -100 -110 -120 -130 -140 -150 -160 -170 -180 -190 -200 -210 -220 -230 -240
Chemical Shift / ppm

5 – ^{1}H

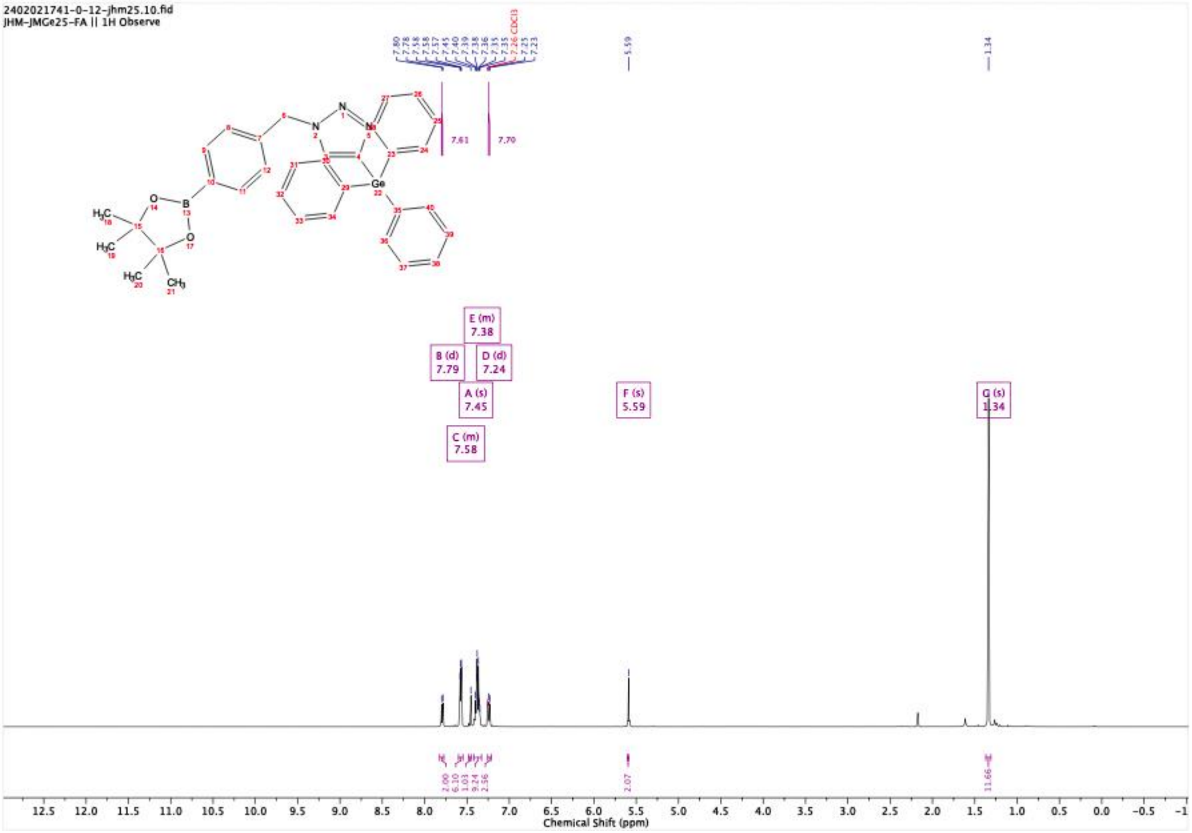

^{13}C DEPTQ

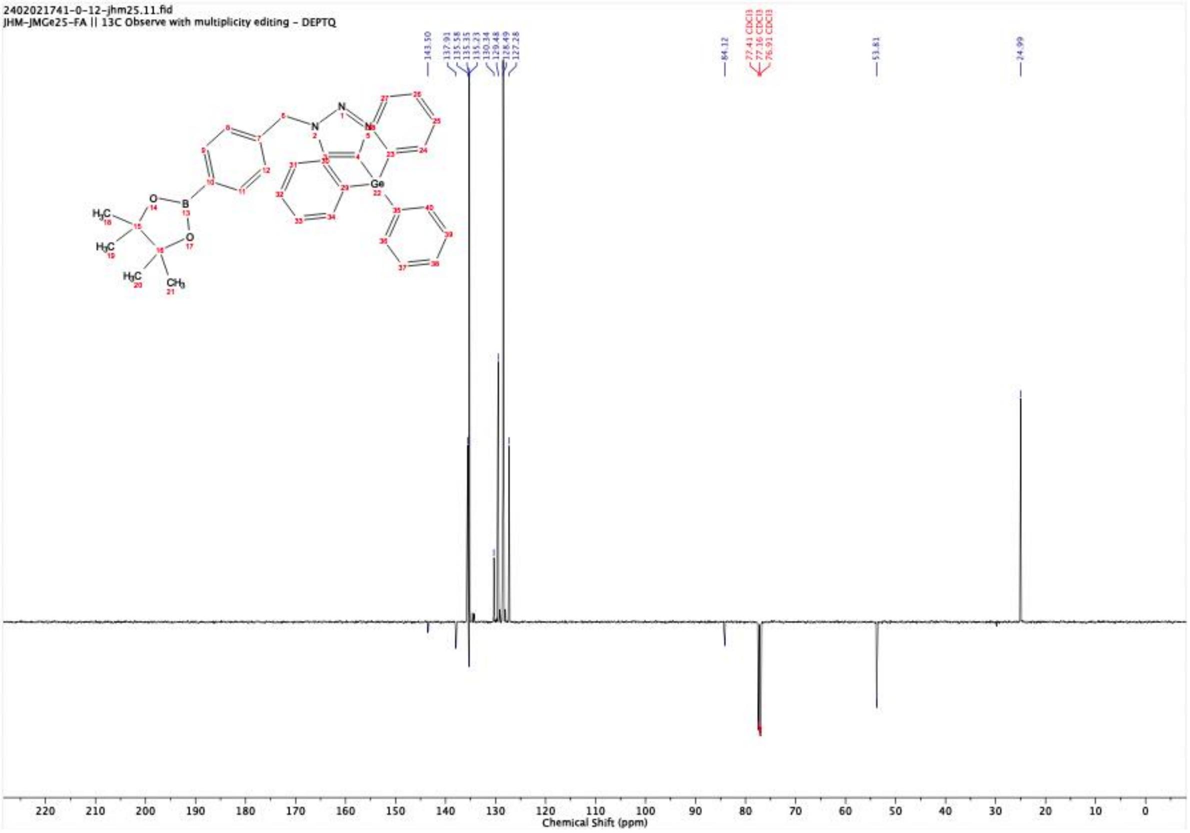

^{11}B

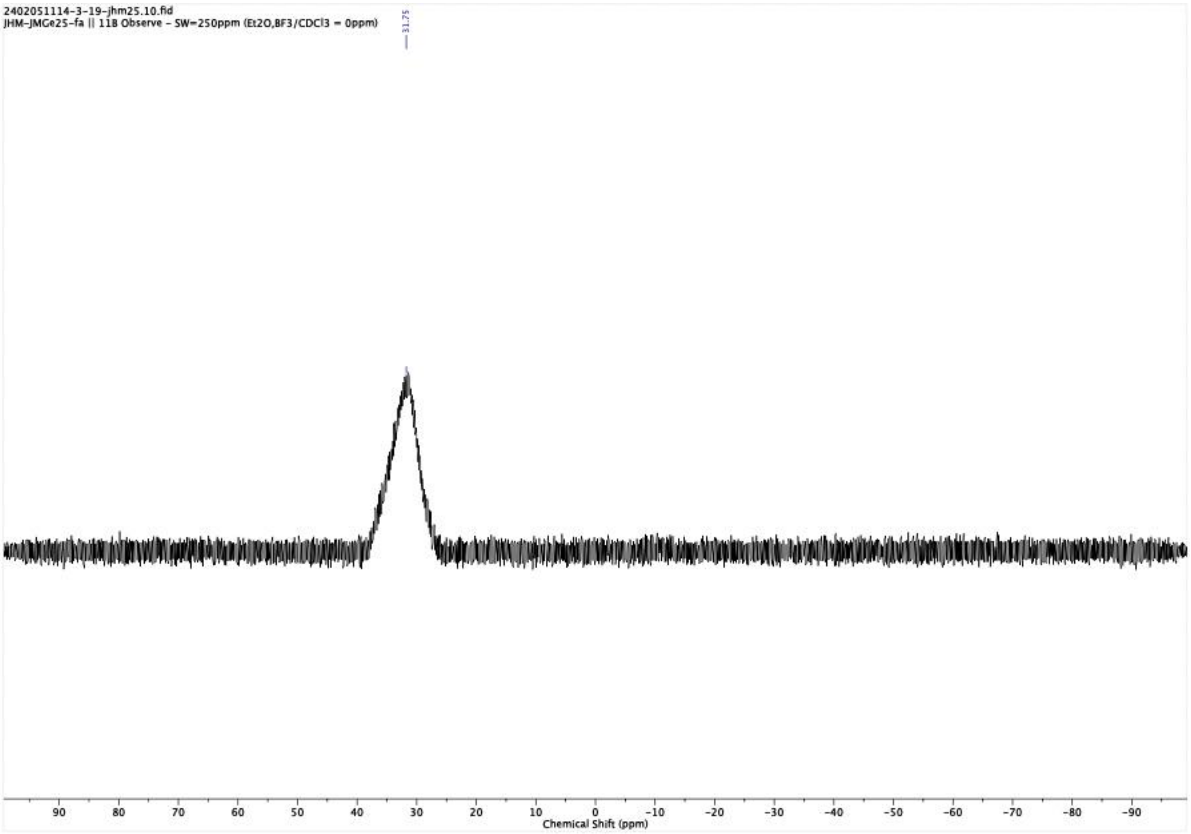

2402051114-3-19-jhm25.10.fid
JHM-JMGe25-fa || 11B Observe - SW=250ppm (Et2O,BF3/CDCl3 = 0ppm)
31.75
90 80 70 60 50 40 30 20 10 0 -10 -20 -30 -40 -50 -60 -70 -80 -90
Chemical Shift (ppm)

6 – $^{1}H\{^{19}F\}$

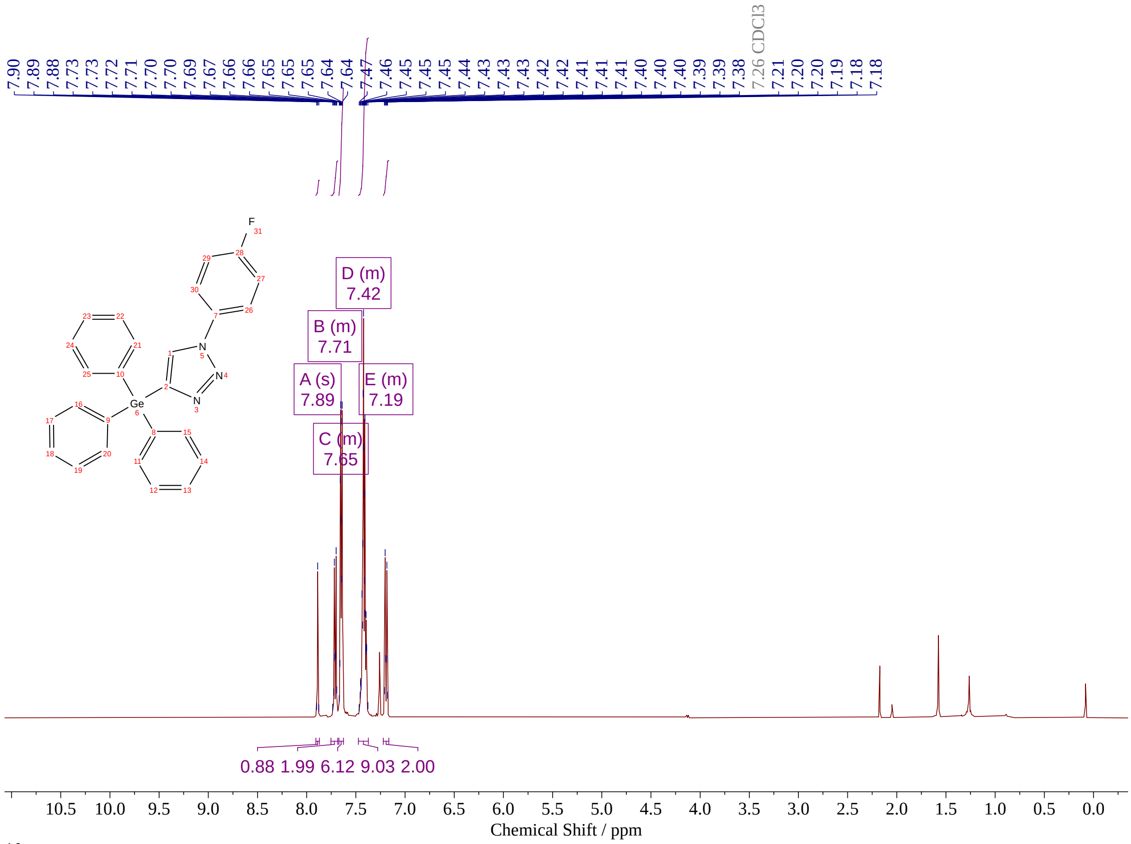

^{13}C DEPTQ

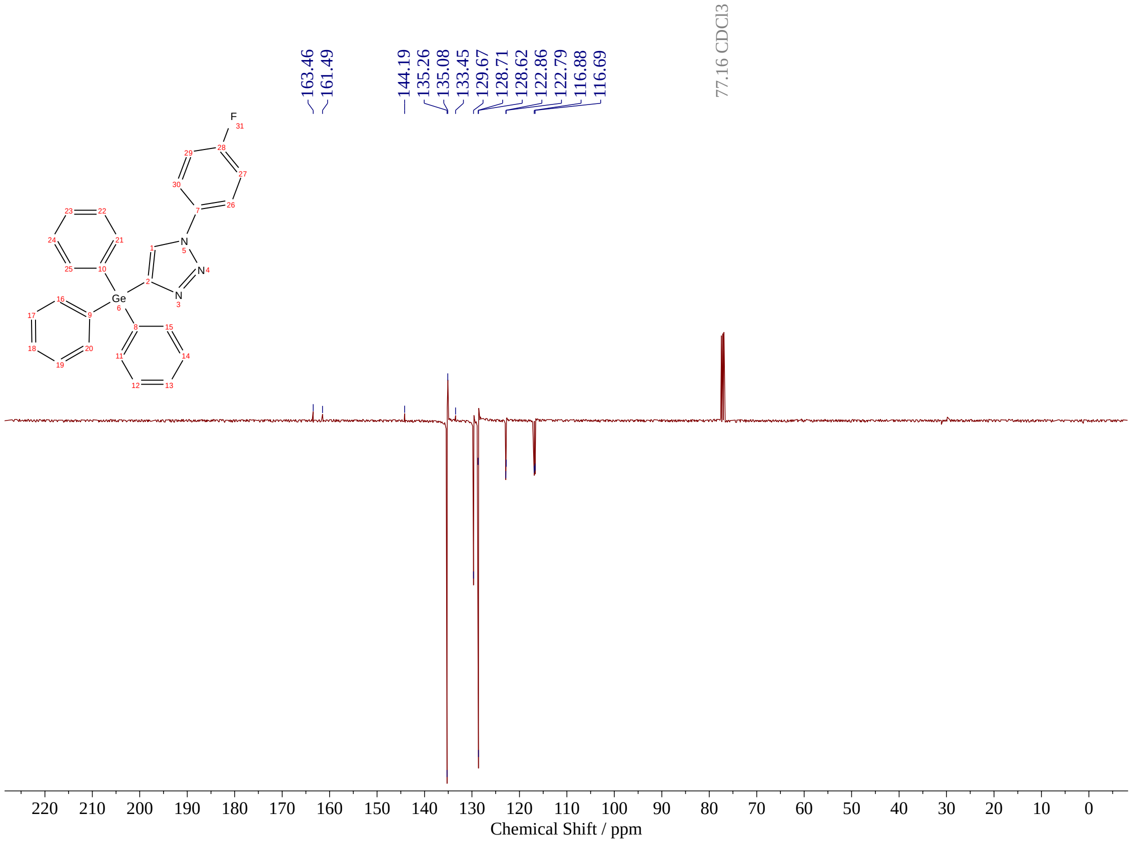

$^{19}F\{^{1}H\}$

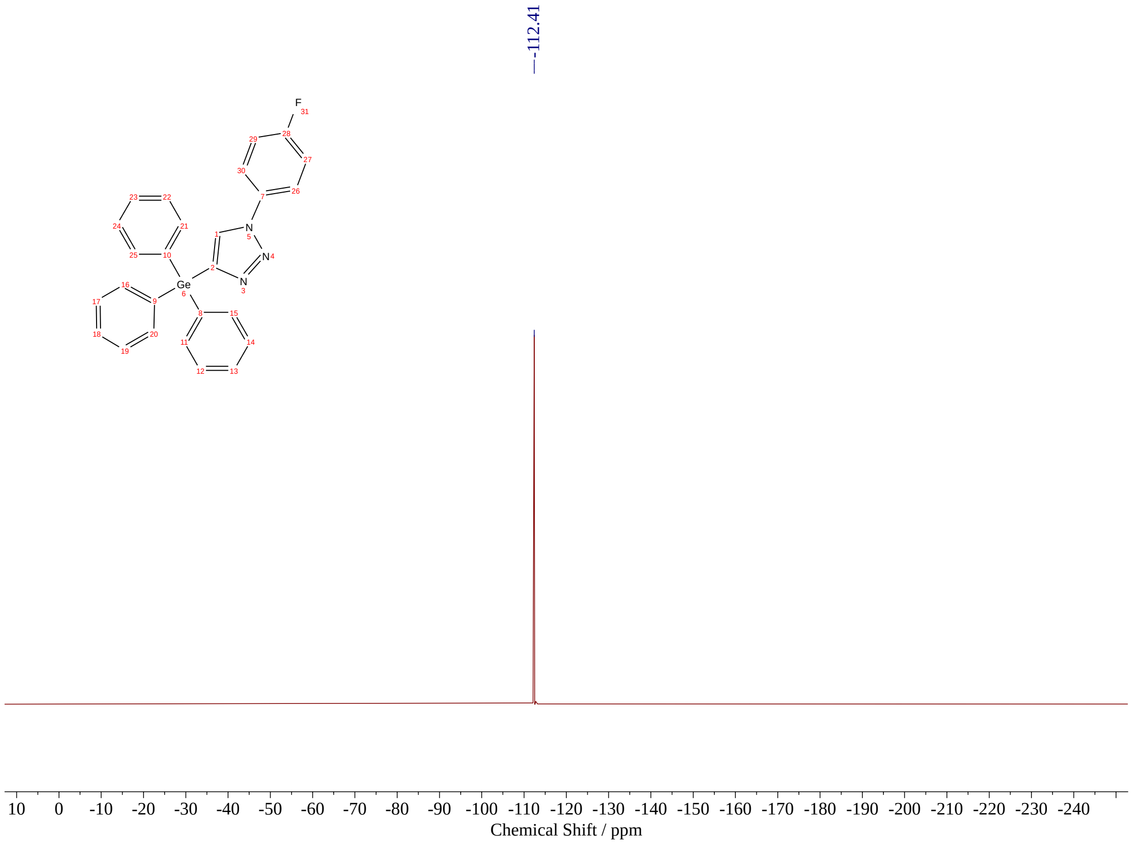
-112.41
F
Ge
N
N
N
10
0
-10
-20
-30
-40
-50
-60
-70
-80
-90
-100
-110
-120
-130
-140
-150
-160
-170
-180
-190
-200
-210
-220
-230
-240
Chemical Shift / ppm

7 – ^{1}H

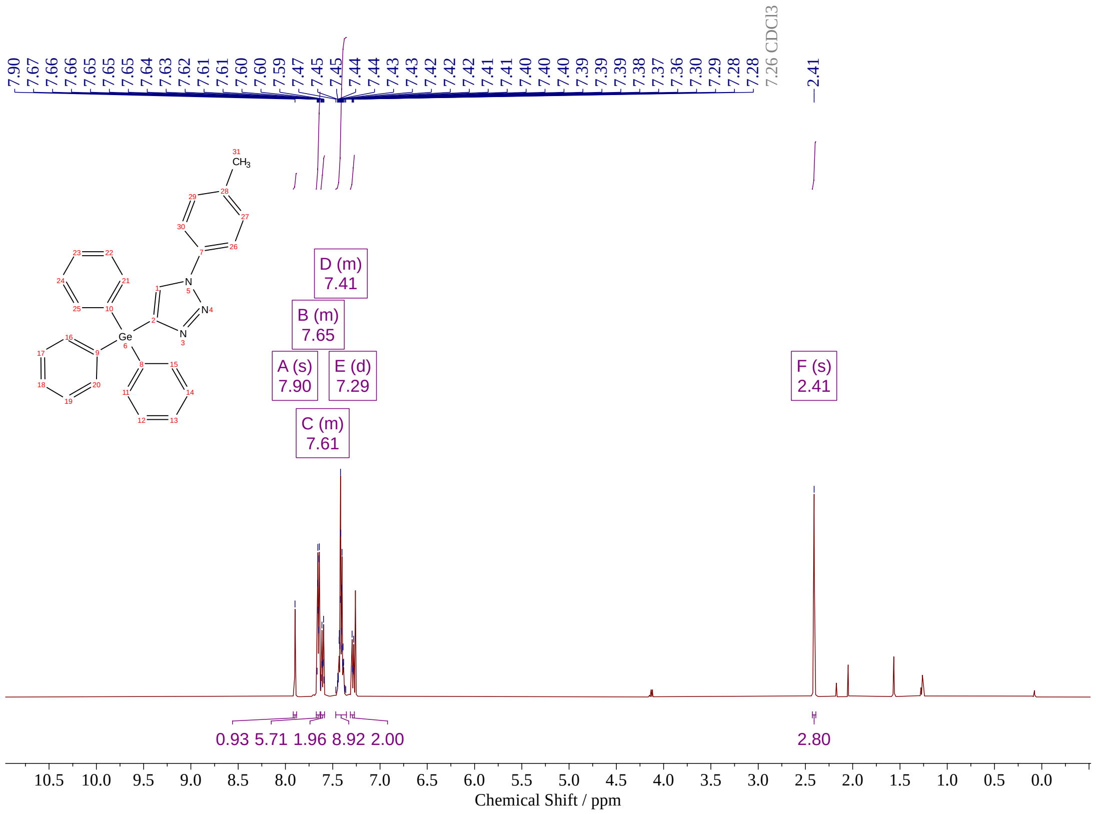

^{13}C DEPTQ

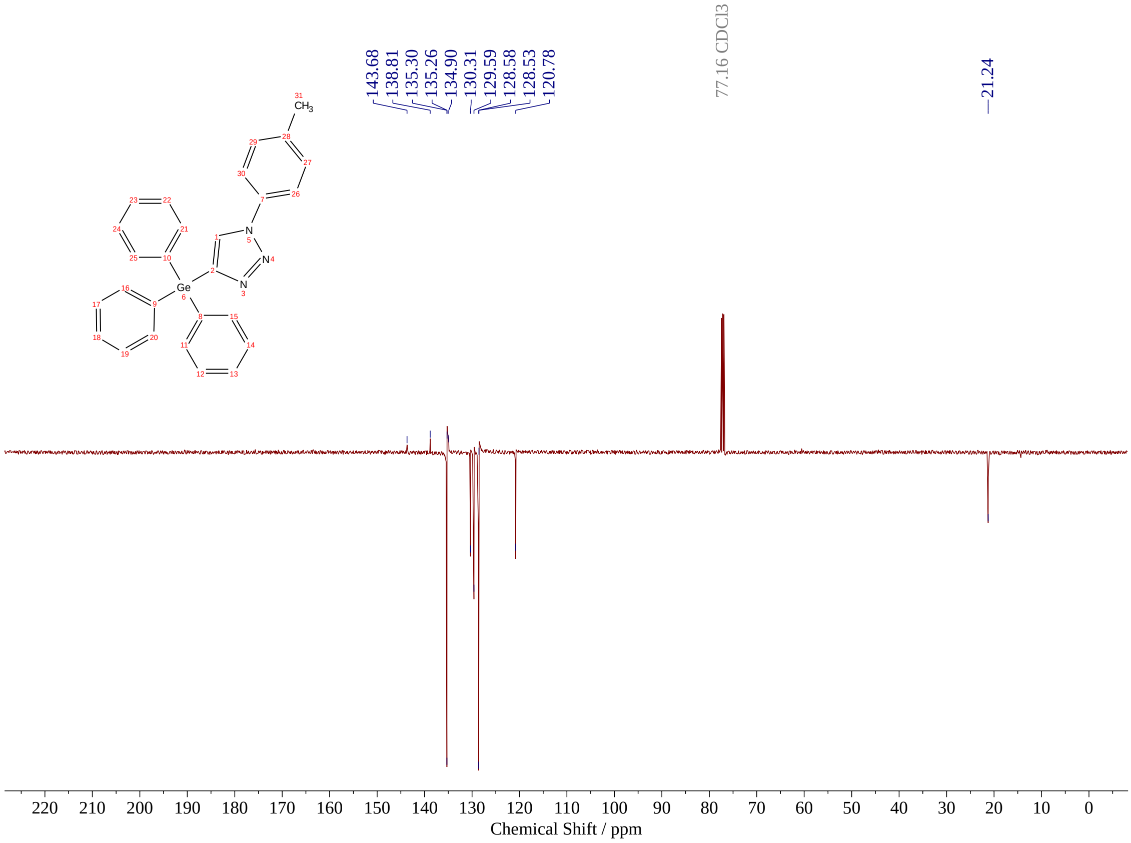

8 – ^{1}H

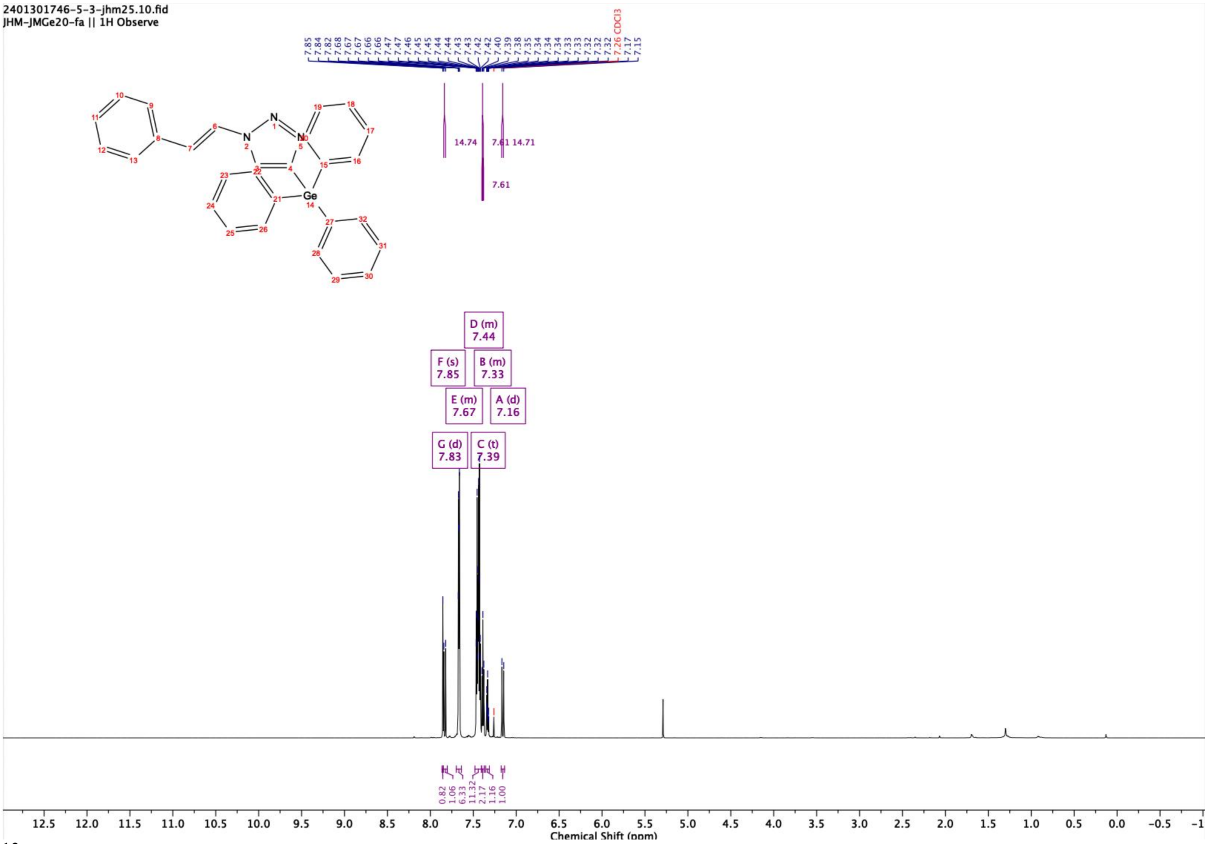

^{13}C DEPTQ

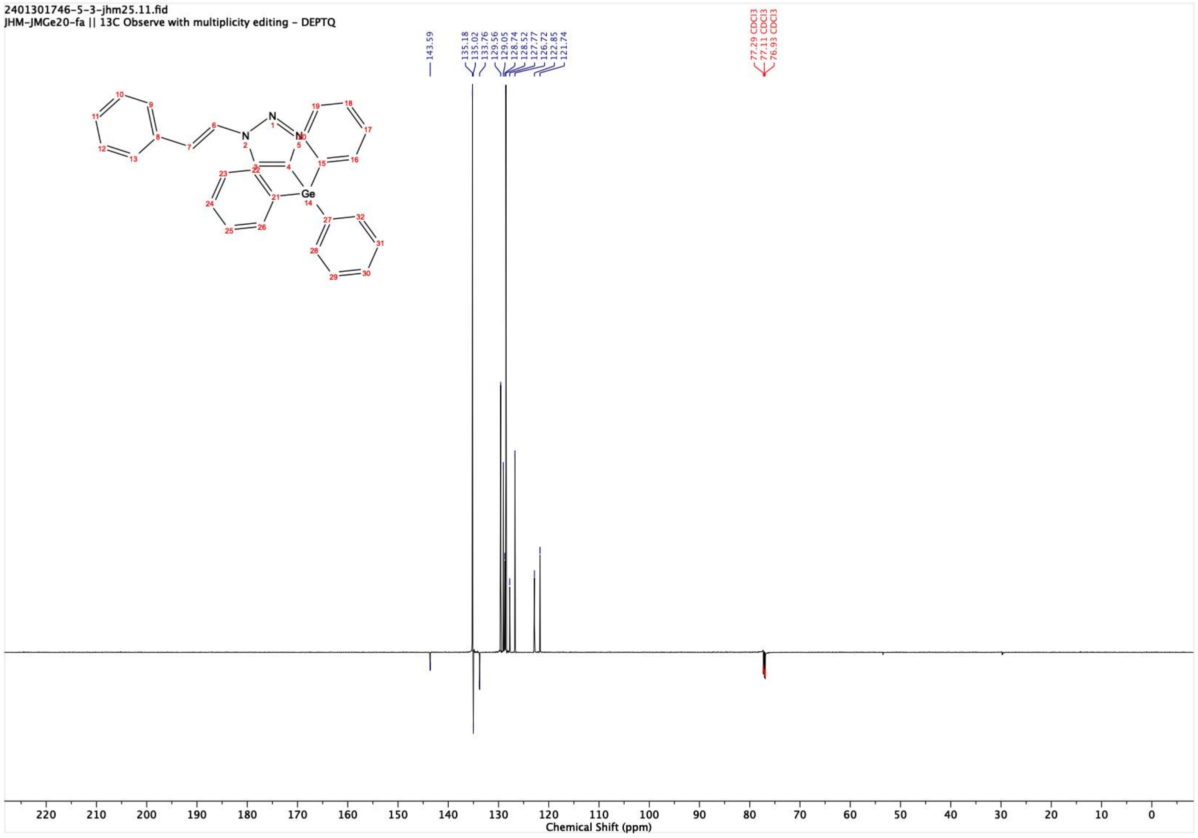

9 – ^{1}H

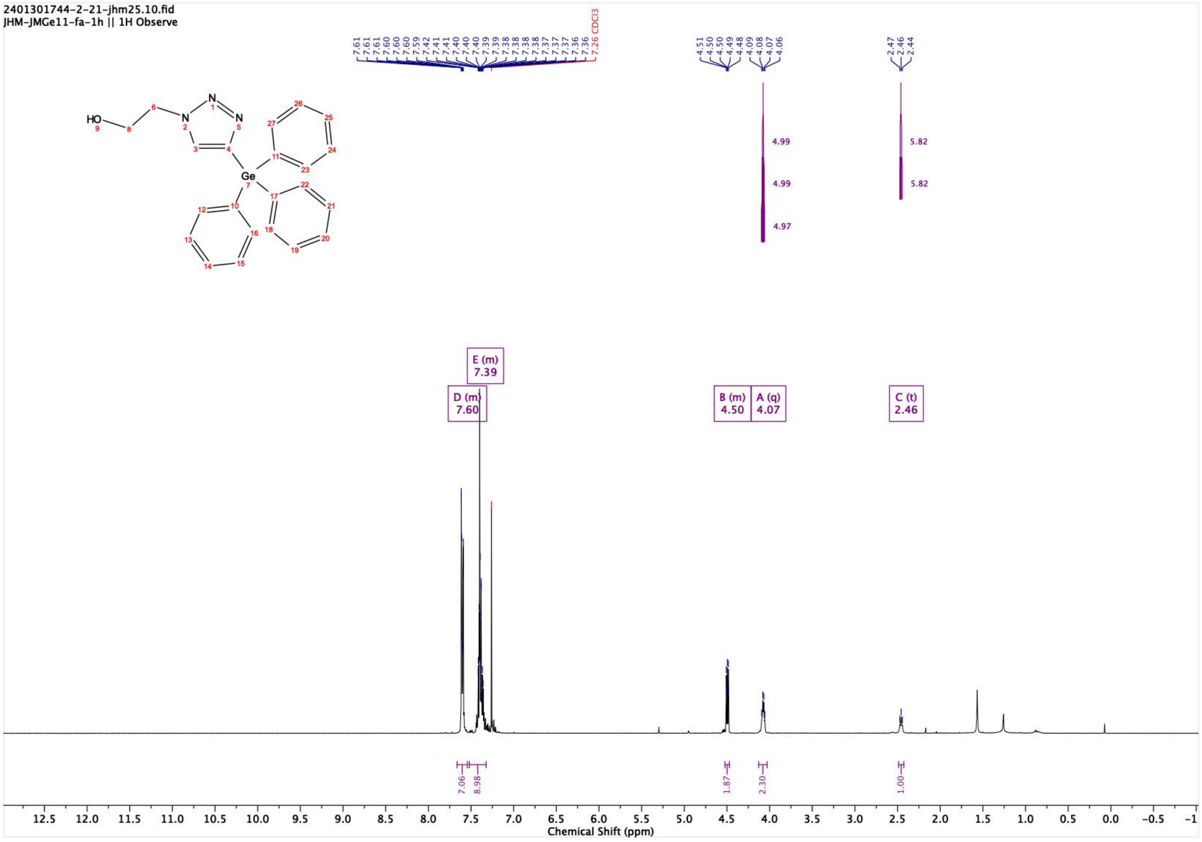

^{13}C DEPTQ

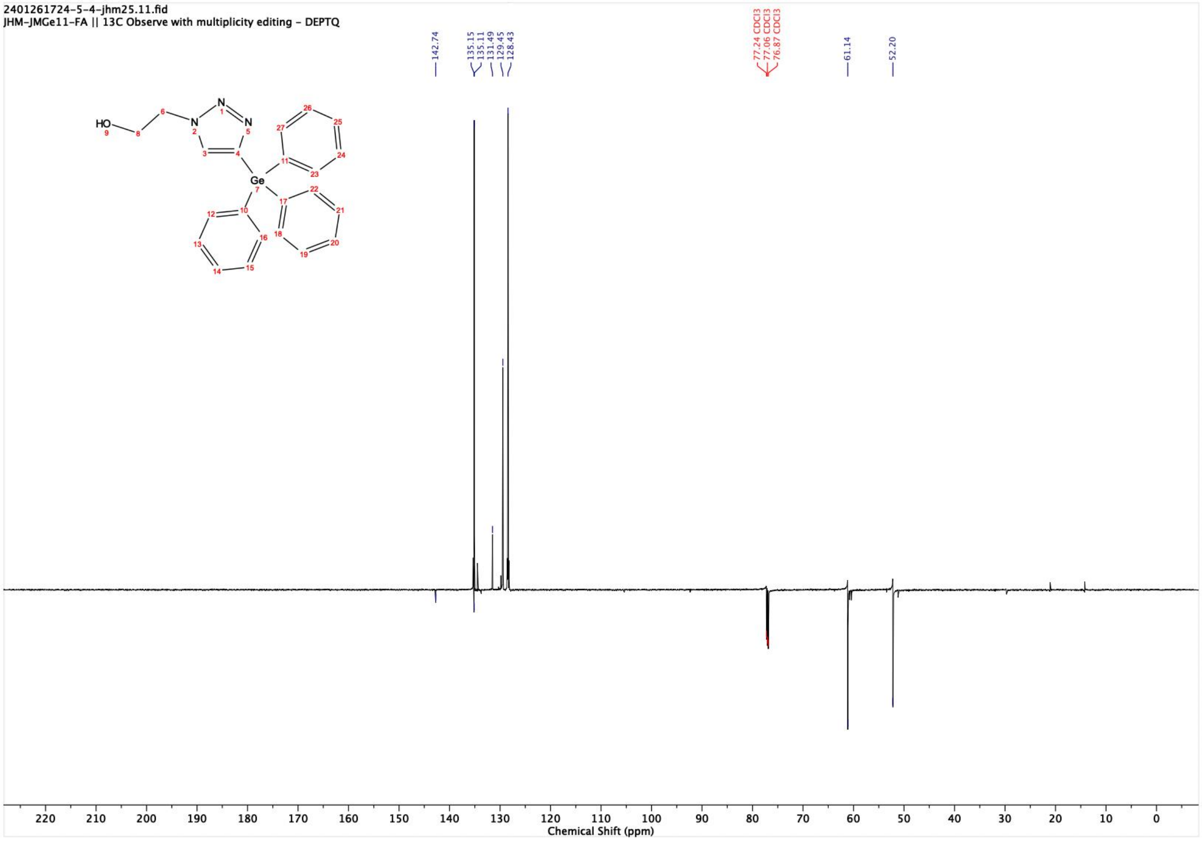

10 – ^{1}H

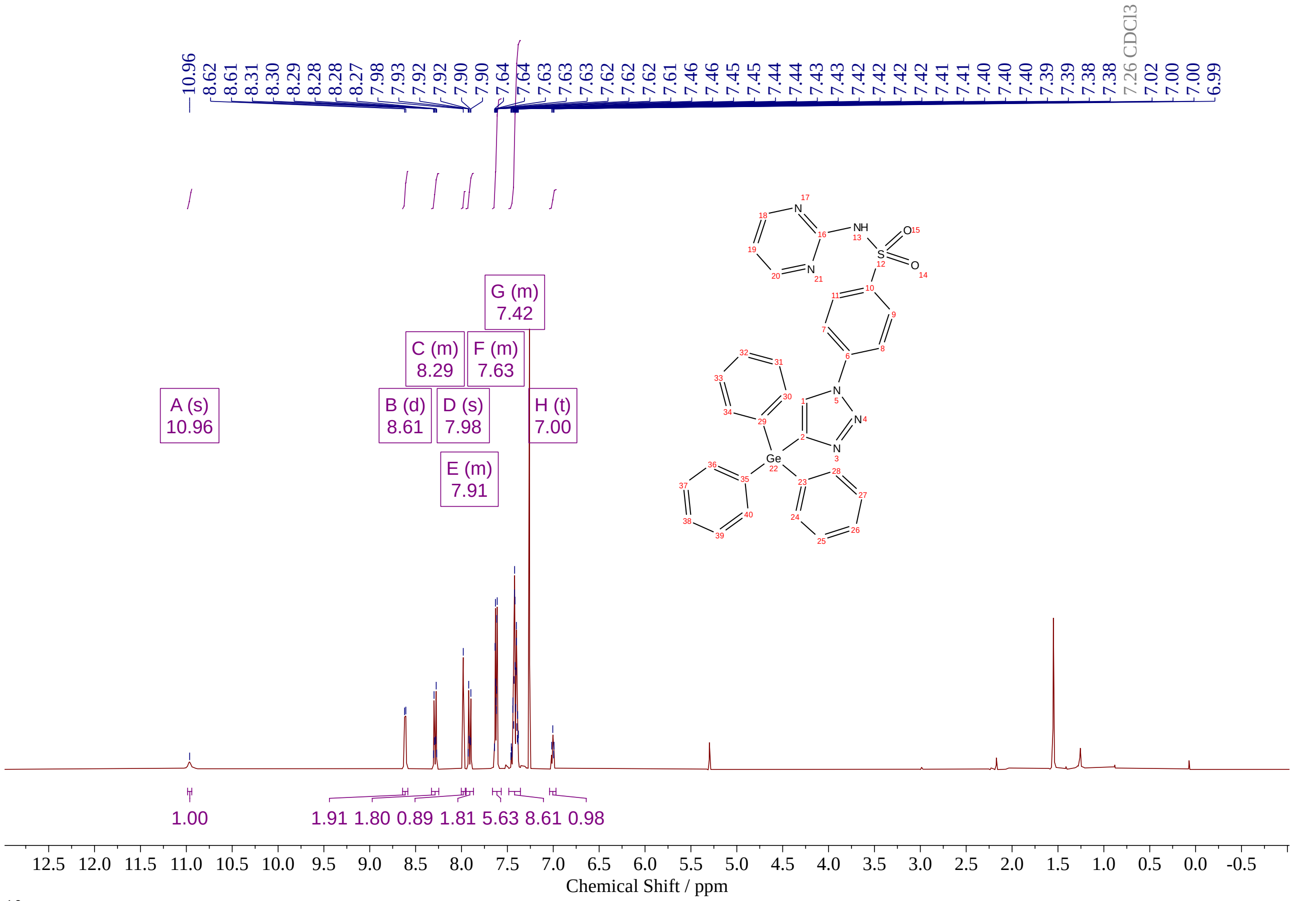

^{13}C DEPTQ

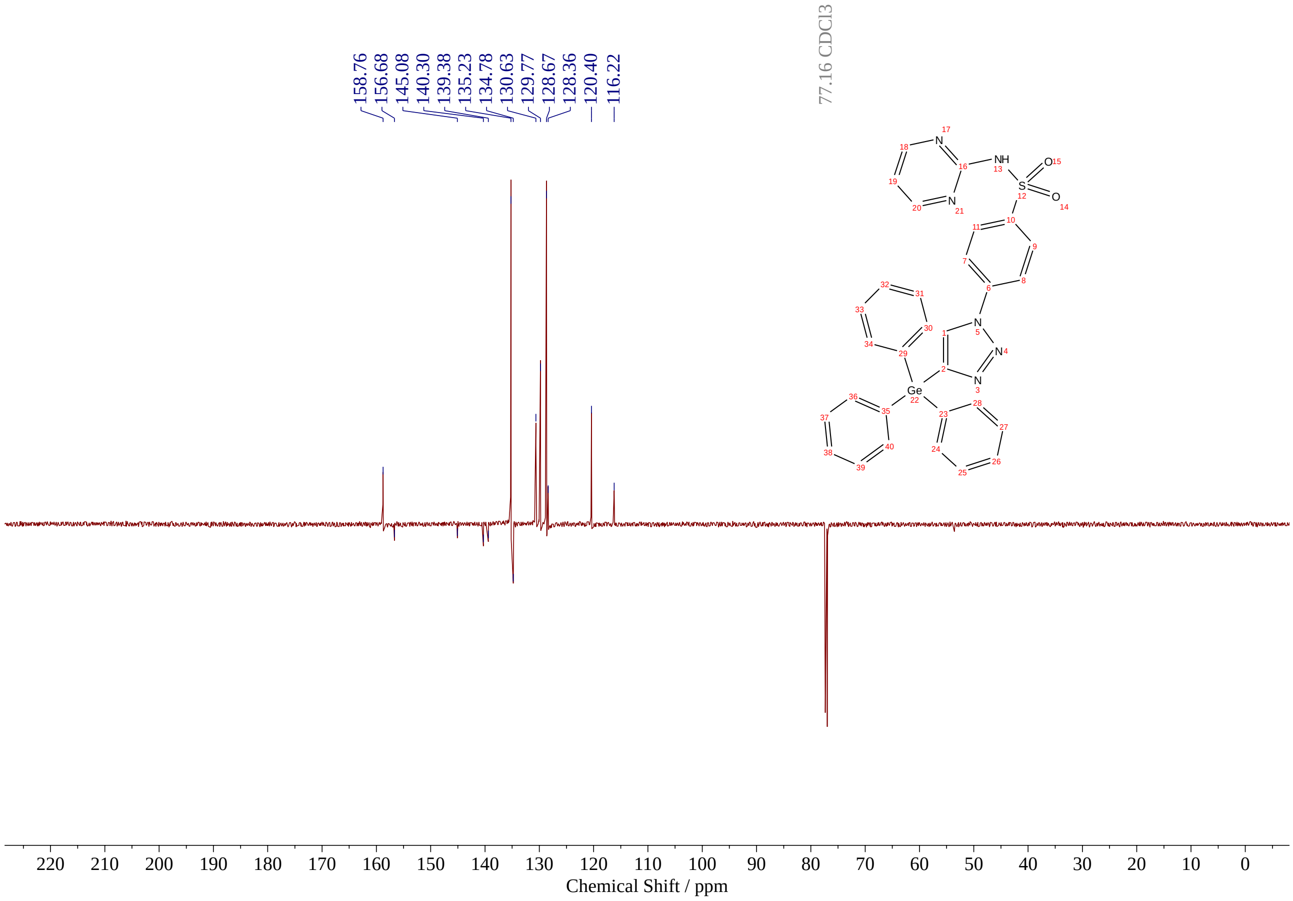

11 – ^{1}H

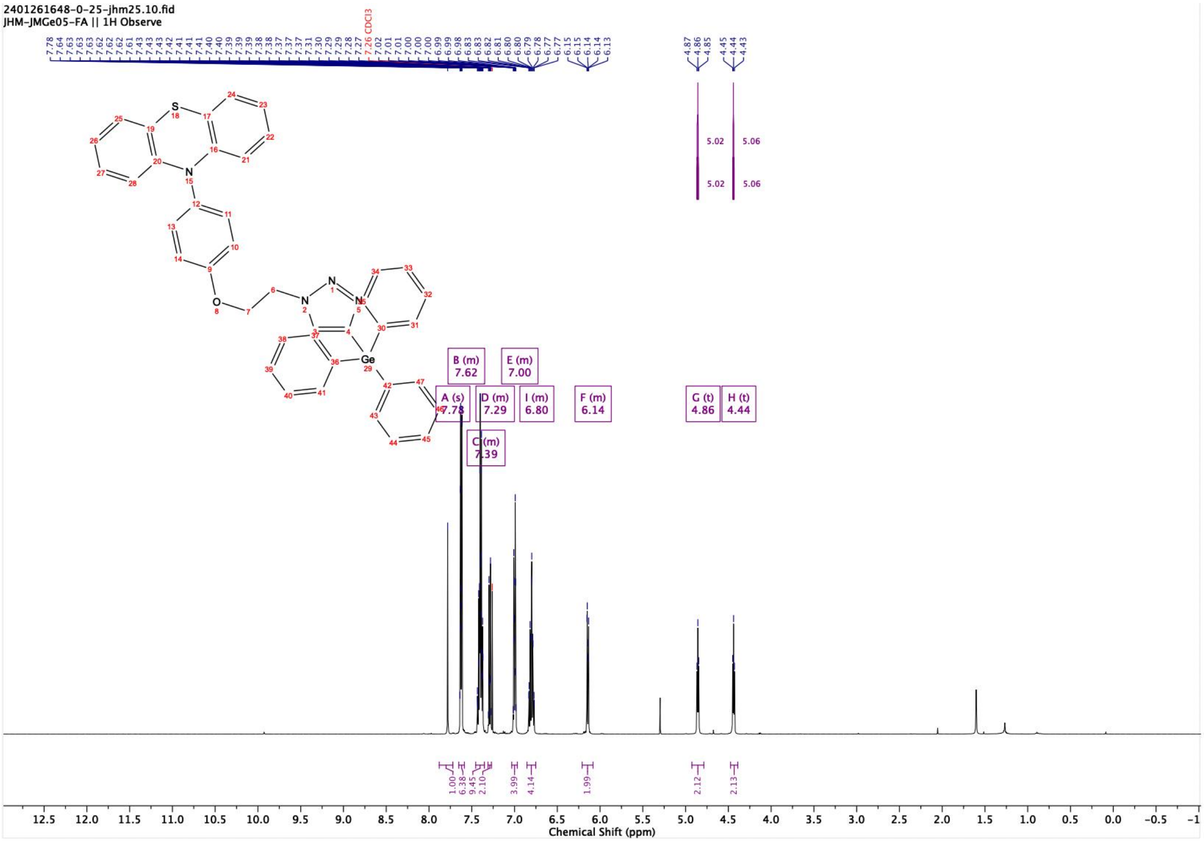

^{13}C DEPTQ

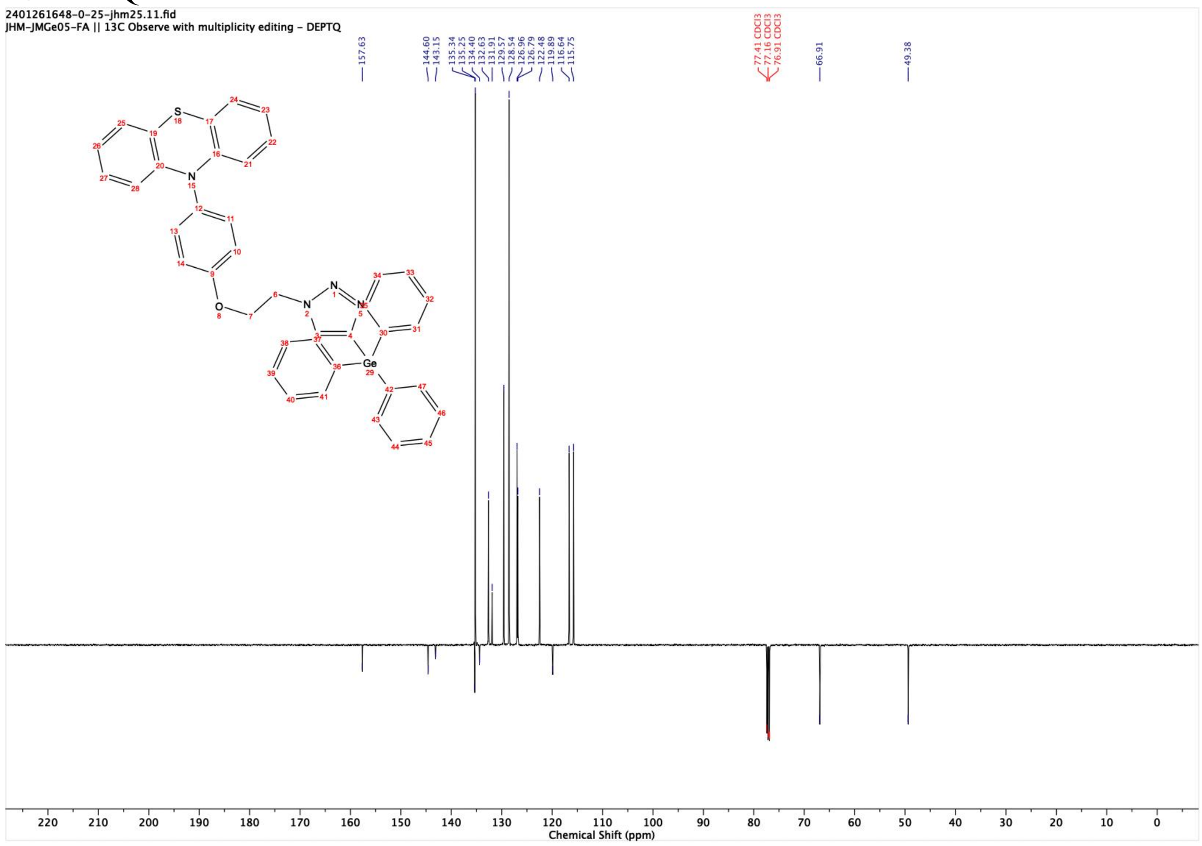

12 – [1]H

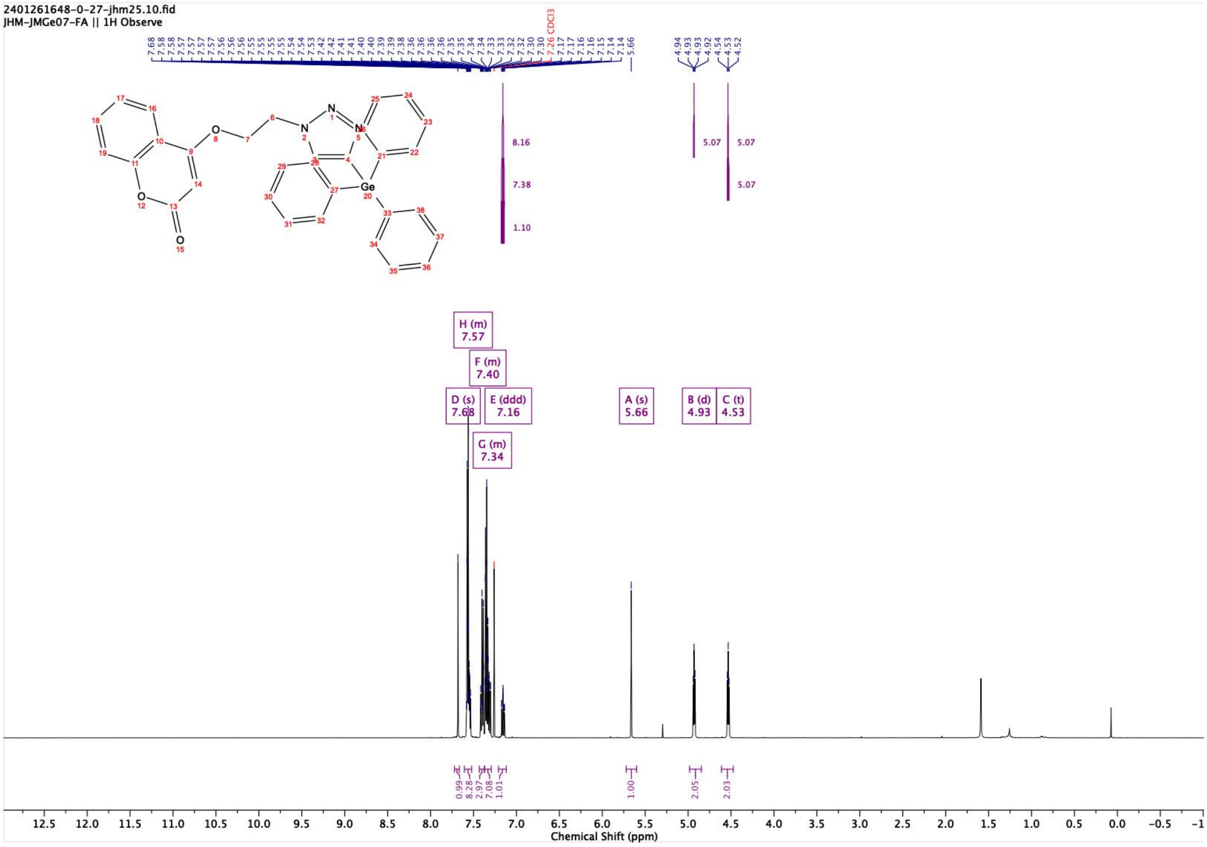

^{13}C DEPTQ

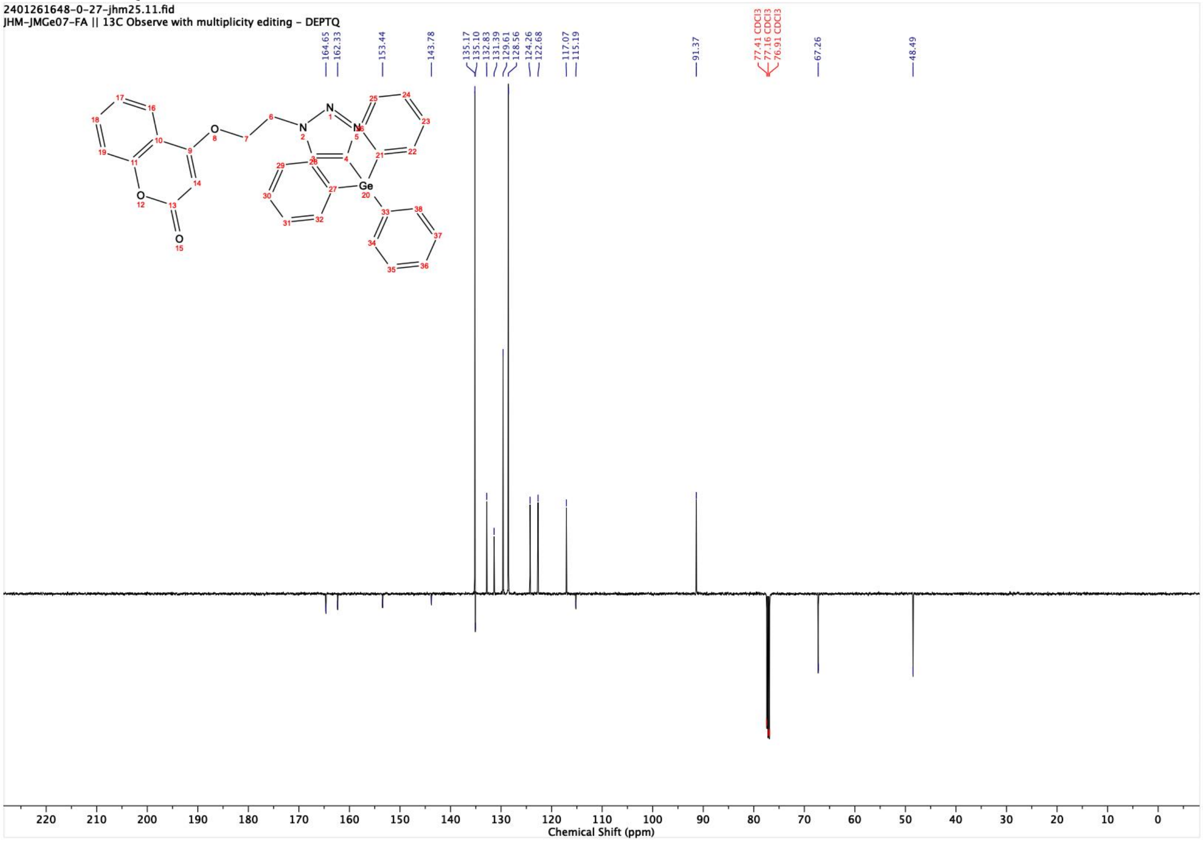

13 – ^{1}H

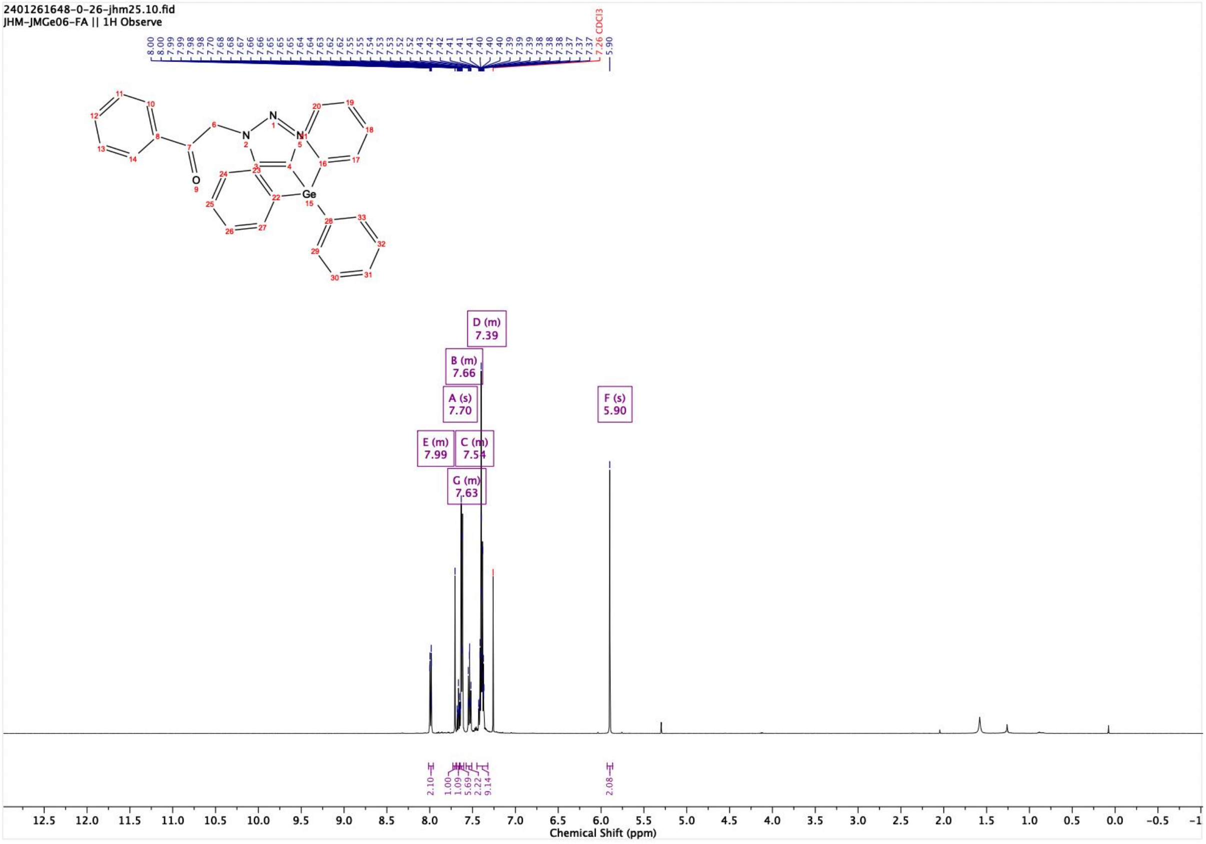

^{13}C DEPTQ

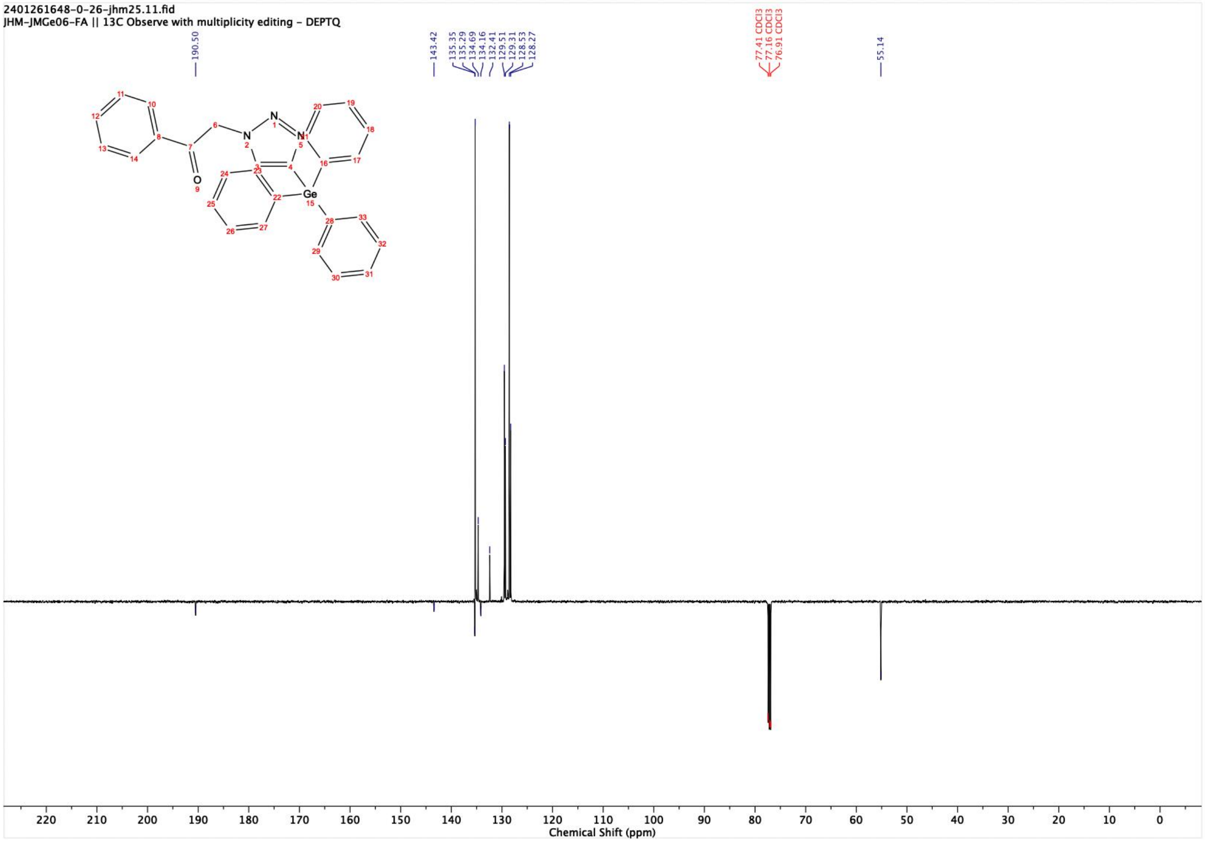

14 – ¹H

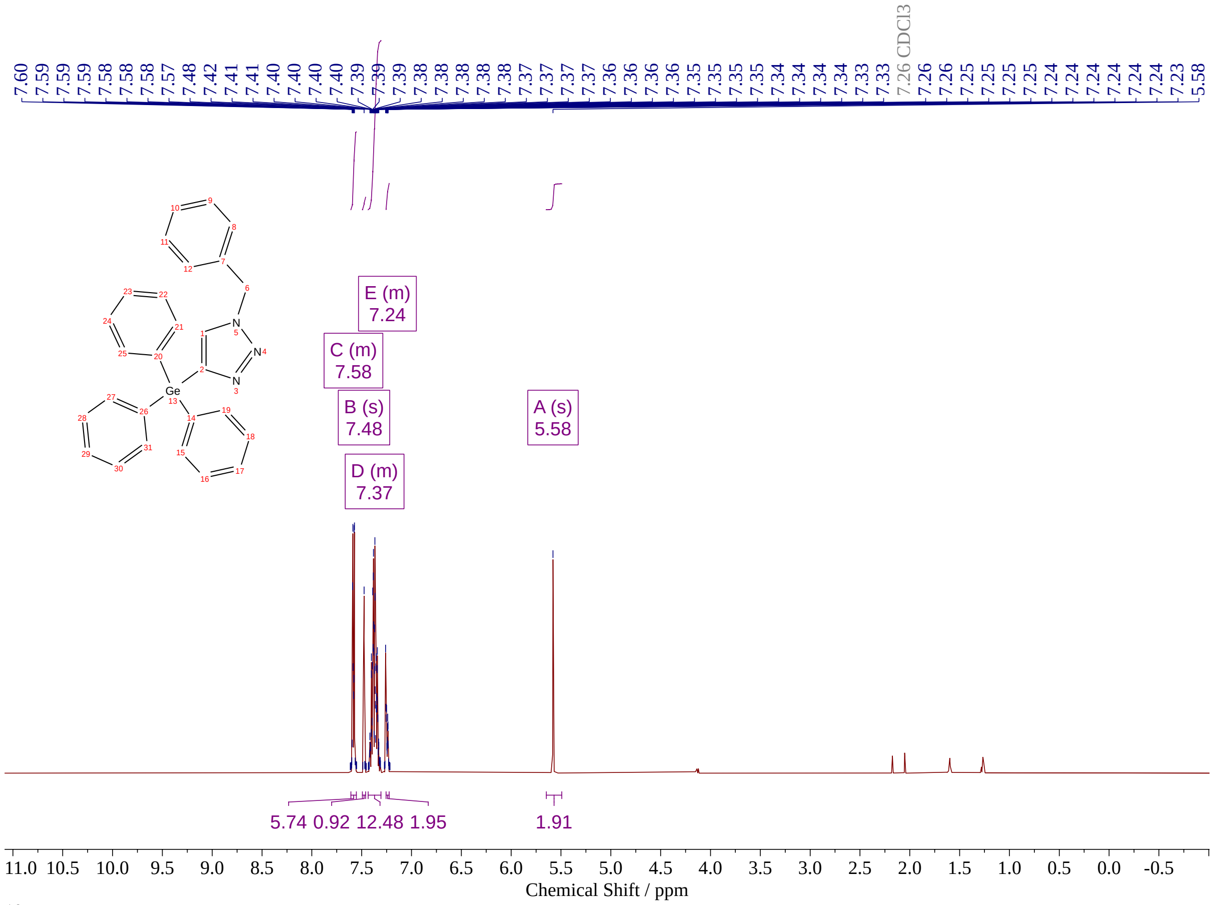

¹³C DEPTQ

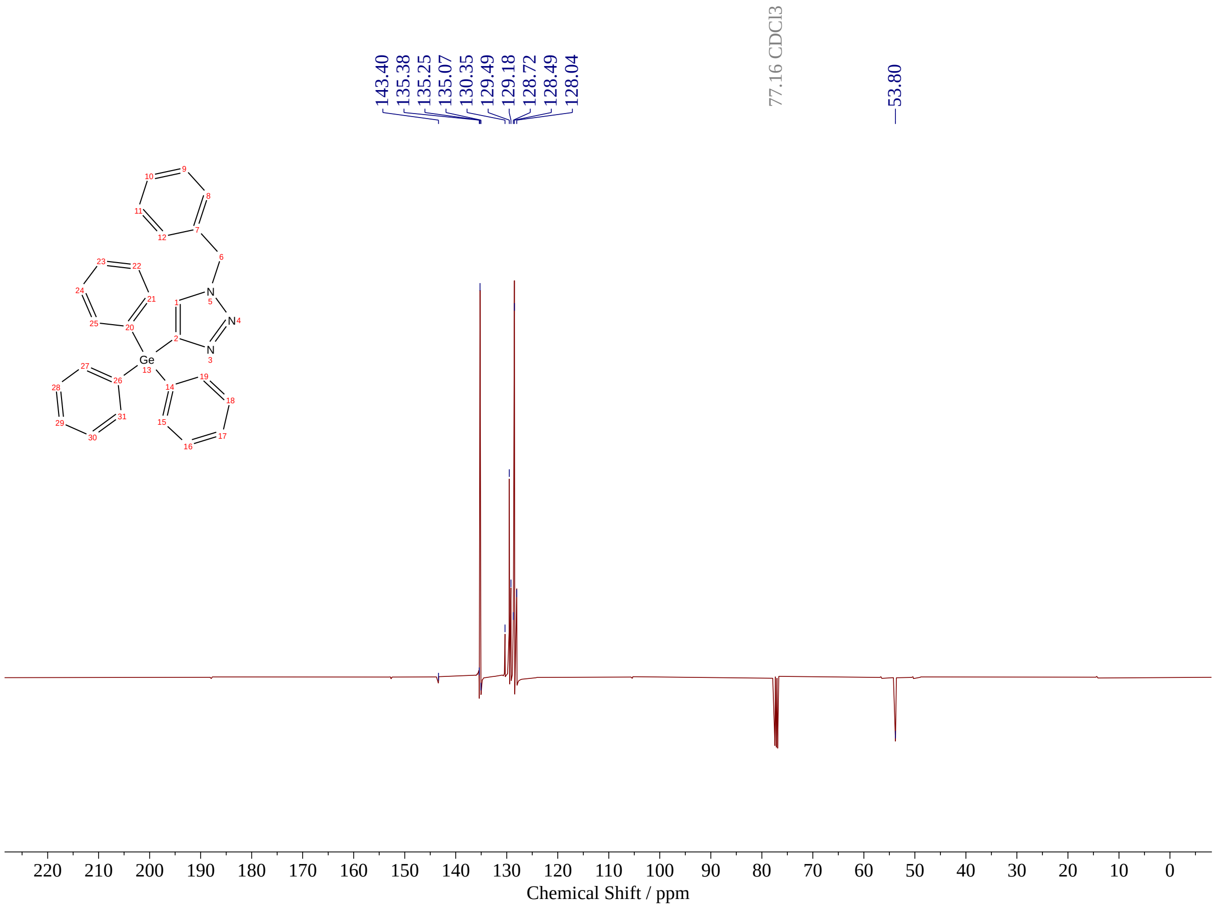

15 – ^{1}H

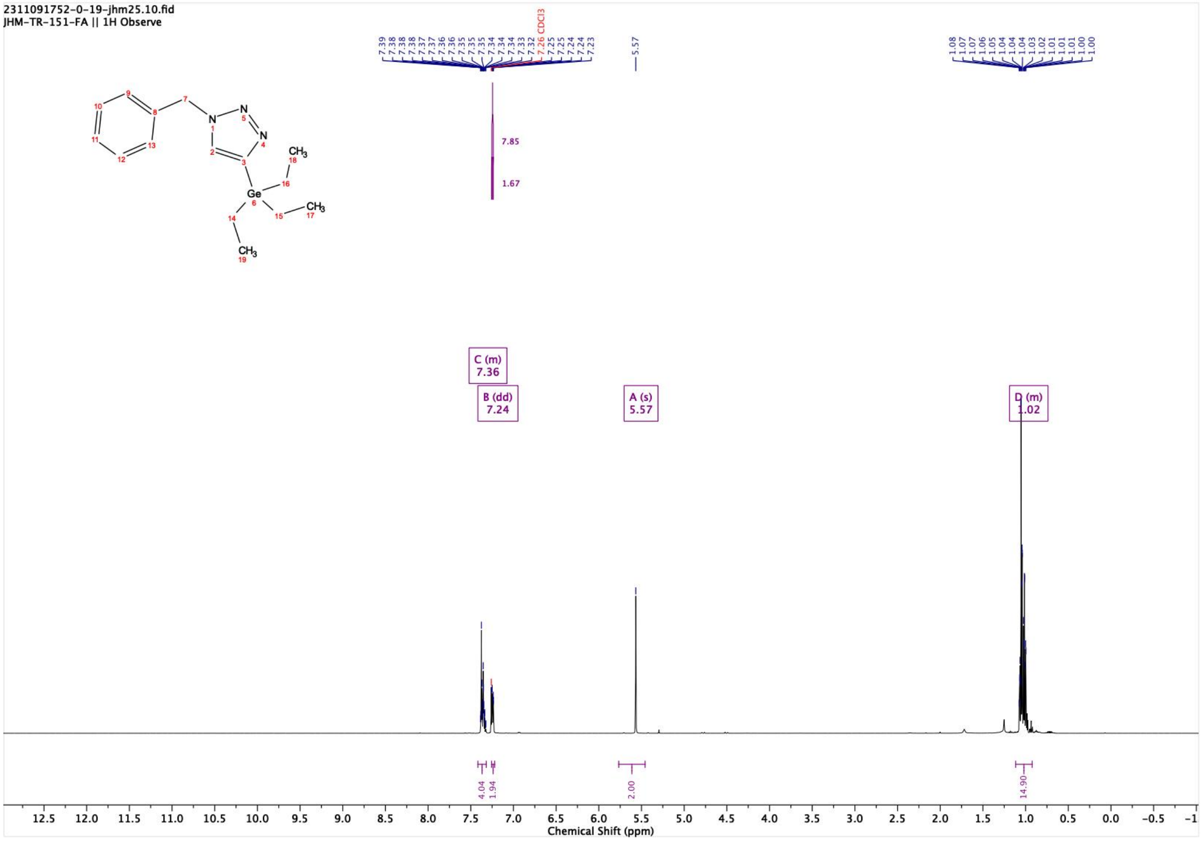

^{13}C DEPTQ

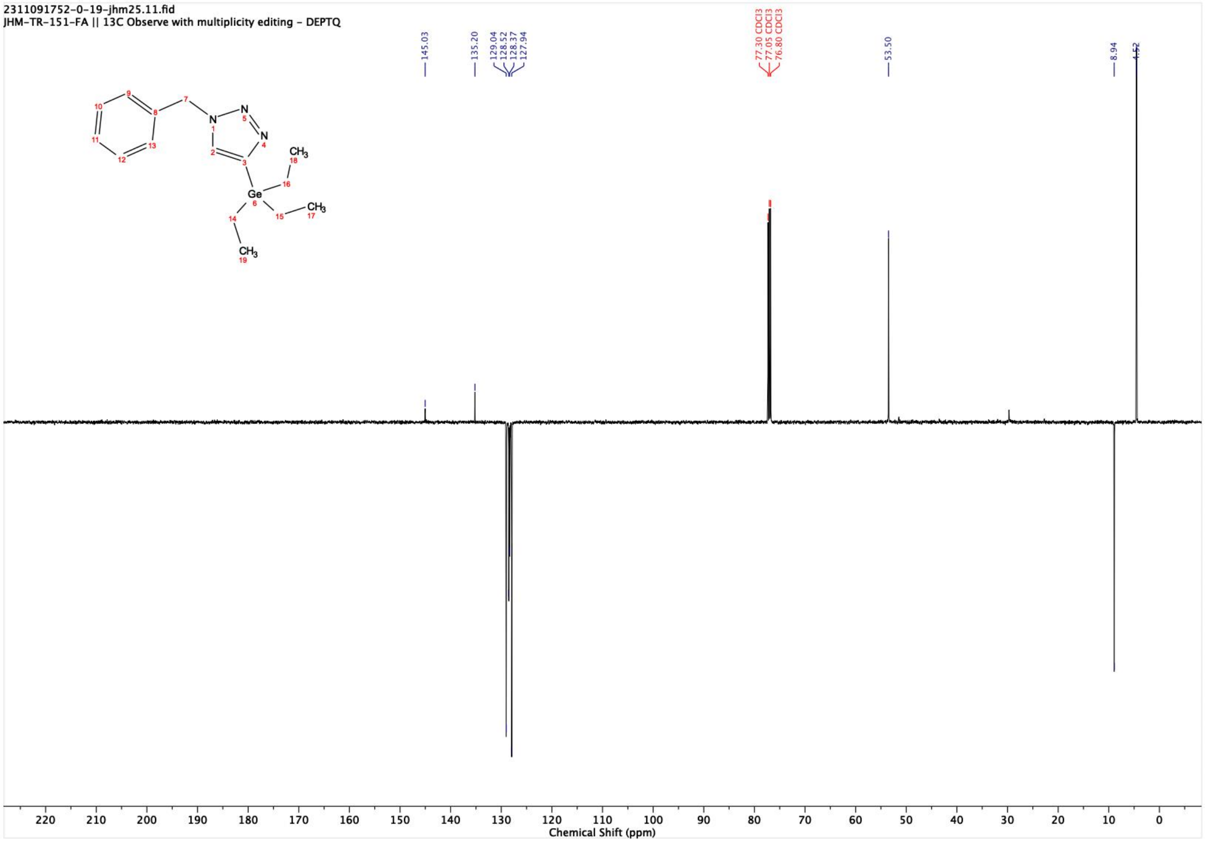

16 – ^{1}H

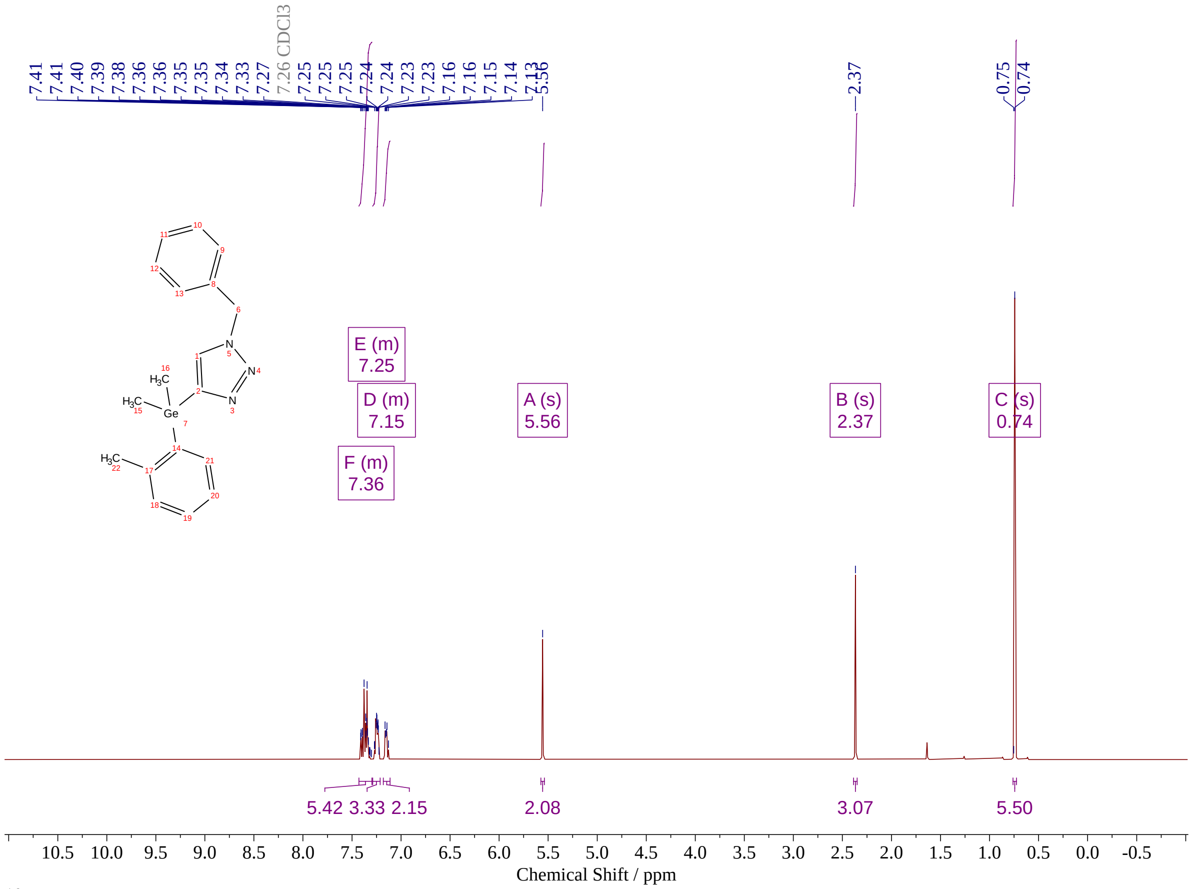

^{13}C DEPTQ

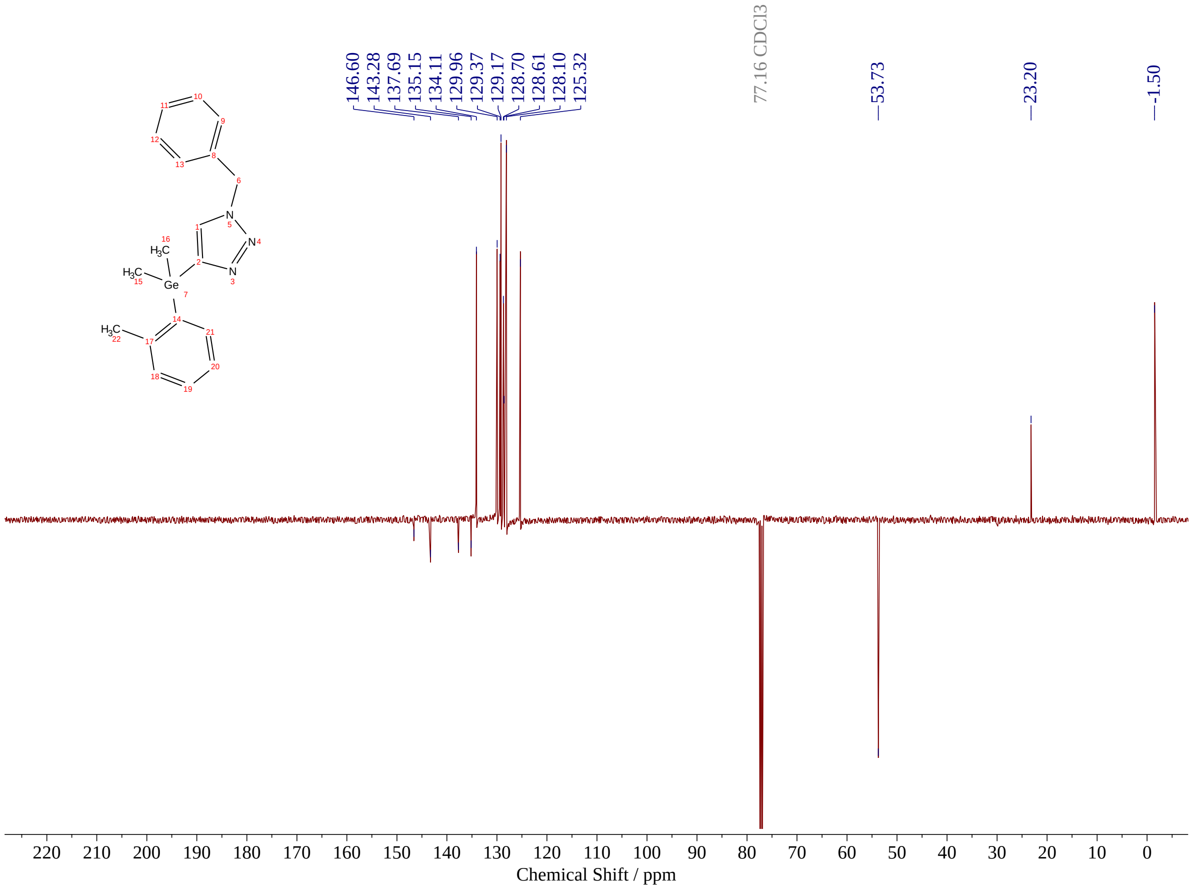

17 – ^{1}H

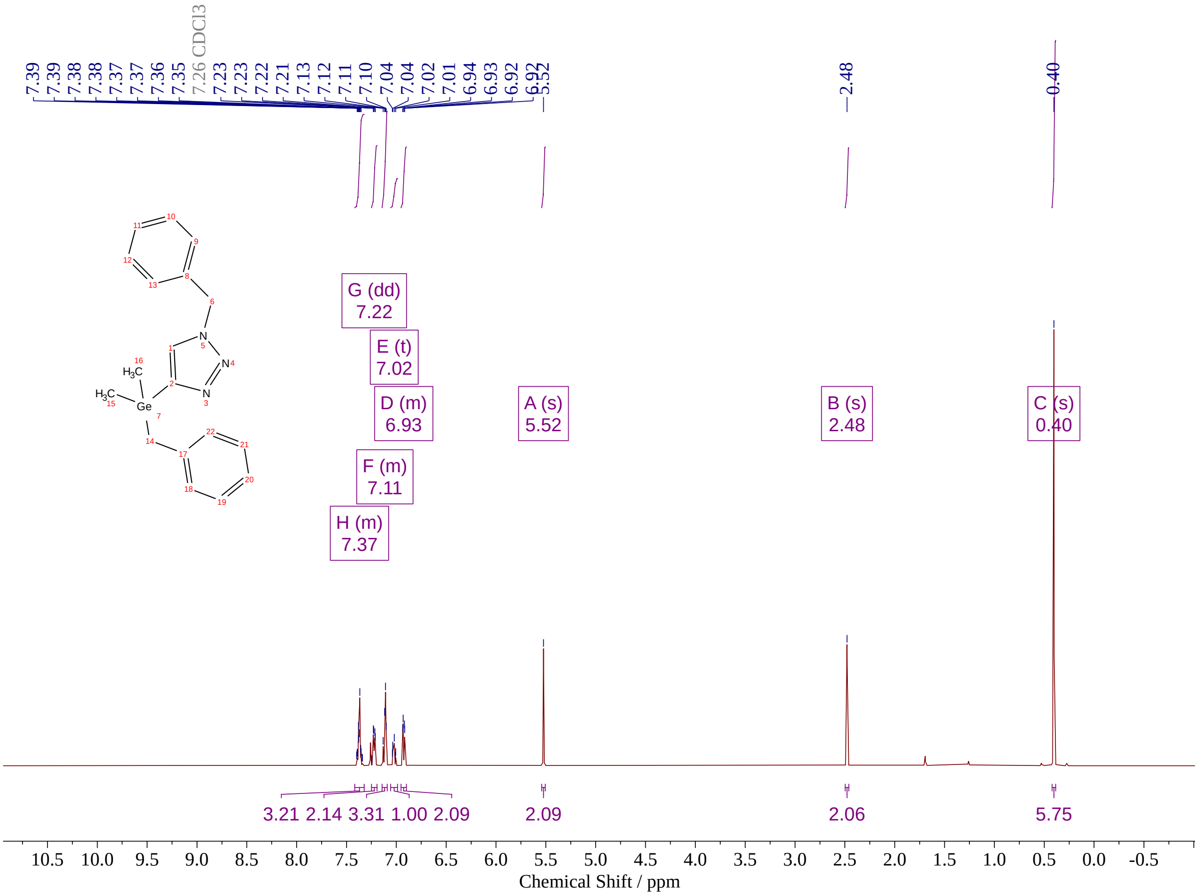

^{13}C DEPTQ

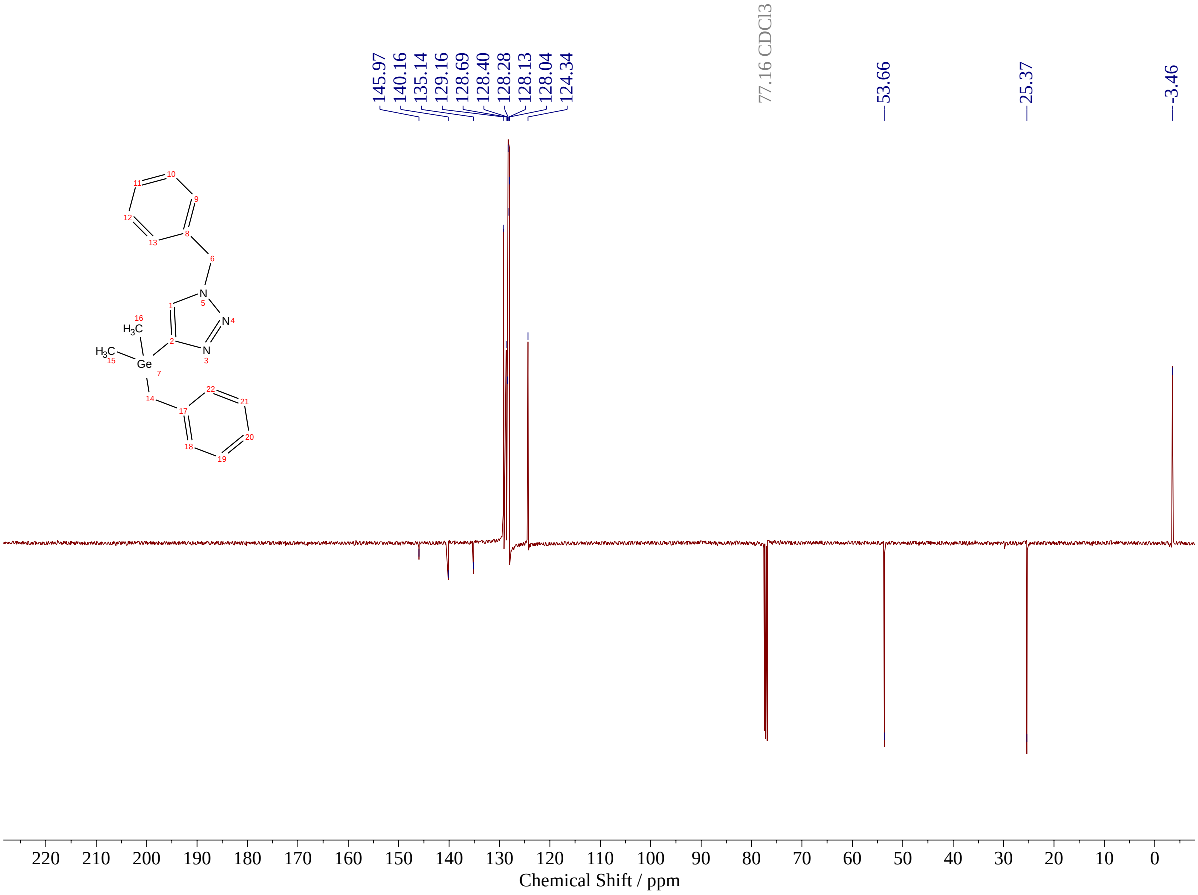

18 – ^{1}H

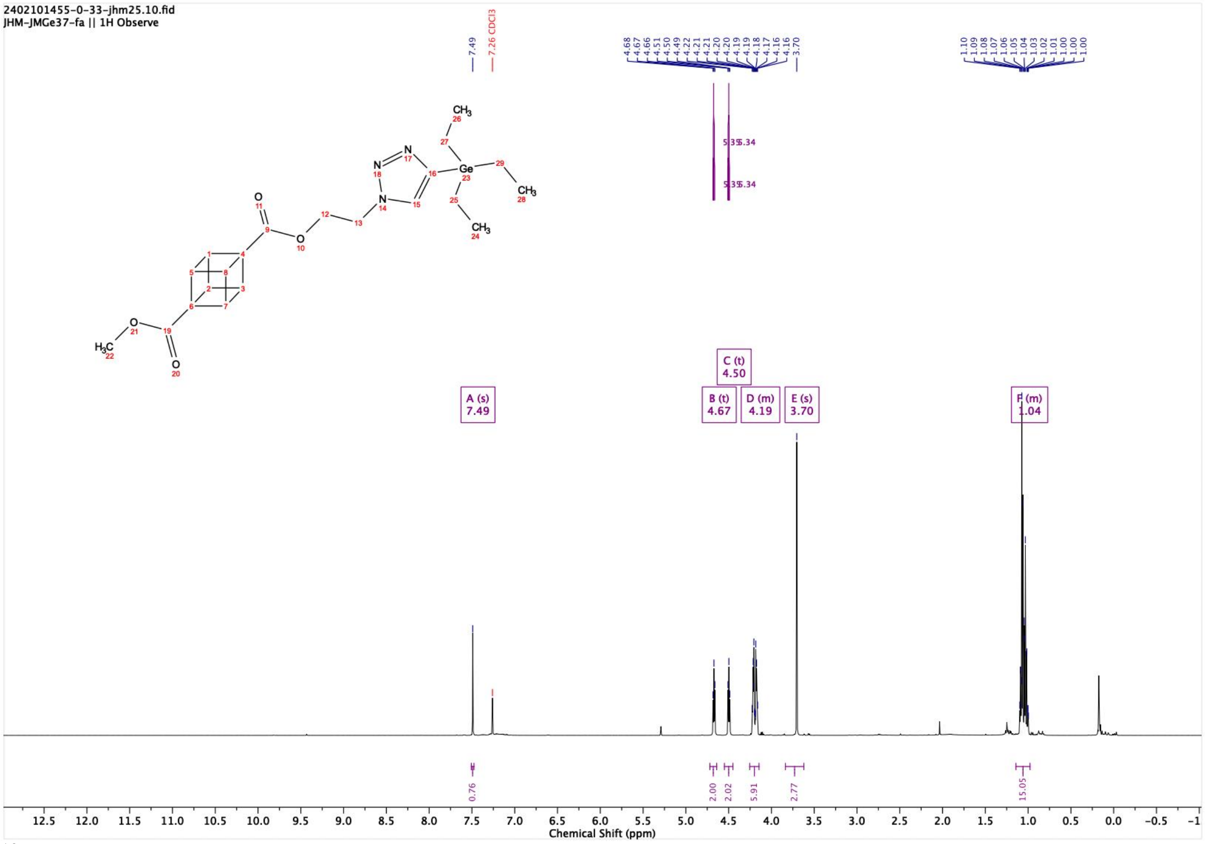

^{13}C DEPTQ

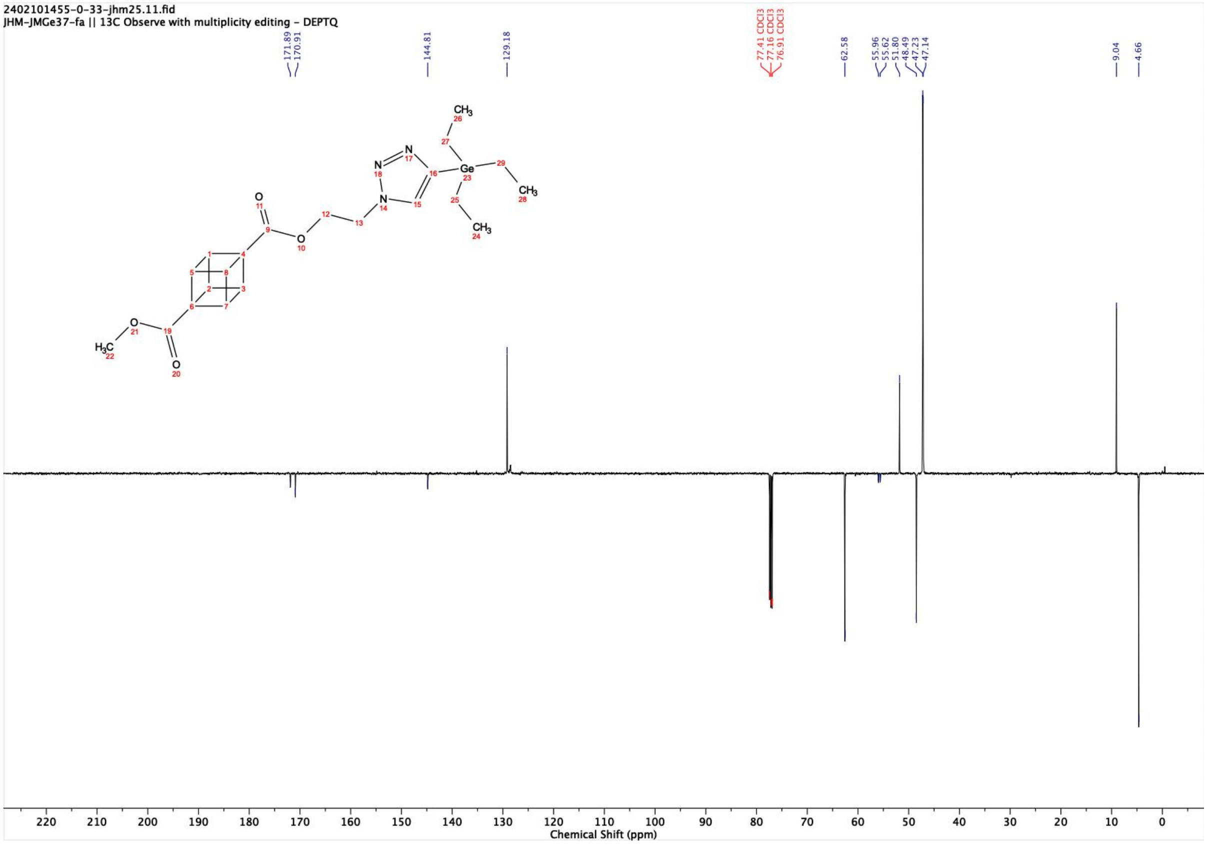

19 – ^{1}H

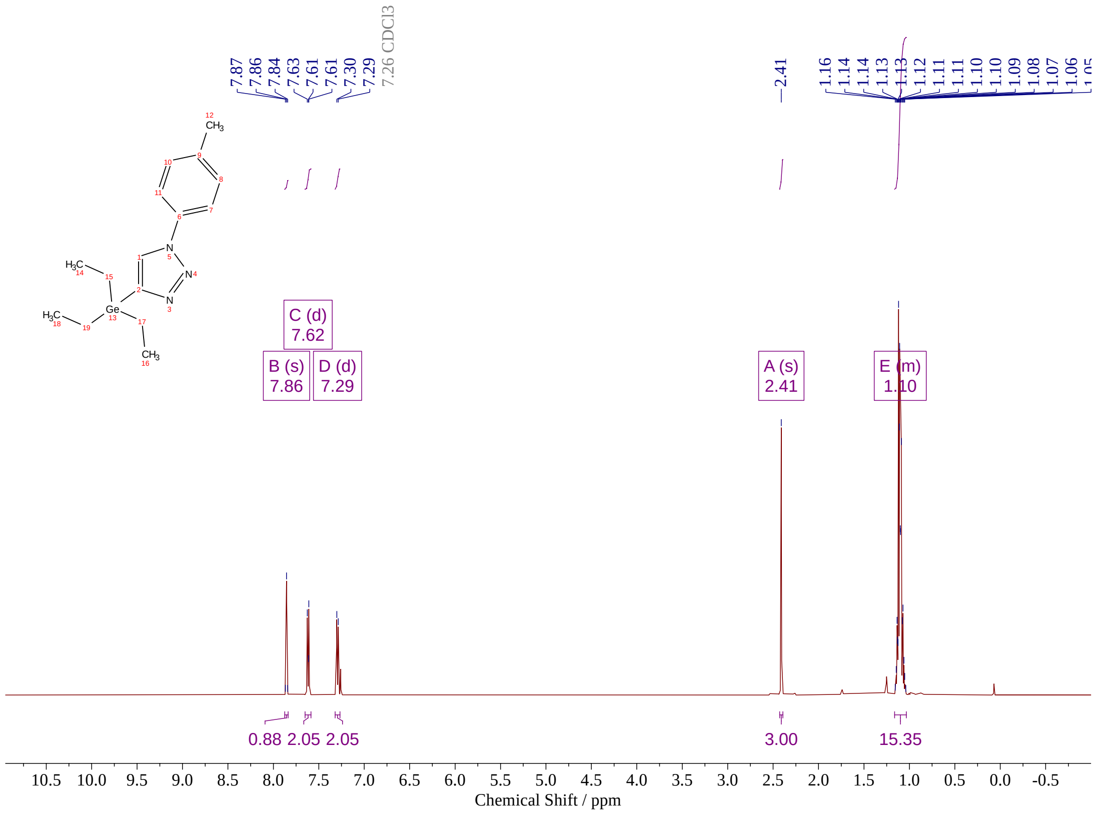

^{13}C DEPTQ

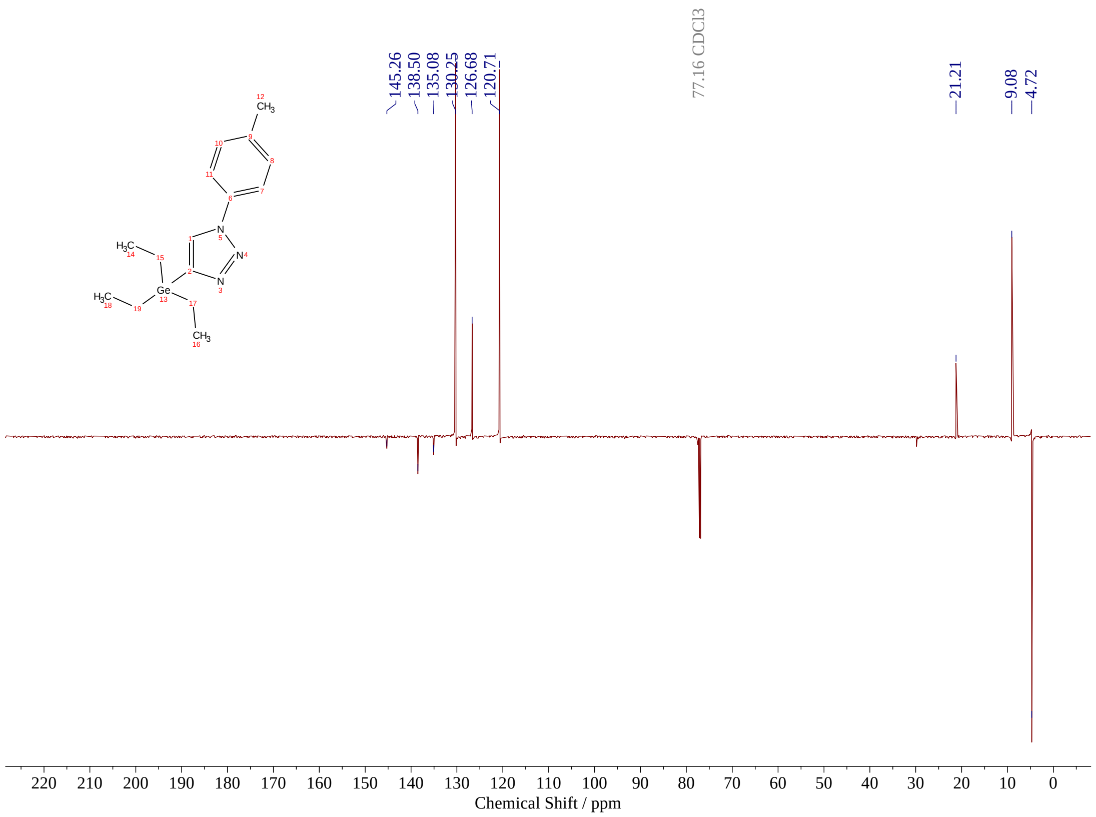

20 – ^{1}H

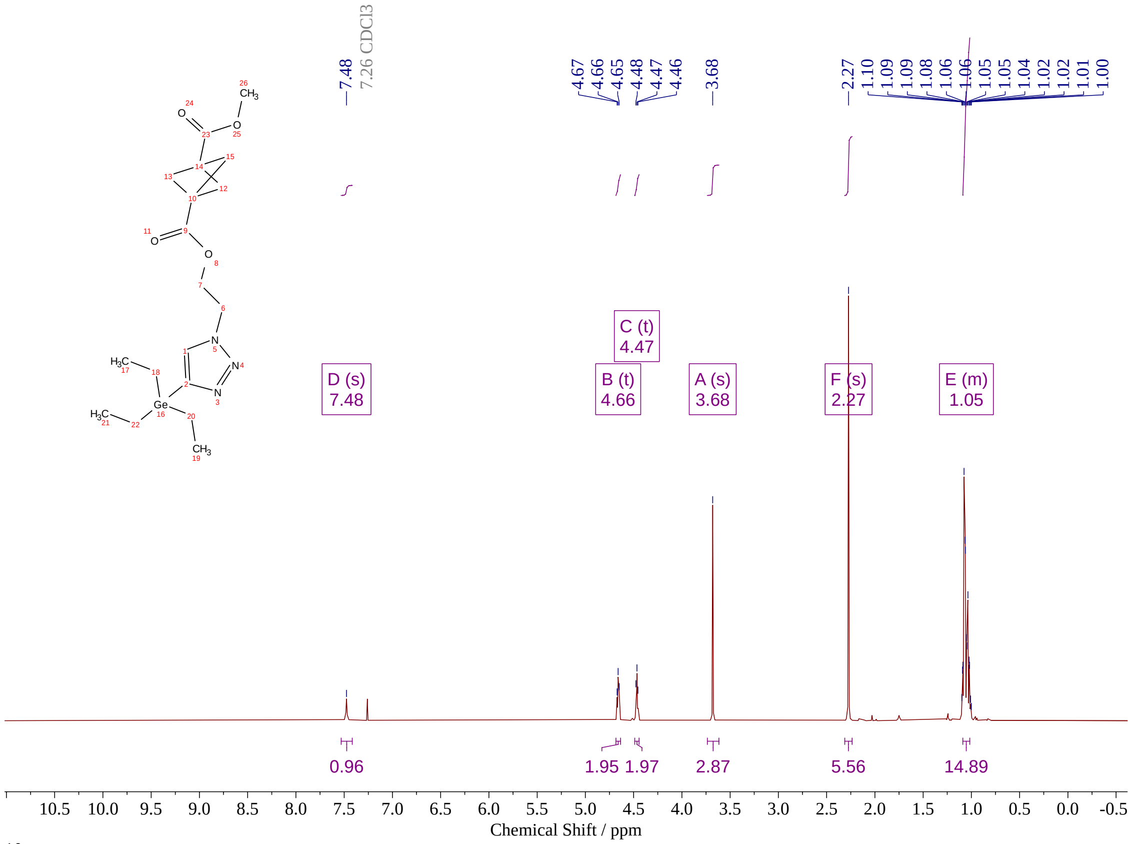

^{13}C DEPTQ

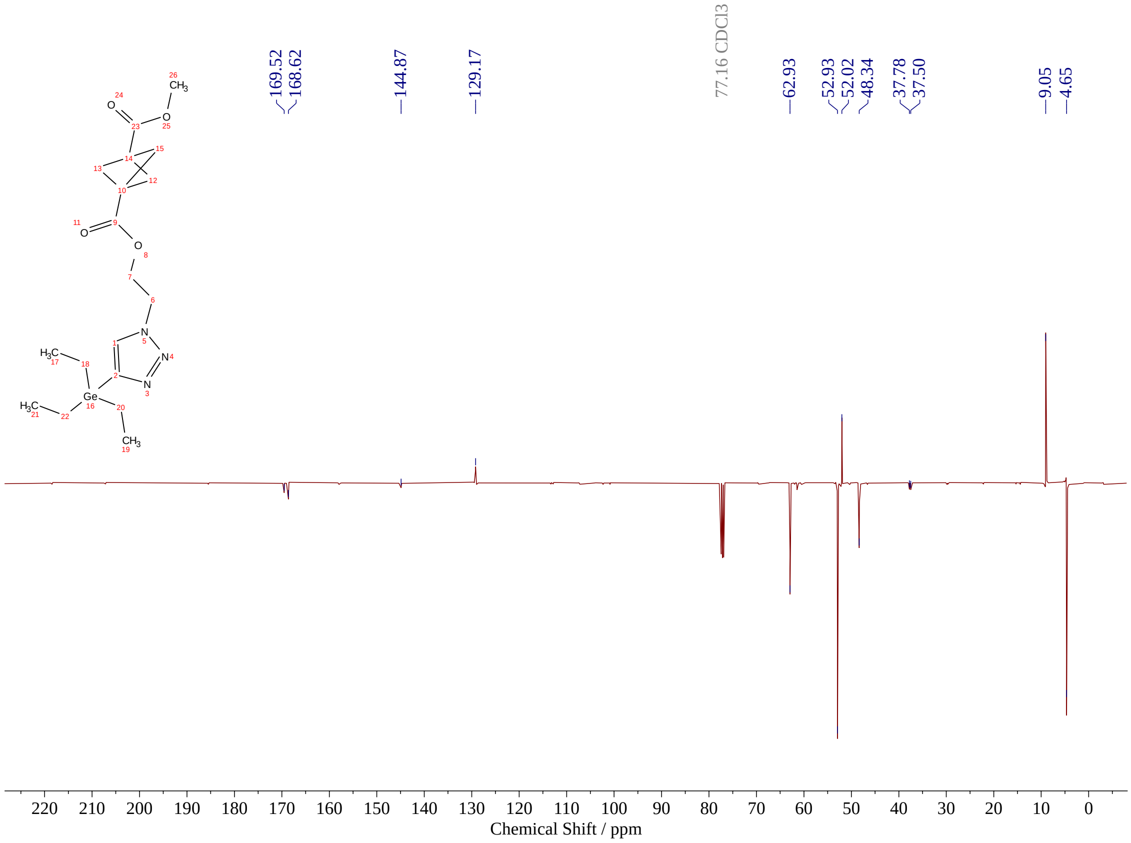

21 – ^{1}H

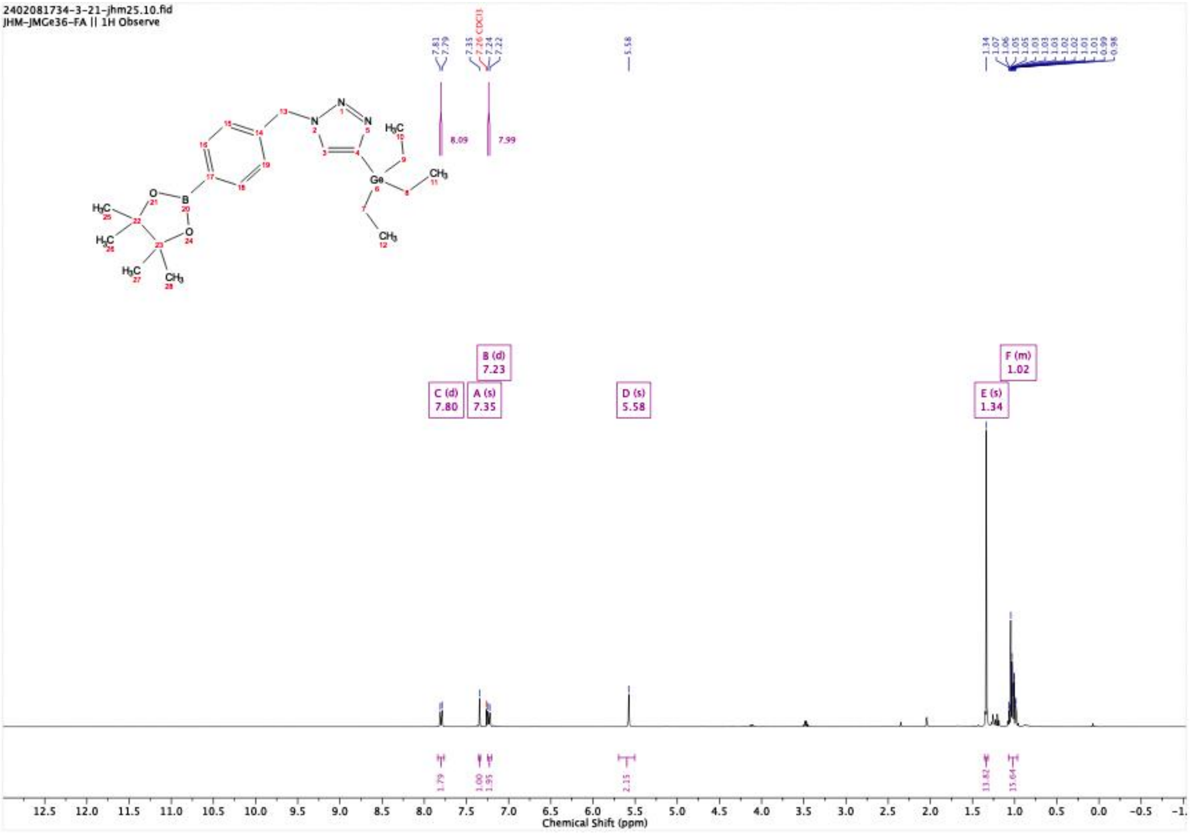

^{13}C DEPTQ

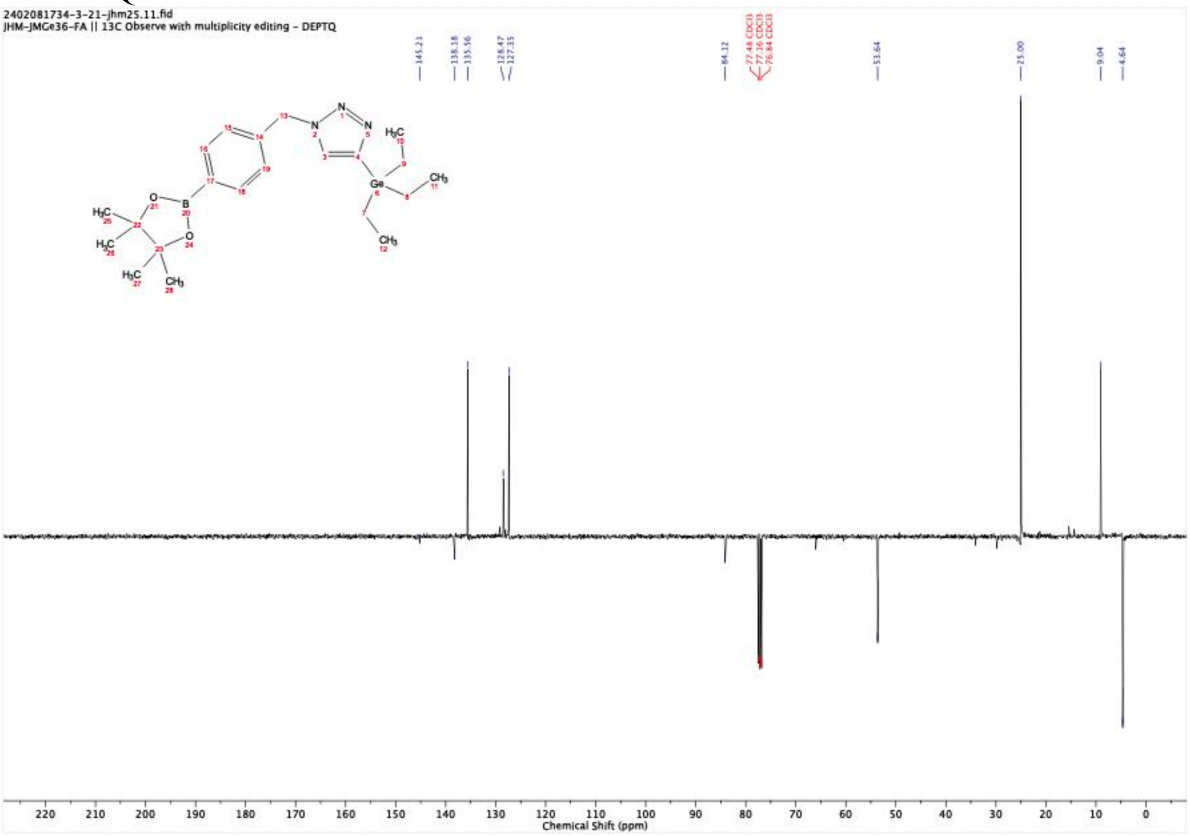

^{11}B

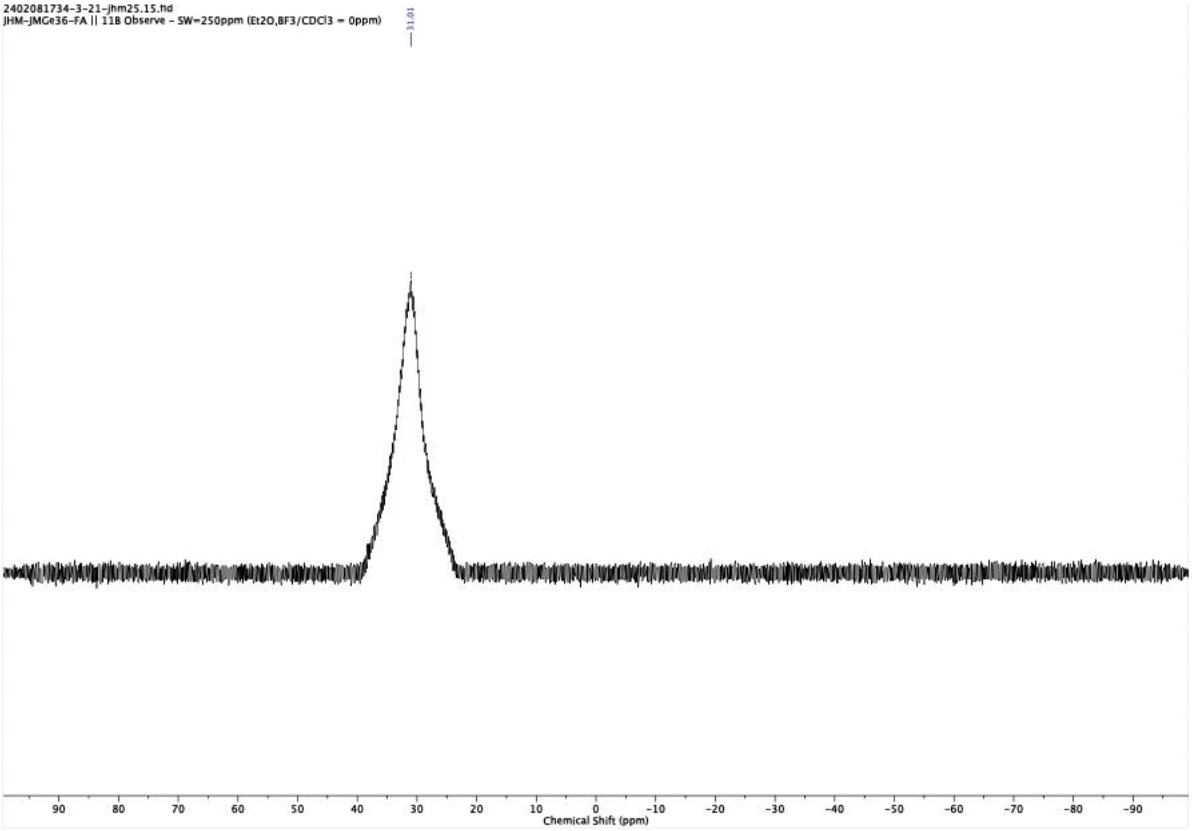

2402081734-3-21-jhm25.15.fid
JHM-JMGe36-FA || 11B Observe - SW=250ppm (Et2O,BF3/CDCl3 = 0ppm)
31.01
90
80
70
60
50
40
30
20
10
0
-10
-20
-30
-40
-50
-60
-70
-80
-90
Chemical Shift (ppm)

22 – ¹H

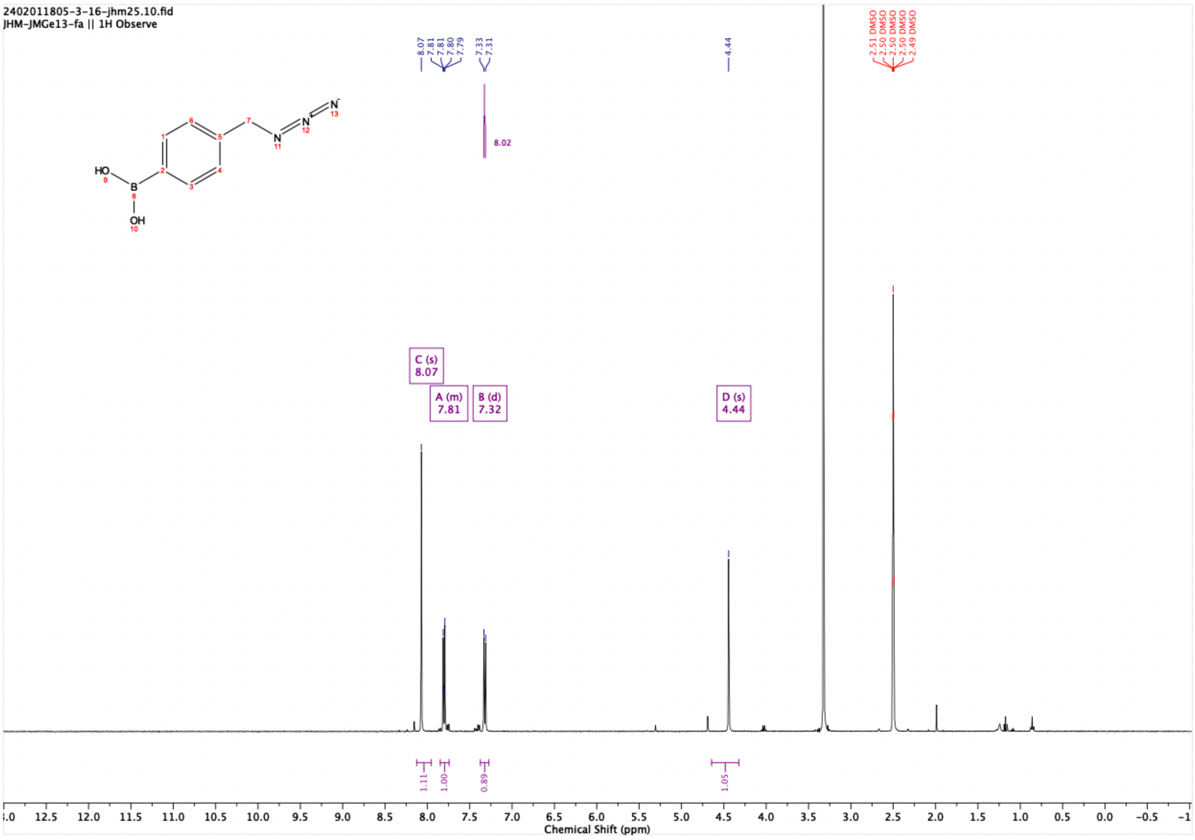

¹³C DEPTQ

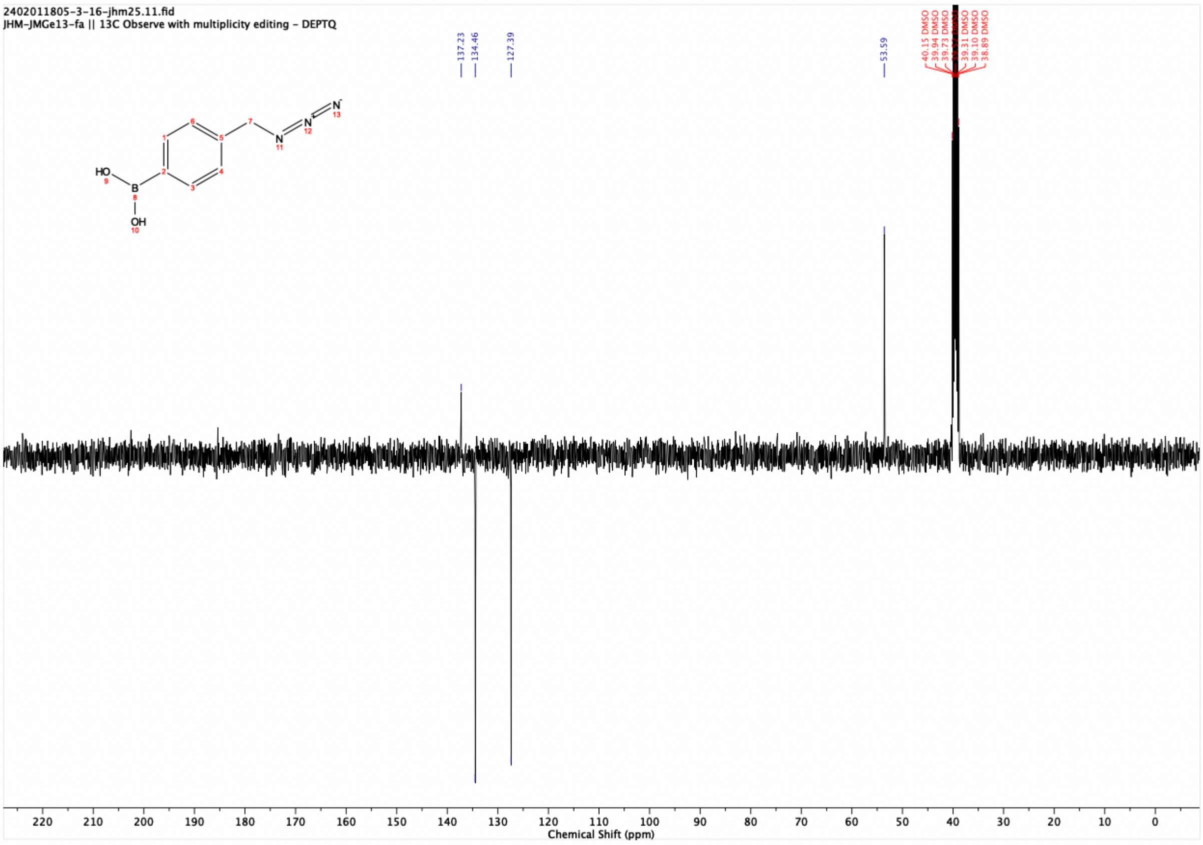

23 – ^{1}H

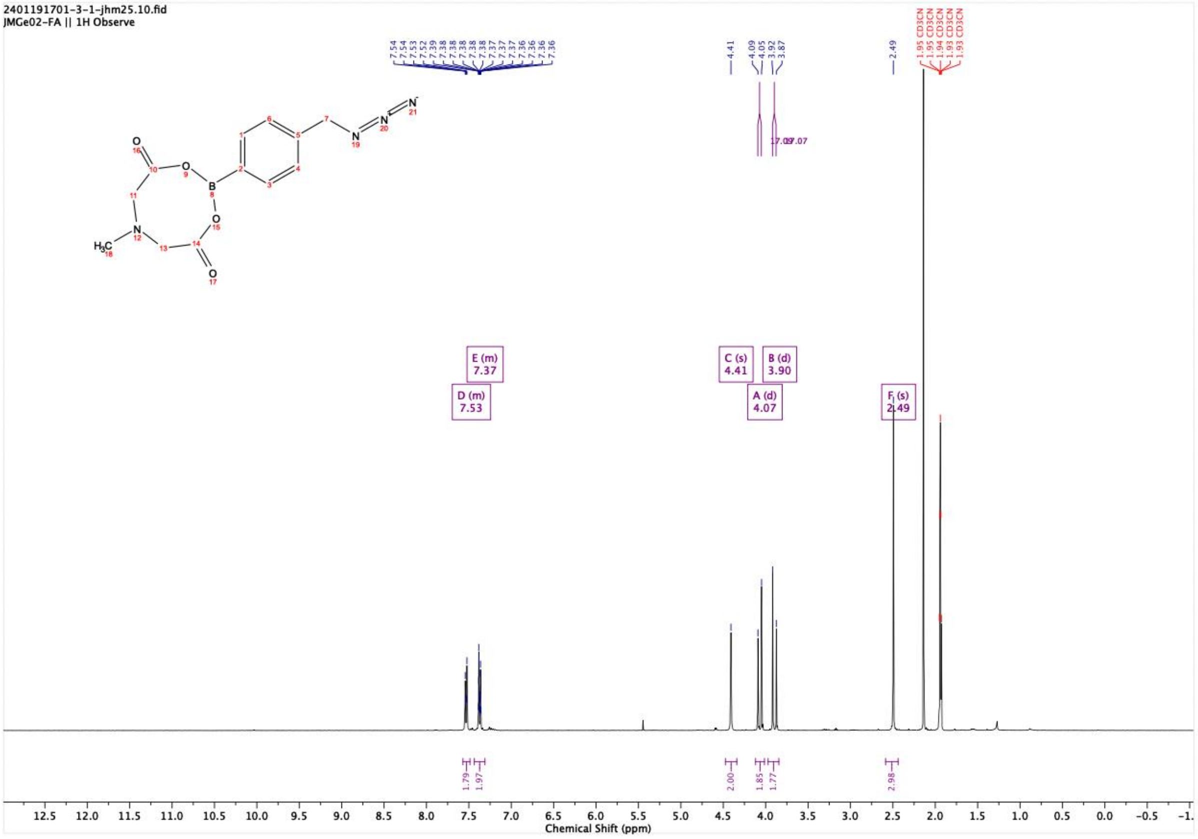

^{13}C DEPTQ

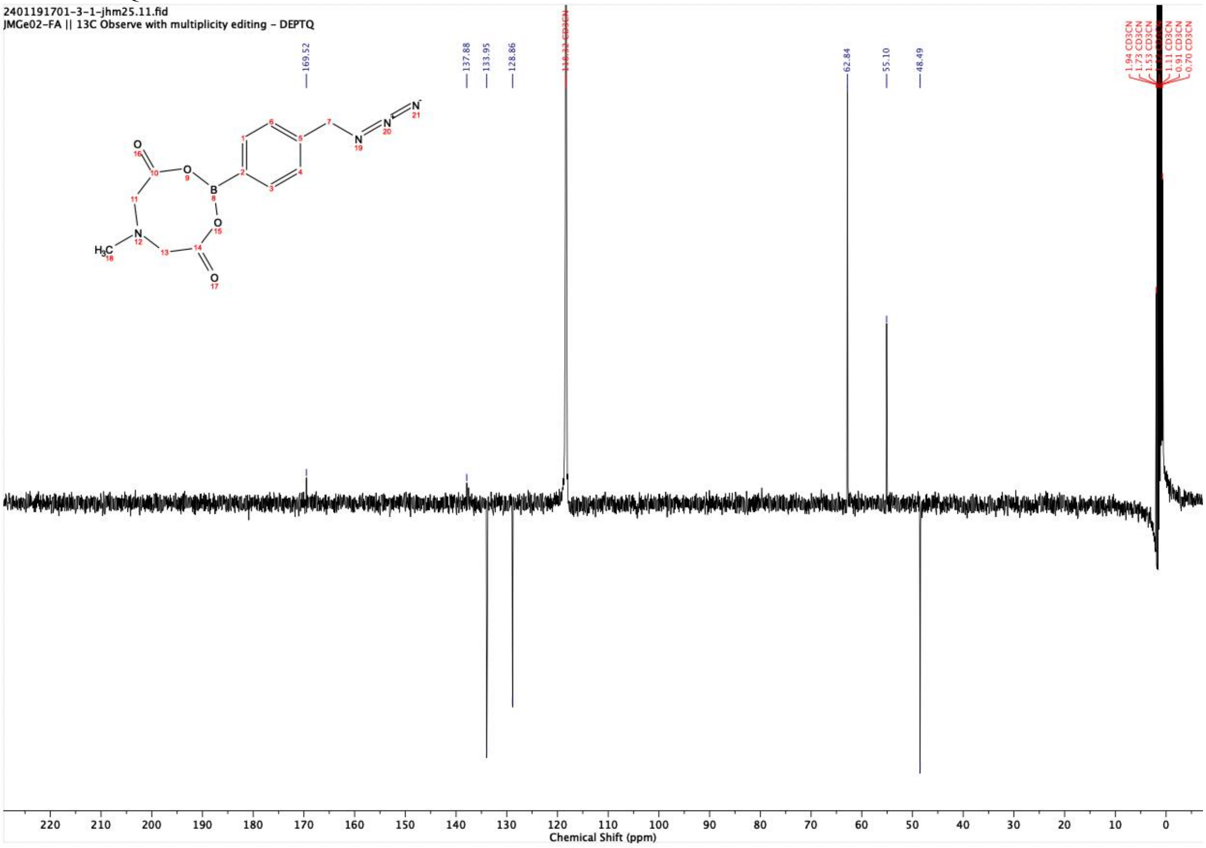

^{11}B

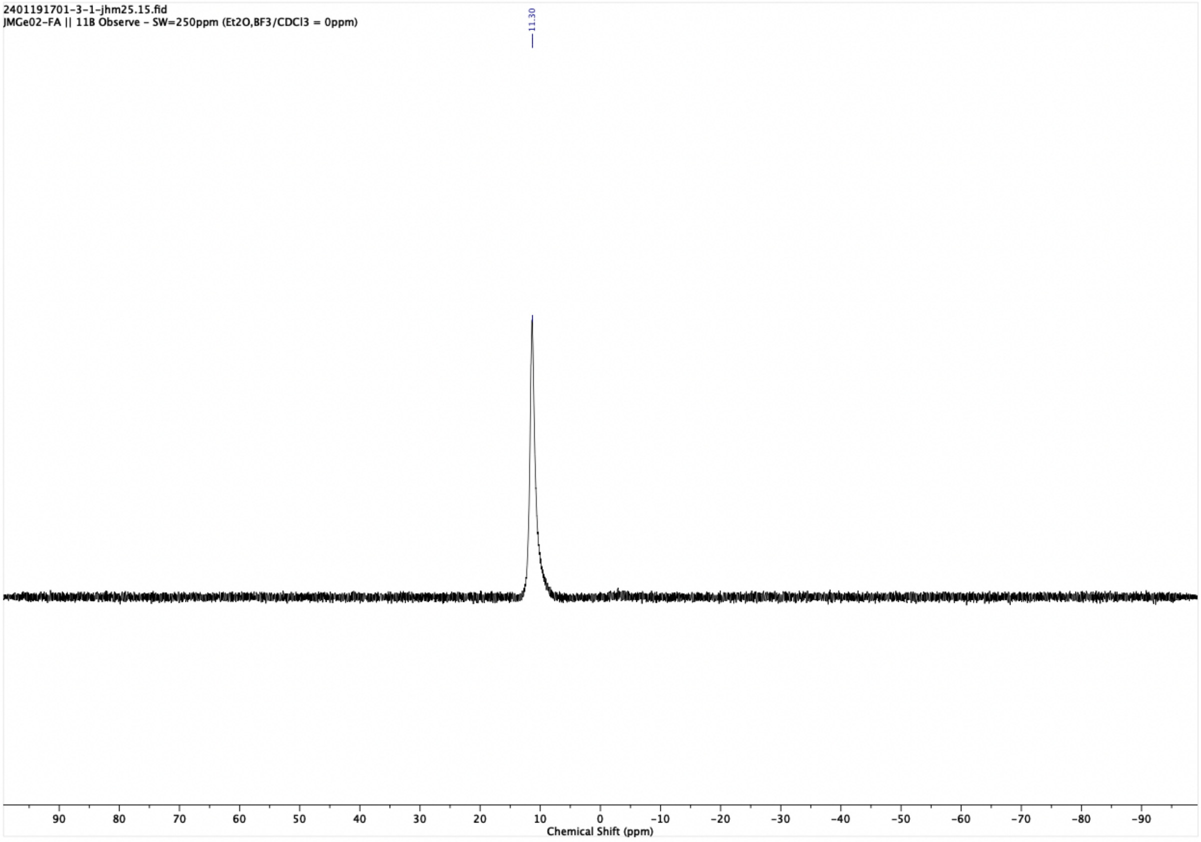

2401191701-3-1-jhm25.15.fid
JMGe02-FA || 11B Observe - SW=250ppm (Et2O,BF3/CDCl3 = 0ppm)
11.30
90 80 70 60 50 40 30 20 10 0 -10 -20 -30 -40 -50 -60 -70 -80 -90
Chemical Shift (ppm)

24 – ^{1}H

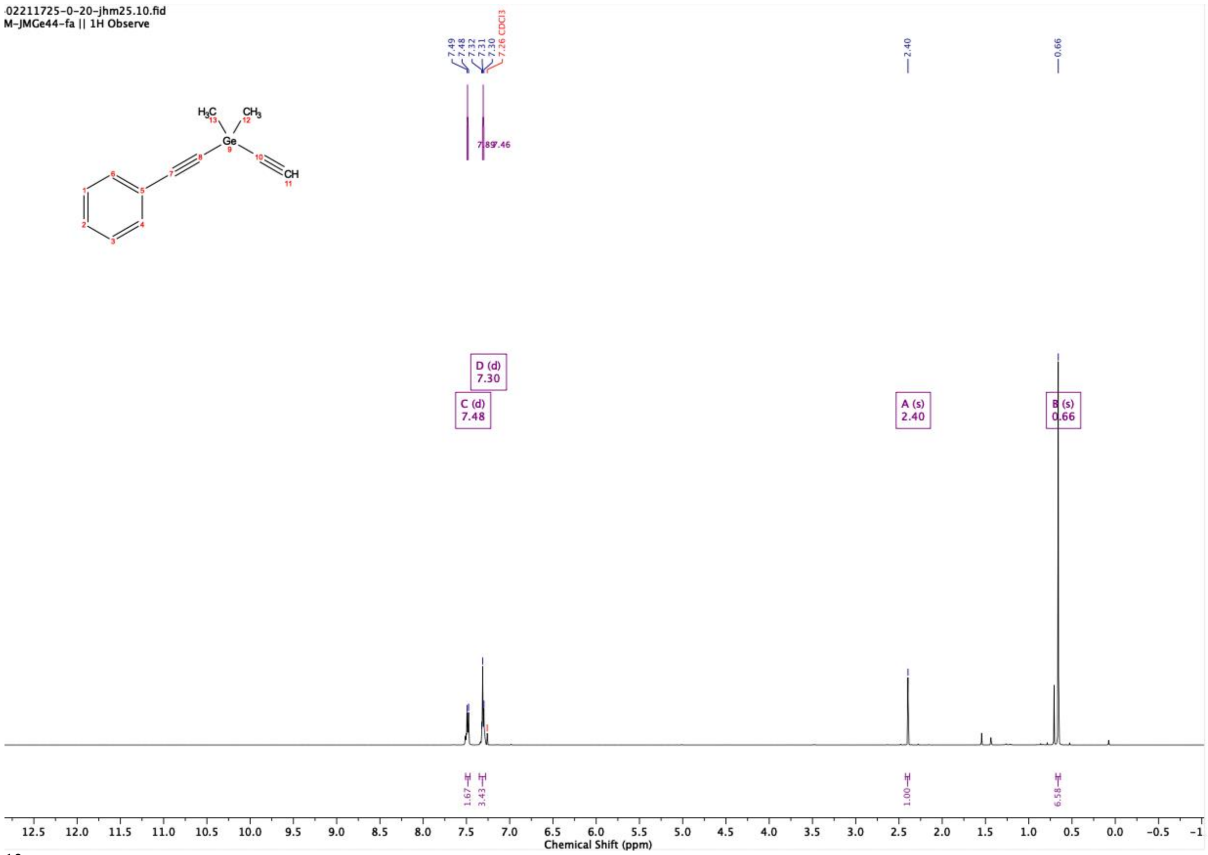

^{13}C DEPTQ

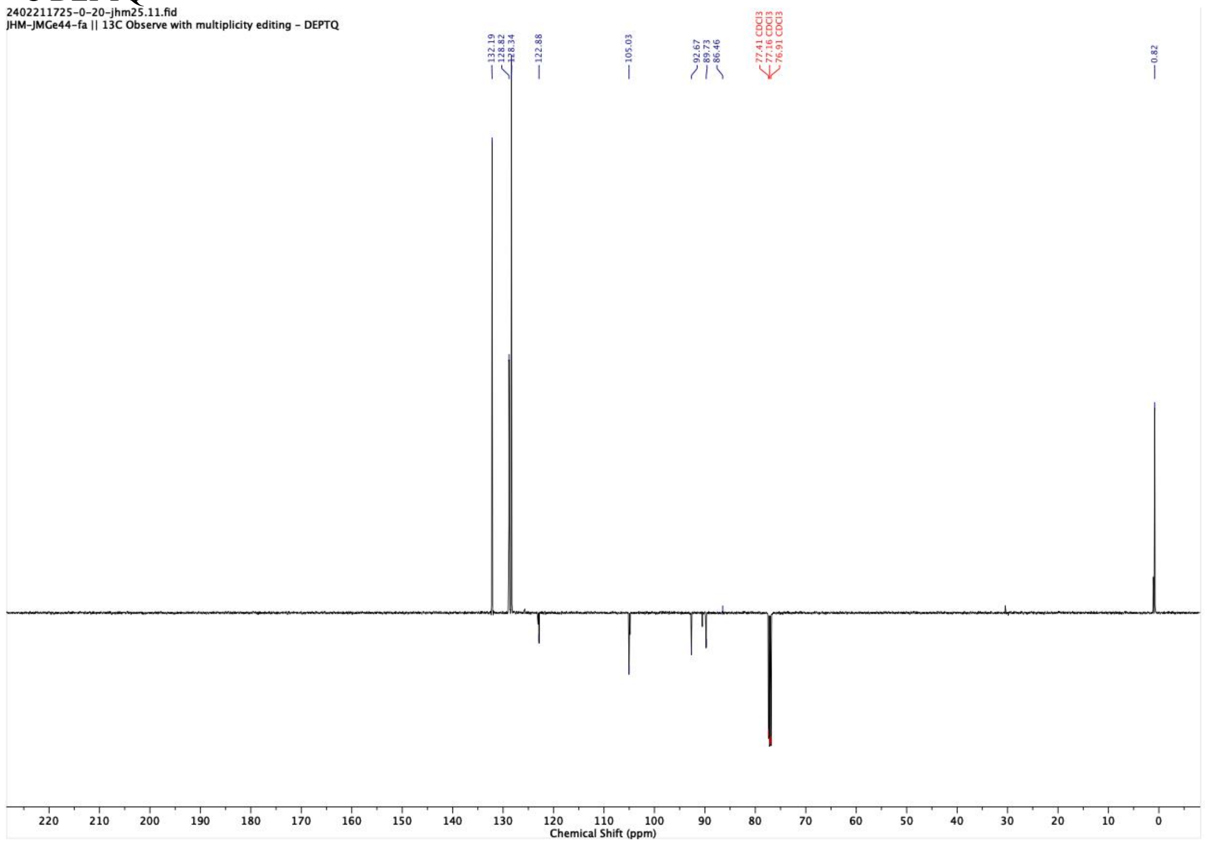

25 – ^{1}H

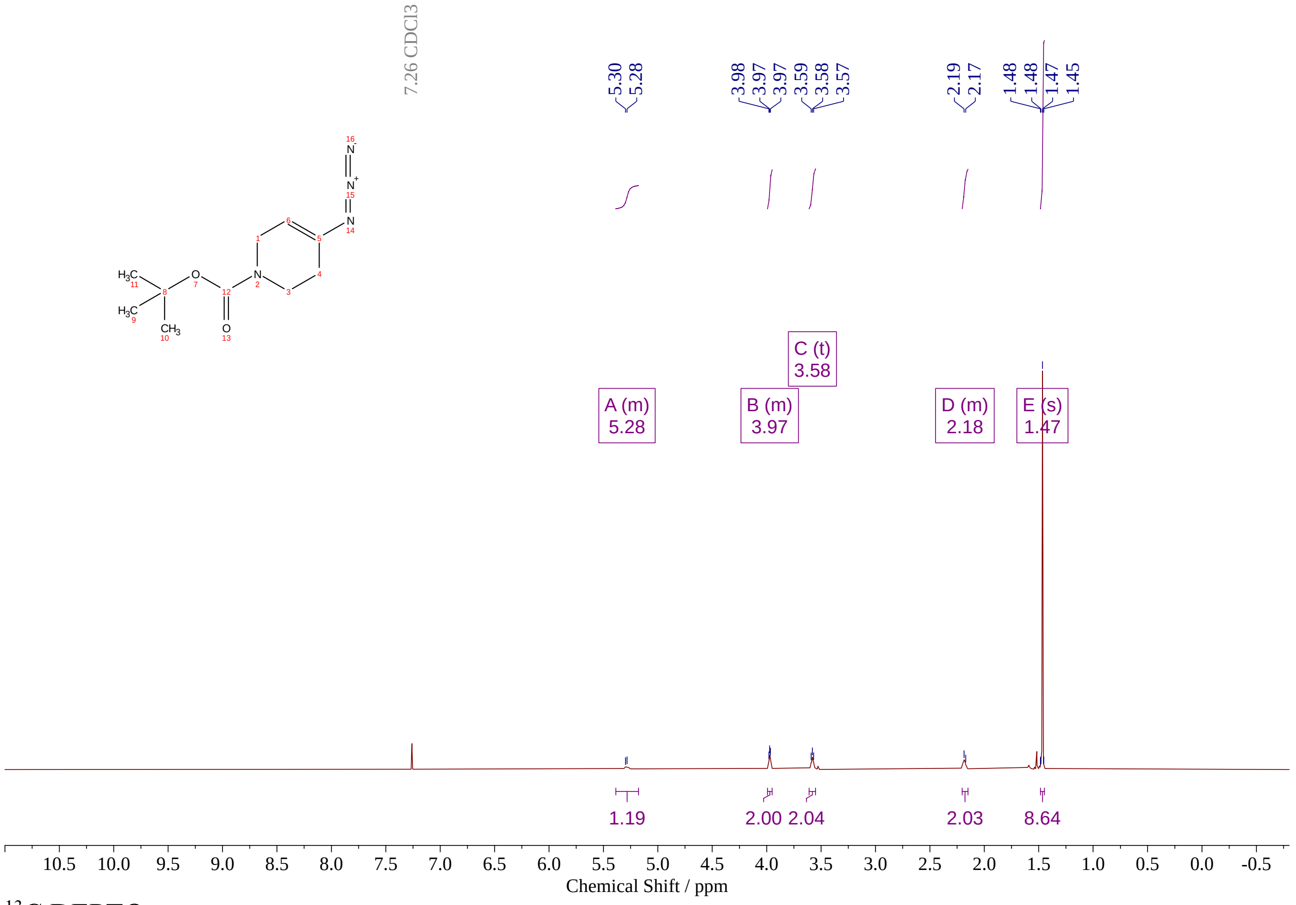

^{13}C DEPTQ

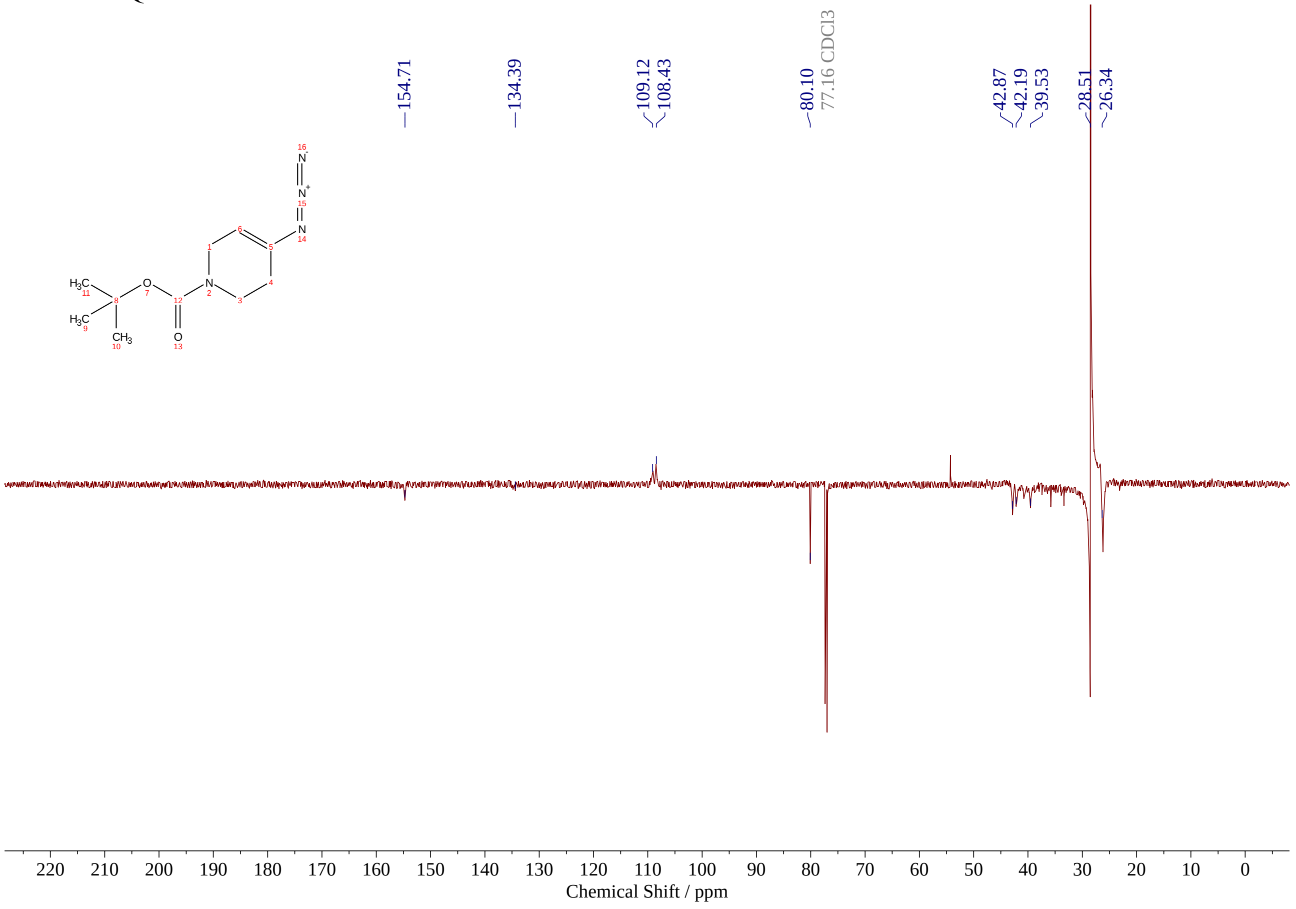

26 – ^{1}H

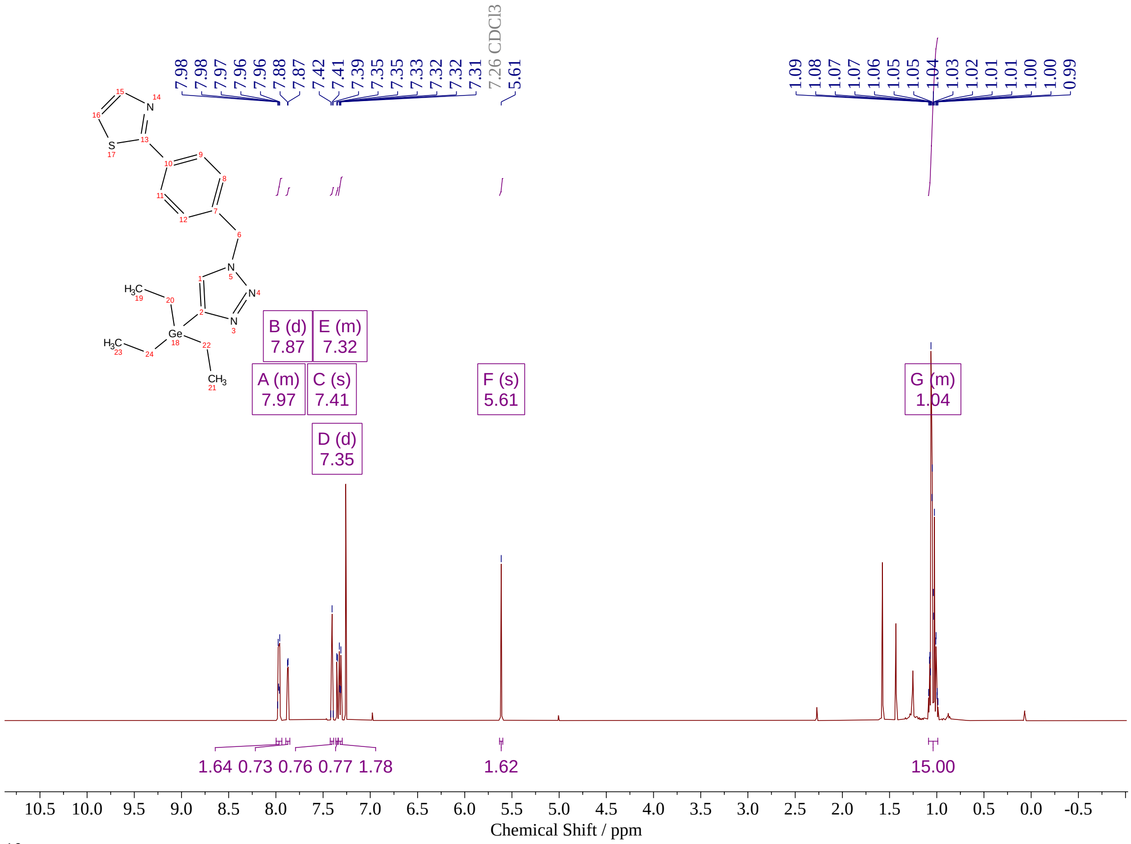

^{13}C DEPTQ

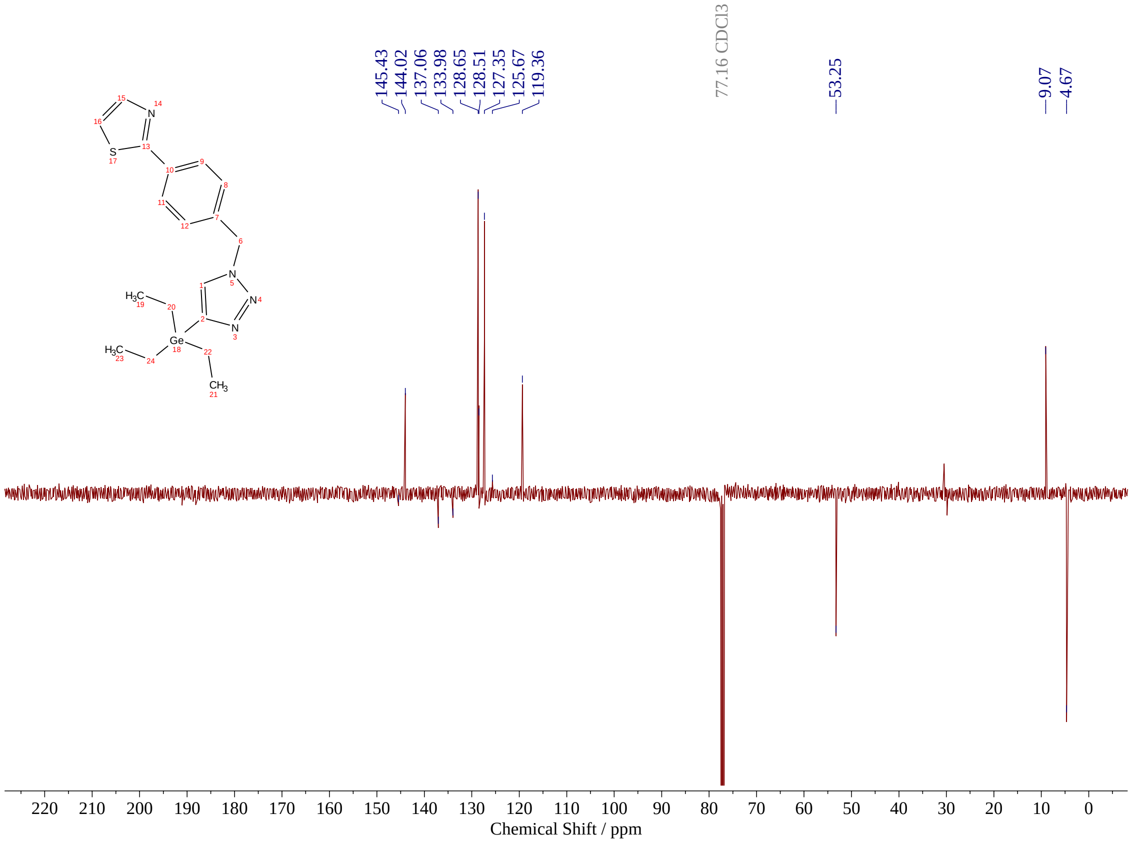

27 – ^{1}H

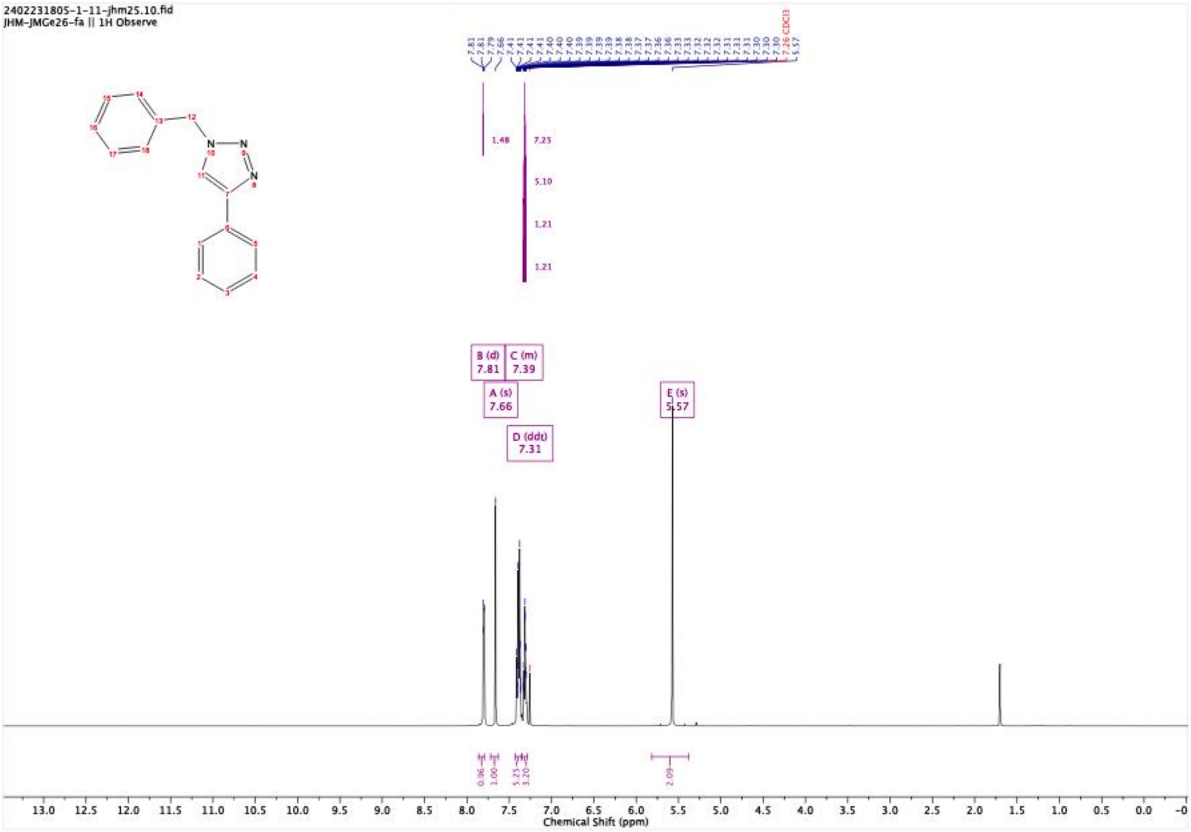

^{13}C DEPTQ

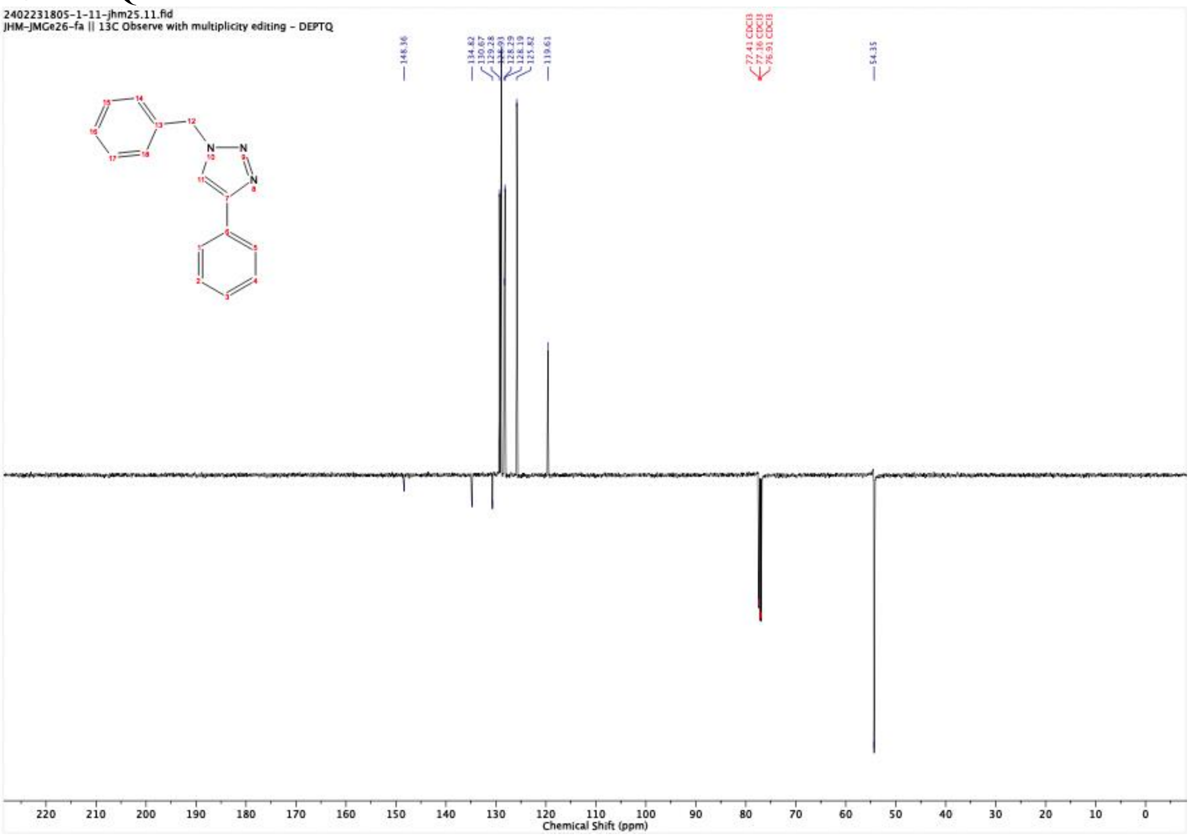

28 – ^{1}H

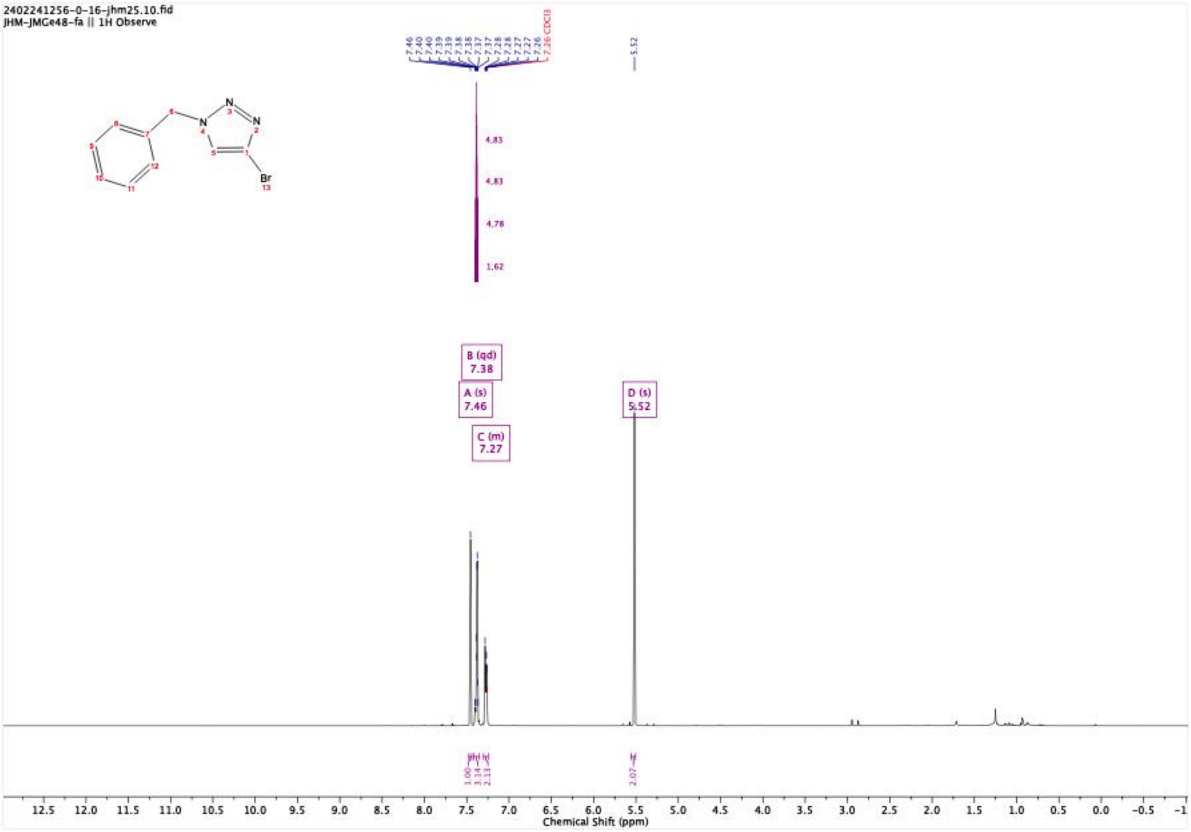

^{13}C DEPTQ

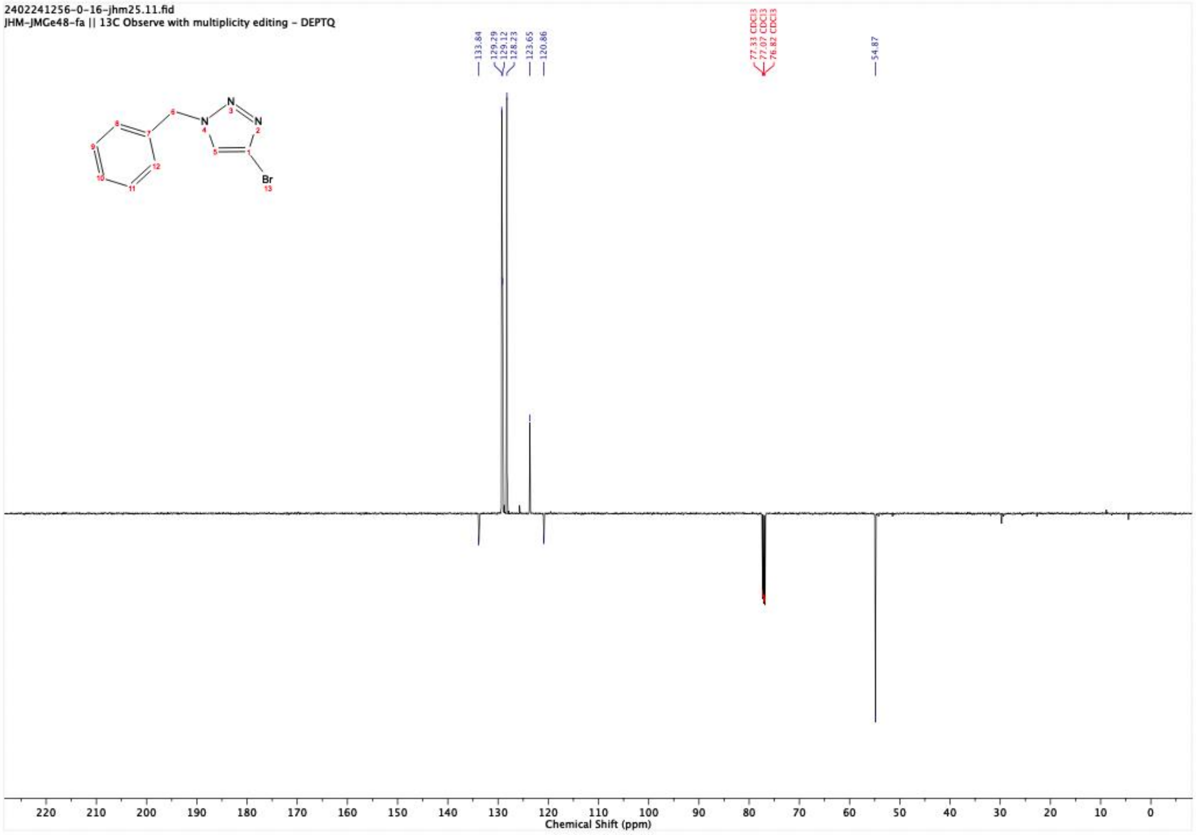

29 – ^{1}H

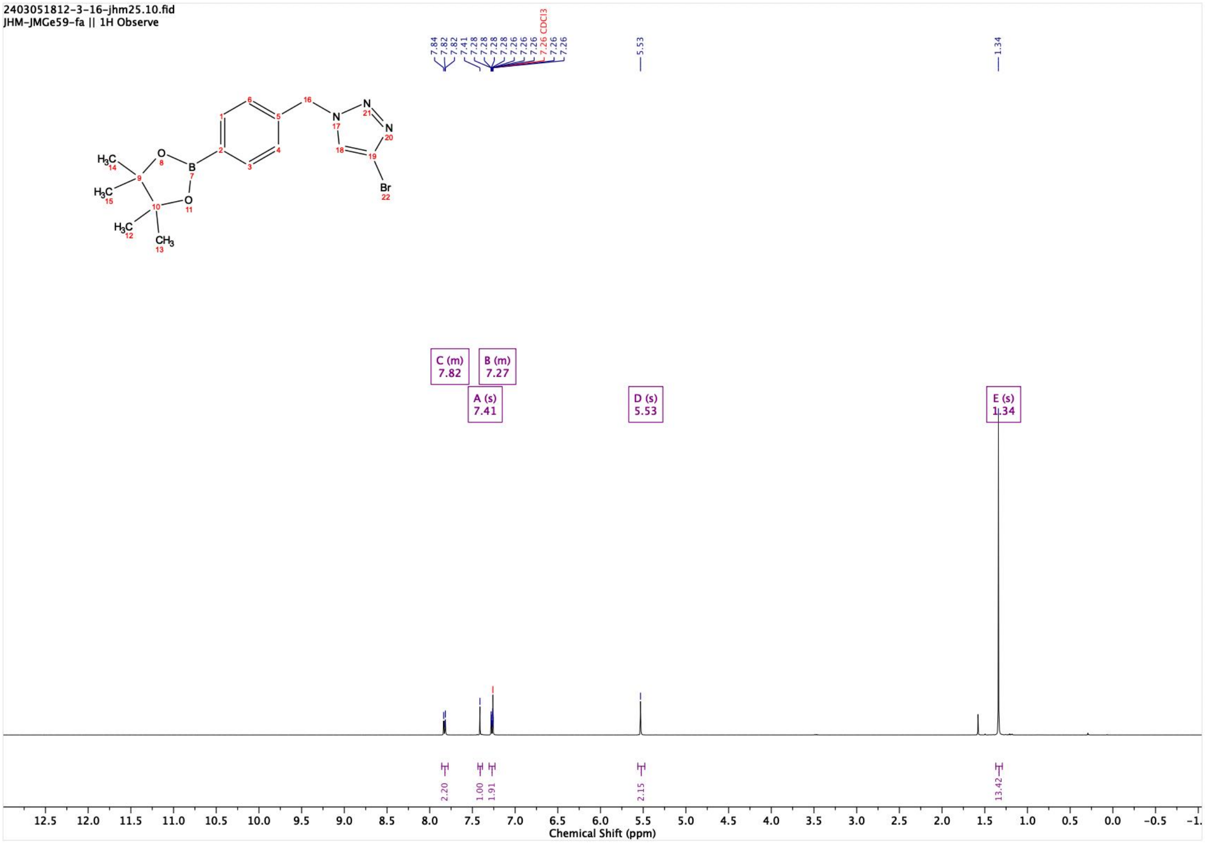

^{13}C DEPTQ

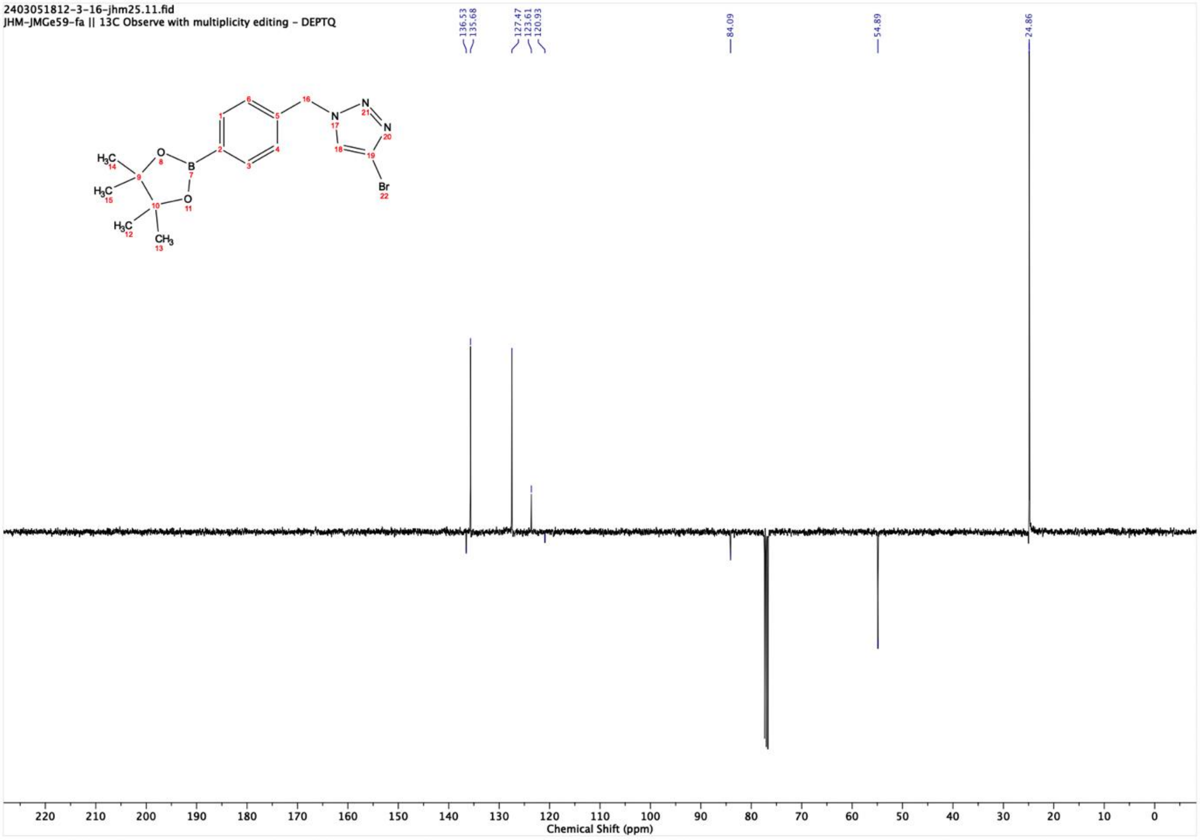

^{11}B

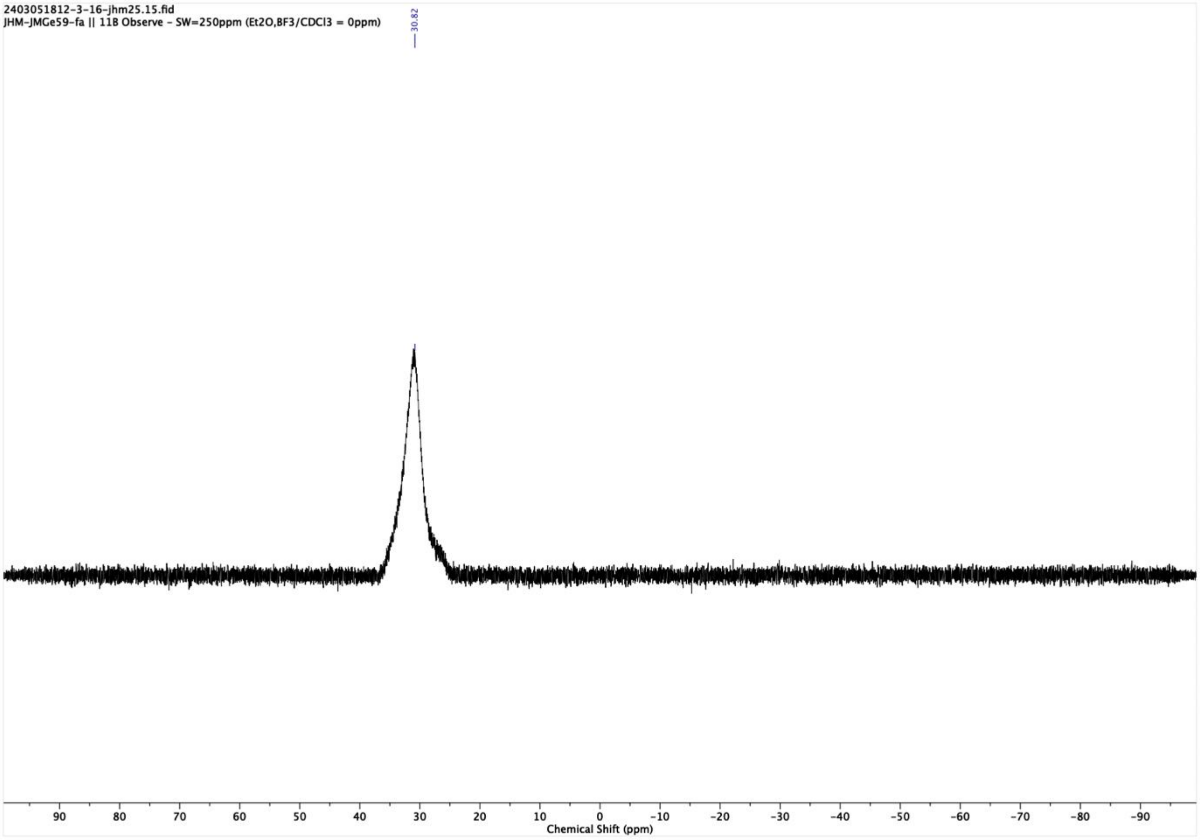

2403051812-3-16-jhm25.15.fid
JHM-JMGe59-fa || 11B Observe - SW=250ppm (Et2O,BF3/CDCl3 = 0ppm)
30.82
90
80
70
60
50
40
30
20
10
0
-10
-20
-30
-40
-50
-60
-70
-80
-90
Chemical Shift (ppm)

30 – ^{1}H

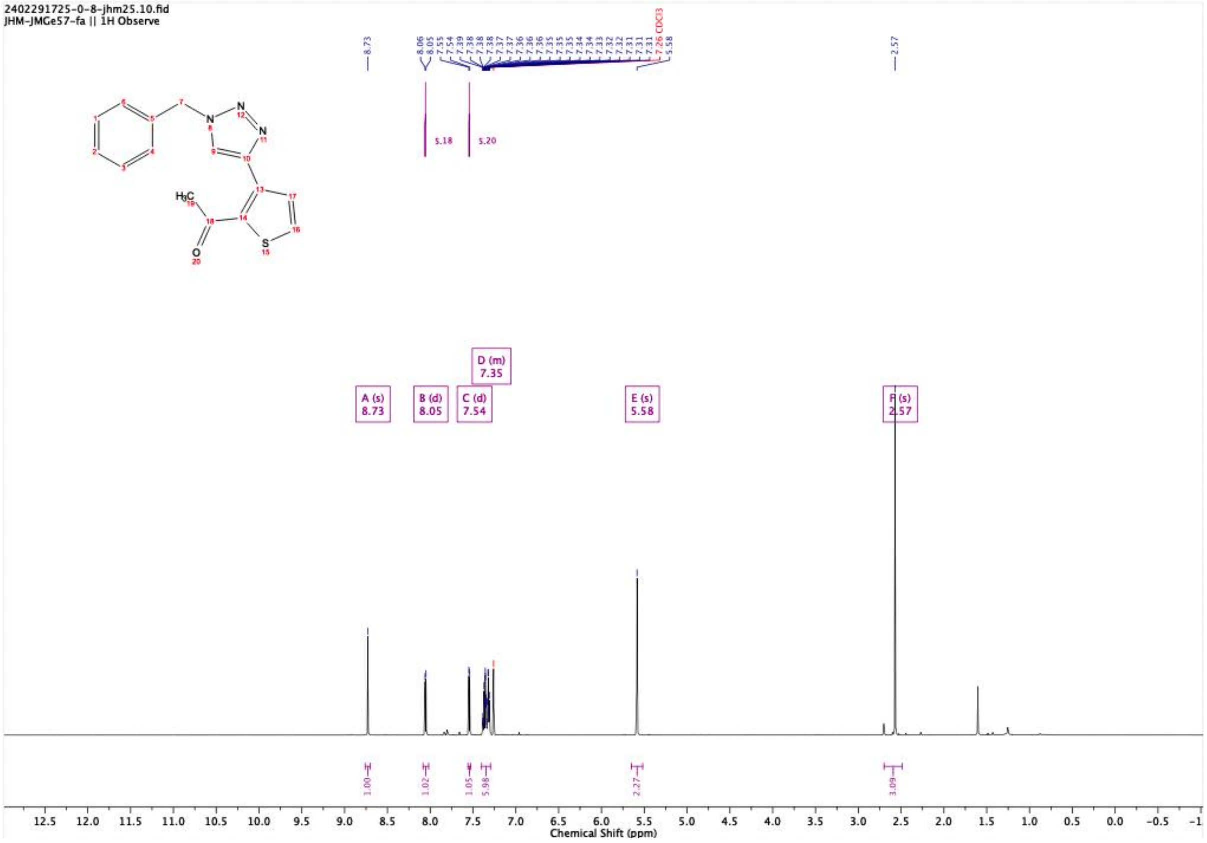

^{13}C DEPTQ

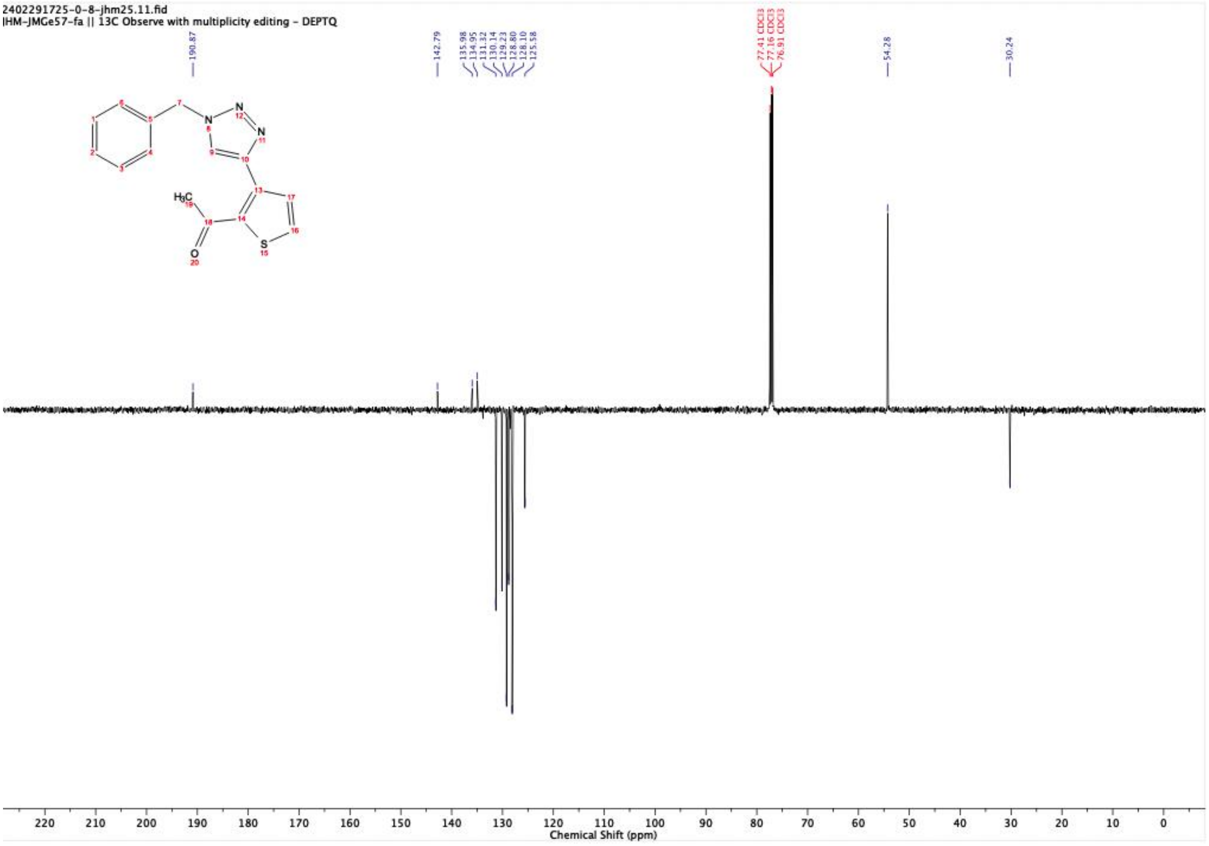

31 – ^{1}H

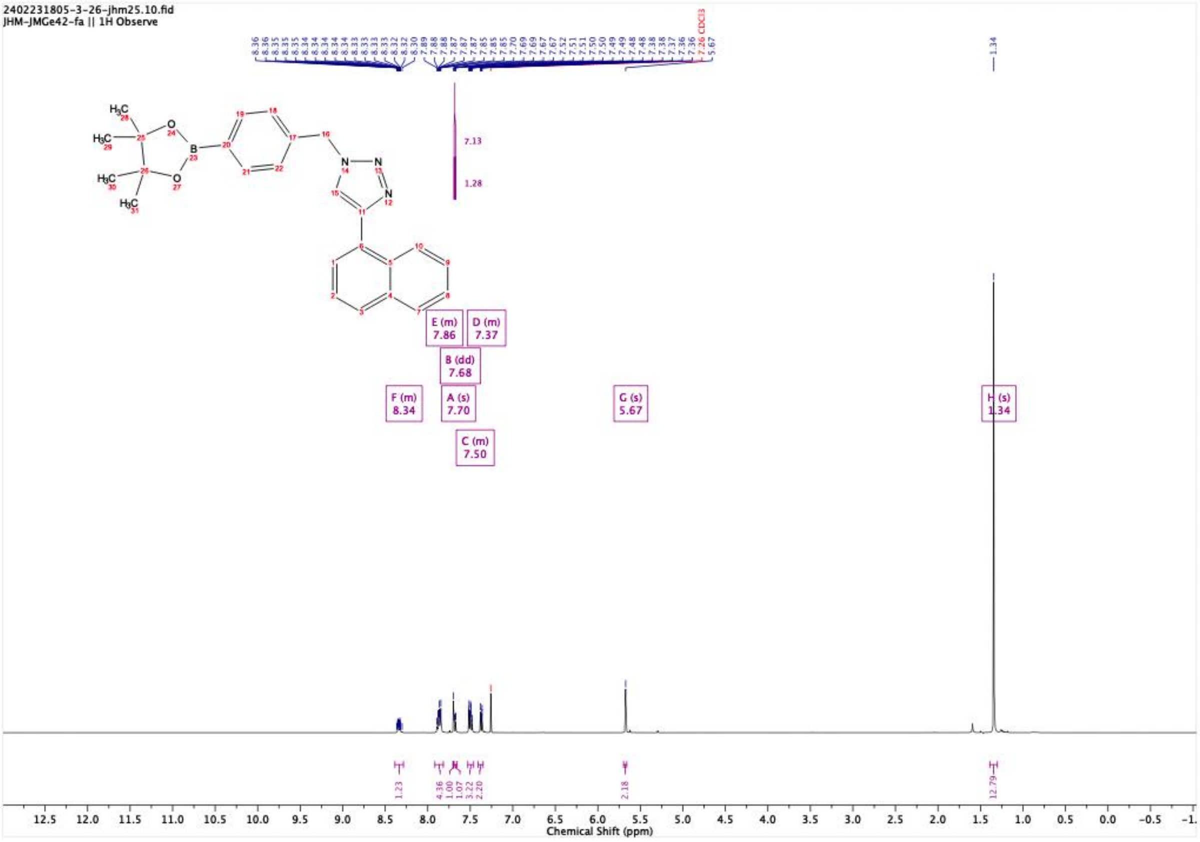

^{13}C DEPTQ

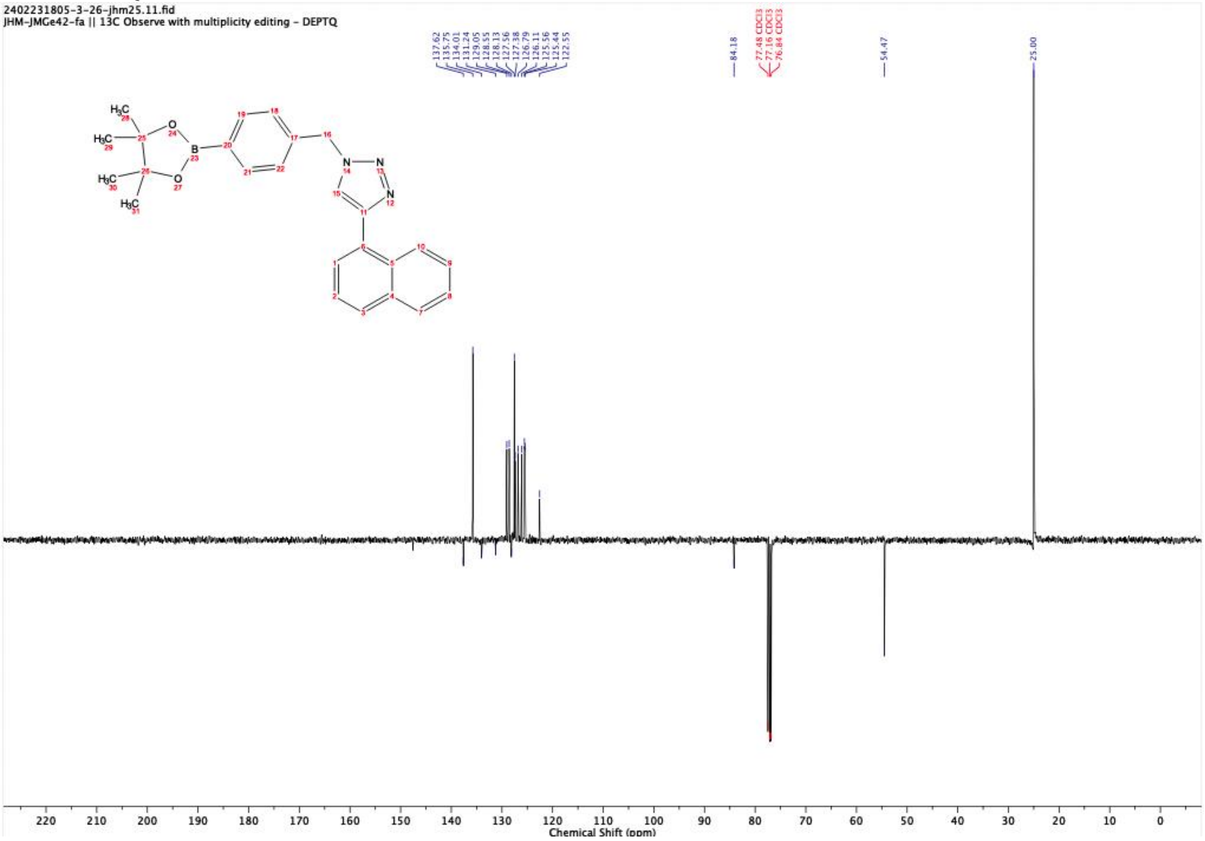

[11]B

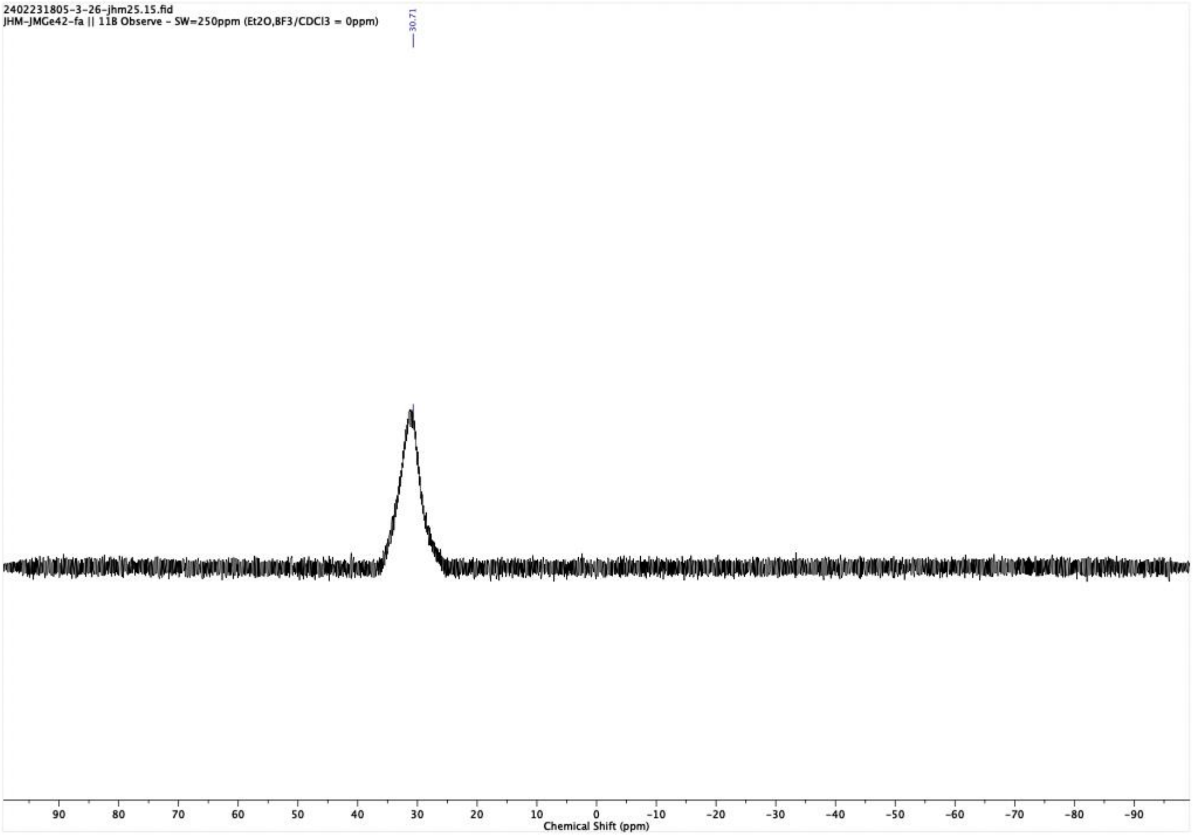
2402231805-3-26-jhm25.15.fid
JHM-JMGe42-fa || 11B Observe - SW=250ppm (Et2O,BF3/CDCl3 = 0ppm)
30.71
90
80
70
60
50
40
30
20
10
0
-10
-20
-30
-40
-50
-60
-70
-80
-90
Chemical Shift (ppm)

32 – ^{1}H

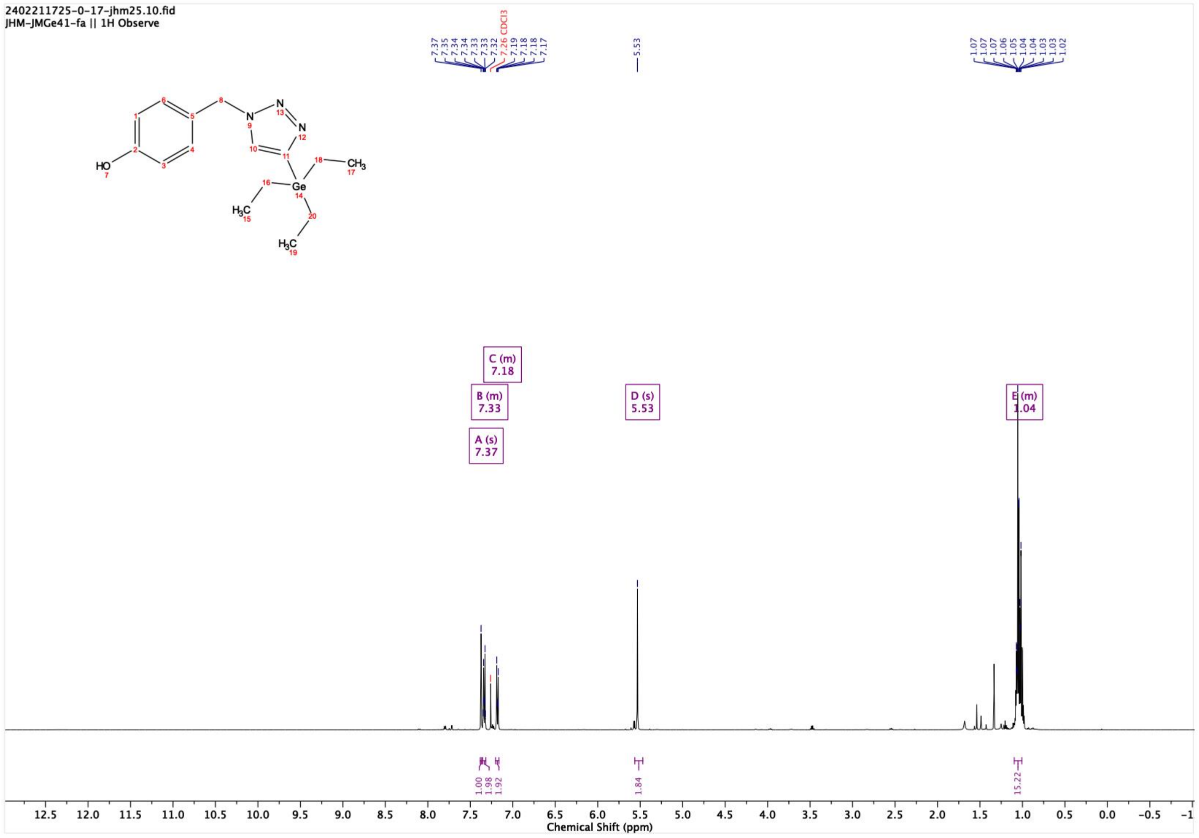

^{13}C DEPTQ

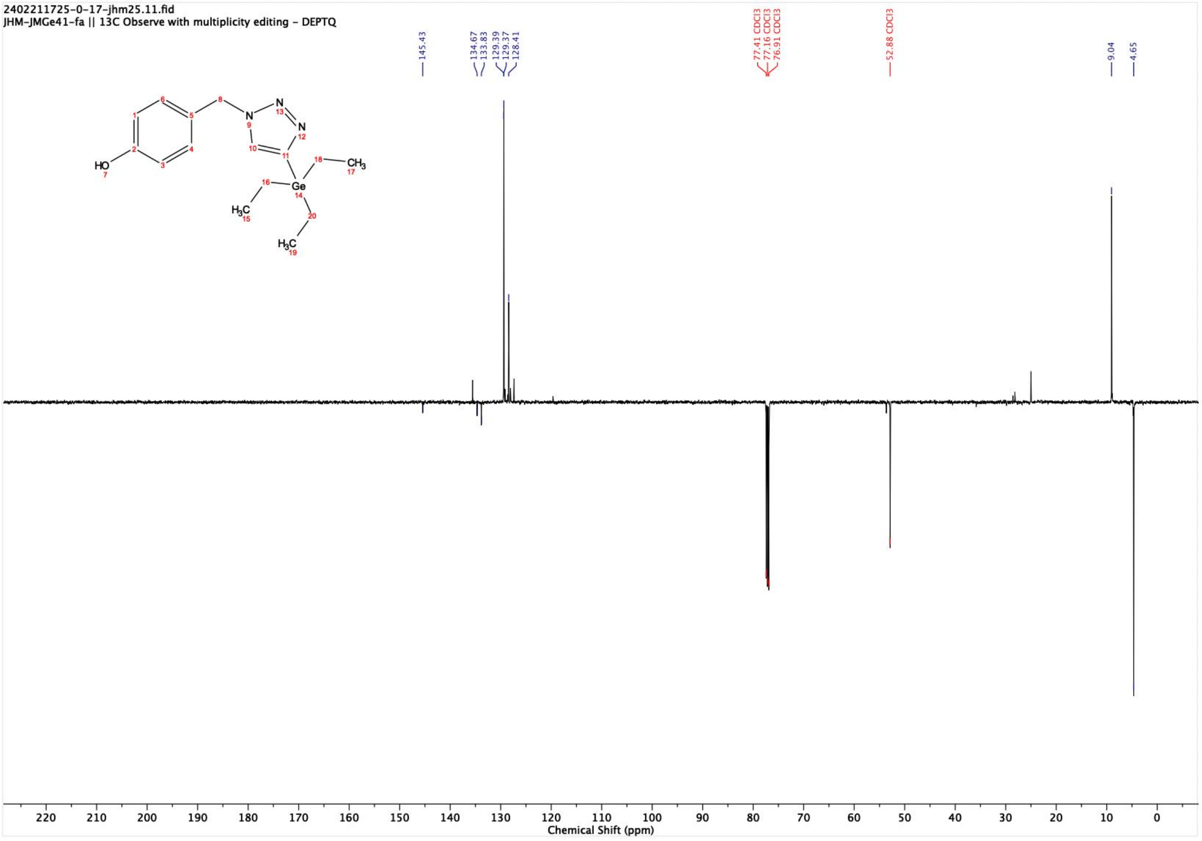

33 – ^{1}H

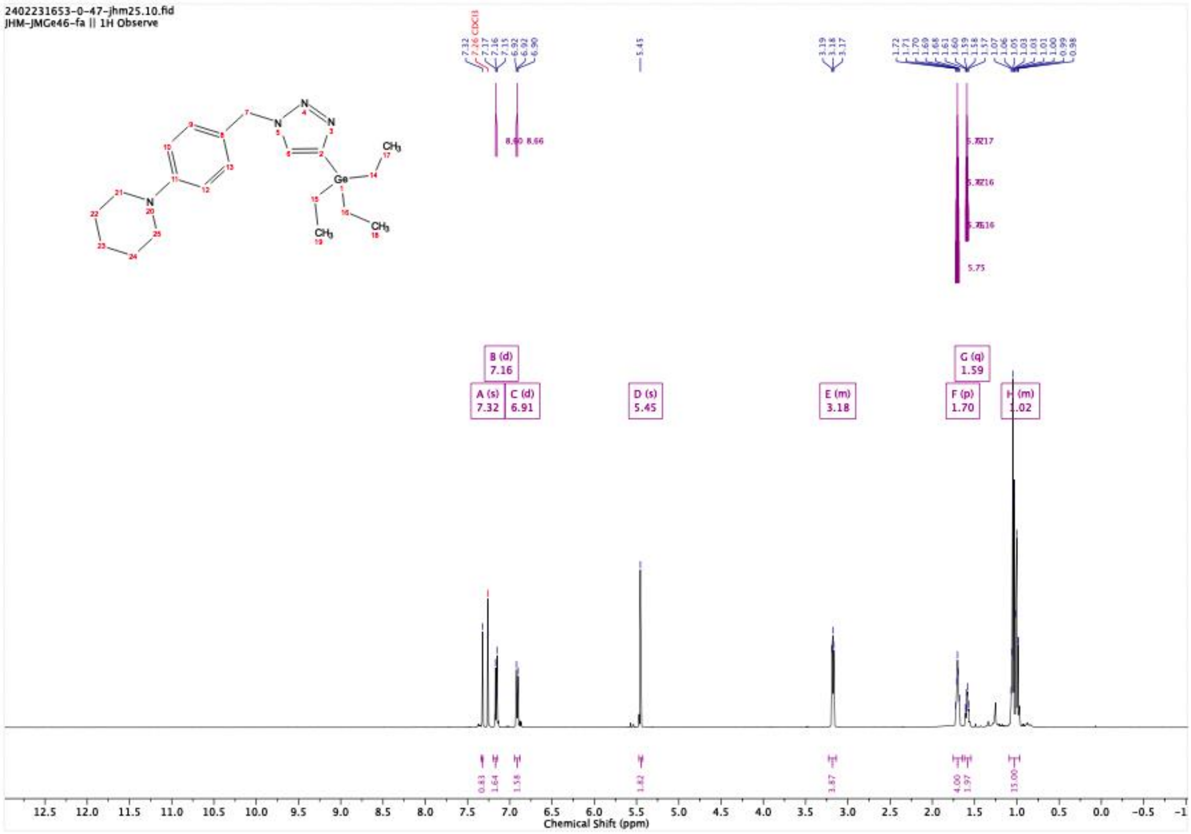

^{13}C DEPTQ

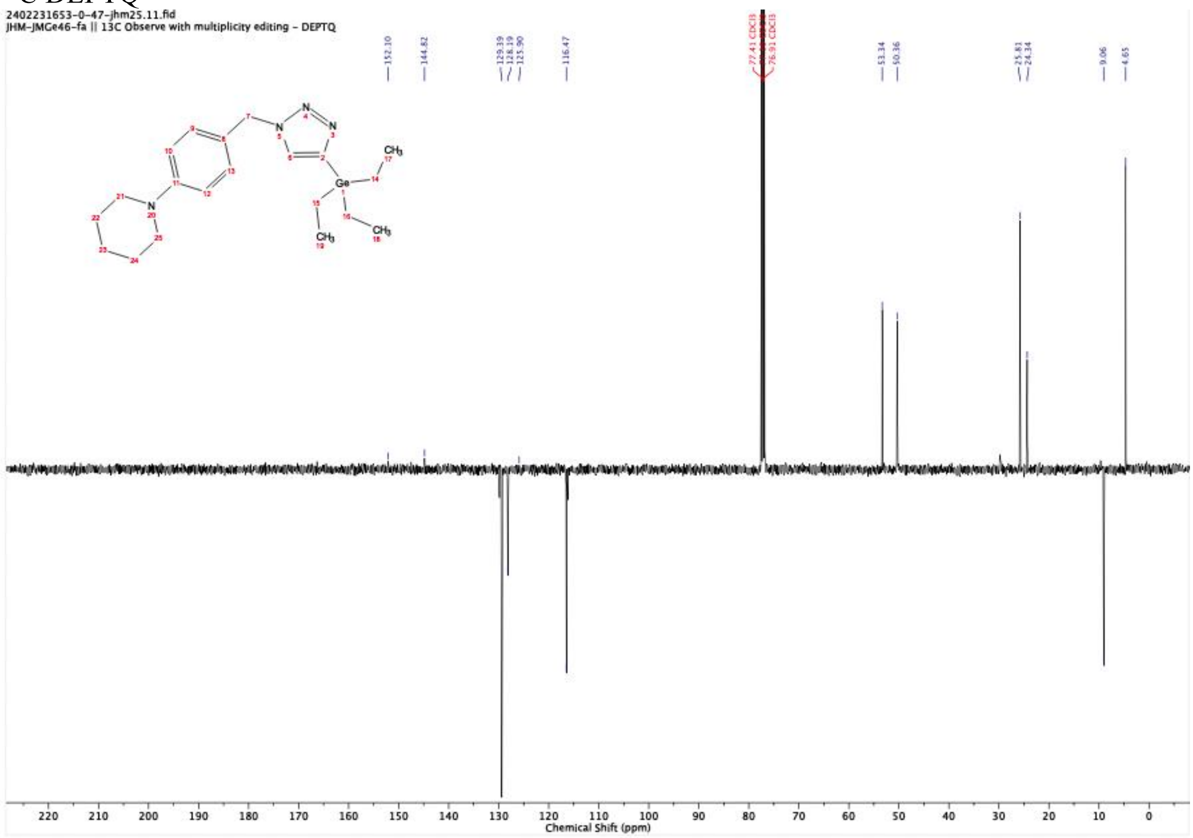

34 – ^{1}H

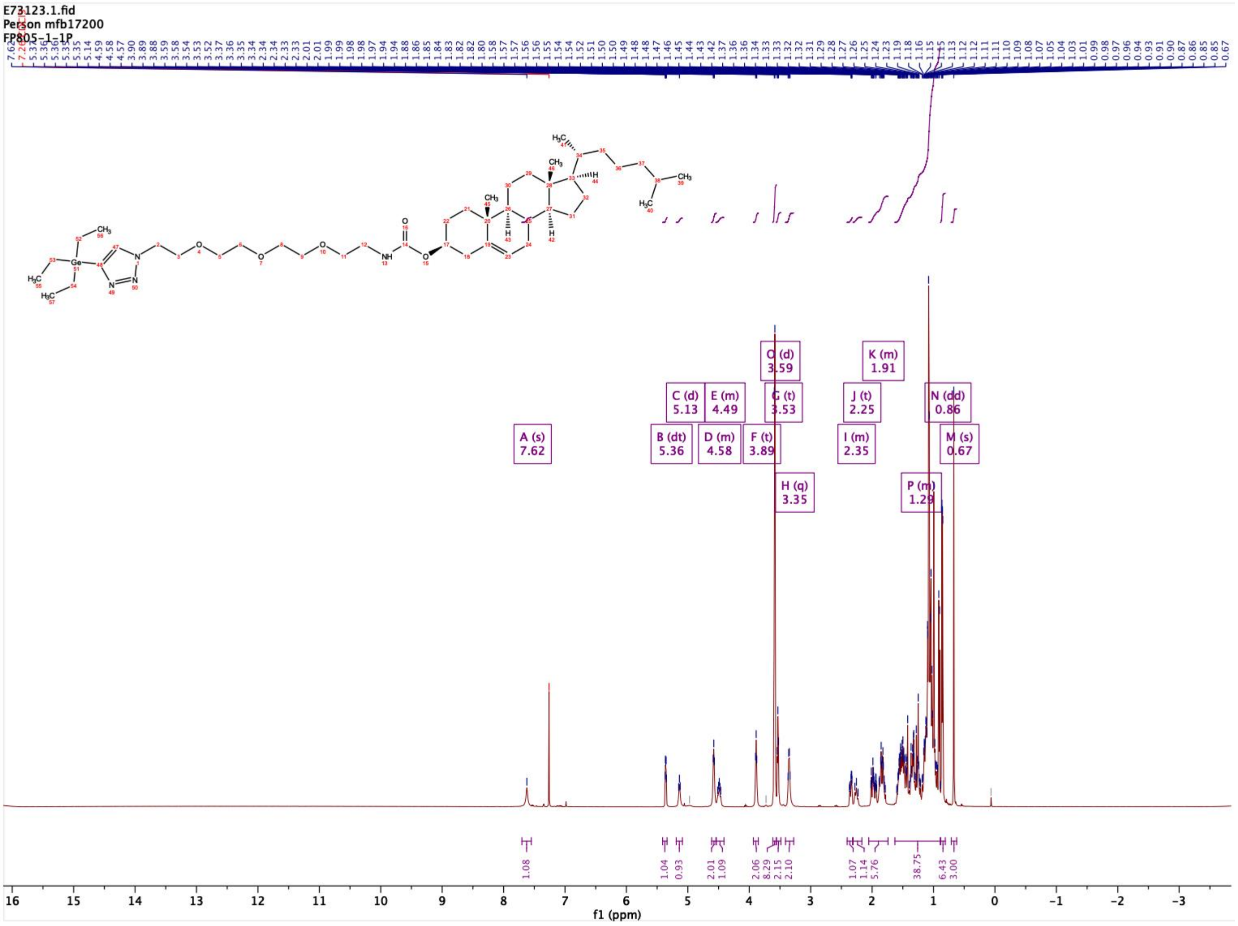
E73123.1.fid
Person mfb17200
FP805-1-1P
f1 (ppm)

^{13}C

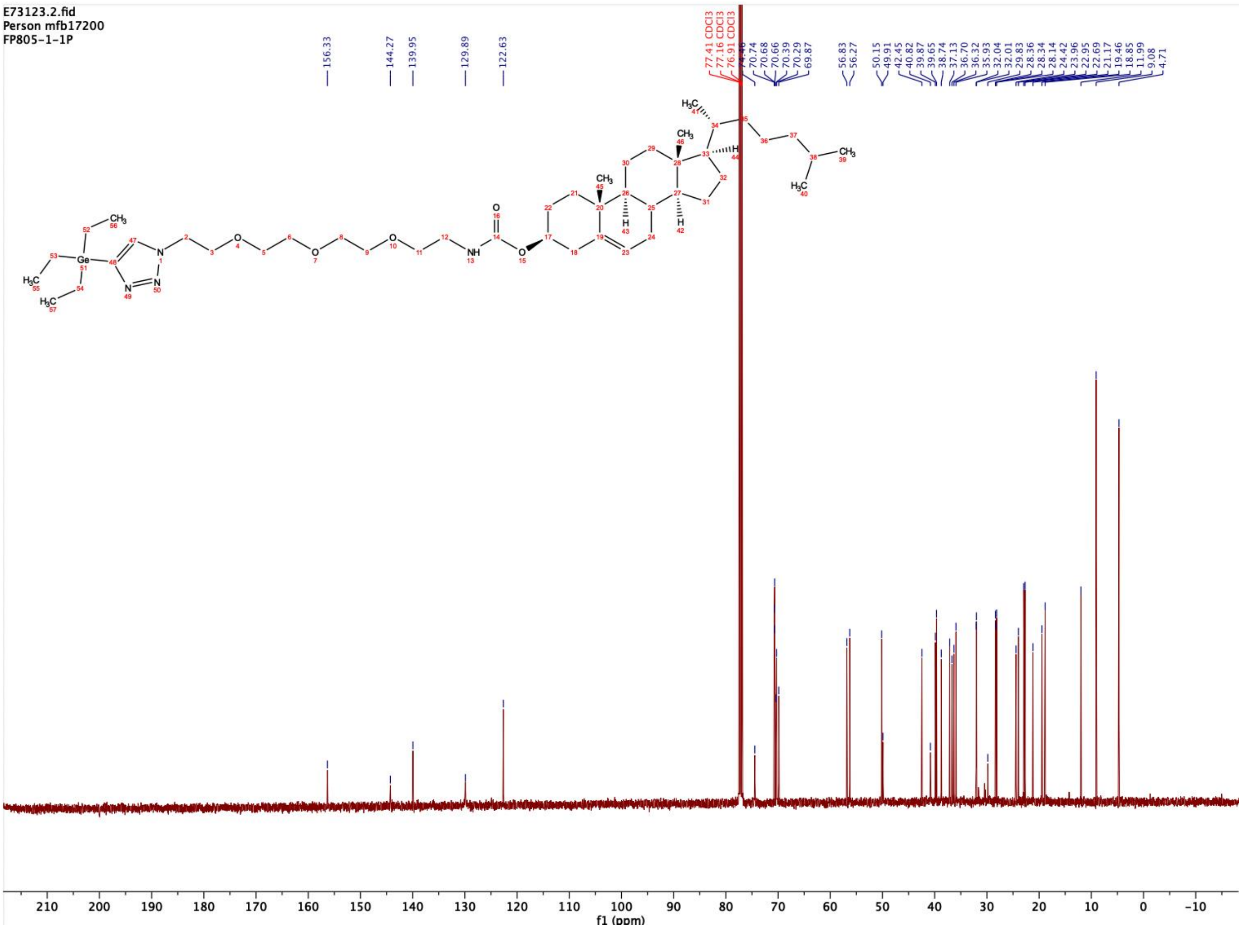
E73123.2.fid
Person mfb17200
FP805-1-1P
f1 (ppm)

35 – ^{1}H

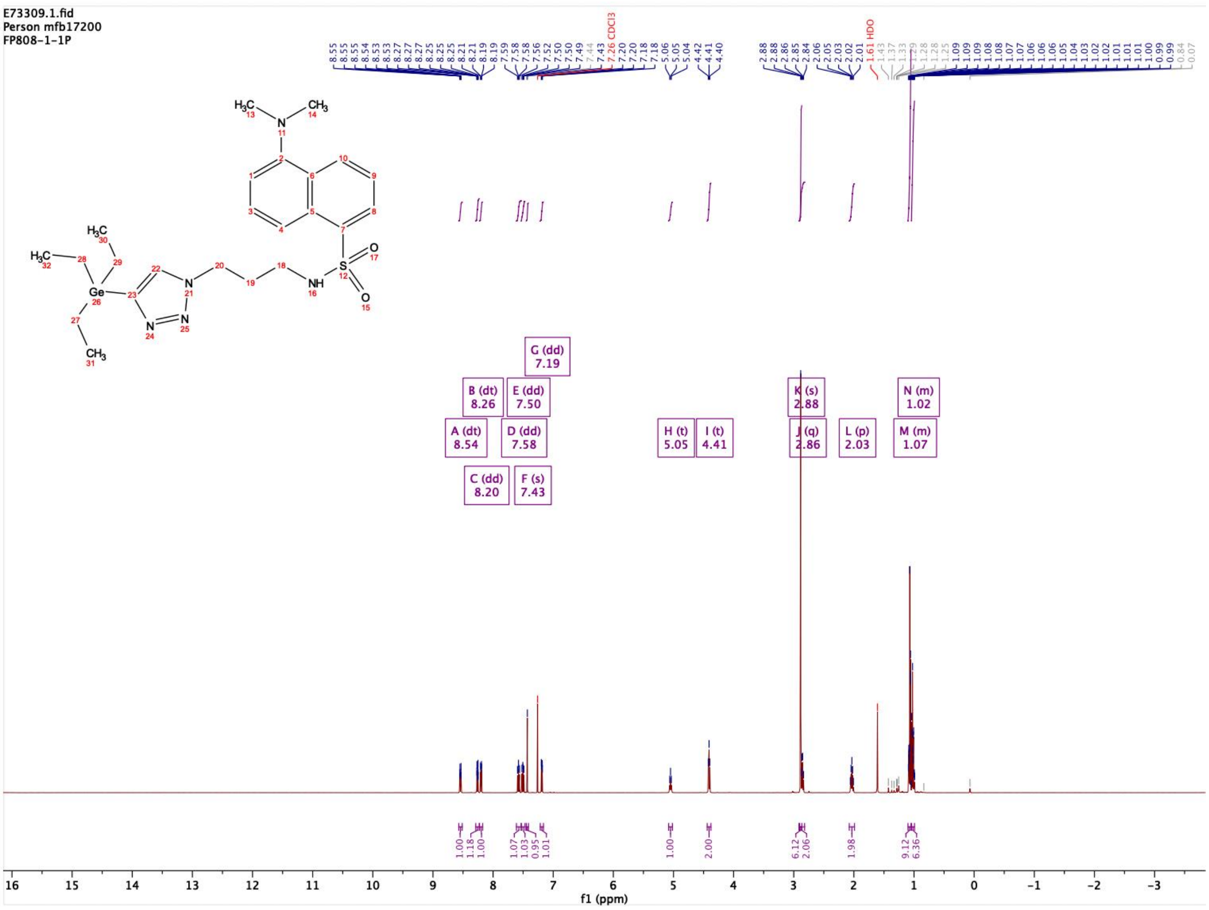

E73309.1.fid
Person mfb17200
FP808-1-1P
A (dt) 8.54
B (dt) 8.26
C (dd) 8.20
D (dd) 7.58
E (dd) 7.50
F (s) 7.43
G (dd) 7.19
H (t) 5.05
I (t) 4.41
J (q) 2.86
K (s) 2.88
L (p) 2.03
M (m) 1.07
N (m) 1.02
f1 (ppm)

^{13}C

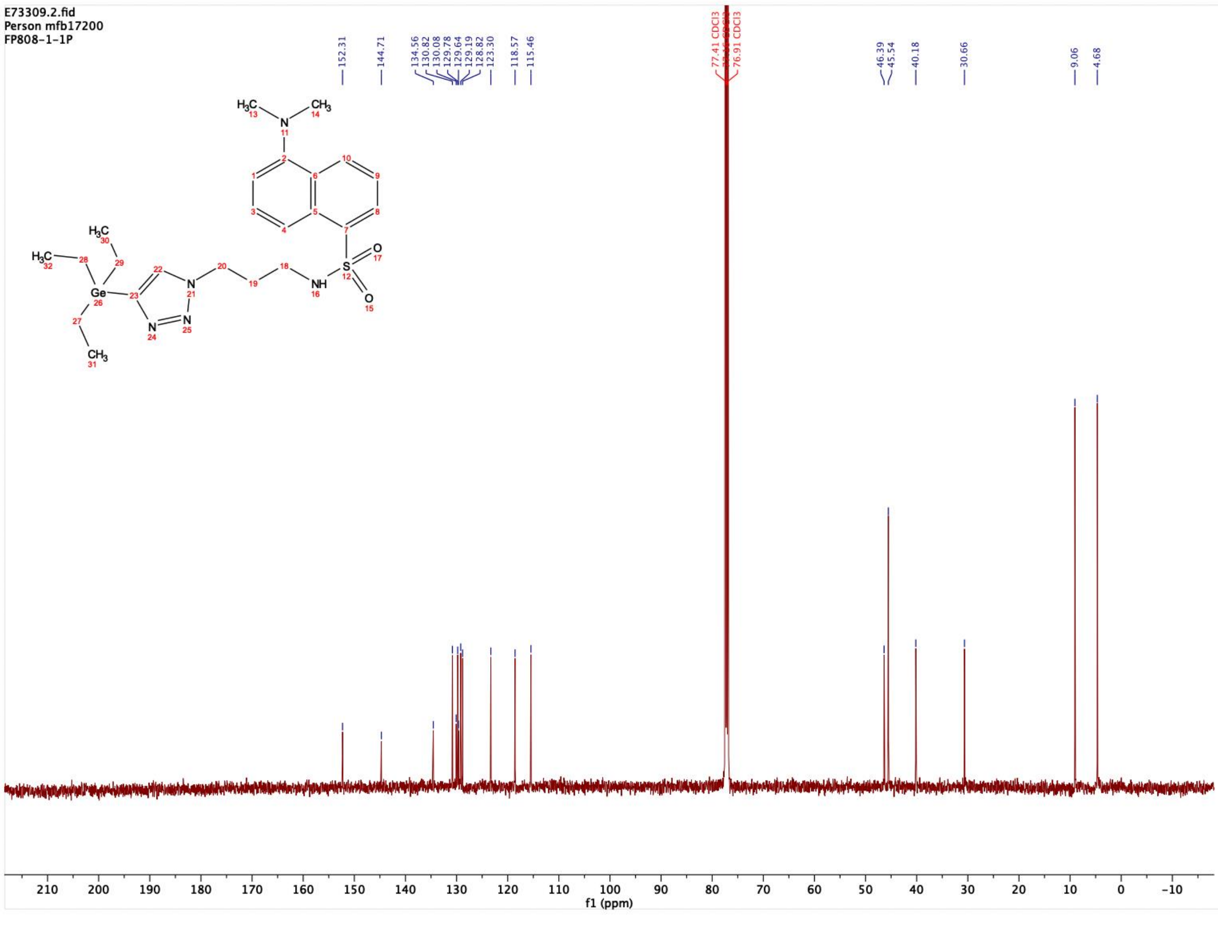

E73309.2.fid
Person mfb17200
FP808-1-1P
152.31
144.71
134.56
130.82
130.08
129.78
129.64
129.19
128.82
123.30
118.57
115.46
77.41 CDCl3
76.91 CDCl3
46.39
45.54
40.18
30.66
9.06
4.68
f1 (ppm)
