# Supplementary material for: Germanyl triazoles as a platform for CuAAC diversification and chemoselective orthogonal cross-coupling
Source: Beilstein J Org Chem. 2024 Dec 5;20:3198–204. doi: 10.3762/bjoc.20.265 (PMC11635283; doi:10.3762/bjoc.20.265)

BEILSTEIN JOURNAL OF ORGANIC CHEMISTRY

Supporting Information

for

Germanyl triazoles as a platform for CuAAC diversification and chemoselective orthogonal cross-coupling

John M. Halford-McGuff, Thomas M. Richardson, Aidan P. McKay, Frederik Peschke, Glenn A. Burley and Allan J. B. Watson

Beilstein J. Org. Chem. **2024,** *20,* 3198–3204. doi:10.3762/bjoc.20.265

Checkcif file for compound 13

License and Terms: This is a supporting information file under the terms of the Creative Commons Attribution License (https://creativecommons.org/licenses/by/4.0). Please note that the reuse, redistribution and reproduction in particular requires that the author(s) and source are credited and that individual graphics may be subject to special legal provisions.
The license is subject to the *Beilstein Journal of Organic Chemistry* terms and conditions: (https://www.beilstein-journals.org/bjoc/terms)

checkCIF/PLATON report

Structure factors have been supplied for datablock(s) 13

THIS REPORT IS FOR GUIDANCE ONLY. IF USED AS PART OF A REVIEW PROCEDURE FOR PUBLICATION, IT SHOULD NOT REPLACE THE EXPERTISE OF AN EXPERIENCED CRYSTALLOGRAPHIC REFEREE.

No syntax errors found. CIF dictionary Interpreting this report

Datablock: 13

Bond precision:	C-C = 0.0129 A		Wavelength=0.71073
Cell:	a=7.19194(13)	b=15.2354(4)	c=42.5349(9)
	alpha=90	beta=90	gamma=90
Temperature:	100 K		

	Calculated	Reported
Volume	4660.64(18)	4660.64(18)
Space group	P 21 21 21	P 21 21 21
Hall group	P 2ac 2ab	P 2ac 2ab
Moiety formula	C28 H23 Ge N3 O	C28 H23 Ge N3 O
Sum formula	C28 H23 Ge N3 O	C28 H23 Ge N3 O
Mr	490.10	490.08
Dx,g cm-3	1.397	1.397
Z	8	8
Mu (mm-1)	1.340	1.340
F000	2016.0	2016.0
F000'	2017.76	
h,k,lmax	9,20,58	9,20,53
Nref	12594[7057]	10795
Tmin,Tmax	0.886,0.987	0.646,1.000
Tmin'	0.886	

Correction method= # Reported T Limits: Tmin=0.646 Tmax=1.000
AbsCorr = MULTI-SCAN

Data completeness= 1.53/0.86 Theta(max)= 29.184

R(reflections)= 0.0524(8363) wR2(reflections)= 0.1014(10795)

S = 1.091 Npar= 596

The following ALERTS were generated. Each ALERT has the format
test-name_ALERT_alert-type_alert-level.
Click on the hyperlinks for more details of the test.

Alert level C

```
STRVA01_ALERT_4_C           Flack test results are ambiguous.
           From the CIF: _refine_ls_abs_structure_Flack    0.490
           From the CIF: _refine_ls_abs_structure_Flack_su    0.030
PLAT213_ALERT_2_C Atom C10            has ADP max/min Ratio .....        3.5 oblate
PLAT220_ALERT_2_C NonSolvent   Resd 1  C   Ueq(max)/Ueq(min) Range       4.7 Ratio
PLAT220_ALERT_2_C NonSolvent   Resd 2  C   Ueq(max)/Ueq(min) Range       4.7 Ratio
PLAT341_ALERT_3_C Low Bond Precision on  C-C Bonds ..............   0.01285 Ang.
PLAT906_ALERT_3_C Large K Value in the Analysis of Variance ......     3.030 Check
```

Alert level G

```
PLAT003_ALERT_2_G Number of Uiso or Uij Restrained non-H Atoms ...          5 Report
PLAT033_ALERT_4_G Flack x Value Deviates > 3.0 * sigma from Zero .      0.490 Note
PLAT083_ALERT_2_G SHELXL Second Parameter in WGHT  Unusually Large       9.73 Why ?
PLAT111_ALERT_2_G ADDSYM Detects New (Pseudo) Centre of Symmetry .        100 %Fit
PLAT112_ALERT_2_G ADDSYM Detects New (Pseudo) Symm. Elem         c        100 %Fit
PLAT112_ALERT_2_G ADDSYM Detects New (Pseudo) Symm. Elem         a        100 %Fit
PLAT112_ALERT_2_G ADDSYM Detects New (Pseudo) Symm. Elem         b        100 %Fit
PLAT113_ALERT_2_G ADDSYM Suggests Possible Pseudo/New  Space Group       Pbca Check
              Check Model Parameter Symmetry for Reflection Data Support
PLAT186_ALERT_4_G The CIF-Embedded .res File Contains ISOR Records          4 Report
PLAT187_ALERT_4_G The CIF-Embedded .res File Contains RIGU Records          4 Report
PLAT860_ALERT_3_G Number of Least-Squares Restraints ............         144 Note
PLAT910_ALERT_3_G Missing # of FCF Reflection(s) Below Theta(Min).          4 Note
                0  1  1,   0  0  2,   0  1  2,   0  0  4,
PLAT912_ALERT_4_G Missing # of FCF Reflections Above STh/L=  0.600        667 Note
PLAT933_ALERT_2_G Number of HKL-OMIT Records in Embedded .res File          2 Note
                0  0  4,   0  1  2,
PLAT952_ALERT_5_G Calculated (ThMax) and CIF-Reported Lmax Differ.          5 Units
PLAT958_ALERT_1_G Calculated (ThMax) and Actual (FCF) Lmax Differ.          5 Units
PLAT969_ALERT_5_G The 'Henn et al.' R-Factor-gap value ..........        2.43 Note
              Predicted wR2: Based on SigI**2  4.18 or SHELX Weight  9.57
PLAT978_ALERT_2_G Number C-C Bonds with Positive Residual Density.          0 Info
```

0 **ALERT level A** = Most likely a serious problem - resolve or explain
0 **ALERT level B** = A potentially serious problem, consider carefully
6 **ALERT level C** = Check. Ensure it is not caused by an omission or oversight
18 **ALERT level G** = General information/check it is not something unexpected

1 ALERT type 1 CIF construction/syntax error, inconsistent or missing data
12 ALERT type 2 Indicator that the structure model may be wrong or deficient
4 ALERT type 3 Indicator that the structure quality may be low
5 ALERT type 4 Improvement, methodology, query or suggestion
2 ALERT type 5 Informative message, check

It is advisable to attempt to resolve as many as possible of the alerts in all categories. Often the minor alerts point to easily fixed oversights, errors and omissions in your CIF or refinement strategy, so attention to these fine details can be worthwhile. In order to resolve some of the more serious problems it may be necessary to carry out additional measurements or structure refinements. However, the purpose of your study may justify the reported deviations and the more serious of these should normally be commented upon in the discussion or experimental section of a paper or in the "special_details" fields of the CIF. checkCIF was carefully designed to identify outliers and unusual parameters, but every test has its limitations and alerts that are not important in a particular case may appear. Conversely, the absence of alerts does not guarantee there are no aspects of the results needing attention. It is up to the individual to critically assess their own results and, if necessary, seek expert advice.

Publication of your CIF in IUCr journals

A basic structural check has been run on your CIF. These basic checks will be run on all CIFs submitted for publication in IUCr journals (*Acta Crystallographica*, *Journal of Applied Crystallography*, *Journal of Synchrotron Radiation*); however, if you intend to submit to *Acta Crystallographica Section C* or *E* or *IUCrData*, you should make sure that full publication checks are run on the final version of your CIF prior to submission.

Publication of your CIF in other journals

Please refer to the *Notes for Authors* of the relevant journal for any special instructions relating to CIF submission.

PLATON version of 06/01/2024; check.def file version of 05/01/2024

Datablock 13 - ellipsoid plot

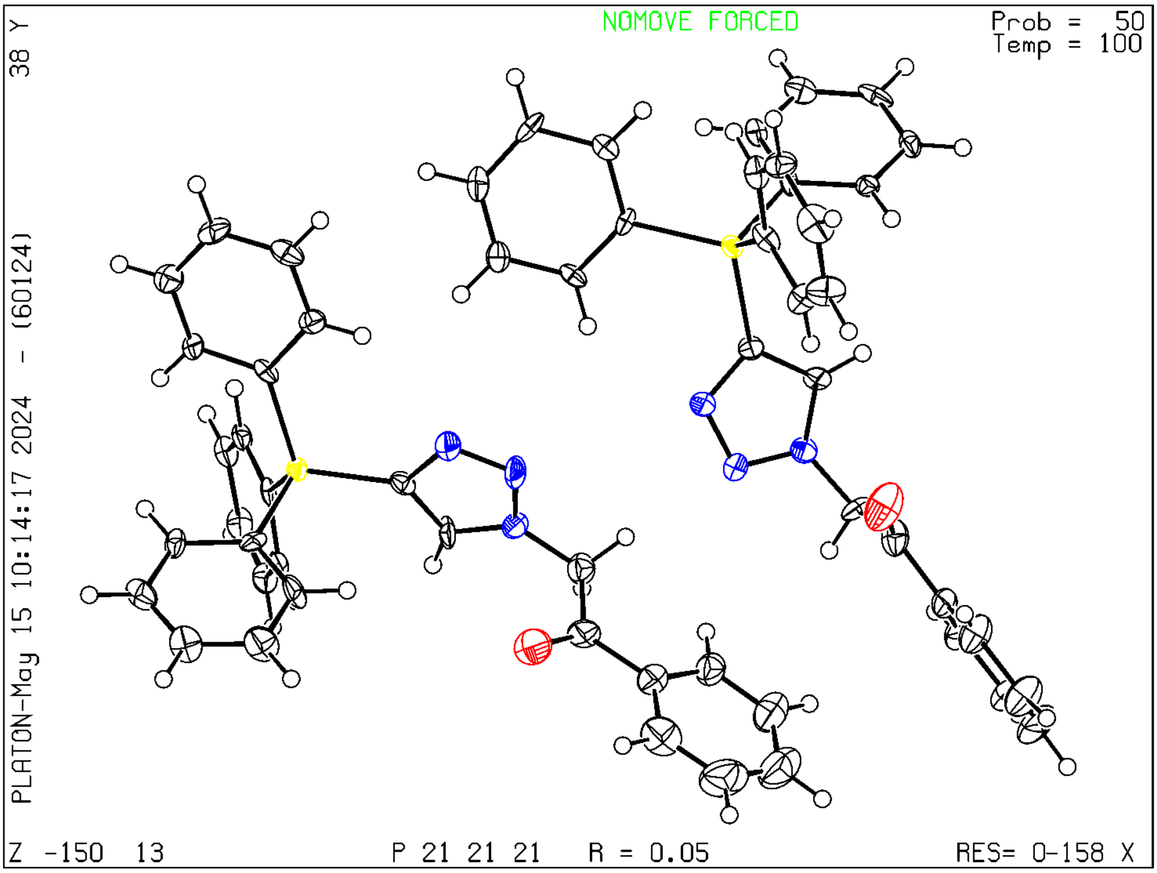

Supplement: File 3 — Checkcif file for compound 13. [file Beilstein_J_Org_Chem-20-3198-s003.pdf]
